# Causal association between circulating inflammatory cytokines and intracranial aneurysm and subarachnoid hemorrhage

**DOI:** 10.1111/ene.16326

**Published:** 2024-05-06

**Authors:** Qiang He, Wenjing Wang, Yang Xiong, Chuanyuan Tao, Lu Ma, Chao You

**Affiliations:** ^1^ Department of Neurosurgery, West China Hospital Sichuan University Chengdu Sichuan China; ^2^ Department of Pharmacy, Institute of Metabolic Diseases and Pharmacotherapy, West China Hospital Sichuan University Chengdu Sichuan China; ^3^ Department of Urology, West China Hospital Sichuan University Chengdu Sichuan China

**Keywords:** causal association, inflammatory cytokines, intracranial aneurysm, Mendelian randomization, subarachnoid hemorrhage

## Abstract

**Background and purpose:**

The causal association between inflammatory cytokines and the development of intracranial aneurysm (IA), unruptured IA (uIA) and subarachnoid hemorrhage (SAH) lacks clarity.

**Methods:**

The summary‐level datasets for inflammatory cytokines were extracted from a genome‐wide association study of the Finnish Cardiovascular Risk in Young Adults Study and the FINRISK survey. The summary statistics datasets related to IA, uIA and SAH were obtained from the genome‐wide association study meta‐analysis of the International Stroke Genetics Consortium and FinnGen Consortium. The primary method employed for analysis was inverse variance weighting (false discovery rate), supplemented by sensitivity analyses to address pleiotropy and enhance robustness.

**Results:**

In the International Stroke Genetics Consortium, 10, six and eight inflammatory cytokines exhibited a causal association with IA, uIA and SAH, respectively (false discovery rate, *p* < 0.05). In FinnGen datasets, macrophage Inflammatory Protein‐1 Alpha (MIP_1A), MIP_1A and interferon γ‐induced protein 10 (IP_10) were verified for IA, uIA and SAH, respectively. In the reverse Mendelian randomization analysis, the common cytokines altered by uIA and SAH were vascular endothelial growth factor (VEGF), MIP_1A, IL_9, IL_10 and IL_17, respectively. The meta‐analysis results show that MIP_1A and IP_10 could be associated with the decreased risk of IA, and MIP_1A and IP_10 were associated with the decreased risk of uIA and SAH, respectively. Notably, the levels of VEGF, MIP_1A, IL_9, IL_10 and TNF_A were increased with uIA. Comprehensive heterogeneity and pleiotropy analyses confirmed the robustness of these results.

**Conclusion:**

Our study unveils a bidirectional association between inflammatory cytokines and IA, uIA and SAH. Further investigations are essential to validate their relationship and elucidate the underlying mechanisms.

## INTRODUCTION

Intracranial aneurysm (IA), characterized by balloon‐shaped dilatation, has a worldwide prevalence of 3.2% in the general population [1]. The rupture of IA causing subarachnoid hemorrhage (SAH) is a fatal subtype of stroke, which kills up to one‐third of patients within 24 h and leads to severe neurological deficits in survivors [2]. IA development is influenced by genetic and environmental factors, with inflammation playing a crucial role in its pathogenesis and rupture [3]. Notably, targeting various inflammatory cytokines in preclinical models has demonstrated efficacy and shows promise for interventions [4, 5].

Cytokines, as crucial signaling proteins, play a pivotal role in inflammation associated with IA, IA rupture and SAH. Specific cytokines can have either pro‐inflammatory or anti‐inflammatory effects. For instance, patients with SAH exhibit significant changes in cytokine levels in brain tissue and cerebrospinal fluid (CSF) [6, 7]. The elevated levels of interleukin (IL) 6 and tumor necrosis factor alpha (TNF‐α) in CSF post‐SAH are linked to the risk of cerebral vasospasm and neurological deficits [8]. Conversely, increased CSF levels of the anti‐inflammatory cytokine IL‐4 are associated with favorable SAH outcomes [9]. Moreover, studies indicate that elevated cytokine levels in blood plasma and serum signal systemic inflammatory processes [10]. Elevated levels of IL‐1β, monocyte chemoattractant protein 1 (MCP‐1) and TNF‐α in plasma are correlated with the risk of IA development [11]. Endothelial dysfunction, induced by intercellular adhesion molecule 1 (ICAM1) and vascular cell adhesion protein 1 (VCAM1), smooth muscle cell loss from TNF‐α and IL‐1β, and cell death caused by matrix metalloproteinase and TNF‐α collectively contribute to the progressive weakening of the arterial wall, leading to dilatation, aneurysm formation and eventual rupture [12]. Despite these insights, a comprehensive study investigating systemic inflammation's effects on IA, unruptured IA (uIA) and SAH is currently lacking. Additionally, controversy persists regarding whether systemic inflammation is a cause or a downstream effect of IA, uIA and SAH. The specific roles of inflammatory cytokines, whether pro‐ or anti‐inflammatory, need clarification. Importantly, observational studies may have inherent limitations, such as a restricted sample size and retrospective design, which could compromise their ability to reflect a genuine causal association.

Mendelian randomization (MR) is a robust and effective method that utilizes genetic variants (single‐nucleotide polymorphisms, SNPs) to investigate the causal relationship between inflammatory cytokines and IA, uIA and SAH [13]. SNPs, guided by the random principles of meiosis, undergo random assortment during zygote formation in gestation [12, 14], rendering the results of MR analyses resilient against reverse causality and confusion. Previous studies leveraging MR analysis have successfully identified causal relationships between inflammatory cytokines and various neurological diseases, including stroke [15], Parkinson's disease [16] and epilepsy [17]. Despite these advancements, a comprehensive analysis of the causal association between inflammatory cytokines and the risk of IA, uIA and SAH is yet to be undertaken. Therefore, this two‐sample bidirectional and replicate MR study aims to elucidate the potential causal connections between individual inflammatory cytokines and the development of IA, uIA and SAH. The findings may offer actionable insights into the roles of cytokines and provide evidence supporting the targeted treatment of specific inflammatory cytokines for IA, uIA and SAH.

## METHODS

### Ethical approval and study design

**FIGURE 1:**
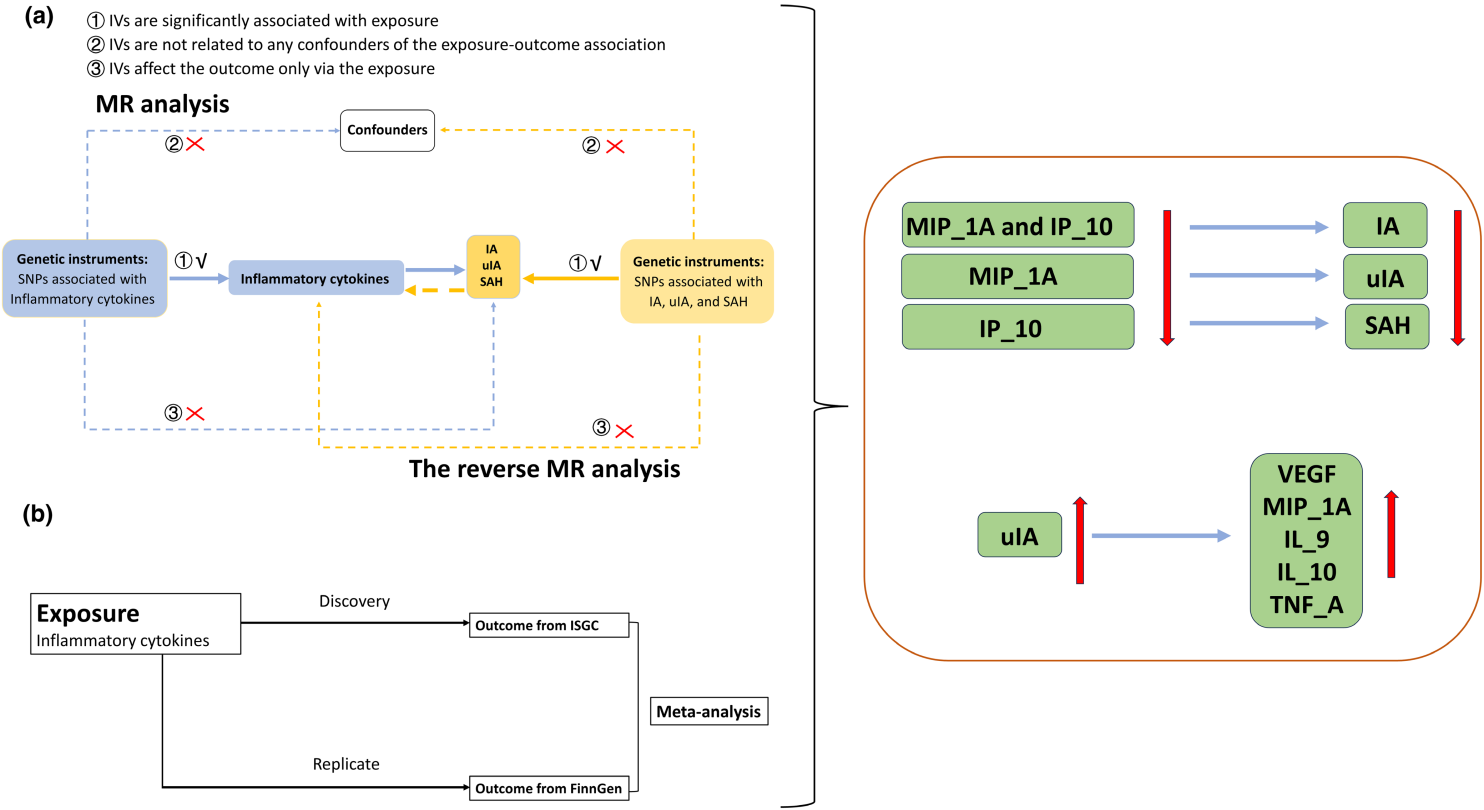
Study design of the bidirectional two‐sample MR for the causal association between genetically predicted inflammatory cytokines and IA, uIA and SAH. IA, intracranial aneurysm; ISGC, International Stroke Genetics Consortium; IV, instrumental variable; MR, Mendelian randomization; SAH, subarachnoid hemorrhage; SNP, single nucleotide polymorphism; uIA, unruptured intracranial aneurysm.

The summary‐level statistics for inflammatory cytokines associated with IA, uIA and SAH were obtained from publicly available, de‐identified datasets and previously approved studies. Relevant ethics committee approvals and the informed consent of participants have been obtained. Ethical approval is exempted for this MR study, as it solely utilizes summary‐level datasets. The study adheres to the Strengthening the Reporting of Observational Studies in Epidemiology using Mendelian Randomization (STROBE‐MR) checklist [18, 19], and the study flowchart is displayed in Figure 1.

### Data sources of 41 circulating inflammatory cytokines

The summary‐level datasets for the 41 circulating inflammatory cytokines were derived from the re‐analyzed outcomes of a serum cytokine‐associated genome‐wide association study (GWAS) (https://data.bris.ac.uk/data/dataset/3074krb6t2frj29yh2b03x3wxj) [20]. The datasets encompass information from the Finnish Cardiovascular Risk in Young Adults Study (*N* = 1980; average age 37.4 years for males, 37.5 years for females) and the FINRISK survey (FINRISK1997 *N* = 4608, average age 48.3 years for males, 47.3 years for females; FINRISK2002 *N* = 1705, average age 60.4 years for males, 60.1 years for females). A total of 8293 participants of European ancestry were registered from 1980 to 2011. Cytokine levels were recorded at baseline when participants visited the assessment center. Using Bio‐Rad's premixed Bio‐Plex Pro Human Cytokine 27‐plex Assay and 21‐plex Assay, a total of 41 inflammatory cytokines were quantified and analyzed with the Bio‐Plex 200 reader featuring Bio‐Plex 6.0 software [20]. Primary covariates, including age, sex and the top 10 genetic principal components, were adjusted. The sources of circulating inflammatory cytokines are displayed in Table S1.

### Data sources of IA, uIA and SAH

The summary‐level datasets for IA (7495 cases and 71,934 controls), SAH (5140 cases and 71,934 controls) and uIA (2070 cases and 71,934 controls) were extracted from the GWAS meta‐analysis conducted by the International Stroke Genetics Consortium (ISGC) [21]. Access to the public website is available through the Cerebrovascular Disease Knowledge Portal (www.cerebrovascularportal.org). IA includes both unruptured and ruptured cases, with proportions divided into ruptured (69%), unruptured (28%) and unknown rupture status (3.8%). Detailed sources for IA, uIA and SAH are presented in Table S1.

The replicate summary statistics datasets for IA, uIA and SAH were acquired from the FinnGen Consortium [22]. The public website is https://r7.risteys.finngen.fi/. This dataset includes 960 IA cases and 284,164 controls, 1519 uIA cases and 284,164 controls, and 3201 SAH cases and 284,164 controls. Comprehensive sources for IA, uIA and SAH in the FinnGen Consortium are detailed in Table S1.

There is no overlap between the exposure and outcome datasets. In cases where index SNPs for the exposure were not available in the outcome dataset, they were substituted with proxy SNPs (*r* ^2^ > 0.8) identified using 1000 genomes of European reference data.

### Selection of genetic instrument selection

Initially, SNPs associated with circulating inflammatory cytokines were selected at a genome‐wide significance level of *p* < 5 × 10^−8^. However, due to the limited number of SNPs selected, genetic instruments associated with inflammatory cytokines were chosen at a locus‐wide significance level (*p* < 5 × 10^−6^). Independent SNPs were obtained using the threshold of *r* ^2^ < 0.001 and a clumping window of 10,000 kb, with the European population as a reference. The instrumental variables (IVs) are detailed in Table S2. The MR Pleiotropy RESidual Sum and Outlier (MR‐PRESSO) approach was employed to identify significant SNPs explaining possible pleiotropy [23], and outlier SNPs were subsequently removed. *F* statistics = (Beta/Se)^2^ results, indicating the strength of MR, were assessed, and SNPs with an *F* statistics value below 10, suggesting insufficient strength [24, 25], were pruned. In this formula, beta is the correlation coefficient between SNPs and traits, SE represents standard error. All *F* statistics values in this MR study exceeded 10.

### Main statistical analyses

The primary method employed for exploring causal associations in this MR study was the random effects inverse variance weighting (IVW) approach. This analysis was chosen for its ability to provide a robust causal estimate in the absence of directional pleiotropy, ensuring no violation of the independence assumption. To adjust the results for multiple comparisons, the false discovery rate was introduced, applying the Benjamini and Hochberg procedure. All analyses were conducted using R software (version 4.2.2) and various packages including TwoSampleMR (version 0.5.6), mr.raps (version 0.4.1), MRPRESSO (version 1.0), MendelianRandomization (version 0.9.0) and ggplot2 (version 3.4.0).

### Sensitivity analyses

Multiple sensitivity analyses were performed using various methods, including MR‐Egger, weighted median, maximum likelihood, MR Robust Adjusted Profile Score (MR‐RAPS) and MR‐PRESSO. MR‐Egger, operating on the assumption of Instrument Strength Independent of Direct Effect (InSIDE), assessed the existence of pleiotropy through the intercept term. A close‐to‐zero intercept indicated the absence of horizontal pleiotropy, aligning results with IVW [26]. Weighted median‐based MR analysis corrected causal effect estimations, assuming that at least half of the instruments were valid [27, 28]. Maximum likelihood, relying on the absence of heterogeneity and horizontal pleiotropy, provided unbiased findings under the true hypothesis. Standard errors in maximum likelihood were smaller than those in IVW [29]. MR‐PRESSO analysis removed significant outliers to mitigate horizontal pleiotropy, requiring up to 50% valid instruments and depending on the InSIDE assumption [23]. MR‐RAPS analysis, capable of maintaining statistical power in the presence of weak SNPs, verified the conclusion's robustness [30]. Cochran's *Q* statistic explored heterogeneity amongst variant‐specific estimates, and leave‐one‐out analysis confirmed the robustness of the conclusion.

## RESULTS

### Genetic instrument variables for 41 inflammatory cytokines in MR analysis

Table S2 provides a summary of SNPs utilized as IVs for each inflammatory cytokine. The MR results, depicting the causal effects of inflammatory cytokines on IA, uIA and SAH, are presented in Table S3. Based on the IVW method, the results indicate that 10, six and eight inflammatory cytokines exhibit a causal association with IA, uIA and SAH, respectively (Table S3).

### Causal effects of the genetically predicted 41 inflammatory cytokines on IA, uIA and SAH in MR analysis

As shown in Figures S1 and S2, genetically predicted vascular endothelial growth factor (VEGF) (*p* = 0.0018), MCP_3 (*p* = 4.73E‐21), and IL_12_P70 (*p* = 0.0012) were causally related to the increased risk of IA. The odds ratios (ORs) for these links were 1.08 (95% confidence interval [CI] 1.03–1.14) for VEGF, 1.15 (95% CI 1.12–1.19) for MCP_3 and 1.11 (95% CI 1.04–1.19) for IL_12_P70. In contrast, the inverse causal association between RANTES (*p* = 0.0119), MIP_1A (*p* = 0.0049), FGF_BASIC (*p* = 1.10E‐57), TNF_B (*p* = 5.52E‐05), IP_10 (*p* = 0.0047), IL_18 (*p* = 0.0113) and IL_17 (*p* = 0.0028). The ORs for these inverse associations were 0.86 (95% CI 0.76–0.97) for RANTES, 0.84 (95% CI 0.75–0.95) for MIP_1A, 0.67 (95% CI 0.63–0.70) for FGF_BASIC, 0.90 (95% CI 0.85–0.95) for TNF_B, 0.88 (95% CI 0.80–0.96) for IP_10, 0.94 (95% CI 0.90–0.99) for IL_18 and 0.82 (95% CI 0.72–0.93) for IL_17.

Amongst these inflammatory cytokines, uIA risk was intensified by interferon γ (IFN_G) (OR 1.14, 95% CI 1.09–1.19, *p* = 1.44E‐08), IL_9 (OR 1.24, 95% CI 1.14–1.35, *p* = 2.54E‐07) and IL_7 (OR 1.09, 95% CI 1.03–1.15, *p* = 0.0026), whilst MIP_1A (OR 0.77, 95% CI 0.64–0.92, *p* = 0.0048), FGF_BASIC (OR 0.59, 95% CI 0.40–0.86, *p* = 0.0063) and MCP_3 (OR 0.87, 95% CI 0.85–0.88, *p* = 1.32E‐43) decreased the risk of uIA (Figures S3 and S4).

Figures S5 and S6 display the positive causal links between inflammatory cytokines and SAH. Genetically predicted VEGF (OR 1.12, 95% CI 1.04–1.20, *p* = 0.0022), MCP_3 (OR 1.28, 95% CI 1.26–1.29, *p* = 1.01E‐06) and IL_12_P70 (OR 1.15, 95% CI 1.06–1.26, *p* = 0.0015) were causally related to the increased risk of SAH, whilst the risk of SAH was decreased by RANTES (OR 0.81, 95% CI 0.70–0.92, *p* = 0.0017), FGF_BASIC (OR 0.62, 95% CI 0.45–0.86, *p* = 0.0039), IP_10 (OR 0.91, 95% CI 0.88–0.95, *p* = 4.78E‐06), MIG (also known CXCL9, OR 0.79, 95% CI 0.70–0.88, *p* = 5.18E‐05) and IL_18 (OR 0.90, 95% CI 0.85–0.95, *p* = 0.0002).

In sensitivity analyses, leave‐one‐out analyses showed no significant SNPs for IA, uIA and SAH (Figures S7–S9). The results of MR‐Egger and MR‐PRESSO analyses demonstrated no signs of pleiotropy (Table S4). Moreover, the results of Cochran's *Q* test demonstrated no signs of heterogeneity (Table S5).

FGF_BASIC and MCP_3 were common inflammatory cytokines amongst IA, uIA and SAH (Figure S10). RANTES, VEGF, IP_10, IL_18 and IL_12_P70 were common inflammatory cytokines between IA and SAH, and MIP_1A was a common inflammatory cytokine between IA and uIA.

### Causal effects of the genetically predicted 41 inflammatory cytokines on IA, uIA and SAH in FinnGen datasets in replicate MR analysis

Mendelian randomization results of the causal effects of inflammatory cytokines on IA, uIA and SAH in FinnGen datasets are shown in Table S6.

Figures S11 and S12 display the positive MR results of the association between inflammatory cytokines and IA. The risk of IA was decreased by IL_13 (OR 0.86, 95% CI 0.80–0.93, *p* = 0.0001), IL_1B (OR 0.81, 95% CI 0.76–0.86, *p* = 1.84E‐11), MIP_1A (OR 0.61, 95% CI 0.45–0.82, *p* = 0.0011) and IP_10 (OR 0.63, 95% CI 0.50–0.80, *p* = 0.0001).

For uIA in FinnGen datasets (Figures S13 and S14), genetically predicted PDGF_BB (*p* = 0.0004) was causally related to the increased risk of uIA. The OR for this link was 1.26 (95% CI 1.11–1.44) for Platelet‐derived growth factor‐BB (PDGF_BB). In contrast, there was an inverse causal association between MIP_1A (*p* = 3.34E‐12), IP_10 (*p* = 0.0002), granulocyte colony stimulating factor (G_CSF) (*p* = 0.0020) and β nerve growth factor (B_NGF) (*p* = 2.68E‐07). The ORs for these inverse associations were 0.58 (95% CI 0.50–0.68) for MIP_1A, 0.78 (95% CI 0.69–0.89) for IP_10, 0.84 (95% CI 0.75–0.94) for G_CSF and 0.82 (95% CI 0.76–0.88) for B_NGF.

Amongst these inflammatory cytokines, SAH risk was decreased by MIP_1A (OR 0.70, 95% CI 0.57–0.87, *p* = 0.0013) and IP_10 (OR 0.81, 95% CI 0.73–0.90, *p* = 8.29E‐05) (Figures S15 and S16).

Leave‐one‐out analysis of sensitivity analyses showed no significant SNPs for IA, uIA and SAH (Figures S17–S19). The results of MR‐Egger and MR‐PRESSO analyses demonstrated no signs of pleiotropy (Table S7). Moreover, the results of Cochran's *Q* test demonstrated no signs of heterogeneity (Table S8).

MIP_1A and IP_10 were common inflammatory cytokines amongst IA, uIA and SAH (Figure S20).

### Common inflammatory cytokines for IA, uIA and SAH between MR and replicate MR analysis

As shown in Figures S21–S23, MIP_1A, MIP_1A and IP_10 were the common inflammatory cytokines for IA, uIA and SAH in MR and replicate MR analysis, respectively.

### Genetic instrument variables for 41 inflammatory cytokines in the reverse MR analysis

The IVs for IA, uIA and SAH are summarized in Table S9. Twelve, one and five IVs were selected for IA, uIA and SAH, respectively. As shown in Table S10, *F* statistics of these IVs for IA, uIA and SAH were between 50.50 and 51.99, 32.64 and 53.18, respectively.

### Causal effects of the genetically predicted IA, uIA and SAH on 41 inflammatory cytokines in the reverse MR analysis

Mendelian randomization results of causal effects of IA, uIA and SAH on 41 inflammatory cytokines are shown in Table S11. The positive MR results for IA are displayed in Figures S24 and S25. All inflammatory cytokines were increased after IA. Specifically, IA increased the level of IL_6 (OR 1.12, 95% CI 1.04–1.20, *p* = 0.0013), IL_4 (OR 1.13, 95% CI 1.06–1.20, *p* = 0.0002), MIF (migration inhibitory factor, OR 1.10, 95% CI 1.03–1.19, *p* = 0.0045), VEGF (OR 1.08, 95% CI 1.00–1.16, *p* = 0.0289), IL_8 (OR 1.10, 95% CI 1.01–1.22, *p* = 0.0485), IFN_G (OR 1.13, 95% CI 1.06–1.21, *p* = 0.0001), FGF_BASIC (OR 1.06, 95% CI 1.00–1.13, *p* = 0.0307), IL_9 (OR 1.10, 95% CI 1.02–1.20, *p* = 0.0133), IL_1RA (OR 1.18, 95% CI 1.05–1.32, *p* = 0.0032), G_CSF (OR 1.06, 95% CI 1.01–1.12, *p* = 0.022), IL_10 (OR 1.11, 95% CI 1.04–1.17, *p* = 0.0002), IL_2 (OR 1.12, 95% CI 1.00–1.25, *p* = 0.0437) and IL_12_P70 (OR 1.14, 95% CI 1.05–1.23, *p* = 1.67E‐03).

For uIA, a total of 13 inflammatory cytokines were increased after uIA (Figure S26). uIA increased the level of IL_6 (OR 1.36, 95% CI 1.16–1.60, *p* = 0.0001), IL_13 (OR 1.37, 95% CI 1.08–1.75, *p* = 0.0092), IL_4 (OR 1.22, 95% CI 1.04–1.44, *p* = 0.0134), VEGF (OR 1.27, 95% CI 1.07–1.51, *p* = 0.0058), MIP_1A (OR 1.29, 95% CI 1.01–1.65, *p* = 0.0350), IFN_G (OR 1.27, 95% CI 1.07–1.49, *p* = 0.0045), FGF_BASIC (OR 1.25, 95% CI 1.06–1.48, *p* = 0.007), IL_9 (OR 1.29, 95% CI 1.01–1.63, *p* = 0.0356), IL_1RA (OR 1.50, 95% CI 1.18–1.91, *p* = 0.0007), IL_10 (OR 1.26, 95% CI 1.07–1.49, *p* = 0.0050), TNF_A (OR 1.29, 95% CI 1.01–1.65, *p* = 0.0377), IL_12_P70 (OR 1.28, 95% CI 1.09–1.50, *p* = 0.0025) and IL_17 (OR 1.22, 95% CI 1.03–1.43, *p* = 0.0183).

As for SAH, six inflammatory cytokines were increased after SAH (Figures S27 and S28). The level of IL_4, VEGF, IFN_G, IL_10, IL_12_P70 and IL_17 increased after SAH. Specifically, SAH increased the level of IL_4 (OR 1.13, 95% CI 1.06–1.20, *p* = 7.10E‐05), VEGF (OR 1.10, 95% CI 1.00–1.23, *p* = 0.0488), IFN_G (OR 1.12, 95% CI 1.03–1.22, *p* = 0.0055), IL_10 (OR 1.11, 95% CI 1.02–1.20, *p* = 0.0083), IL_12_P70 (OR 1.15, 95% CI 1.03–1.29, *p* = 0.0082) and IL_17 (OR 1.08, 95% CI 1.00–1.17, *p* = 0.0464).

In sensitivity analysis, no signs of pleiotropy and heterogeneity were observed (Table S12). Leave‐one‐out analysis of sensitivity analyses showed no significant SNPs for IA, uIA and SAH (Figures S29 and S30).

IL_4, VEGF, IFN_G, IL_10 and IL_12_P70 were common inflammatory cytokines between IA, uIA and SAH (Figure S31). IL_6, FGF_BASIC, IL_9 and IL_1RA were common inflammatory cytokines between IA and uIA. IL_17 was a common inflammatory cytokine between uIA and SAH.

### Causal effects of the genetically predicted IA, uIA and SAH on 41 inflammatory cytokines in FinnGen datasets in replicate reverse MR analysis

As shown in Table S13, *F* statistics of these IVs for IA, uIA and SAH were between 35.49, 30.92 and 37.56, respectively. MR results of causal effects of IA, uIA and on SAH 41 inflammatory cytokines in FinnGen datasets are shown in Table S14.

The positive MR results for IA in FinnGen datasets are displayed in Figures S32 and S33. The level of IL_1B was increased after IA (OR 1.03, 95% CI 1.01–1.06, *p* = 0.0129). For uIA, all the inflammatory cytokines were increased (Figures S34 and S35). Specifically, uIA increased the level of VEGF (OR 1.18, 95% CI 1.04–1.34, *p* = 0.0080), MIP_1A (OR 1.24, 95% CI 1.23–1.25, *p* = 1.00E‐08), stem cell growth factor β (SCGF_B) (OR 1.06, 95% CI 1.02–1.10, *p* = 0.0019), IL_5 (OR 1.08, 95% CI 1.01–1.16, *p* = 0.0196), stem cell factor (SCF) (OR 1.03, 95% CI 1.02–1.04, *p* = 1.56E‐29), IL_9 (OR 1.21, 95% CI 1.18–1.25, *p* = 6.21E‐48), MCP_3 (OR 1.34, 95% CI 1.13–1.58, *p* = 0.0005), PDGF_BB (OR 1.03, 95% CI 1.01–1.05, *p* = 0.0003), G_CSF (OR 1.08, 95% CI 1.01–1.14, *p* = 0.0101), IL_10 (OR 1.13, 95% CI 1.02–1.26, *p* = 0.0188), TNF_A (OR 1.25, 95% CI 1.20–1.30, *p* = 1.75E‐29), IL_7 (OR 1.03, 95% CI 1.01–1.06, *p* = 0.0016), IL_2 (OR 1.12, 95% CI 1.01–1.24, *p* = 0.0259) and TRAIL (tumor necrosis factor‐related apoptosis‐inducing ligand, OR 1.03, 95% CI 1.01–1.07, *p* = 0.0423). As to SAH, the level of CTACK (cutaneous T cell‐attracting chemokine, OR 1.14, 95% CI 1.00–1.30, *p* = 0.0401) was increased, whereas TNF_B (OR 0.88, 95% CI 0.85–0.91, *p* = 1.24E‐15), HGF (OR 0.91, 95% CI 0.84–0.98, *p* = 0.0126), EOTAXIN (also known as CCL11 OR 0.92, 95% CI 0.85–0.99, *p* = 0.0428), SCF (OR 0.89, 95% CI 0.81–0.98, *p* = 0.0244), TNF_A (OR 0.91, 95% CI 0.86–0.96, *p* = 0.0025), IL_7 (OR 0.90, 95% CI 0.85–0.95, *p* = 0.0001), TRAIL (OR 0.94, 95% CI 0.89–0.99, *p* = 0.0355), IL_17 (OR 0.93, 95% CI 0.87–0.99, *p* = 0.0399) and B_NGF (OR 0.88, 95% CI 0.81–0.95, *p* = 0.0011) were decreased (Figures S36 and S37).

In sensitivity analysis, no evidence of pleiotropy and heterogeneity was observed (Table S15). Leave‐one‐out analysis of sensitivity analyses showed no significant SNPs for IA, uIA and SAH (Figures S38–S40).

SCF, TNF_A, IL_7 and TRAIL were common inflammatory cytokines between uIA and SAH (Figure S41).

### Common inflammatory cytokines for IA, uIA and SAH between reverse MR and replicate reverse MR analysis

As shown in Figures S42–S44, the common cytokines altered by uIA were VEGF, MIP_1A, IL_9 and IL_10 in reverse MR and replicate reverse MR analysis. The common cytokine altered by SAH was IL_17.

### Combined results from the meta‐analysis

**FIGURE 2:**
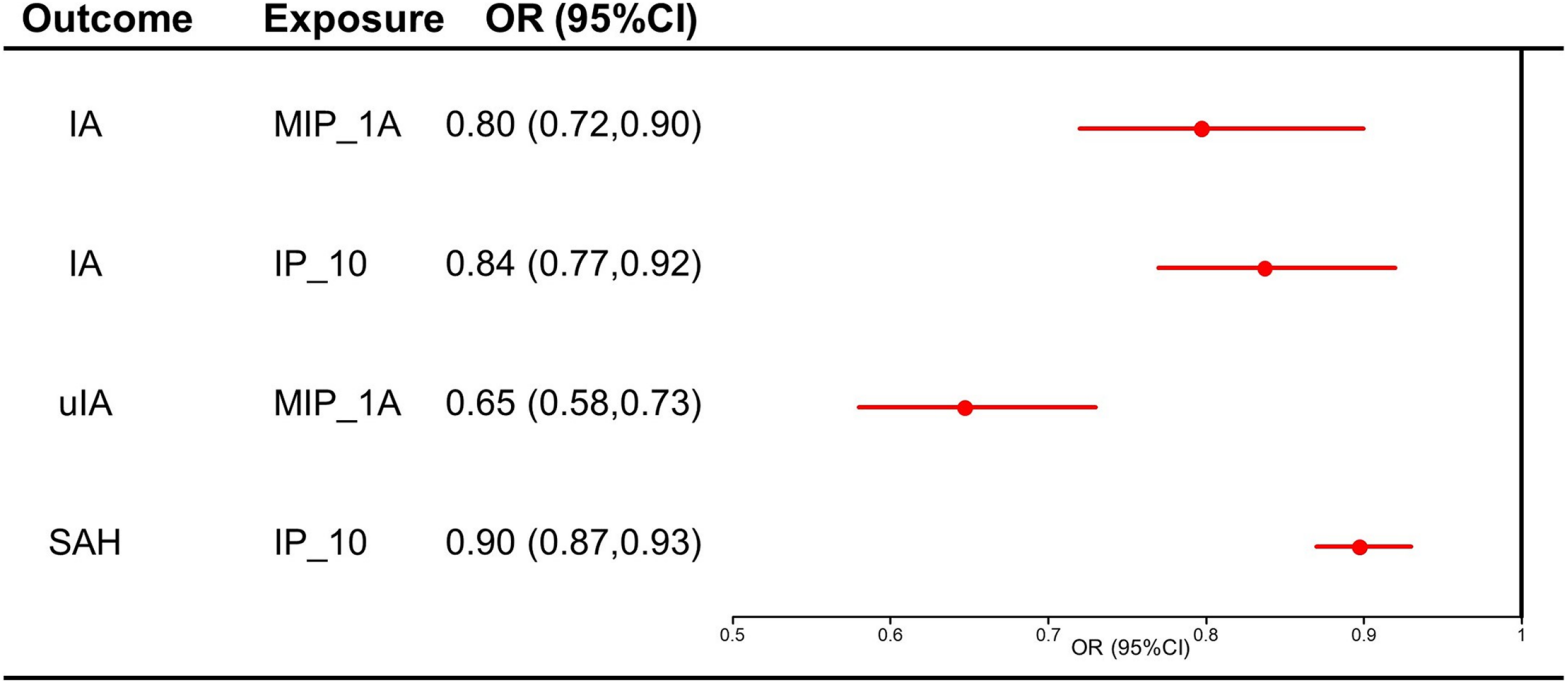
Forest plot of results from a meta‐analysis of MR results and replicate MR results. IA, intracranial aneurysm; MR, Mendelian randomization; SAH, subarachnoid hemorrhage; uIA, unruptured intracranial aneurysm.

To assess the robustness of our findings, a meta‐analysis was conducted, consolidating the outcomes derived from both MR and replicate MR analyses. The summarized results of the meta‐analyses for MR and replicate MR analyses in Figure 2 reveal that MIP_1A and IP_10 could be causally associated with the decreased risk of IA (MIP_1A, OR_meta_ = 0.80, 95% CI 0.72–0.90; IP_10, OR_meta_ = 0.84, 95% CI 0.77–0.92) and MIP_1A was associated with the decreased risk of uIA (OR_meta_ = 0.65, 95% CI 0.58–0.73). In addition, IP_10 was related to the decreased risk of SAH (OR_meta_ = 0.90, 95% CI 0.87–0.93).

**FIGURE 3:**
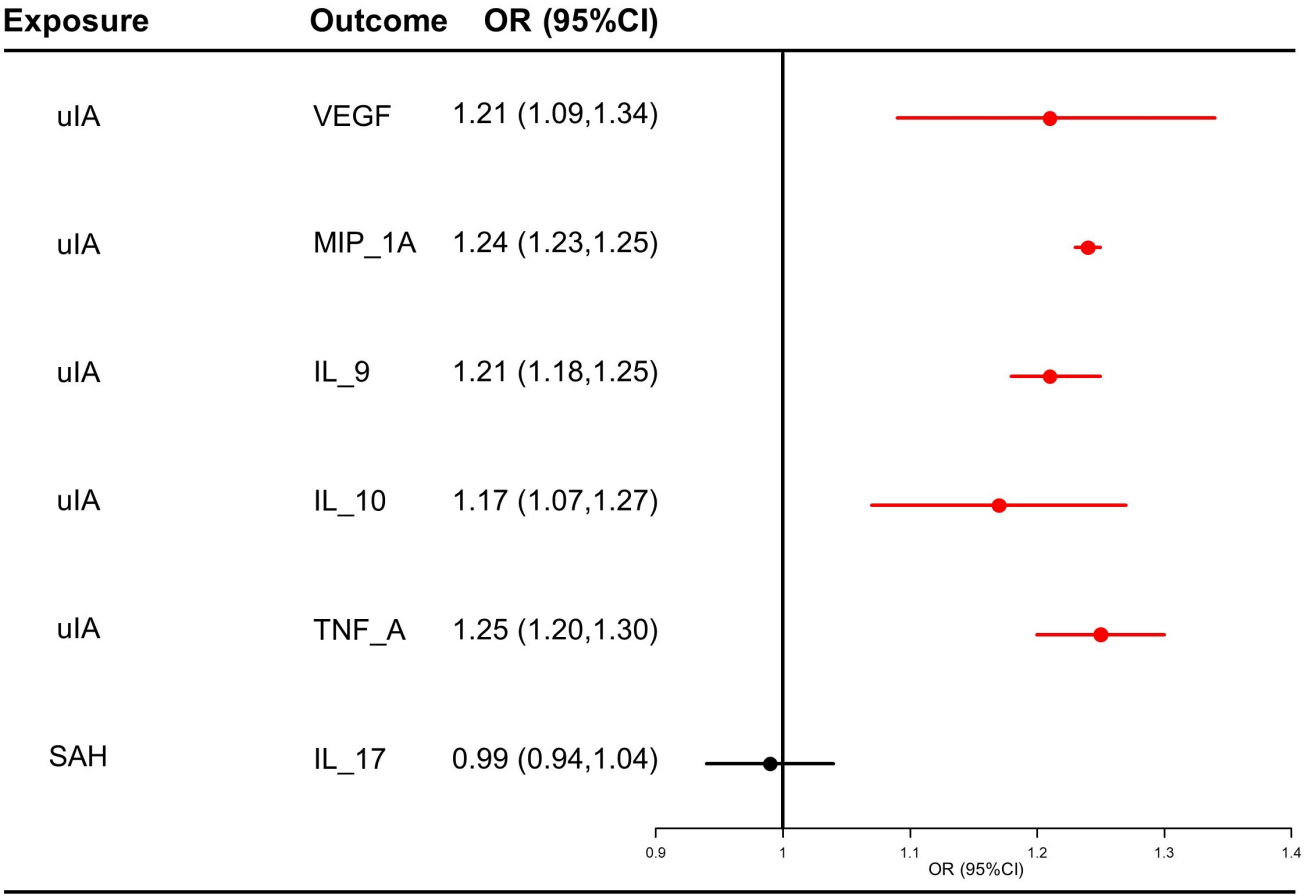
Forest plot of results from a meta‐analysis of reverse MR results and replicate reverse MR results. MR, Mendelian randomization; SAH, subarachnoid hemorrhage; uIA, unruptured intracranial aneurysm.

The consolidated outcomes derived from both reverse MR and replicate reverse MR analyses in Figure 3 demonstrate that the levels of VEGF (OR_meta_ = 1.21, 95% CI 1.09–1.34), MIP_1A (OR_meta_ = 1.24, 95% CI 1.23–1.25), IL_9 (OR_meta_ = 1.21, 95% CI 1.18–1.25), IL_10 (OR_meta_ = 1.17, 95% CI 1.07–1.27) and TNF_A (OR_meta_ = 1.25, 95% CI 1.20–1.30) were increased with uIA.

## DISCUSSION

In this MR study, the causal effects of inflammatory cytokines on the risk of IA, uIA and SAH using multiple datasets were initially identified. In the ISGC datasets, our results revealed causal associations of 10, six and eight inflammatory cytokines with IA, uIA and SAH, respectively. FGF_BASIC and MCP_3 were common inflammatory cytokines amongst IA, uIA and SAH. RANTES, VEGF, IP_10, IL_18 and IL_12_P70 were common inflammatory cytokines between IA and SAH, and MIP_1A was a common inflammatory cytokine between IA and uIA. In replicate MR analyses for FinnGen datasets, four, five and two inflammatory cytokines were identified as causally associated with IA, uIA and SAH, respectively. MIP_1A and IP_10 were common inflammatory cytokines amongst IA, uIA and SAH. These results from MR and replicate MR analyses revealed that MIP_1A, MIP_1A and IP_10 were the common inflammatory cytokines for IA, uIA and SAH respectively in MR and replicate MR analyses. In reverse MR analysis for ISGC datasets, IA, uIA and SAH were associated with increased levels of 13, 13 and six inflammatory factors, respectively. IL_4, VEGF, IFN_G, IL_10 and IL_12_P70 were common inflammatory cytokines amongst IA, uIA and SAH. IL_6, FGF_BASIC, IL_9 and IL_1RA were common inflammatory cytokines amongst IA and uIA. IL_17 was a common inflammatory cytokine amongst uIA and SAH. In the replicate reverse MR analysis for FinnGen datasets, IA, uIA and SAH altered the levels of one, 14 and 10 inflammatory factors, respectively. SCF, TNF_A, IL_7 and TRAIL were common inflammatory cytokines between uIA and SAH. The common cytokines altered by uIA from ISGC and FinnGen datasets were VEGF, MIP_1A, IL_9 and IL_10 in reverse MR and replicate reverse MR analyses. IL_17 was the common cytokine altered by SAH. In the meta‐analysis of MR results, MIP_1A and IP_10 were found to be causally associated with decreased IA risk, and MIP_1A was associated with decreased uIA risk. Additionally, IP_10 was related to decreased SAH risk. The meta‐analysis of reverse MR results demonstrated that the levels of VEGF, MIP_1A, IL_9, IL_10 and TNF_A increased with the development of uIA. Overall, our results offer novel insights into specific inflammatory cytokines' effects on IA, uIA and SAH development, influencing the cytokine levels reciprocally. Targeting these specific cytokines may be beneficial in preventing and treating IA, uIA and SAH.

Currently, research on the role of MIP_1A (also known as CCL3) in the development of cerebral aneurysms is limited. A clinical study involving 55 IA specimens and control arteries found an increased level of MIP_1A in IA and suggested that MIP_1A plays a crucial role in macrophage recruitment in the pathogenesis of IA progression [31]. However, there are notable limitations that significantly impact the interpretation of this conclusion. First, although the IA specimens are collected from patients, the extra‐cranial arteries are considered as controls. Attention should be paid to considerable differences in the gene expression profile between intra‐cranial arteries and extra‐cranial arteries. Additionally, verifying their results using a human carotid artery with a synthetic phenotype may enhance the expression of some pro‐inflammatory genes. Therefore, their results should be interpreted with caution. A bioinformatic analysis also observed the increased level of MIP_1A in patients with IA [32]. These studies did not elucidate the role of MIP_1A in the formation of IA. In contrast, studies on the role of MIP_1A in the formation of IA in extra‐cranial arteries were illuminated. In a preclinical abdominal aortic aneurysm model induced by CaCl_2_, an increased expression of CCL3 was observed. However, Ccl3^−/−^ and Ccr5^−/−^ (Chemokine receptors) mice exhibit exaggerated abdominal aortic aneurysm (AAA) with augmented macrophage infiltration and (matrix metalloproteinase) MMP‐9 expression, whilst CCL3 treatment reverses this phenomenon in both wild‐type and Ccl3^−/−^ mice by the CCL3–CCR5 axis [33]. This study suggests that the CCL3–CCR5 axis can prevent CaCl_2_‐induced aortic inflammation and subsequent aneurysm formation. The results of murine carotid aneurysms also demonstrated that MCP‐1 exerts inflammatory intra‐aneurysmal tissue healing through an MIP‐1A‐ and MIP‐2‐dependent pathway [34]. The study did not explain the role of MIP‐1A in IA formation. In this MR study, it was found that MIP_1A can decrease the formation of IA and uIA.

Currently, research on the role of IP_10 (CXCL10) in mouse models for abdominal aortic aneurysm primarily focuses on extra‐cranial arteries, and these studies yield conflicting results. A clinical study revealed a correlation between CXCL10 and outward arterial remodeling, intimal expansion, matrix degradation and IA rupture [35]. Notably, a significant increase in IP_10 is detected in popliteal artery aneurysms and thoracic aortic aneurysms [36, 37], which have also been detected in IA [38] and SAH [39]. Despite these findings, the specific role of IP_10 remains unclear. Intriguingly, in ApoE^−/−^CXCL10^−/−^ mice, IA induced by angiotensin II infusion exhibits a higher frequency of dilatation and rupture, suggesting a protective role for CXCL10 in IA formation [40]. In line with this, this MR study similarly demonstrates that an increased level of IP_10 acts as a protector against IA and SAH.

In the reverse MR analysis, elevated levels of VEGF, MIP_1A, IL_9, IL_10 and TNF_A in plasma were also observed associated with uIA. Previous research has shown increased expression of VEGF [41], CCL3 [31, 32], IL_10 [4] and TNF_A [4] in human IA. However, the alteration in IL_9 levels in IA is not well documented. Interestingly, an increased level of IL_9 has been reported in aneurysmal abdominal aorta [42] and aortic dissection [43].

This MR study was designed to comprehensively investigate the causal association between a diverse array of inflammatory cytokines and the development of IA, uIA and SAH. The aim was to establish a bidirectional relationship, identifying factors that play a protective role in the pathogenesis of these conditions, thereby offering potential targets for prevention. In particular, our study boasts the largest sample size across multiple independent datasets, encompassing discovery, validation and meta‐analysis stages. Rigorous criteria, including false discovery rate correction and adherence to meta‐analysis standards, ensure the robustness of our results. This expansive approach sets our study apart. Furthermore, the bidirectional MR design addresses the limitations of reverse causation and residual confounding often encountered in observational studies. Leveraging serum, a widely accessible diagnostic tool, facilitates sample collection from both patients and healthy controls, aiding in the identification of high‐risk individuals, especially those with predisposing factors like hypertension and obesity. Considering the common use of aspirin as an anti‐inflammatory drug, our findings may have implications for individuals at high risk of IA, uIA and SAH [44, 45]. Finally, previous studies have focused on the pathogenic mechanism involved in IA, uIA and SAH, whilst our study provides crucial clues for the research to explore the role of inflammatory cytokines in the natural history of the development of IA, uIA and SAH.

Whilst our study provides valuable insights, it is essential to acknowledge several limitations. First, the threshold filtering of the IVs is set at a significance level of *p* < 5 × 10^−6^, which may be considered relatively lenient. Additionally, some analyses face constraints due to the relatively limited number of IVs, impacting the statistical power to reject null hypotheses in certain associations. Furthermore, the generalizability of our findings is confined by the use of GWAS summary‐level data exclusively from European participants. Another constraint arises from the unavailability of individual‐level association data, as genetic information on the subjects is not accessible. Although our study identified changes in the levels of several cytokines, the specific roles of these factors in disease progression remain unknown, primarily due to the current lack of datasets and methodologies to elucidate them. Moreover, it is important to note that cytokines exhibit dynamic expression patterns in the body, with specific inflammatory cytokines induced to high levels but only for brief periods during inflammation. Consequently, the static nature of MR studies limits their ability to capture the dynamic changes in cytokines. Finally, it is crucial to recognize that the levels of circulating cytokines analyzed in MR studies may not fully represent the complex cytokine microenvironment during the course of the disease.

## CONCLUSION

In conclusion, novel and comprehensive evidence about the possible bidirectional causal association between inflammatory cytokines and IA, uIA and SAH is provided, indicating that these inflammatory factors might represent the etiology and new biomarkers for the diagnosis and treatment of these diseases in clinical practice. MIP_1A and IP_10 could be causally associated with the decreased risk of IA, and MIP_1A was associated with the decreased risk of uIA. In addition, IP_10 was related to the decreased risk of SAH. The level of VEGF, MIP_1A, IL_9, IL_10 and TNF_A was increased with the development of uIA. Future research is needed to verify the causal association between inflammatory cytokines and IA, uIA and SAH and clarify the specific mechanism.

## AUTHOR CONTRIBUTIONS

**Qiang He:** Conceptualization; methodology; software; validation; visualization; writing – original draft. **Wenjing Wang:** Conceptualization; validation; formal analysis. **Yang Xiong:** Validation; visualization; resources. **Chuanyuan Tao:** Investigation; funding acquisition; formal analysis. **Lu Ma:** Supervision; investigation. **Chao You:** Writing – review and editing; software; data curation; supervision.

## FUNDING INFORMATION

The project is supported by funding from National Natural Science Foundation of China grants 82301497.

## CONFLICT OF INTEREST STATEMENT

All authors declare that the research was conducted in the absence of any commercial or financial relationships that could be construed as a potential conflict of interest.

## Supporting information


Appendix S1.

## Data Availability

All the data in our MR study are publicly accessible (https://data.bris.ac.uk/data/dataset/3074krb6t2frj29yh2b03x3wxj and www.cerebrovascularportal.org).
