# Supplementary material for: Causal association between circulating inflammatory cytokines and intracranial aneurysm and subarachnoid hemorrhage
Source: Eur J Neurol. 2024 May 6;31(8):e16326. doi: 10.1111/ene.16326 (PMC11235611; doi:10.1111/ene.16326)
Supplement: Supplementary file 1 — Appendix S1. [file ENE-31-e16326-s001.docx]

**Methods**

**Results**

**Table S1** The summary-level GWAS data sources in MR analysis

**Table S2** The IVs used in MR analysis

**Table S3** The MR results of causal effects of inflammatory cytokines in the IA, uIA, and SAH in MR analysis

**Table S4** The MR-Egger results of causal effects of inflammatory cytokines on the IA, uIA, SAH in MR analysis

**Table S5** The Cochran’s test results of causal effects of inflammatory cytokines on the IA, uIA, SAH in MR analysis

**Table S6** The MR results of causal effects of inflammatory cytokines on the IA, uIA and SAH in FinnGen in replicate MR analysis

**Table S7** The MR-Egger results of causal effects of inflammatory cytokines on the IA, uIA and SAH in FinnGen datasets in replicate MR analysis

**Table S8** The Cochran’s test results of causal effects of inflammatory cytokines on the uIA and SAH in FinnGen datasets in replicate MR analysis

**Table S9** IVs used in reverse MR analysis

**Table S10** The MR-Egger and Cochran’s Q test of IA, uIA, and SAH in reverse MR analyses

**Table S11** The MR results of IA, uIA, and SAH on inflammation cytokines in reverse MR analyses

**Table S12** The MR-Egger and Cochran’s test results of IA, uIA, and SAH on forty-one inflammation cytokines in the reverse MR analysis

**Table S13** The MR-Egger and Cochran’s Q test of IA, uIA, and SAH in FinnGen datasets in replicate reverse MR analyses

**Table S14** The MR results of IA, uIA, and SAH on inflammation cytokines in FinnGen datasets in replicate reverse MR analyses

**Table S15** The MR-Egger and Cochran’s test results of IA, uIA, and SAH on forty-one inflammation cytokines in FinnGen datasets in replicate reverse MR analysis

**Figure S1** Causal association between inflammatory cytokines and IA in MR analysis

**Figure S2** The scatter plots of the association between genetically predicted inflammation cytokines on IA in MR analysis.

**Figure S3** Causal association between inflammatory cytokines and uIA in MR analysis.

**Figure S4** The scatter plots of the association between genetically predicted inflammation cytokines on uIA in MR analysis.

**Figure S5** Causal association between inflammatory cytokines and SAH in MR analysis.

**Figure S6** The scatter plots of the association between genetically predicted inflammation cytokines on SAH in MR analysis.

**Figure S7** The leave-one-out analysis for causal association between inflammation cytokines and IA in MR analysis.

**Figure S8** The leave-one-out analysis for causal association between inflammation cytokines and uIA in MR analysis.

**Figure S9** The leave-one-out analysis for causal association between inflammation cytokines and SAH in MR analysis.

**Figure S10** The common inflammation cytokines among IA, uIA, and SAH in MR analysis. IA, intracranial aneurysm; uIA, unruptured intracranial aneurysm, SAH, subarachnoid hemorrhage; MR, mendelian randomization.

**Figure S11** Causal association between inflammatory cytokines and IA in replicate MR analysis. IA, intracranial aneurysm; MR, mendelian randomization.

**Figure S12** The scatter plots of the association between genetically predicted inflammation cytokines on IA in FinnGen datasets in replicate MR analysis. IA, intracranial aneurysm; MR, mendelian randomization.

**Figure S13** Causal association between inflammatory cytokines and uIA in replicate MR analysis. uIA, unruptured intracranial aneurysm; MR, mendelian randomization.

**Figure S14** The scatter plots of the association between genetically predicted inflammation cytokines on uIA in FinnGen datasets in replicate MR analysis.

**Figure S15** Causal association between inflammatory cytokines and SAH in replicate MR analysis.

**Figure S16** The scatter plots of the association between genetically predicted inflammation cytokines on SAH in FinnGen datasets in replicate MR analysis.

**Figure S17** The leave-one-out analysis for causal association between inflammation cytokines and IA in FinnGen datasets in replicate MR analysis.

**Figure S18** The leave-one-out analysis for causal association between inflammation cytokines and uIA in FinnGen datasets in replicate MR analysis.

**Figure S19** The leave-one-out analysis for causal association between inflammation cytokines and SAH in FinnGen datasets in replicate MR analysis.

**Figure S20** The common inflammation cytokines among IA, uIA, and SAH in FinnGen datasets in replicate MR analysis.

**Figure S21** The common inflammation cytokines for IA between MR and replicate MR analysis.

**Figure S22** The common inflammation cytokines for uIA between MR and replicate MR analysis. uIA, unruptured intracranial aneurysm; MR, mendelian randomization.

**Figure S23** The common inflammation cytokines for SAH between MR and replicate MR analysis.

**Figure S24** Causal association between inflammatory cytokines and IA in reverse MR analysis.

**Figure S25** The scatter plots of the association between genetically predicted IA on inflammation cytokines in reverse MR analysis.

**Figure S26** Causal association between inflammatory cytokines and uIA in reverse MR analysis.

**Figure S27** Causal association between inflammatory cytokines and SAH in reverse MR analysis.

**Figure S28** The scatter plots of the association between genetically predicted SAH on inflammation cytokines in reverse MR analysis.

**Figure S29** The leave-one-out analysis for causal association between IA and inflammation cytokines in reverse MR analysis.

**Figure S30** The leave-one-out analysis for causal association between SAH and inflammation cytokines in reverse MR analysis.

**Figure S31** The common inflammation cytokines among IA, uIA, and SAH in reverse MR analysis.

**Figure S32** Causal association between inflammatory cytokines and IA in FinnGen datasets in replicate reverse MR analysis.

**Figure S33** The scatter plots of the association between genetically predicted IA on inflammation cytokines in FinnGen datasets in replicate reverse MR analysis.

**Figure S34** Causal association between inflammatory cytokines and uIA in FinnGen datasets in replicate reverse MR analysis.

**Figure S35** The scatter plots of the association between genetically predicted uIA on inflammation cytokines in FinnGen datasets in replicate reverse MR analysis.

**Figure S36** Causal association between inflammatory cytokines and SAH in FinnGen datasets in replicate reverse MR analysis.

**Figure S37** The scatter plots of the association between genetically predicted SAH on inflammation cytokines in FinnGen datasets in replicate reverse MR analysis.

**Figure S38** The leave-one-out analysis for causal association between IA and inflammation cytokines in FinnGen datasets in replicate reverse MR analysis.

**Figure S39** The leave-one-out analysis for causal association between uIA and inflammation cytokines in FinnGen datasets in replicate reverse MR analysis.

**Figure S40** The leave-one-out analysis for causal association between SAH and inflammation cytokines in FinnGen datasets in replicate reverse MR analysis.

**Figure S41** The common inflammation cytokines among IA, uIA, and SAH in FinnGen datasets in replicate reverse MR analysis.

**Figure S42** The common inflammation cytokines for IA between reverse MR and replicate reverse MR analysis.

**Figure S43** The common inflammation cytokines for uIA between reverse MR and replicate reverse MR analysis.

**Figure S44** The common inflammation cytokines for SAH between reverse MR and replicate reverse MR analysis

**Methods**

**Ethical approval and study design**

The summary-level statistics for InF associated with IA, uIA, and SAH were obtained from publicly available, de-identified datasets and previously approved studies. Relevant ethics committee approvals and the informed consent of participants have been obtained. Ethical approval is exempted for this MR study. The study adheres to the Strengthening the Reporting of Observational Studies in Epidemiology using MR (STROBE-MR) checklist ^1,2^.

**Sensitivity analyses**

Multiple sensitivity analyses were performed using various methods, including MR-Egger, Weighted Median, Maximum Likelihood, MR Robust Adjusted Profile Score (MR-RAPS), and MR-PRESSO. MR-Egger, operating on the assumption of Instrument Strength Independent of Direct Effect (InSIDE), assessed the existence of pleiotropy through the intercept term. A close-to-zero intercept indicated the absence of horizontal pleiotropy, aligning results with IVW ^3^. Weighted median-based MR analysis corrected causal effect estimations, assuming that at least half of the instruments were valid ^4,5^. Maximum likelihood, relying on the absence of heterogeneity and horizontal pleiotropy, provided unbiased findings under the true hypothesis. Standard errors in Maximum Likelihood were smaller than those in IVW ^6^. MR-PRESSO analysis removed significant outliers to mitigate horizontal pleiotropy, requiring up to 50% valid instruments and depending on InSIDE assumption ^7^. MR-RAPS analysis, capable of maintaining statistical power in the presence of weak SNPs, verified the conclusion's robustness ^8^. Cochran's Q statistic explored heterogeneity among variant-specific estimates, and leave-one-out analysis confirmed the robustness of the conclusion.

**RESULTS**

**Causal effects of InF on IA, uIA, and SAH in FinnGen in replicate MR analysis**

MR results of causal effects of InF on IA, uIA, and SAH in FinnGen datasets were shown in **Table S6**.

**Figure S14** and **Figure S15** displayed the positive MR results of the association between InF and IA. The risk of IA was decreased by IL-13 (OR=0.86, 95%CI=0.80-0.93, *P*=0.0001), IL-1B (OR=0.81, 95%CI=0.76-0.86, *P*=1.84E-11), MIP-1A (OR=0.61, 95%CI=0.45-0.82, *P*=0.0011), and IP-10 (OR=0.63, 95%CI=0.50-0.80, *P*=0.0001).

For uIA in FinnGen datasets (**Figure 16** and **Figure S17**), genetically predicted PDGF-BB (*P*=0.0004) was causally related to the increased risk of uIA. The OR for these links were 1.26(95%CI=1.11-1.44) for PDGF-BB. In contrast, an inverse causal association between MIP-1A (*P*=3.34E-12), IP-10 (*P*=0.0002), G-CSF (*P*=0.0020), B-NGF (*P*=2.68E-07). The OR for these inverse associations were 0.58(95%CI=0.50-0.68) for MIP-1A, 0.78(95%CI=0.69-0.89) for IP-10, 0.84(95%CI=0.75-0.94) for G-CSF, and 0.82(95%CI=0.76-0.88) for B-NGF.

Among these InF, SAH risk was decreased by MIP-1A (OR=0.70, 95%CI=0.57-0.87, *P*=0.0013) and IP-10 (OR=0.81, 95%CI=0.73-0.90, *P*=8.29E-05) (**Figure 18** and **Figure S19**).

Leave-one-out analysis of sensitivity analyses showed no significant SNPs for IA, uIA, and SAH (**Figure S20-S22**). The results of MR-Egger and MR-PRESSO analyses demonstrated no signs of pleiotropy (**Table S7**). Moreover, the results of Cochran’s *Q* test demonstrated no signs of heterogeneity (**Table S8**).

MIP-1A and IP-10 were common InF among IA, uIA, and SAH (**Figure S20**).

**Common InF between MR and replicate MR analysis**

As shown in **Figure S21-S23**, MIP-1A, MIP-1A, and IP-10 were the common InF for IA, uIA, and SAH in MR and replicate MR analysis, respectively.

**Causal effects of IA, uIA, and SAH from ISGC on InF in the reverse MR analysis**

The IVs for IA, uIA, and SAH were summarized in **Table S9**. Twelve, one, and five IVs were selected for IA, uIA, and SAH, respectively. As shown in **Table S10**, F statistics of these IVs for IA, uIA, and SAH were between 50.50 and 51.99, 32.64, and 53.18, respectively

MR results of causal effects of IA, uIA, and SAH on InF were shown in **Table S11**. The positive MR results for IA were displayed in **Figure S27** and **Figure S28**. All InF were increased after IA. Specifically, IA increased the level of IL-6 (OR=1.12, 95%CI=1.04-1.20, *P*=0.0013), IL-4 (OR=1.13, 95%CI=1.06-1.20), *P*=0.0002), MIF (OR=1.10, 95%CI=1.03-1.19), *P*=0.0045), VEGF (OR=1.08, 95%CI=1.00-1.16, *P*=0.0289), IL-8(OR=1.10, 95%CI=1.01-1.22, *P*=0.0485), IFN-G (OR=1.13, 95%CI=1.06-1.21, *P*=0.0001), FGF-BASIC (OR=1.06, 95%CI=1.00-1.13, *P*=0.0307), IL-9(OR=1.10, 95%CI=1.02-1.20, *P*=0.0133), IL-1RA (OR=1.18, 95%CI=1.05-1.32, *P*=0.0032), G-CSF (OR=1.06, 95%CI=1.01-1.12, *P*=0.022), IL-10(OR=1.11, 95%CI=1.04-1.17, *P*=0.0002), IL-2(OR=1.12, 95%CI=1.00-1.25, *P*=0.0437), and IL-12-P70(OR=1.14, 95%CI=1.05-1.23, *P*=1.67E-03).

For uIA, a total of thirteen InF were increased after uIA (**Figure S29**). uIA increased the level of IL-6 (OR=1.36, 95%CI=1.16-1.60, *P*=0.0001), IL-13 (OR=1.37, 95%CI=1.08-1.75, *P*=0.0092), IL-4 (OR=1.22, 95%CI=1.04-1.44, *P*=0.0134), VEGF (OR=1.27, 95%CI=1.07-1.51, *P*=0.0058), MIP-1A (OR=1.29, 95%CI=1.01-1.65, *P*=0.0350), IFN-G (OR=1.27, 95%CI=1.07- 1.49, *P*=0.0045), FGF-BASIC (OR=1.25, 95%CI=1.06-1.48, *P*=0.007), IL-9 (OR=1.29, 95%CI=1.01-1.63, *P*=0.0356), IL-1RA (OR=1.50, 95%CI=1.18-1.91, *P*=0.0007), IL-10 (OR=1.26, 95%CI=1.07-1.49, *P*=0.0050), TNF-A (OR=1.29, 95%CI=1.01-1.65, *P*=0.0377), IL-12-P70 (OR=1.28, 95%CI=1.09-1.50, *P*=0.0025), and IL-17 (OR=1.22, 95%CI=1.03-1.43, *P*=0.0183).

As to SAH, six InF were increased after SAH (**Figure S30** and **Figure S31**). The level of IL-4, VEGF, IFN-G, IL-10, IL-12_P70, and IL-17 increased after SAH. Specifically, SAH increased the level of IL-4 (OR=1.13, 95%CI=1.06-1.20, *P*=7.10E-05), VEGF (OR=1.10, 95%CI=1.00-1.23, *P*=0.0488), IFN-G (OR=1.12, 95%CI=1.03-1.22, *P*=0.0055), IL-10 (OR=1.11, 95%CI=1.02-1.20, *P*=0.0083), IL-12-P70 (OR=1.15, 95%CI=1.03-1.29, *P*=0.0082), and IL-17 (OR=1.08, 95%CI=1.00-1.17, *P*=0.0464).

In sensitivity analysis, we found that no signs of pleiotropy and heterogeneity were observed (**Table S12**). Leave-one-out analysis of sensitivity analyses showed no significant SNPs for IA, uIA, and SAH (**Figure S32-S33**).

IL-4, VEGF, IFN-G, IL-10, and IL-12-P70 were common InF among IA, uIA, and SAH (**Figure S34**). IL-6, FGF-BASIC, IL-9, and IL-1RA were common InF among IA and uIA. IL-17 was a common inflammation cytokine among uIA and SAH.

**Causal effects of IA, uIA, and SAH on InF in FinnGen in replicate reverse MR analysis**

As shown in **Table S13**, F statistics of these IVs for IA, uIA, and SAH were between 35.49, 30.92, and 37.56, respectively. MR results of causal effects of IA, uIA, and SAH on InF in FinnGen datasets were shown in **Table S14**.

The positive MR results for IA in FinnGen datasets were displayed in **Figure S35** and **Figure S36**. The level of IL-1B was increased after IA (OR=1.03, 95%CI=1.01-1.06, *P*=0.0129). For uIA, all the InF were increased (**Figure S37** and **Figure S38**). Specifically, uIA increased the level of VEGF (OR=1.18, 95%CI=1.04-1.34, *P*=0.0080), MIP-1A (OR=1.24, 95%CI=1.23-1.25, *P*=1.00E-08), SCGF-B (OR=1.06, 95%CI=1.02-1.10, *P*=0.0019), IL-5 (OR=1.08, 95%CI=1.01,1.16- *P*=0.0196), SCF (OR=1.03, 95%CI=1.02,1.04- *P*=1.56E-29), IL_9 (OR=1.21, 95%CI=1.18,1.25- *P*=6.21E-48), MCP-3 (OR=1.34, 95%CI=1.13-1.58, *P*=0.0005), PDGF-BB (OR=1.03, 95%CI=1.01-1.05, *P*=0.0003), G_-CSF (OR=1.08, 95%CI=1.01-1.14, *P*=0.0101), IL-10 (OR=1.13, 95%CI=1.02-1.26, *P*=0.0188), TNF-A (OR=1.25, 95%CI=1.20-1.30, *P*=1.75E-29), IL-7 (OR=1.03, 95%CI=1.01-1.06, *P*=0.0016), IL-2 (OR=1.12, 95%CI=1.01-1.24, *P*=0.0259), and TRAIL (OR=1.03, 95%CI=1.01-1.07, *P*=0.0423). As to SAH, the level of CTACK (OR=1.14, 95%CI=1.00-1.30, *P*=0.0401) was increased, whereas TNF-B (OR=0.88, 95%CI=0.85-0.91, *P*=1.24E-15), HGF (OR=0.91, 95%CI=0.84-0.98, *P*=0.0126), EOTAXIN (OR=0.92, 95%CI=0.85-0.99, *P*=0.0428), SCF (OR=0.89, 95%CI=0.81-0.98, *P*=0.0244), TNF-A (OR=0.91, 95%CI=0.86-0.96, *P*=0.0025), IL_7 (OR=0.90, 95%CI=0.85-0.95, *P*=0.0001), TRAIL (OR=0.94, 95%CI=0.89-0.99, *P*=0.0355), IL_17 (OR=0.93, 95%CI=0.87-0.99, *P*=0.0399), and B-NGF (OR=0.88, 95%CI=0.81-0.95, *P*=0.0011) were decreased (**Figure S39** and **Figure S40**).

In sensitivity analysis, no evidence of pleiotropy and heterogeneity was observed (**Table S15**). Leave-one-out analysis of sensitivity analyses showed no significant SNPs for IA, uIA, and SAH (**Figure S41-S43**).

SCF, TNF_A, IL_7, and TRAIL were common InF between uIA and SAH (**Figure S41**).

**Common InF between reverse MR and replicate reverse MR analysis**

As shown in **Figure S45-S47**, the common cytokines altered by uIA were VEGF, MIP-1A, IL-9, and IL-10 in reverse MR and replicate reverse MR analysis. The common cytokine altered by SAH was IL-17.

**Table S1** The summary-level GWAS data sources in MR analysis

| **Traits** | **PMID** | **Samples** | **Unit** | **Mean age (years)** | **Female** | **Race** | **Consortium/cohorts** | **Web** |
| --- | --- | --- | --- | --- | --- | --- | --- | --- |
| **Exposure** |  |  |  |  |  |  |  |  |
| inflammatory cytokines | 27989323 | 8,293 | SD | Finnish Young Finns Study: 37  FINRISK survey: 60 | NA | European | Finnish Young Finns Study, FINRISK survey | https://data.bris.ac.uk/data/dataset/3g3i5smgghp0s2uvm1doflkx9x |
| **Outcome** |  |  |  |  |  |  |  |  |
| IA | 33199917 | 7,495 | 71,934 | NA | NA | European | ISGC | www.cerebrovascularportal.org |
| uIA | 33199917 | 2,070 | 71,934 | NA | NA | European | ISGC | www.cerebrovascularportal.org |
| SAH | 33199917 | 5,140 | 71,934 | NA | NA | European | ISGC | www.cerebrovascularportal.org |
| **Replicate** |  |  |  |  |  |  |  |  |
| IA | 36653562 | 960 | 284,164 | 53.41 | 59.89% | European | FinnGen | https://r7.risteys.finngen.fi/ |
| uIA | 36653562 | 1,519 | 284,164 | 57.43 | 64.84% | European | FinnGen | https://r7.risteys.finngen.fi/ |
| SAH | 36653562 | 3,201 | 284,164 | 56.50 | 56.16% | European | FinnGen | https://r7.risteys.finngen.fi/ |

GWAS, gene-wide association study; MR, mendelian randomization; IA, intracranial aneurysm; uIA, unruptured intracranial aneurysm; SAH, subarachnoid hemorrhage; SD, standard deviation; NA, not available; ISGC, International Stroke Genetics Consortium

**Table S2** The IVs used in MR analysis

| **exposure** | **SNP** | **effect allele** | **other allele** | **eaf** | **beta** | **se** | **pval** |
| --- | --- | --- | --- | --- | --- | --- | --- |
| B_NGF | rs71641308 | T | C | 0.0995 | 0.1969 | 0.0429 | 4.42E-06 |
| B_NGF | rs28637706 | T | G | 0.3140 | -0.1554 | 0.0261 | 2.72E-09 |
| B_NGF | rs7970581 | T | G | 0.2662 | 0.1358 | 0.0280 | 1.22E-06 |
| B_NGF | rs73472576 | T | C | 0.4570 | -0.1146 | 0.0251 | 4.81E-06 |
| CTACK | rs7333764 | T | C | 0.0412 | 0.2811 | 0.0591 | 2.00E-06 |
| CTACK | rs118084576 | A | G | 0.0112 | 0.5675 | 0.1226 | 3.66E-06 |
| CTACK | rs62578137 | T | C | 0.2421 | -0.1311 | 0.0286 | 4.66E-06 |
| CTACK | rs135564 | A | G | 0.2632 | -0.1672 | 0.0267 | 3.59E-10 |
| CTACK | rs76395525 | A | G | 0.0118 | 0.5193 | 0.1081 | 1.55E-06 |
| CTACK | rs116303454 | A | G | 0.0218 | 0.3754 | 0.0810 | 3.58E-06 |
| CTACK | rs57338032 | A | G | 0.8287 | 0.1443 | 0.0316 | 4.83E-06 |
| CTACK | rs60247384 | T | C | 0.3512 | 0.1128 | 0.0245 | 4.30E-06 |
| CTACK | rs72729450 | T | C | 0.0123 | -0.5123 | 0.1094 | 2.81E-06 |
| CTACK | rs2070074 | A | G | 0.8910 | 0.4401 | 0.0372 | 2.60E-32 |
| CTACK | rs141331414 | A | G | 0.0914 | 0.1977 | 0.0415 | 1.89E-06 |
| CTACK | rs57789542 | T | C | 0.9951 | -0.7687 | 0.1659 | 3.58E-06 |
| CTACK | rs55764737 | T | C | 0.9846 | 0.5424 | 0.0967 | 2.01E-08 |
| EOTAXIN | rs12075 | A | G | 0.5229 | 0.1692 | 0.0155 | 1.21E-27 |
| EOTAXIN | rs79722574 | T | C | 0.1422 | -0.1092 | 0.0227 | 1.50E-06 |
| EOTAXIN | rs75426604 | A | C | 0.0876 | -0.1371 | 0.0291 | 2.40E-06 |
| EOTAXIN | rs187131 | C | G | 0.8902 | 0.1264 | 0.0253 | 5.74E-07 |
| EOTAXIN | rs11087905 | A | C | 0.3778 | 0.0954 | 0.0188 | 4.07E-07 |
| EOTAXIN | rs80341932 | A | G | 0.8094 | 0.1010 | 0.0204 | 7.40E-07 |
| EOTAXIN | rs59808887 | T | C | 0.0601 | -0.1698 | 0.0356 | 1.89E-06 |
| EOTAXIN | rs2027855 | T | C | 0.6153 | 0.0743 | 0.0162 | 4.27E-06 |
| EOTAXIN | rs2228467 | T | C | 0.9215 | -0.4154 | 0.0291 | 3.47E-46 |
| EOTAXIN | rs5754733 | A | C | 0.8306 | -0.1050 | 0.0213 | 8.20E-07 |
| EOTAXIN | rs112347425 | T | C | 0.0978 | 0.1595 | 0.0276 | 7.77E-09 |
| EOTAXIN | rs745331 | A | G | 0.6825 | -0.0821 | 0.0176 | 3.04E-06 |
| EOTAXIN | rs2211994 | T | C | 0.2760 | 0.0876 | 0.0177 | 6.98E-07 |
| EOTAXIN | rs2024050 | A | G | 0.0739 | 0.1640 | 0.0302 | 5.47E-08 |
| EOTAXIN | rs147287945 | A | G | 0.0741 | -0.1512 | 0.0313 | 1.36E-06 |
| EOTAXIN | rs9317045 | A | C | 0.8615 | 0.1172 | 0.0236 | 6.95E-07 |
| FGF_BASIC | rs13412535 | A | G | 0.1856 | -0.1129 | 0.0224 | 4.76E-07 |
| FGF_BASIC | rs75168112 | T | C | 0.8097 | -0.1024 | 0.0214 | 1.64E-06 |
| FGF_BASIC | rs61990749 | C | G | 0.1545 | 0.1124 | 0.0228 | 8.23E-07 |
| FGF_BASIC | rs9903590 | T | C | 0.8953 | 0.1281 | 0.0267 | 1.62E-06 |
| FGF_BASIC | rs116745220 | A | G | 0.9951 | -0.6176 | 0.1324 | 3.09E-06 |
| FGF_BASIC | rs145577605 | A | G | 0.0378 | 0.2043 | 0.0427 | 1.67E-06 |
| FGF_BASIC | rs78873483 | A | G | 0.1025 | 0.1286 | 0.0282 | 4.98E-06 |
| G_CSF | rs117261691 | T | C | 0.0894 | 0.1318 | 0.0288 | 4.67E-06 |
| G_CSF | rs115256310 | A | G | 0.9954 | -0.6788 | 0.1359 | 5.85E-07 |
| G_CSF | rs74148555 | T | C | 0.0144 | -0.3771 | 0.0753 | 5.59E-07 |
| G_CSF | rs11903143 | A | G | 0.7066 | 0.0889 | 0.0175 | 3.78E-07 |
| G_CSF | rs586313 | T | C | 0.2417 | -0.0883 | 0.0187 | 2.36E-06 |
| G_CSF | rs2324653 | C | G | 0.5740 | -0.0812 | 0.0160 | 4.17E-07 |
| G_CSF | rs145756094 | C | G | 0.9962 | -0.7323 | 0.1479 | 7.40E-07 |
| G_CSF | rs2671444 | A | G | 0.6506 | -0.0776 | 0.0166 | 2.86E-06 |
| G_CSF | rs76287671 | T | C | 0.2333 | 0.0894 | 0.0189 | 2.19E-06 |
| G_CSF | rs77318030 | T | C | 0.9563 | -0.2031 | 0.0427 | 2.02E-06 |
| GROA | rs12075 | A | G | 0.5354 | 0.3724 | 0.0236 | 3.46E-56 |
| GROA | rs1113500 | T | G | 0.5981 | 0.1162 | 0.0243 | 1.72E-06 |
| GROA | rs76390238 | C | G | 0.0095 | 0.6223 | 0.1352 | 4.14E-06 |
| GROA | rs185768063 | A | G | 0.9735 | 0.4038 | 0.0760 | 1.06E-07 |
| GROA | rs140734053 | A | G | 0.0066 | 0.7333 | 0.1545 | 2.07E-06 |
| GROA | rs62024303 | A | G | 0.9617 | -0.3013 | 0.0660 | 4.91E-06 |
| GROA | rs78653452 | T | G | 0.0071 | -0.7395 | 0.1559 | 2.09E-06 |
| GROA | rs118158560 | A | G | 0.0430 | 0.2761 | 0.0592 | 3.09E-06 |
| GROA | rs508977 | T | G | 0.7566 | -0.3838 | 0.0279 | 4.57E-43 |
| GROA | rs188345231 | T | C | 0.0102 | 0.6177 | 0.1322 | 2.97E-06 |
| GROA | rs114991247 | T | C | 0.9226 | -0.2202 | 0.0463 | 1.97E-06 |
| HGF | rs1617833 | C | G | 0.6189 | -0.0749 | 0.0160 | 2.93E-06 |
| HGF | rs13412535 | A | G | 0.1892 | -0.1043 | 0.0213 | 9.67E-07 |
| HGF | rs180840563 | A | T | 0.0402 | -0.2022 | 0.0416 | 1.15E-06 |
| HGF | rs57146176 | A | G | 0.7998 | -0.0987 | 0.0208 | 2.18E-06 |
| HGF | rs4245058 | T | C | 0.0619 | -0.1552 | 0.0331 | 2.68E-06 |
| HGF | rs5745687 | T | C | 0.0375 | -0.3008 | 0.0404 | 9.92E-14 |
| HGF | rs2003620 | T | C | 0.0270 | 0.2277 | 0.0487 | 2.98E-06 |
| HGF | rs11060254 | A | G | 0.3306 | -0.0765 | 0.0166 | 3.97E-06 |
| HGF | rs3748034 | T | G | 0.1278 | 0.1529 | 0.0233 | 5.21E-11 |
| IFN_G | rs10481651 | A | G | 0.6116 | -0.0793 | 0.0168 | 2.18E-06 |
| IFN_G | rs2073438 | A | G | 0.2506 | 0.0920 | 0.0188 | 9.55E-07 |
| IFN_G | rs60059008 | A | G | 0.6862 | 0.0852 | 0.0176 | 1.30E-06 |
| IFN_G | rs11843756 | T | G | 0.9542 | 0.1812 | 0.0391 | 3.62E-06 |
| IFN_G | rs73479333 | C | G | 0.1354 | -0.1123 | 0.0240 | 2.82E-06 |
| IFN_G | rs78296352 | T | G | 0.0186 | 0.3419 | 0.0650 | 1.42E-07 |
| IFN_G | rs74148555 | T | C | 0.0144 | -0.3771 | 0.0770 | 9.86E-07 |
| IFN_G | rs12420286 | T | C | 0.9699 | 0.2357 | 0.0500 | 2.45E-06 |
| IFN_G | rs2188420 | C | G | 0.7912 | 0.1005 | 0.0201 | 5.90E-07 |
| IFN_G | rs115729819 | A | G | 0.9716 | 0.2511 | 0.0514 | 1.05E-06 |
| IFN_G | rs10761731 | A | T | 0.6214 | -0.0813 | 0.0167 | 1.07E-06 |
| IFN_G | rs113600793 | A | C | 0.0631 | 0.1871 | 0.0371 | 4.43E-07 |
| IL_10 | rs41282660 | A | G | 0.8646 | -0.1169 | 0.0254 | 4.23E-06 |
| IL_10 | rs6085948 | A | G | 0.7946 | 0.0977 | 0.0202 | 1.28E-06 |
| IL_10 | rs3002131 | C | G | 0.1195 | 0.1191 | 0.0260 | 4.59E-06 |
| IL_10 | rs383684 | A | G | 0.7555 | 0.0920 | 0.0197 | 3.17E-06 |
| IL_10 | rs339203 | T | C | 0.8359 | 0.0954 | 0.0203 | 2.75E-06 |
| IL_10 | rs282258 | T | C | 0.4311 | 0.0993 | 0.0162 | 8.63E-10 |
| IL_10 | rs6921438 | A | G | 0.4815 | -0.2876 | 0.0166 | 1.38E-67 |
| IL_10 | rs2375980 | C | G | 0.5552 | 0.0844 | 0.0165 | 2.97E-07 |
| IL_10 | rs10493718 | A | C | 0.1603 | -0.1081 | 0.0222 | 1.07E-06 |
| IL_10 | rs3025021 | T | C | 0.3459 | 0.0913 | 0.0194 | 2.61E-06 |
| IL_10 | rs2086656 | T | C | 0.6621 | -0.0800 | 0.0170 | 2.59E-06 |
| IL_10 | rs10457128 | A | G | 0.6390 | -0.0854 | 0.0172 | 6.96E-07 |
| IL_10 | rs6799107 | T | C | 0.8089 | -0.0950 | 0.0206 | 3.99E-06 |
| IL_10 | rs7088799 | T | G | 0.6191 | -0.0815 | 0.0166 | 9.35E-07 |
| IL_10 | rs10888839 | C | G | 0.1208 | 0.1203 | 0.0250 | 1.56E-06 |
| IL_10 | rs1530455 | T | C | 0.3523 | 0.0820 | 0.0174 | 2.53E-06 |
| IL_10 | rs111913416 | A | T | 0.5300 | -0.0846 | 0.0172 | 8.16E-07 |
| IL_12_P70 | rs2123852 | T | C | 0.2039 | 0.0942 | 0.0204 | 3.73E-06 |
| IL_12_P70 | rs782107 | A | G | 0.4709 | 0.0765 | 0.0156 | 9.13E-07 |
| IL_12_P70 | rs72831623 | A | G | 0.0645 | 0.1929 | 0.0367 | 1.51E-07 |
| IL_12_P70 | rs6993770 | A | T | 0.7786 | 0.0918 | 0.0188 | 1.06E-06 |
| IL_12_P70 | rs282258 | T | C | 0.4279 | 0.0726 | 0.0156 | 3.28E-06 |
| IL_12_P70 | rs6921438 | A | G | 0.4834 | -0.3784 | 0.0160 | 5.78E-124 |
| IL_12_P70 | rs34291323 | T | C | 0.6454 | 0.0954 | 0.0198 | 1.49E-06 |
| IL_12_P70 | rs71361173 | T | G | 0.8780 | 0.1105 | 0.0238 | 3.57E-06 |
| IL_12_P70 | rs9472183 | A | G | 0.4566 | -0.1006 | 0.0157 | 1.38E-10 |
| IL_12_P70 | rs2375980 | C | G | 0.5561 | 0.0952 | 0.0159 | 1.94E-09 |
| IL_12_P70 | rs273702 | A | G | 0.9015 | -0.1270 | 0.0270 | 2.52E-06 |
| IL_12_P70 | rs13209117 | A | G | 0.2453 | 0.0981 | 0.0186 | 1.27E-07 |
| IL_12_P70 | rs41282644 | A | G | 0.0849 | 0.1401 | 0.0303 | 3.74E-06 |
| IL_12_P70 | rs6532374 | T | C | 0.8525 | -0.1033 | 0.0226 | 4.61E-06 |
| IL_12_P70 | rs10761731 | A | T | 0.6227 | -0.0965 | 0.0161 | 2.12E-09 |
| IL_13 | rs7073807 | T | C | 0.1351 | 0.1618 | 0.0354 | 4.77E-06 |
| IL_13 | rs138854806 | A | G | 0.0247 | -0.4204 | 0.0839 | 5.45E-07 |
| IL_13 | rs77955971 | A | C | 0.0247 | 0.4408 | 0.0868 | 3.76E-07 |
| IL_13 | rs12623722 | A | G | 0.3054 | -0.1189 | 0.0257 | 3.61E-06 |
| IL_13 | rs6921438 | A | G | 0.4800 | -0.4139 | 0.0242 | 1.28E-65 |
| IL_13 | rs76339001 | A | T | 0.9767 | -0.4375 | 0.0886 | 7.92E-07 |
| IL_13 | rs10995615 | T | C | 0.8577 | -0.1591 | 0.0341 | 3.12E-06 |
| IL_13 | rs27949 | T | C | 0.6670 | -0.1144 | 0.0250 | 4.83E-06 |
| IL_13 | rs6799107 | T | C | 0.8071 | -0.1472 | 0.0299 | 8.66E-07 |
| IL_13 | rs117795020 | A | G | 0.0304 | -0.3584 | 0.0716 | 5.48E-07 |
| IL_13 | rs75383097 | C | G | 0.0132 | -0.5369 | 0.1160 | 3.70E-06 |
| IL_13 | rs139083458 | T | C | 0.0034 | 0.9995 | 0.2110 | 2.17E-06 |
| IL_13 | rs147747784 | C | G | 0.0285 | 0.3690 | 0.0765 | 1.44E-06 |
| IL_16 | rs1801020 | A | G | 0.2655 | 0.1678 | 0.0271 | 5.63E-10 |
| IL_16 | rs116135478 | A | G | 0.9933 | 0.8296 | 0.1637 | 4.05E-07 |
| IL_16 | rs144691581 | A | G | 0.0197 | 0.4929 | 0.0958 | 2.67E-07 |
| IL_16 | rs117916513 | A | G | 0.0173 | -0.4713 | 0.0982 | 1.61E-06 |
| IL_16 | rs9706053 | T | C | 0.0173 | 0.4412 | 0.0928 | 1.98E-06 |
| IL_16 | rs117217798 | T | C | 0.0889 | -0.2064 | 0.0440 | 2.77E-06 |
| IL_16 | rs4253283 | T | C | 0.3087 | 0.1506 | 0.0260 | 7.22E-09 |
| IL_16 | rs4778636 | A | G | 0.0381 | -0.7286 | 0.0630 | 6.21E-31 |
| IL_16 | rs1255143 | T | C | 0.5695 | 0.1387 | 0.0241 | 8.53E-09 |
| IL_17 | rs17282552 | T | C | 0.9520 | -0.2026 | 0.0403 | 4.88E-07 |
| IL_17 | rs12735700 | T | G | 0.1925 | -0.0943 | 0.0206 | 4.50E-06 |
| IL_17 | rs78296352 | T | G | 0.0186 | 0.2949 | 0.0645 | 4.81E-06 |
| IL_17 | rs149738638 | T | C | 0.9382 | -0.1553 | 0.0337 | 4.11E-06 |
| IL_17 | rs117556572 | T | C | 0.0158 | -0.5256 | 0.1097 | 1.66E-06 |
| IL_17 | rs148562661 | C | G | 0.9605 | 0.2161 | 0.0434 | 6.37E-07 |
| IL_17 | rs184080173 | T | C | 0.9693 | 0.2360 | 0.0471 | 5.39E-07 |
| IL_17 | rs9568764 | C | G | 0.2756 | 0.0825 | 0.0180 | 4.68E-06 |
| IL_17 | rs17106604 | T | C | 0.1539 | 0.1119 | 0.0225 | 6.23E-07 |
| IL_17 | rs11640734 | C | G | 0.8673 | -0.1150 | 0.0240 | 1.61E-06 |
| IL_17 | rs1530455 | T | C | 0.3534 | 0.1088 | 0.0173 | 3.29E-10 |
| IL_18 | rs12420140 | A | G | 0.2830 | -0.2479 | 0.0261 | 1.95E-21 |
| IL_18 | rs117266781 | T | C | 0.0073 | 0.7051 | 0.1436 | 9.18E-07 |
| IL_18 | rs4885797 | C | G | 0.4507 | -0.1093 | 0.0238 | 4.39E-06 |
| IL_18 | rs78716465 | A | G | 0.0330 | 0.3173 | 0.0679 | 2.98E-06 |
| IL_18 | rs17229943 | A | C | 0.9176 | -0.3076 | 0.0463 | 3.06E-11 |
| IL_18 | rs7444013 | A | G | 0.0169 | -0.5318 | 0.0955 | 2.59E-08 |
| IL_18 | rs143370787 | C | G | 0.9554 | -0.3447 | 0.0660 | 1.75E-07 |
| IL_18 | rs385076 | T | C | 0.3444 | -0.2472 | 0.0247 | 1.56E-23 |
| IL_18 | rs1979967 | T | C | 0.2157 | 0.1400 | 0.0285 | 8.72E-07 |
| IL_18 | rs10414578 | T | C | 0.1295 | -0.1817 | 0.0347 | 1.64E-07 |
| IL_18 | rs78623212 | T | C | 0.0053 | 0.8322 | 0.1676 | 6.82E-07 |
| IL_18 | rs116383510 | A | C | 0.9868 | -0.5412 | 0.1052 | 2.70E-07 |
| IL_18 | rs4482818 | A | G | 0.6090 | 0.1233 | 0.0243 | 4.11E-07 |
| IL_18 | rs610473 | A | G | 0.3522 | 0.1274 | 0.0242 | 1.43E-07 |
| IL_1B | rs61335305 | A | C | 0.0189 | 0.4333 | 0.0928 | 3.02E-06 |
| IL_1B | rs62015704 | A | G | 0.8688 | 0.1786 | 0.0372 | 1.62E-06 |
| IL_1B | rs143319329 | T | C | 0.0209 | 0.4357 | 0.0930 | 2.84E-06 |
| IL_1B | rs115242021 | A | C | 0.0601 | 0.2795 | 0.0553 | 4.25E-07 |
| IL_1RA | rs187166731 | T | C | 0.0582 | -0.2424 | 0.0504 | 1.55E-06 |
| IL_1RA | rs61335305 | A | C | 0.0182 | 0.4315 | 0.0904 | 1.81E-06 |
| IL_1RA | rs6699436 | A | G | 0.0995 | -0.1858 | 0.0404 | 4.37E-06 |
| IL_1RA | rs11869294 | C | G | 0.9205 | -0.2286 | 0.0470 | 1.13E-06 |
| IL_1RA | rs3876037 | A | G | 0.6783 | 0.1234 | 0.0270 | 4.73E-06 |
| IL_1RA | rs4441609 | T | C | 0.4757 | 0.1056 | 0.0231 | 4.75E-06 |
| IL_1RA | rs56134659 | A | G | 0.4835 | -0.1109 | 0.0236 | 2.56E-06 |
| IL_1RA | rs1054402 | T | C | 0.2506 | 0.1325 | 0.0269 | 8.20E-07 |
| IL_1RA | rs11627423 | A | C | 0.6414 | 0.1178 | 0.0246 | 1.65E-06 |
| IL_1RA | rs147747784 | C | G | 0.0284 | 0.3582 | 0.0754 | 2.04E-06 |
| IL_2 | rs13412535 | A | G | 0.1886 | 0.1740 | 0.0331 | 1.45E-07 |
| IL_2 | rs61335305 | A | C | 0.0184 | 0.4439 | 0.0913 | 1.16E-06 |
| IL_2 | rs7615304 | A | G | 0.4531 | -0.1139 | 0.0240 | 2.16E-06 |
| IL_2 | rs16836080 | A | G | 0.3355 | 0.1158 | 0.0253 | 4.84E-06 |
| IL_2 | rs170117 | T | C | 0.1377 | -0.1637 | 0.0347 | 2.44E-06 |
| IL_2 | rs2690020 | A | G | 0.5177 | 0.1158 | 0.0245 | 2.27E-06 |
| IL_2 | rs62124990 | T | G | 0.0083 | -0.7013 | 0.1490 | 2.50E-06 |
| IL_2 | rs4634519 | A | G | 0.7379 | -0.1249 | 0.0268 | 3.18E-06 |
| IL_2RA | rs115360066 | A | G | 0.8882 | 0.1776 | 0.0377 | 2.42E-06 |
| IL_2RA | rs4733117 | A | C | 0.7938 | 0.1439 | 0.0291 | 7.91E-07 |
| IL_2RA | rs12722497 | A | C | 0.0624 | 0.6287 | 0.0482 | 7.98E-39 |
| IL_2RA | rs759244 | A | T | 0.3940 | -0.1094 | 0.0238 | 4.26E-06 |
| IL_2RA | rs28441585 | A | T | 0.7688 | 0.1269 | 0.0271 | 2.93E-06 |
| IL_2RA | rs185231391 | T | C | 0.9945 | 0.8568 | 0.1803 | 2.00E-06 |
| IL_2RA | rs11241559 | T | G | 0.2643 | -0.1240 | 0.0264 | 2.75E-06 |
| IL_2RA | rs117244812 | A | G | 0.0079 | -0.7187 | 0.1493 | 1.47E-06 |
| IL_2RA | rs12799226 | T | C | 0.2361 | -0.1285 | 0.0277 | 3.56E-06 |
| IL_4 | rs17713451 | A | G | 0.1149 | 0.1255 | 0.0252 | 6.41E-07 |
| IL_4 | rs79597994 | T | C | 0.0049 | -0.5855 | 0.1271 | 4.06E-06 |
| IL_4 | rs73023729 | A | G | 0.0519 | -0.1796 | 0.0365 | 8.56E-07 |
| IL_4 | rs10512267 | T | C | 0.5752 | -0.0824 | 0.0160 | 2.73E-07 |
| IL_4 | rs2073438 | A | G | 0.2513 | 0.0847 | 0.0183 | 3.73E-06 |
| IL_4 | rs6969391 | T | C | 0.6589 | 0.0767 | 0.0166 | 3.59E-06 |
| IL_4 | rs2849346 | C | G | 0.4871 | 0.0765 | 0.0164 | 3.22E-06 |
| IL_4 | rs9941733 | A | G | 0.8355 | 0.1156 | 0.0229 | 4.33E-07 |
| IL_4 | rs9508291 | T | C | 0.9495 | -0.1680 | 0.0358 | 2.67E-06 |
| IL_4 | rs6765768 | A | G | 0.6540 | 0.0796 | 0.0167 | 1.85E-06 |
| IL_4 | rs117146485 | T | C | 0.9837 | -0.2856 | 0.0625 | 4.95E-06 |
| IL_4 | rs12238729 | T | C | 0.0162 | 0.5271 | 0.1096 | 1.51E-06 |
| IL_4 | rs116705532 | T | G | 0.9925 | -0.4675 | 0.0978 | 1.73E-06 |
| IL_4 | rs13106889 | A | T | 0.1648 | -0.1186 | 0.0224 | 1.22E-07 |
| IL_4 | rs7613691 | A | G | 0.9482 | 0.1787 | 0.0382 | 2.96E-06 |
| IL_5 | rs6737109 | T | C | 0.5190 | 0.1135 | 0.0246 | 3.81E-06 |
| IL_5 | rs148634917 | A | G | 0.9860 | -0.5170 | 0.1087 | 1.97E-06 |
| IL_5 | rs7578892 | C | G | 0.4852 | -0.1175 | 0.0247 | 2.00E-06 |
| IL_5 | rs9472168 | A | G | 0.5389 | 0.1568 | 0.0253 | 5.42E-10 |
| IL_5 | rs73040130 | T | C | 0.9388 | 0.2745 | 0.0525 | 1.71E-07 |
| IL_5 | rs28793375 | T | C | 0.1298 | 0.1697 | 0.0362 | 2.75E-06 |
| IL_5 | rs11680908 | A | G | 0.9485 | 0.2593 | 0.0552 | 2.62E-06 |
| IL_5 | rs72831687 | A | G | 0.0137 | -0.5337 | 0.1104 | 1.32E-06 |
| IL_5 | rs74811276 | A | G | 0.0761 | 0.2170 | 0.0471 | 4.08E-06 |
| IL_6 | rs13412535 | A | G | 0.1890 | -0.1186 | 0.0214 | 3.14E-08 |
| IL_6 | rs72831623 | A | G | 0.0645 | 0.1970 | 0.0369 | 9.29E-08 |
| IL_6 | rs4684700 | T | C | 0.5142 | -0.0747 | 0.0162 | 3.91E-06 |
| IL_6 | rs76856708 | T | C | 0.9856 | 0.3360 | 0.0697 | 1.43E-06 |
| IL_6 | rs73273528 | T | C | 0.0211 | 0.2680 | 0.0553 | 1.25E-06 |
| IL_6 | rs113098456 | A | G | 0.0725 | -0.1553 | 0.0339 | 4.64E-06 |
| IL_6 | rs10982213 | A | G | 0.2723 | -0.0849 | 0.0176 | 1.35E-06 |
| IL_6 | rs1333040 | T | C | 0.4508 | 0.0747 | 0.0157 | 1.99E-06 |
| IL_6 | rs2404476 | A | G | 0.5184 | 0.0734 | 0.0156 | 2.68E-06 |
| IL_6 | rs75101555 | C | G | 0.9895 | -0.3625 | 0.0781 | 3.44E-06 |
| IL_6 | rs10752777 | A | T | 0.1273 | 0.1083 | 0.0235 | 4.17E-06 |
| IL_7 | rs62006410 | T | C | 0.2450 | -0.1492 | 0.0302 | 7.59E-07 |
| IL_7 | rs115215018 | T | C | 0.0101 | 0.5985 | 0.1308 | 4.76E-06 |
| IL_7 | rs10196226 | A | G | 0.1643 | 0.1538 | 0.0327 | 2.50E-06 |
| IL_7 | rs17091524 | T | C | 0.9849 | 0.5092 | 0.1015 | 5.24E-07 |
| IL_7 | rs218247 | C | G | 0.2258 | -0.1343 | 0.0285 | 2.42E-06 |
| IL_7 | rs6921438 | A | G | 0.4814 | -0.3204 | 0.0246 | 8.71E-39 |
| IL_7 | rs117509142 | T | C | 0.9569 | -0.3213 | 0.0684 | 2.60E-06 |
| IL_7 | rs141425475 | T | C | 0.9814 | -0.4801 | 0.1018 | 2.39E-06 |
| IL_7 | rs78346957 | A | G | 0.0161 | 0.4632 | 0.1008 | 4.30E-06 |
| IL_7 | rs28793375 | T | C | 0.1305 | 0.1644 | 0.0360 | 4.87E-06 |
| IL_7 | rs77981494 | T | C | 0.9840 | -0.5201 | 0.1055 | 8.23E-07 |
| IL_7 | rs1958987 | T | C | 0.7051 | 0.1261 | 0.0263 | 1.60E-06 |
| IL_7 | rs147747784 | C | G | 0.0287 | 0.4110 | 0.0777 | 1.22E-07 |
| IL_8 | rs141926526 | A | C | 0.9916 | -0.6221 | 0.1308 | 1.96E-06 |
| IL_8 | rs12075 | A | G | 0.5353 | 0.1148 | 0.0235 | 9.97E-07 |
| IL_8 | rs116726256 | T | C | 0.9343 | -0.2247 | 0.0489 | 4.26E-06 |
| IL_8 | rs183628733 | T | C | 0.9917 | 0.6547 | 0.1417 | 3.82E-06 |
| IL_8 | rs12438669 | A | C | 0.6834 | -0.1182 | 0.0252 | 2.60E-06 |
| IL_8 | rs2673604 | A | C | 0.6829 | -0.1180 | 0.0254 | 3.29E-06 |
| IL_8 | rs3786107 | A | G | 0.9287 | 0.2463 | 0.0517 | 1.94E-06 |
| IL_8 | rs75840288 | A | C | 0.9839 | 0.5125 | 0.1121 | 4.85E-06 |
| IL_9 | rs4880409 | T | C | 0.9664 | -0.3552 | 0.0716 | 6.95E-07 |
| IL_9 | rs117807175 | C | G | 0.0121 | -0.5225 | 0.1106 | 2.33E-06 |
| IL_9 | rs41294750 | T | C | 0.0308 | 0.3442 | 0.0736 | 2.92E-06 |
| IL_9 | rs76963786 | T | C | 0.0462 | -0.2856 | 0.0556 | 2.78E-07 |
| IL_9 | rs73443903 | A | C | 0.0685 | 0.2162 | 0.0460 | 2.57E-06 |
| IL_9 | rs3736858 | C | G | 0.7976 | -0.1351 | 0.0291 | 3.37E-06 |
| IP_10 | rs397816 | T | C | 0.5891 | 0.1211 | 0.0248 | 1.03E-06 |
| IP_10 | rs75970138 | A | G | 0.0139 | -0.4845 | 0.1037 | 2.99E-06 |
| IP_10 | rs6707974 | A | G | 0.8626 | 0.1574 | 0.0337 | 3.03E-06 |
| IP_10 | rs9450351 | T | C | 0.9387 | -0.2651 | 0.0488 | 5.48E-08 |
| IP_10 | rs79848609 | A | C | 0.9449 | 0.2514 | 0.0535 | 2.64E-06 |
| IP_10 | rs34383175 | T | C | 0.0350 | -0.3196 | 0.0653 | 9.90E-07 |
| IP_10 | rs143799975 | A | G | 0.9949 | -0.7551 | 0.1638 | 4.01E-06 |
| IP_10 | rs4862111 | T | C | 0.2313 | 0.1448 | 0.0317 | 4.84E-06 |
| IP_10 | rs7645625 | T | G | 0.5724 | -0.1116 | 0.0236 | 2.19E-06 |
| IP_10 | rs113831257 | A | G | 0.0417 | 0.3639 | 0.0641 | 1.39E-08 |
| IP_10 | rs8112909 | A | G | 0.8159 | -0.1390 | 0.0297 | 2.96E-06 |
| M_CSF | rs9387100 | T | C | 0.4291 | -0.1350 | 0.0290 | 3.34E-06 |
| M_CSF | rs78296352 | T | G | 0.0195 | 0.5220 | 0.1110 | 2.58E-06 |
| M_CSF | rs34089869 | T | C | 0.1039 | 0.2194 | 0.0462 | 2.08E-06 |
| M_CSF | rs62294910 | A | G | 0.0504 | 0.3472 | 0.0687 | 4.38E-07 |
| M_CSF | rs56367447 | T | C | 0.0305 | -0.4878 | 0.0876 | 2.57E-08 |
| M_CSF | rs117867915 | T | C | 0.9793 | 0.5224 | 0.1096 | 1.87E-06 |
| M_CSF | rs116274860 | T | G | 0.9908 | 0.8262 | 0.1739 | 2.03E-06 |
| M_CSF | rs12962919 | T | C | 0.0644 | 0.3025 | 0.0659 | 4.39E-06 |
| M_CSF | rs4269021 | C | G | 0.9101 | -0.2459 | 0.0504 | 1.05E-06 |
| M_CSF | rs11963606 | C | G | 0.9826 | -0.5353 | 0.1170 | 4.73E-06 |
| M_CSF | rs72723242 | T | G | 0.0177 | -0.4969 | 0.1083 | 4.43E-06 |
| MCP_1_MCAF | rs10744620 | T | C | 0.3748 | 0.0783 | 0.0161 | 1.12E-06 |
| MCP_1_MCAF | rs12075 | A | G | 0.5230 | 0.2186 | 0.0154 | 1.36E-45 |
| MCP_1_MCAF | rs7632755 | A | G | 0.0647 | 0.2984 | 0.0315 | 2.79E-21 |
| MCP_1_MCAF | rs7517040 | A | G | 0.2334 | -0.0970 | 0.0190 | 3.41E-07 |
| MCP_1_MCAF | rs7033586 | A | G | 0.9680 | -0.2200 | 0.0467 | 2.43E-06 |
| MCP_1_MCAF | rs111995966 | T | G | 0.9297 | 0.1428 | 0.0309 | 3.79E-06 |
| MCP_1_MCAF | rs7197349 | A | G | 0.8124 | 0.0971 | 0.0206 | 2.40E-06 |
| MCP_1_MCAF | rs2288370 | T | C | 0.6304 | -0.1036 | 0.0162 | 1.56E-10 |
| MCP_1_MCAF | rs12073356 | A | G | 0.0686 | -0.1436 | 0.0310 | 3.49E-06 |
| MCP_1_MCAF | rs56212190 | T | C | 0.0519 | 0.1799 | 0.0372 | 1.32E-06 |
| MCP_1_MCAF | rs146522229 | T | C | 0.0047 | -0.5942 | 0.1161 | 3.09E-07 |
| MCP_1_MCAF | rs143815843 | A | G | 0.0315 | -0.2049 | 0.0447 | 4.61E-06 |
| MCP_1_MCAF | rs2036297 | A | G | 0.3840 | 0.1182 | 0.0160 | 1.30E-13 |
| MCP_1_MCAF | rs9317045 | A | C | 0.8618 | 0.1157 | 0.0235 | 8.43E-07 |
| MCP_3 | rs2838065 | A | G | 0.2719 | -0.2210 | 0.0479 | 3.92E-06 |
| MCP_3 | rs28394764 | A | T | 0.9696 | 0.5970 | 0.1282 | 3.19E-06 |
| MCP_3 | rs3129806 | T | C | 0.5729 | -0.1975 | 0.0433 | 4.98E-06 |
| MCP_3 | rs10892381 | T | C | 0.6629 | 0.2432 | 0.0473 | 2.69E-07 |
| MCP_3 | rs62492260 | T | G | 0.1886 | -0.2802 | 0.0578 | 1.23E-06 |
| MCP_3 | rs117286643 | A | G | 0.0225 | 0.6934 | 0.1474 | 2.54E-06 |
| MIF | rs2330634 | C | G | 0.3681 | 0.1549 | 0.0249 | 4.57E-10 |
| MIF | rs2294689 | C | G | 0.3018 | -0.1338 | 0.0287 | 3.04E-06 |
| MIF | rs11551183 | C | G | 0.9756 | 0.3666 | 0.0795 | 4.00E-06 |
| MIF | rs113218956 | A | G | 0.0044 | -0.8789 | 0.1876 | 2.82E-06 |
| MIF | rs141009259 | T | C | 0.9886 | -0.6194 | 0.1285 | 1.44E-06 |
| MIF | rs78098071 | T | C | 0.9809 | -0.4583 | 0.0915 | 5.51E-07 |
| MIF | rs12594190 | A | G | 0.7008 | 0.1321 | 0.0266 | 6.85E-07 |
| MIF | rs3814097 | A | G | 0.5533 | -0.1163 | 0.0251 | 3.55E-06 |
| MIF | rs35890933 | T | G | 0.8474 | 0.1676 | 0.0365 | 4.46E-06 |
| MIF | rs13142904 | T | C | 0.0896 | -0.2232 | 0.0425 | 1.47E-07 |
| MIG | rs11177248 | A | G | 0.0351 | 0.3157 | 0.0667 | 2.22E-06 |
| MIG | rs139010077 | T | C | 0.0169 | 0.4337 | 0.0943 | 4.19E-06 |
| MIG | rs6679677 | A | C | 0.1458 | 0.1628 | 0.0327 | 6.51E-07 |
| MIG | rs1796086 | T | C | 0.9127 | -0.2172 | 0.0400 | 5.62E-08 |
| MIG | rs191555775 | A | T | 0.9090 | 0.2279 | 0.0412 | 3.28E-08 |
| MIG | rs816960 | T | C | 0.3692 | -0.1179 | 0.0242 | 1.15E-06 |
| MIG | rs112861654 | A | G | 0.9454 | -0.2682 | 0.0527 | 3.64E-07 |
| MIG | rs62562991 | A | G | 0.0097 | 0.6239 | 0.1259 | 7.24E-07 |
| MIG | rs13143163 | C | G | 0.0512 | 0.2735 | 0.0582 | 2.62E-06 |
| MIG | rs111607343 | A | G | 0.0134 | -0.5235 | 0.1119 | 2.93E-06 |
| MIG | rs77086208 | T | C | 0.0286 | 0.3270 | 0.0694 | 2.50E-06 |
| MIG | rs55876513 | T | G | 0.7131 | 0.1638 | 0.0254 | 1.05E-10 |
| MIG | rs117831247 | T | C | 0.0056 | -0.8819 | 0.1730 | 3.45E-07 |
| MIP_1A | rs184154340 | A | G | 0.0304 | 0.3251 | 0.0689 | 2.40E-06 |
| MIP_1A | rs6900267 | A | C | 0.9308 | -0.2472 | 0.0515 | 1.60E-06 |
| MIP_1A | rs12690897 | A | G | 0.2920 | 0.1215 | 0.0260 | 3.07E-06 |
| MIP_1A | rs57786342 | A | G | 0.2336 | 0.1390 | 0.0283 | 8.91E-07 |
| MIP_1A | rs60198979 | A | G | 0.0788 | -0.2154 | 0.0455 | 2.22E-06 |
| MIP_1A | rs116615337 | A | G | 0.4232 | 0.1286 | 0.0278 | 3.66E-06 |
| MIP_1B | rs113877493 | T | C | 0.1645 | -0.6070 | 0.0217 | 3.67E-172 |
| MIP_1B | rs76582507 | A | G | 0.0156 | 0.3259 | 0.0676 | 1.42E-06 |
| MIP_1B | rs2411161 | T | C | 0.9508 | 0.1719 | 0.0365 | 2.55E-06 |
| MIP_1B | rs6908843 | A | G | 0.1684 | 0.0997 | 0.0209 | 1.78E-06 |
| MIP_1B | rs116237296 | A | G | 0.0054 | 0.5284 | 0.1115 | 2.15E-06 |
| MIP_1B | rs9793308 | A | G | 0.6036 | 0.0842 | 0.0177 | 2.04E-06 |
| MIP_1B | rs281748 | C | G | 0.7041 | -0.0794 | 0.0171 | 3.28E-06 |
| MIP_1B | rs72799710 | T | C | 0.1514 | -0.1037 | 0.0217 | 1.79E-06 |
| MIP_1B | rs117657747 | A | G | 0.0526 | 0.2089 | 0.0453 | 4.01E-06 |
| MIP_1B | rs113010081 | T | C | 0.8670 | -0.5799 | 0.0236 | 1.57E-133 |
| MIP_1B | rs76356863 | A | T | 0.9852 | -0.3456 | 0.0667 | 2.22E-07 |
| MIP_1B | rs5743614 | T | C | 0.1278 | 0.1115 | 0.0232 | 1.57E-06 |
| MIP_1B | rs1564708 | T | C | 0.2780 | -0.1697 | 0.0187 | 1.29E-19 |
| MIP_1B | rs141102180 | T | G | 0.0449 | 0.3298 | 0.0392 | 3.75E-17 |
| MIP_1B | rs3760440 | A | G | 0.6437 | 0.1242 | 0.0162 | 1.73E-14 |
| MIP_1B | rs79068918 | C | G | 0.0909 | 0.2674 | 0.0271 | 5.54E-23 |
| MIP_1B | rs17138331 | A | G | 0.9092 | -0.1434 | 0.0295 | 1.13E-06 |
| MIP_1B | rs1437220 | T | C | 0.9207 | 0.1437 | 0.0315 | 4.92E-06 |
| MIP_1B | rs117453826 | A | G | 0.9810 | -0.5907 | 0.0591 | 1.53E-23 |
| MIP_1B | rs72791296 | T | C | 0.0349 | 0.2364 | 0.0466 | 3.97E-07 |
| MIP_1B | rs76776296 | A | G | 0.9806 | 0.3130 | 0.0598 | 1.63E-07 |
| PDGF_BB | rs13412535 | A | G | 0.1892 | 0.3317 | 0.0214 | 2.89E-54 |
| PDGF_BB | rs73162807 | A | C | 0.0259 | -0.2313 | 0.0499 | 3.55E-06 |
| PDGF_BB | rs4965869 | T | C | 0.2446 | 0.1843 | 0.0181 | 2.22E-24 |
| PDGF_BB | rs11766649 | A | G | 0.8018 | 0.0902 | 0.0196 | 3.96E-06 |
| PDGF_BB | rs35859699 | A | G | 0.0109 | -0.3854 | 0.0838 | 4.22E-06 |
| PDGF_BB | rs116445074 | T | G | 0.0184 | 0.2869 | 0.0587 | 1.02E-06 |
| PDGF_BB | rs9941733 | A | G | 0.8364 | 0.1165 | 0.0227 | 3.02E-07 |
| PDGF_BB | rs13037046 | A | T | 0.1871 | -0.0948 | 0.0206 | 3.96E-06 |
| PDGF_BB | rs72777070 | T | G | 0.7854 | -0.1048 | 0.0200 | 1.56E-07 |
| PDGF_BB | rs55680718 | T | C | 0.1173 | -0.1359 | 0.0245 | 2.96E-08 |
| PDGF_BB | rs2324229 | T | C | 0.6134 | 0.0884 | 0.0161 | 4.02E-08 |
| PDGF_BB | rs12289510 | A | G | 0.4767 | -0.0772 | 0.0158 | 1.00E-06 |
| PDGF_BB | rs9936075 | A | G | 0.6436 | -0.0767 | 0.0163 | 2.68E-06 |
| PDGF_BB | rs11247305 | C | G | 0.9376 | -0.1687 | 0.0364 | 3.47E-06 |
| RANTES | rs147509526 | T | C | 0.0333 | -0.3558 | 0.0715 | 6.57E-07 |
| RANTES | rs62438851 | A | G | 0.8961 | -0.1904 | 0.0413 | 4.01E-06 |
| RANTES | rs9675798 | T | G | 0.9466 | -0.2583 | 0.0552 | 2.89E-06 |
| RANTES | rs72793342 | A | G | 0.2003 | -0.1505 | 0.0307 | 9.08E-07 |
| RANTES | rs112072646 | A | G | 0.0204 | 0.4209 | 0.0859 | 9.62E-07 |
| RANTES | rs2251660 | A | C | 0.8627 | 0.1831 | 0.0356 | 2.69E-07 |
| RANTES | rs2731672 | T | C | 0.2670 | -0.1242 | 0.0272 | 4.83E-06 |
| RANTES | rs7170339 | C | G | 0.0197 | -0.4283 | 0.0904 | 2.19E-06 |
| RANTES | rs7000423 | T | C | 0.6274 | -0.1314 | 0.0252 | 1.85E-07 |
| RANTES | rs74472919 | T | C | 0.0434 | 0.3547 | 0.0600 | 3.35E-09 |
| SCF | rs13412535 | A | G | 0.1892 | -0.1065 | 0.0213 | 5.59E-07 |
| SCF | rs113127926 | A | C | 0.0418 | 0.1974 | 0.0418 | 2.34E-06 |
| SCF | rs1568119 | T | C | 0.0051 | -0.5946 | 0.1129 | 1.37E-07 |
| SCF | rs72678285 | A | T | 0.8512 | 0.1062 | 0.0231 | 4.43E-06 |
| SCF | rs78666213 | T | G | 0.9797 | -0.2845 | 0.0574 | 7.15E-07 |
| SCF | rs78369473 | T | C | 0.0266 | -0.2256 | 0.0484 | 3.14E-06 |
| SCF | rs4841899 | T | C | 0.7421 | -0.1002 | 0.0178 | 1.67E-08 |
| SCF | rs1557570 | T | G | 0.2954 | 0.1172 | 0.0169 | 4.13E-12 |
| SCF | rs80271436 | A | G | 0.0263 | -0.2393 | 0.0484 | 7.49E-07 |
| SCF | rs635634 | T | C | 0.2060 | -0.1035 | 0.0191 | 5.71E-08 |
| SCF | rs7039247 | C | G | 0.6644 | 0.0790 | 0.0168 | 2.46E-06 |
| SCGF_B | rs17876031 | A | G | 0.3240 | -0.1496 | 0.0254 | 3.67E-09 |
| SCGF_B | rs77954165 | T | C | 0.9528 | 0.2631 | 0.0562 | 2.87E-06 |
| SCGF_B | rs12480722 | T | C | 0.8752 | 0.1654 | 0.0353 | 2.81E-06 |
| SCGF_B | rs78217154 | T | C | 0.9796 | 0.3942 | 0.0861 | 4.72E-06 |
| SCGF_B | rs116924815 | T | C | 0.0273 | 0.6046 | 0.0737 | 2.25E-16 |
| SCGF_B | rs13287050 | A | T | 0.7202 | -0.1210 | 0.0263 | 4.12E-06 |
| SCGF_B | rs4656185 | A | G | 0.2892 | 0.2103 | 0.0254 | 1.29E-16 |
| SCGF_B | rs149009264 | A | G | 0.9848 | 0.4551 | 0.0985 | 3.79E-06 |
| SCGF_B | rs143829871 | T | C | 0.9047 | -0.1866 | 0.0399 | 2.85E-06 |
| SCGF_B | rs7815967 | T | C | 0.1991 | 0.1325 | 0.0288 | 4.37E-06 |
| SCGF_B | rs264157 | A | G | 0.4743 | 0.1079 | 0.0233 | 3.69E-06 |
| SCGF_B | rs1149926 | T | C | 0.0239 | -0.3458 | 0.0749 | 3.92E-06 |
| SCGF_B | rs151194174 | A | G | 0.0187 | 0.4536 | 0.0941 | 1.45E-06 |
| SCGF_B | rs117716477 | A | C | 0.0199 | 0.8242 | 0.0840 | 1.03E-22 |
| SCGF_B | rs112346514 | T | C | 0.0302 | -0.3261 | 0.0703 | 3.54E-06 |
| SCGF_B | rs150733161 | T | C | 0.0142 | -0.5255 | 0.1120 | 2.69E-06 |
| SCGF_B | rs139413256 | A | G | 0.0139 | -0.5174 | 0.1076 | 1.53E-06 |
| SCGF_B | rs34911860 | A | G | 0.0291 | -0.3674 | 0.0787 | 3.00E-06 |
| SDF_1A | rs78037609 | A | G | 0.0104 | -0.6261 | 0.1334 | 2.67E-06 |
| SDF_1A | rs149893336 | A | G | 0.9930 | -0.4940 | 0.1082 | 4.93E-06 |
| SDF_1A | rs78883416 | C | G | 0.3080 | -0.0871 | 0.0182 | 1.76E-06 |
| SDF_1A | rs3988298 | T | C | 0.1042 | -0.1263 | 0.0266 | 2.12E-06 |
| SDF_1A | rs62194947 | T | C | 0.2632 | -0.0852 | 0.0185 | 4.27E-06 |
| SDF_1A | rs10474392 | A | G | 0.3340 | 0.0934 | 0.0177 | 1.38E-07 |
| SDF_1A | rs1600396 | A | G | 0.8096 | -0.0933 | 0.0204 | 4.94E-06 |
| SDF_1A | rs12141941 | T | C | 0.7493 | -0.0881 | 0.0186 | 2.26E-06 |
| SDF_1A | rs10013755 | A | T | 0.9929 | 0.5188 | 0.0995 | 1.85E-07 |
| TNF_A | rs10834997 | A | G | 0.6924 | -0.1230 | 0.0256 | 1.53E-06 |
| TNF_A | rs79105320 | A | G | 0.0112 | 0.5573 | 0.1177 | 2.21E-06 |
| TNF_A | rs111332265 | A | G | 0.9696 | -0.3678 | 0.0745 | 7.91E-07 |
| TNF_A | rs7256693 | T | C | 0.8925 | -0.1841 | 0.0400 | 4.11E-06 |
| TNF_A | rs115669577 | A | G | 0.0038 | 0.9810 | 0.1994 | 8.63E-07 |
| TNF_B | rs78296352 | T | G | 0.0252 | 1.2028 | 0.1366 | 1.28E-18 |
| TNF_B | rs75240021 | C | G | 0.0710 | 0.3713 | 0.0772 | 1.49E-06 |
| TNF_B | rs10925040 | T | C | 0.3710 | 0.1738 | 0.0372 | 2.93E-06 |
| TNF_B | rs753274 | T | C | 0.6085 | -0.1725 | 0.0370 | 3.14E-06 |
| TNF_B | rs7629875 | A | G | 0.9443 | 0.3841 | 0.0774 | 6.90E-07 |
| TRAIL | rs17434886 | T | C | 0.1949 | -0.0918 | 0.0199 | 4.20E-06 |
| TRAIL | rs28521641 | A | T | 0.9630 | -0.7004 | 0.0445 | 7.79E-56 |
| TRAIL | rs747324 | T | C | 0.2828 | -0.0826 | 0.0178 | 3.34E-06 |
| TRAIL | rs138987090 | A | G | 0.9857 | -0.7264 | 0.0749 | 2.97E-22 |
| TRAIL | rs74778900 | T | C | 0.0238 | 0.5791 | 0.0531 | 9.90E-28 |
| TRAIL | rs79287178 | A | G | 0.0389 | -0.4304 | 0.0420 | 1.17E-24 |
| TRAIL | rs72899452 | T | C | 0.0977 | 0.1223 | 0.0264 | 3.75E-06 |
| TRAIL | rs13278062 | T | G | 0.5589 | 0.0800 | 0.0157 | 3.33E-07 |
| TRAIL | rs28431810 | C | G | 0.8874 | -0.1216 | 0.0252 | 1.41E-06 |
| TRAIL | rs550057 | T | C | 0.3049 | -0.0783 | 0.0169 | 3.71E-06 |
| TRAIL | rs193112415 | T | C | 0.9809 | -1.0456 | 0.0620 | 1.01E-63 |
| TRAIL | rs57396456 | T | C | 0.9755 | -0.5641 | 0.0516 | 7.71E-28 |
| TRAIL | rs148051545 | T | C | 0.0114 | -0.4211 | 0.0843 | 5.84E-07 |
| TRAIL | rs62093514 | T | C | 0.0239 | 1.0459 | 0.0549 | 5.80E-81 |
| TRAIL | rs75928541 | A | G | 0.0188 | 0.2784 | 0.0591 | 2.44E-06 |
| TRAIL | rs73039026 | A | C | 0.9821 | -0.3098 | 0.0634 | 1.02E-06 |
| VEGF | rs10411345 | C | G | 0.7823 | -0.1041 | 0.0218 | 1.73E-06 |
| VEGF | rs12456390 | T | C | 0.6772 | -0.0818 | 0.0179 | 4.88E-06 |
| VEGF | rs8045833 | A | G | 0.2132 | 0.1030 | 0.0211 | 1.01E-06 |
| VEGF | rs7030781 | A | T | 0.5878 | 0.1403 | 0.0172 | 3.45E-16 |
| VEGF | rs6921438 | A | G | 0.4691 | -0.4866 | 0.0174 | 4.11E-172 |
| VEGF | rs10934631 | T | C | 0.8517 | -0.1132 | 0.0244 | 3.61E-06 |
| VEGF | rs9472183 | A | G | 0.4547 | -0.1264 | 0.0170 | 9.54E-14 |
| VEGF | rs10967186 | T | C | 0.5780 | 0.0899 | 0.0169 | 1.09E-07 |
| VEGF | rs4082730 | A | G | 0.0305 | 0.2455 | 0.0533 | 4.12E-06 |
| VEGF | rs13209117 | A | G | 0.2474 | 0.1253 | 0.0200 | 3.70E-10 |
| VEGF | rs73418461 | A | G | 0.0266 | -0.2498 | 0.0521 | 1.61E-06 |
| VEGF | rs73872715 | T | C | 0.0045 | -0.6079 | 0.1299 | 2.86E-06 |
| VEGF | rs3108686 | A | C | 0.0038 | -0.7967 | 0.1702 | 2.86E-06 |
| VEGF | rs10761731 | A | T | 0.6204 | -0.1146 | 0.0174 | 4.31E-11 |
| VEGF | rs143479231 | A | G | 0.0363 | -0.2628 | 0.0489 | 7.90E-08 |

IV, instrumental variable; MR, mendelian randomization; SNP, single nucleotide polymorphism; se, standard error

**Table S3** The MR results of causal effects of inflammatory cytokines in the IA, uIA, and SAH in MR analysis

| **outcome** | **exposure** | **method** | **nsnp** | **pval** | **OR(95%CI)** |
| --- | --- | --- | --- | --- | --- |
| IA | MCP_1_MCAF | IVW | 8 | 0.5705 | 0.96(0.85,1.10) |
| IA | MCP_1_MCAF | Maximum likelihood | 8 | 0.5733 | 0.96(0.84,1.10) |
| IA | MCP_1_MCAF | MR Egger | 8 | 0.9284 | 0.98(0.70,1.38) |
| IA | MCP_1_MCAF | Weighted median | 8 | 0.9911 | 1.00(0.85,1.18) |
| IA | MCP_1_MCAF | Simple mode | 8 | 0.7905 | 0.97(0.76,1.23) |
| IA | IL_6 | IVW | 4 | 0.1696 | 1.78(0.78,4.07) |
| IA | IL_6 | Maximum likelihood | 4 | 2.90E-05 | 2.26(1.54,3.30) |
| IA | IL_6 | MR Egger | 4 | 0.6986 | 0.66(0.11,4.01) |
| IA | IL_6 | Weighted median | 4 | 0.3321 | 1.20(0.83,1.73) |
| IA | IL_6 | Simple mode | 4 | 0.3589 | 1.26(0.83,1.90) |
| IA | IL_13 | IVW | 5 | 0.2638 | 1.10(0.93,1.32) |
| IA | IL_13 | Maximum likelihood | 5 | 0.0323 | 1.11(1.01,1.22) |
| IA | IL_13 | MR Egger | 5 | 0.7444 | 1.07(0.73,1.58) |
| IA | IL_13 | Weighted median | 5 | 0.0541 | 1.11(1.00,1.22) |
| IA | IL_13 | Simple mode | 5 | 0.8160 | 0.97(0.77,1.22) |
| IA | RANTES | IVW | 6 | 0.0119 | 0.86(0.76,0.97) |
| IA | RANTES | Maximum likelihood | 6 | 0.0412 | 0.85(0.74,0.99) |
| IA | RANTES | MR Egger | 6 | 0.4421 | 0.86(0.60,1.22) |
| IA | RANTES | Weighted median | 6 | 0.0310 | 0.81(0.67,0.98) |
| IA | RANTES | Simple mode | 6 | 0.1719 | 0.78(0.58,1.06) |
| IA | IL_1B | IVW | 2 | 0.9336 | 0.99(0.86,1.15) |
| IA | IL_1B | Maximum likelihood | 2 | 0.9590 | 0.99(0.78,1.26) |
| IA | SDF_1A | Wald ratio | 1 | 0.7581 | 1.10(0.60,2.01) |
| IA | IL_4 | IVW | 5 | 0.9737 | 1.00(0.78,1.29) |
| IA | IL_4 | Maximum likelihood | 5 | 0.9727 | 1.00(0.78,1.29) |
| IA | IL_4 | MR Egger | 5 | 0.4408 | 0.67(0.28,1.63) |
| IA | IL_4 | Weighted median | 5 | 0.6871 | 0.94(0.70,1.26) |
| IA | IL_4 | Simple mode | 5 | 0.7124 | 0.93(0.64,1.34) |
| IA | VEGF | IVW | 7 | 0.0018 | 1.08(1.03,1.14) |
| IA | VEGF | Maximum likelihood | 7 | 0.0563 | 1.08(1.00,1.18) |
| IA | VEGF | MR Egger | 7 | 0.1125 | 1.14(1.00,1.29) |
| IA | VEGF | Weighted median | 7 | 0.0491 | 1.09(1.00,1.19) |
| IA | VEGF | Simple mode | 7 | 0.7543 | 1.03(0.85,1.25) |
| IA | MIP_1A | IVW | 3 | 0.0049 | 0.84(0.75,0.95) |
| IA | MIP_1A | Maximum likelihood | 3 | 0.1294 | 0.84(0.68,1.05) |
| IA | MIP_1A | MR Egger | 3 | 0.7952 | 1.18(0.45,3.09) |
| IA | MIP_1A | Weighted median | 3 | 0.2917 | 0.87(0.66,1.13) |
| IA | MIP_1A | Simple mode | 3 | 0.4869 | 0.88(0.65,1.19) |
| IA | GROA | IVW | 6 | 0.7554 | 0.99(0.93,1.05) |
| IA | GROA | Maximum likelihood | 6 | 0.8009 | 0.99(0.92,1.07) |
| IA | GROA | MR Egger | 6 | 0.5510 | 1.06(0.89,1.27) |
| IA | GROA | Weighted median | 6 | 0.4260 | 0.96(0.88,1.05) |
| IA | GROA | Simple mode | 6 | 0.6135 | 0.97(0.85,1.10) |
| IA | IL_8 | IVW | 4 | 0.6246 | 1.04(0.88,1.24) |
| IA | IL_8 | Maximum likelihood | 4 | 0.5772 | 1.05(0.89,1.22) |
| IA | IL_8 | MR Egger | 4 | 0.7210 | 0.94(0.72,1.24) |
| IA | IL_8 | Weighted median | 4 | 0.9846 | 1.00(0.83,1.19) |
| IA | IL_8 | Simple mode | 4 | 0.8700 | 0.98(0.76,1.26) |
| IA | IFN_G | IVW | 3 | 0.5718 | 0.92(0.68,1.24) |
| IA | IFN_G | Maximum likelihood | 3 | 0.5892 | 0.91(0.66,1.27) |
| IA | IFN_G | MR Egger | 3 | 0.5191 | 1.40(0.70,2.82) |
| IA | IFN_G | Weighted median | 3 | 0.8528 | 1.04(0.70,1.54) |
| IA | IFN_G | Simple mode | 3 | 0.8587 | 1.05(0.65,1.71) |
| IA | FGF_BASIC | IVW | 2 | 1.10E-57 | 0.67(0.63,0.70) |
| IA | FGF_BASIC | Maximum likelihood | 2 | 0.0707 | 0.67(0.43,1.04) |
| IA | TNF_B | IVW | 2 | 5.52E-05 | 0.90(0.85,0.95) |
| IA | TNF_B | Maximum likelihood | 2 | 0.2276 | 0.90(0.75,1.07) |
| IA | MIP_1B | IVW | 3 | 0.4444 | 1.13(0.82,1.57) |
| IA | MIP_1B | Maximum likelihood | 3 | 0.2938 | 1.14(0.89,1.46) |
| IA | MIP_1B | MR Egger | 3 | 0.4440 | 0.68(0.36,1.28) |
| IA | MIP_1B | Weighted median | 3 | 0.2156 | 1.23(0.89,1.70) |
| IA | MIP_1B | Simple mode | 3 | 0.3818 | 1.30(0.82,2.07) |
| IA | SCGF_B | IVW | 5 | 0.2071 | 1.05(0.98,1.12) |
| IA | SCGF_B | Maximum likelihood | 5 | 0.5198 | 1.05(0.91,1.20) |
| IA | SCGF_B | MR Egger | 5 | 0.9577 | 1.02(0.57,1.82) |
| IA | SCGF_B | Weighted median | 5 | 0.4642 | 1.06(0.90,1.25) |
| IA | SCGF_B | Simple mode | 5 | 0.7345 | 0.96(0.77,1.20) |
| IA | CTACK | IVW | 6 | 0.9399 | 1.00(0.90,1.13) |
| IA | CTACK | Maximum likelihood | 6 | 0.9341 | 1.00(0.90,1.12) |
| IA | CTACK | MR Egger | 6 | 0.4942 | 0.93(0.77,1.12) |
| IA | CTACK | Weighted median | 6 | 0.6932 | 0.97(0.84,1.12) |
| IA | CTACK | Simple mode | 6 | 0.4297 | 0.90(0.71,1.14) |
| IA | HGF | IVW | 3 | 0.4659 | 0.93(0.78,1.12) |
| IA | HGF | Maximum likelihood | 3 | 0.4912 | 0.93(0.77,1.14) |
| IA | HGF | MR Egger | 3 | 0.5121 | 0.83(0.56,1.22) |
| IA | HGF | Weighted median | 3 | 0.5361 | 0.93(0.74,1.17) |
| IA | HGF | Simple mode | 3 | 0.7943 | 0.95(0.69,1.32) |
| IA | IL_5 | IVW | 4 | 0.3484 | 1.14(0.87,1.49) |
| IA | IL_5 | Maximum likelihood | 4 | 0.1222 | 1.15(0.96,1.37) |
| IA | IL_5 | MR Egger | 4 | 0.1712 | 0.50(0.26,0.96) |
| IA | IL_5 | Weighted median | 4 | 0.2400 | 1.14(0.92,1.42) |
| IA | IL_5 | Simple mode | 4 | 0.8099 | 0.94(0.61,1.45) |
| IA | IP_10 | IVW | 3 | 0.0047 | 0.88(0.80,0.96) |
| IA | IP_10 | Maximum likelihood | 3 | 0.1805 | 0.88(0.72,1.06) |
| IA | IP_10 | MR Egger | 3 | 0.6127 | 0.89(0.64,1.23) |
| IA | IP_10 | Weighted median | 3 | 0.2780 | 0.89(0.71,1.10) |
| IA | IP_10 | Simple mode | 3 | 0.5069 | 0.90(0.70,1.16) |
| IA | MIG | IVW | 3 | 0.0493 | 0.86(0.74,1.00) |
| IA | MIG | Maximum likelihood | 3 | 0.1630 | 0.86(0.69,1.06) |
| IA | MIG | MR Egger | 3 | 0.8026 | 1.16(0.46,2.94) |
| IA | MIG | Weighted median | 3 | 0.1320 | 0.82(0.64,1.06) |
| IA | MIG | Simple mode | 3 | 0.3235 | 0.81(0.59,1.11) |
| IA | EOTAXIN | IVW | 8 | 0.4234 | 0.95(0.84,1.08) |
| IA | EOTAXIN | Maximum likelihood | 8 | 0.4307 | 0.95(0.83,1.08) |
| IA | EOTAXIN | MR Egger | 8 | 0.3559 | 0.87(0.67,1.14) |
| IA | EOTAXIN | Weighted median | 8 | 0.6413 | 0.96(0.82,1.13) |
| IA | EOTAXIN | Simple mode | 8 | 0.5764 | 1.09(0.82,1.43) |
| IA | M_CSF | IVW | 2 | 0.9076 | 0.97(0.63,1.52) |
| IA | M_CSF | Maximum likelihood | 2 | 0.7984 | 0.97(0.78,1.21) |
| IA | IL_2RA | IVW | 4 | 0.6520 | 1.02(0.93,1.12) |
| IA | IL_2RA | Maximum likelihood | 4 | 0.7211 | 1.02(0.91,1.15) |
| IA | IL_2RA | MR Egger | 4 | 0.6131 | 0.95(0.79,1.13) |
| IA | IL_2RA | Weighted median | 4 | 0.9011 | 0.99(0.88,1.12) |
| IA | IL_2RA | Simple mode | 4 | 0.9230 | 1.01(0.81,1.26) |
| IA | SCF | IVW | 5 | 0.1097 | 1.10(0.98,1.24) |
| IA | SCF | Maximum likelihood | 5 | 0.3550 | 1.10(0.90,1.36) |
| IA | SCF | MR Egger | 5 | 0.5126 | 0.78(0.40,1.52) |
| IA | SCF | Weighted median | 5 | 0.4144 | 1.11(0.86,1.43) |
| IA | SCF | Simple mode | 5 | 0.4688 | 1.15(0.81,1.63) |
| IA | IL_9 | IVW | 2 | 0.1982 | 1.10(0.95,1.26) |
| IA | IL_9 | Maximum likelihood | 2 | 0.3909 | 1.10(0.89,1.35) |
| IA | MCP_3 | IVW | 2 | 4.73E-21 | 1.15(1.12,1.19) |
| IA | MCP_3 | Maximum likelihood | 2 | 0.0918 | 1.15(0.98,1.36) |
| IA | PDGF_BB | IVW | 8 | 0.3509 | 0.96(0.88,1.05) |
| IA | PDGF_BB | Maximum likelihood | 8 | 0.6182 | 0.96(0.81,1.13) |
| IA | PDGF_BB | MR Egger | 8 | 0.7085 | 0.92(0.59,1.42) |
| IA | PDGF_BB | Weighted median | 8 | 0.9257 | 0.99(0.80,1.22) |
| IA | PDGF_BB | Simple mode | 8 | 0.8504 | 1.03(0.75,1.41) |
| IA | IL_1RA | IVW | 3 | 0.0175 | 1.41(1.06,1.88) |
| IA | IL_1RA | Maximum likelihood | 3 | 0.0044 | 1.43(1.12,1.83) |
| IA | IL_1RA | MR Egger | 3 | 0.3779 | 0.16(0.01,1.81) |
| IA | IL_1RA | Weighted median | 3 | 0.0590 | 1.34(0.99,1.82) |
| IA | IL_1RA | Simple mode | 3 | 0.2737 | 1.35(0.91,2.00) |
| IA | IL_16 | IVW | 5 | 0.9459 | 1.00(0.92,1.10) |
| IA | IL_16 | Maximum likelihood | 5 | 0.9439 | 1.00(0.92,1.09) |
| IA | IL_16 | MR Egger | 5 | 0.3946 | 1.07(0.94,1.22) |
| IA | IL_16 | Weighted median | 5 | 0.5865 | 1.03(0.93,1.15) |
| IA | IL_16 | Simple mode | 5 | 0.7900 | 0.97(0.79,1.19) |
| IA | G_CSF | IVW | 2 | 0.1649 | 1.36(0.88,2.08) |
| IA | G_CSF | Maximum likelihood | 2 | 0.1063 | 1.36(0.94,1.99) |
| IA | IL_10 | IVW | 9 | 0.5675 | 1.05(0.88,1.25) |
| IA | IL_10 | Maximum likelihood | 9 | 0.3924 | 1.05(0.93,1.19) |
| IA | IL_10 | MR Egger | 9 | 0.1348 | 1.32(0.96,1.81) |
| IA | IL_10 | Weighted median | 9 | 0.0933 | 1.13(0.98,1.32) |
| IA | IL_10 | Simple mode | 9 | 0.6427 | 1.09(0.76,1.58) |
| IA | IL_18 | IVW | 6 | 0.0113 | 0.94(0.90,0.99) |
| IA | IL_18 | Maximum likelihood | 6 | 0.2246 | 0.94(0.85,1.04) |
| IA | IL_18 | MR Egger | 6 | 0.6605 | 0.95(0.78,1.17) |
| IA | IL_18 | Weighted median | 6 | 0.5728 | 0.97(0.86,1.09) |
| IA | IL_18 | Simple mode | 6 | 0.8026 | 0.98(0.83,1.16) |
| IA | TNF_A | Wald ratio | 1 | 0.3943 | 0.91(0.72,1.14) |
| IA | IL_7 | IVW | 4 | 0.2910 | 1.07(0.94,1.22) |
| IA | IL_7 | Maximum likelihood | 4 | 0.2419 | 1.07(0.95,1.21) |
| IA | IL_7 | MR Egger | 4 | 0.1748 | 1.38(1.02,1.88) |
| IA | IL_7 | Weighted median | 4 | 0.3157 | 1.07(0.94,1.22) |
| IA | IL_7 | Simple mode | 4 | 0.6923 | 0.93(0.67,1.29) |
| IA | IL_2 | IVW | 5 | 0.6049 | 1.04(0.90,1.21) |
| IA | IL_2 | Maximum likelihood | 5 | 0.5671 | 1.04(0.91,1.20) |
| IA | IL_2 | MR Egger | 5 | 0.7004 | 1.07(0.79,1.45) |
| IA | IL_2 | Weighted median | 5 | 0.7061 | 1.04(0.86,1.25) |
| IA | IL_2 | Simple mode | 5 | 0.3696 | 1.15(0.88,1.49) |
| IA | IL_12_P70 | IVW | 6 | 0.0012 | 1.11(1.04,1.19) |
| IA | IL_12_P70 | Maximum likelihood | 6 | 0.0498 | 1.11(1.00,1.24) |
| IA | IL_12_P70 | MR Egger | 6 | 0.1773 | 1.15(0.97,1.37) |
| IA | IL_12_P70 | Weighted median | 6 | 0.0361 | 1.12(1.01,1.25) |
| IA | IL_12_P70 | Simple mode | 6 | 0.7382 | 1.05(0.82,1.34) |
| IA | TRAIL | IVW | 8 | 0.0436 | 0.93(0.87,1.00) |
| IA | TRAIL | Maximum likelihood | 8 | 0.1735 | 0.93(0.84,1.03) |
| IA | TRAIL | MR Egger | 8 | 0.3312 | 0.93(0.82,1.06) |
| IA | TRAIL | Weighted median | 8 | 0.1183 | 0.91(0.81,1.02) |
| IA | TRAIL | Simple mode | 8 | 0.5743 | 0.94(0.77,1.15) |
| IA | IL_17 | IVW | 3 | 0.0028 | 0.82(0.72,0.93) |
| IA | IL_17 | Maximum likelihood | 3 | 0.1880 | 0.82(0.61,1.10) |
| IA | IL_17 | MR Egger | 3 | 0.8488 | 1.31(0.15,11.87) |
| IA | IL_17 | Weighted median | 3 | 0.3178 | 0.84(0.60,1.18) |
| IA | IL_17 | Simple mode | 3 | 0.6600 | 0.89(0.58,1.38) |
| IA | B_NGF | Wald ratio | 1 | 0.9582 | 1.01(0.66,1.55) |
| uIA | MCP_1_MCAF | IVW | 8 | 0.7046 | 0.96(0.77,1.19) |
| uIA | MCP_1_MCAF | Maximum likelihood | 8 | 0.7125 | 0.96(0.76,1.20) |
| uIA | MCP_1_MCAF | MR Egger | 8 | 0.7976 | 1.08(0.61,1.92) |
| uIA | MCP_1_MCAF | Weighted median | 8 | 0.5015 | 0.90(0.66,1.22) |
| uIA | MCP_1_MCAF | Simple mode | 8 | 0.3110 | 0.76(0.47,1.24) |
| uIA | IL_6 | IVW | 4 | 0.0249 | 2.02(1.09,3.72) |
| uIA | IL_6 | Maximum likelihood | 4 | 0.0051 | 2.11(1.25,3.56) |
| uIA | IL_6 | MR Egger | 4 | 0.9435 | 0.95(0.27,3.36) |
| uIA | IL_6 | Weighted median | 4 | 0.1510 | 1.61(0.84,3.11) |
| uIA | IL_6 | Simple mode | 4 | 0.5014 | 1.45(0.56,3.72) |
| uIA | IL_13 | IVW | 5 | 0.2762 | 1.09(0.94,1.26) |
| uIA | IL_13 | Maximum likelihood | 5 | 0.3208 | 1.09(0.92,1.28) |
| uIA | IL_13 | MR Egger | 5 | 0.9454 | 1.01(0.74,1.38) |
| uIA | IL_13 | Weighted median | 5 | 0.4760 | 1.06(0.90,1.26) |
| uIA | IL_13 | Simple mode | 5 | 0.8536 | 0.96(0.67,1.38) |
| uIA | RANTES | IVW | 6 | 0.9128 | 1.01(0.86,1.18) |
| uIA | RANTES | Maximum likelihood | 6 | 0.9478 | 1.01(0.77,1.32) |
| uIA | RANTES | MR Egger | 6 | 0.9044 | 1.04(0.55,1.97) |
| uIA | RANTES | Weighted median | 6 | 0.9898 | 1.00(0.72,1.39) |
| uIA | RANTES | Simple mode | 6 | 0.9729 | 1.01(0.63,1.60) |
| uIA | IL_1B | IVW | 2 | 0.0272 | 1.05(1.01,1.10) |
| uIA | IL_1B | Maximum likelihood | 2 | 0.8015 | 1.05(0.70,1.59) |
| uIA | SDF_1A | Wald ratio | 1 | 0.9280 | 1.05(0.37,2.98) |
| uIA | IL_4 | IVW | 5 | 0.5515 | 0.89(0.62,1.29) |
| uIA | IL_4 | Maximum likelihood | 5 | 0.6145 | 0.89(0.57,1.39) |
| uIA | IL_4 | MR Egger | 5 | 0.8012 | 0.81(0.17,3.73) |
| uIA | IL_4 | Weighted median | 5 | 0.9450 | 0.98(0.57,1.70) |
| uIA | IL_4 | Simple mode | 5 | 0.7675 | 1.14(0.50,2.60) |
| uIA | VEGF | IVW | 7 | 0.3798 | 1.06(0.93,1.22) |
| uIA | VEGF | Maximum likelihood | 7 | 0.4062 | 1.06(0.92,1.23) |
| uIA | VEGF | MR Egger | 7 | 0.8984 | 1.02(0.81,1.27) |
| uIA | VEGF | Weighted median | 7 | 0.5084 | 1.05(0.90,1.23) |
| uIA | VEGF | Simple mode | 7 | 0.7423 | 1.07(0.73,1.55) |
| uIA | MIP_1A | IVW | 3 | 0.0048 | 0.77(0.64,0.92) |
| uIA | MIP_1A | Maximum likelihood | 3 | 0.1976 | 0.77(0.52,1.15) |
| uIA | MIP_1A | MR Egger | 3 | 0.5747 | 0.49(0.08,2.86) |
| uIA | MIP_1A | Weighted median | 3 | 0.3464 | 0.79(0.49,1.29) |
| uIA | MIP_1A | Simple mode | 3 | 0.5416 | 0.82(0.47,1.41) |
| uIA | GROA | IVW | 6 | 0.2022 | 0.94(0.85,1.03) |
| uIA | GROA | Maximum likelihood | 6 | 0.3309 | 0.94(0.82,1.07) |
| uIA | GROA | MR Egger | 6 | 0.9973 | 1.00(0.74,1.36) |
| uIA | GROA | Weighted median | 6 | 0.6927 | 0.97(0.83,1.13) |
| uIA | GROA | Simple mode | 6 | 0.7519 | 0.96(0.77,1.20) |
| uIA | IL_8 | IVW | 4 | 0.3062 | 1.17(0.86,1.59) |
| uIA | IL_8 | Maximum likelihood | 4 | 0.2326 | 1.17(0.90,1.53) |
| uIA | IL_8 | MR Egger | 4 | 0.5643 | 1.22(0.69,2.17) |
| uIA | IL_8 | Weighted median | 4 | 0.3033 | 1.19(0.85,1.67) |
| uIA | IL_8 | Simple mode | 4 | 0.5769 | 1.17(0.72,1.89) |
| uIA | IFN_G | IVW | 3 | 1.44E-08 | 1.14(1.09,1.19) |
| uIA | IFN_G | Maximum likelihood | 3 | 0.6509 | 1.14(0.65,2.01) |
| uIA | IFN_G | MR Egger | 3 | 0.9135 | 1.09(0.32,3.76) |
| uIA | IFN_G | Weighted median | 3 | 0.6811 | 1.14(0.60,2.17) |
| uIA | IFN_G | Simple mode | 3 | 0.7590 | 1.15(0.53,2.52) |
| uIA | FGF_BASIC | IVW | 2 | 0.0063 | 0.59(0.40,0.86) |
| uIA | FGF_BASIC | Maximum likelihood | 2 | 0.1839 | 0.58(0.26,1.29) |
| uIA | TNF_B | IVW | 2 | 0.3567 | 0.84(0.58,1.22) |
| uIA | TNF_B | Maximum likelihood | 2 | 0.2707 | 0.83(0.60,1.15) |
| uIA | MIP_1B | IVW | 3 | 0.0607 | 1.42(0.98,2.06) |
| uIA | MIP_1B | Maximum likelihood | 3 | 0.1135 | 1.43(0.92,2.24) |
| uIA | MIP_1B | MR Egger | 3 | 0.7154 | 0.75(0.23,2.47) |
| uIA | MIP_1B | Weighted median | 3 | 0.0617 | 1.65(0.98,2.79) |
| uIA | MIP_1B | Simple mode | 3 | 0.2829 | 1.70(0.83,3.47) |
| uIA | SCGF_B | IVW | 5 | 0.2272 | 1.17(0.91,1.50) |
| uIA | SCGF_B | Maximum likelihood | 5 | 0.2068 | 1.17(0.92,1.50) |
| uIA | SCGF_B | MR Egger | 5 | 0.5813 | 1.48(0.43,5.13) |
| uIA | SCGF_B | Weighted median | 5 | 0.8443 | 1.03(0.75,1.42) |
| uIA | SCGF_B | Simple mode | 5 | 0.8334 | 1.06(0.63,1.79) |
| uIA | CTACK | IVW | 5 | 0.7579 | 1.03(0.86,1.24) |
| uIA | CTACK | Maximum likelihood | 5 | 0.7724 | 1.03(0.84,1.26) |
| uIA | CTACK | MR Egger | 5 | 0.6830 | 0.93(0.67,1.28) |
| uIA | CTACK | Weighted median | 5 | 0.4055 | 1.11(0.86,1.44) |
| uIA | CTACK | Simple mode | 5 | 0.5352 | 1.16(0.76,1.76) |
| uIA | HGF | IVW | 3 | 0.9832 | 1.00(0.73,1.38) |
| uIA | HGF | Maximum likelihood | 3 | 0.9844 | 1.00(0.71,1.42) |
| uIA | HGF | MR Egger | 3 | 0.5881 | 1.28(0.67,2.43) |
| uIA | HGF | Weighted median | 3 | 0.8954 | 1.03(0.71,1.49) |
| uIA | HGF | Simple mode | 3 | 0.8706 | 1.05(0.62,1.77) |
| uIA | IL_5 | IVW | 4 | 0.2399 | 1.21(0.88,1.65) |
| uIA | IL_5 | Maximum likelihood | 4 | 0.2120 | 1.21(0.89,1.65) |
| uIA | IL_5 | MR Egger | 4 | 0.2979 | 0.45(0.14,1.39) |
| uIA | IL_5 | Weighted median | 4 | 0.3843 | 1.19(0.81,1.74) |
| uIA | IL_5 | Simple mode | 4 | 0.5432 | 1.21(0.70,2.11) |
| uIA | IP_10 | IVW | 3 | 0.1290 | 0.78(0.57,1.07) |
| uIA | IP_10 | Maximum likelihood | 3 | 0.1794 | 0.78(0.54,1.12) |
| uIA | IP_10 | MR Egger | 3 | 0.6369 | 0.77(0.35,1.69) |
| uIA | IP_10 | Weighted median | 3 | 0.3343 | 0.79(0.50,1.27) |
| uIA | IP_10 | Simple mode | 3 | 0.3791 | 0.74(0.43,1.26) |
| uIA | MIG | IVW | 3 | 0.9679 | 1.00(0.81,1.24) |
| uIA | MIG | Maximum likelihood | 3 | 0.9820 | 1.00(0.69,1.46) |
| uIA | MIG | MR Egger | 3 | 0.5925 | 1.90(0.35,10.30) |
| uIA | MIG | Weighted median | 3 | 0.8750 | 0.97(0.63,1.48) |
| uIA | MIG | Simple mode | 3 | 0.8148 | 0.93(0.56,1.55) |
| uIA | EOTAXIN | IVW | 8 | 0.9886 | 1.00(0.84,1.20) |
| uIA | EOTAXIN | Maximum likelihood | 8 | 0.9913 | 1.00(0.79,1.27) |
| uIA | EOTAXIN | MR Egger | 8 | 0.9260 | 0.98(0.59,1.62) |
| uIA | EOTAXIN | Weighted median | 8 | 0.8832 | 0.98(0.72,1.32) |
| uIA | EOTAXIN | Simple mode | 8 | 0.7842 | 0.94(0.59,1.48) |
| uIA | M_CSF | IVW | 2 | 0.9316 | 0.98(0.68,1.43) |
| uIA | M_CSF | Maximum likelihood | 2 | 0.9302 | 0.98(0.67,1.43) |
| uIA | IL_2RA | IVW | 4 | 0.2389 | 1.13(0.92,1.38) |
| uIA | IL_2RA | Maximum likelihood | 4 | 0.2627 | 1.13(0.91,1.40) |
| uIA | IL_2RA | MR Egger | 4 | 0.6422 | 0.91(0.65,1.28) |
| uIA | IL_2RA | Weighted median | 4 | 0.3107 | 1.13(0.89,1.43) |
| uIA | IL_2RA | Simple mode | 4 | 0.2393 | 1.52(0.87,2.66) |
| uIA | SCF | IVW | 5 | 0.8723 | 1.03(0.71,1.49) |
| uIA | SCF | Maximum likelihood | 5 | 0.8705 | 1.03(0.71,1.50) |
| uIA | SCF | MR Egger | 5 | 0.4411 | 1.71(0.52,5.62) |
| uIA | SCF | Weighted median | 5 | 0.6542 | 0.90(0.58,1.41) |
| uIA | SCF | Simple mode | 5 | 0.6734 | 0.87(0.46,1.62) |
| uIA | IL_9 | IVW | 2 | 2.54E-07 | 1.24(1.14,1.35) |
| uIA | IL_9 | Maximum likelihood | 2 | 0.2522 | 1.24(0.86,1.79) |
| uIA | MCP_3 | IVW | 2 | 1.32E-43 | 0.87(0.85,0.88) |
| uIA | MCP_3 | Maximum likelihood | 2 | 0.3270 | 0.87(0.65,1.16) |
| uIA | PDGF_BB | IVW | 8 | 0.9787 | 1.00(0.78,1.28) |
| uIA | PDGF_BB | Maximum likelihood | 8 | 0.9818 | 1.00(0.74,1.34) |
| uIA | PDGF_BB | MR Egger | 8 | 0.4889 | 0.75(0.35,1.61) |
| uIA | PDGF_BB | Weighted median | 8 | 0.6338 | 1.10(0.75,1.60) |
| uIA | PDGF_BB | Simple mode | 8 | 0.8098 | 1.07(0.64,1.77) |
| uIA | IL_1RA | IVW | 3 | 0.2155 | 1.37(0.83,2.25) |
| uIA | IL_1RA | Maximum likelihood | 3 | 0.1338 | 1.39(0.90,2.12) |
| uIA | IL_1RA | MR Egger | 3 | 0.9465 | 0.73(0.00,1081.77) |
| uIA | IL_1RA | Weighted median | 3 | 0.1166 | 1.56(0.90,2.72) |
| uIA | IL_1RA | Simple mode | 3 | 0.2627 | 1.74(0.86,3.50) |
| uIA | IL_16 | IVW | 5 | 0.5568 | 0.97(0.89,1.06) |
| uIA | IL_16 | Maximum likelihood | 5 | 0.7407 | 0.97(0.84,1.14) |
| uIA | IL_16 | MR Egger | 5 | 0.8399 | 1.03(0.82,1.29) |
| uIA | IL_16 | Weighted median | 5 | 0.9391 | 0.99(0.83,1.19) |
| uIA | IL_16 | Simple mode | 5 | 0.9982 | 1.00(0.79,1.27) |
| uIA | G_CSF | IVW | 2 | 0.4206 | 1.33(0.66,2.66) |
| uIA | G_CSF | Maximum likelihood | 2 | 0.3844 | 1.34(0.69,2.58) |
| uIA | IL_10 | IVW | 9 | 0.6726 | 0.96(0.78,1.17) |
| uIA | IL_10 | Maximum likelihood | 9 | 0.6833 | 0.96(0.78,1.18) |
| uIA | IL_10 | MR Egger | 9 | 0.3072 | 1.27(0.83,1.92) |
| uIA | IL_10 | Weighted median | 9 | 0.5087 | 1.09(0.84,1.41) |
| uIA | IL_10 | Simple mode | 9 | 0.4347 | 0.78(0.44,1.41) |
| uIA | IL_18 | IVW | 6 | 0.4363 | 1.04(0.95,1.14) |
| uIA | IL_18 | Maximum likelihood | 6 | 0.6868 | 1.04(0.87,1.24) |
| uIA | IL_18 | MR Egger | 6 | 0.6976 | 1.08(0.74,1.58) |
| uIA | IL_18 | Weighted median | 6 | 0.6682 | 1.05(0.85,1.28) |
| uIA | IL_18 | Simple mode | 6 | 0.7712 | 1.04(0.80,1.36) |
| uIA | TNF_A | Wald ratio | 1 | 0.2616 | 0.81(0.55,1.18) |
| uIA | IL_7 | IVW | 4 | 0.0026 | 1.09(1.03,1.15) |
| uIA | IL_7 | Maximum likelihood | 4 | 0.4106 | 1.09(0.89,1.34) |
| uIA | IL_7 | MR Egger | 4 | 0.7678 | 1.10(0.64,1.87) |
| uIA | IL_7 | Weighted median | 4 | 0.4667 | 1.09(0.87,1.36) |
| uIA | IL_7 | Simple mode | 4 | 0.6770 | 1.08(0.77,1.51) |
| uIA | IL_2 | IVW | 5 | 0.9801 | 1.00(0.78,1.28) |
| uIA | IL_2 | Maximum likelihood | 5 | 0.9794 | 1.00(0.78,1.27) |
| uIA | IL_2 | MR Egger | 5 | 0.9776 | 1.01(0.62,1.65) |
| uIA | IL_2 | Weighted median | 5 | 0.8406 | 0.97(0.71,1.33) |
| uIA | IL_2 | Simple mode | 5 | 0.7861 | 1.07(0.69,1.66) |
| uIA | IL_12_P70 | IVW | 6 | 0.4295 | 1.09(0.88,1.35) |
| uIA | IL_12_P70 | Maximum likelihood | 6 | 0.3609 | 1.09(0.91,1.32) |
| uIA | IL_12_P70 | MR Egger | 6 | 0.8004 | 1.05(0.72,1.54) |
| uIA | IL_12_P70 | Weighted median | 6 | 0.4924 | 1.07(0.88,1.31) |
| uIA | IL_12_P70 | Simple mode | 6 | 0.9109 | 1.03(0.62,1.70) |
| uIA | TRAIL | IVW | 8 | 0.5867 | 1.05(0.88,1.26) |
| uIA | TRAIL | Maximum likelihood | 8 | 0.5931 | 1.05(0.87,1.27) |
| uIA | TRAIL | MR Egger | 8 | 0.2013 | 1.19(0.94,1.50) |
| uIA | TRAIL | Weighted median | 8 | 0.3220 | 1.11(0.90,1.37) |
| uIA | TRAIL | Simple mode | 8 | 0.4436 | 1.16(0.81,1.65) |
| uIA | IL_17 | IVW | 3 | 0.5588 | 0.87(0.55,1.38) |
| uIA | IL_17 | Maximum likelihood | 3 | 0.6159 | 0.87(0.50,1.50) |
| uIA | IL_17 | MR Egger | 3 | 0.7714 | 0.41(0.01,44.08) |
| uIA | IL_17 | Weighted median | 3 | 0.2788 | 0.69(0.35,1.35) |
| uIA | IL_17 | Simple mode | 3 | 0.5055 | 0.69(0.28,1.71) |
| uIA | B_NGF | Wald ratio | 1 | 0.7138 | 1.15(0.54,2.44) |
| SAH | MCP_1_MCAF | IVW | 8 | 0.4666 | 0.95(0.84,1.08) |
| SAH | MCP_1_MCAF | Maximum likelihood | 8 | 0.5501 | 0.95(0.82,1.11) |
| SAH | MCP_1_MCAF | MR Egger | 8 | 0.8727 | 0.97(0.66,1.41) |
| SAH | MCP_1_MCAF | Weighted median | 8 | 0.8181 | 0.98(0.81,1.18) |
| SAH | MCP_1_MCAF | Simple mode | 8 | 0.5226 | 0.89(0.65,1.24) |
| SAH | IL_6 | IVW | 4 | 0.2799 | 1.71(0.65,4.49) |
| SAH | IL_6 | Maximum likelihood | 4 | 0.0004 | 2.17(1.41,3.34) |
| SAH | IL_6 | MR Egger | 4 | 0.6392 | 0.55(0.06,4.75) |
| SAH | IL_6 | Weighted median | 4 | 0.4735 | 1.17(0.76,1.80) |
| SAH | IL_6 | Simple mode | 4 | 0.4816 | 1.21(0.76,1.92) |
| SAH | IL_13 | IVW | 5 | 0.2252 | 1.14(0.92,1.40) |
| SAH | IL_13 | Maximum likelihood | 5 | 0.0202 | 1.14(1.02,1.28) |
| SAH | IL_13 | MR Egger | 5 | 0.6366 | 1.13(0.72,1.79) |
| SAH | IL_13 | Weighted median | 5 | 0.0277 | 1.15(1.01,1.29) |
| SAH | IL_13 | Simple mode | 5 | 0.8442 | 0.97(0.71,1.32) |
| SAH | RANTES | IVW | 6 | 0.0017 | 0.81(0.70,0.92) |
| SAH | RANTES | Maximum likelihood | 6 | 0.0137 | 0.80(0.67,0.96) |
| SAH | RANTES | MR Egger | 6 | 0.4194 | 0.83(0.54,1.25) |
| SAH | RANTES | Weighted median | 6 | 0.0338 | 0.79(0.64,0.98) |
| SAH | RANTES | Simple mode | 6 | 0.1014 | 0.70(0.50,0.99) |
| SAH | IL_1B | IVW | 2 | 0.7298 | 0.97(0.81,1.16) |
| SAH | IL_1B | Maximum likelihood | 2 | 0.8293 | 0.97(0.73,1.29) |
| SAH | SDF_1A | Wald ratio | 1 | 0.8773 | 1.06(0.52,2.16) |
| SAH | IL_4 | IVW | 5 | 0.5477 | 1.11(0.80,1.54) |
| SAH | IL_4 | Maximum likelihood | 5 | 0.4766 | 1.11(0.83,1.49) |
| SAH | IL_4 | MR Egger | 5 | 0.7037 | 0.76(0.21,2.72) |
| SAH | IL_4 | Weighted median | 5 | 0.9387 | 1.01(0.70,1.47) |
| SAH | IL_4 | Simple mode | 5 | 0.9571 | 1.01(0.62,1.67) |
| SAH | VEGF | IVW | 7 | 0.0022 | 1.12(1.04,1.20) |
| SAH | VEGF | Maximum likelihood | 7 | 0.0283 | 1.12(1.01,1.23) |
| SAH | VEGF | MR Egger | 7 | 0.0772 | 1.19(1.02,1.39) |
| SAH | VEGF | Weighted median | 7 | 0.0162 | 1.14(1.02,1.26) |
| SAH | VEGF | Simple mode | 7 | 0.6354 | 0.93(0.71,1.22) |
| SAH | MIP_1A | IVW | 3 | 0.1454 | 0.88(0.73,1.05) |
| SAH | MIP_1A | Maximum likelihood | 3 | 0.3035 | 0.87(0.68,1.13) |
| SAH | MIP_1A | MR Egger | 3 | 0.6112 | 1.49(0.49,4.55) |
| SAH | MIP_1A | Weighted median | 3 | 0.4862 | 0.89(0.65,1.23) |
| SAH | MIP_1A | Simple mode | 3 | 0.6328 | 0.90(0.61,1.31) |
| SAH | GROA | IVW | 6 | 0.4875 | 1.02(0.96,1.10) |
| SAH | GROA | Maximum likelihood | 6 | 0.5927 | 1.02(0.94,1.12) |
| SAH | GROA | MR Egger | 6 | 0.4724 | 1.09(0.88,1.34) |
| SAH | GROA | Weighted median | 6 | 0.8919 | 1.01(0.91,1.12) |
| SAH | GROA | Simple mode | 6 | 0.9976 | 1.00(0.86,1.17) |
| SAH | IL_8 | IVW | 4 | 0.8362 | 1.02(0.85,1.23) |
| SAH | IL_8 | Maximum likelihood | 4 | 0.8294 | 1.02(0.85,1.23) |
| SAH | IL_8 | MR Egger | 4 | 0.4398 | 0.87(0.65,1.16) |
| SAH | IL_8 | Weighted median | 4 | 0.8251 | 0.98(0.79,1.20) |
| SAH | IL_8 | Simple mode | 4 | 0.7462 | 0.94(0.66,1.33) |
| SAH | IFN_G | IVW | 3 | 0.3626 | 0.83(0.55,1.25) |
| SAH | IFN_G | Maximum likelihood | 3 | 0.3158 | 0.82(0.56,1.21) |
| SAH | IFN_G | MR Egger | 3 | 0.5380 | 1.45(0.64,3.29) |
| SAH | IFN_G | Weighted median | 3 | 0.9265 | 0.98(0.60,1.60) |
| SAH | IFN_G | Simple mode | 3 | 0.9724 | 1.01(0.56,1.83) |
| SAH | FGF_BASIC | IVW | 2 | 0.0039 | 0.62(0.45,0.86) |
| SAH | FGF_BASIC | Maximum likelihood | 2 | 0.0648 | 0.62(0.37,1.03) |
| SAH | TNF_B | IVW | 2 | 0.3302 | 0.91(0.75,1.10) |
| SAH | TNF_B | Maximum likelihood | 2 | 0.3451 | 0.91(0.74,1.11) |
| SAH | MIP_1B | IVW | 3 | 0.9798 | 1.00(0.73,1.38) |
| SAH | MIP_1B | Maximum likelihood | 3 | 0.9764 | 1.00(0.76,1.33) |
| SAH | MIP_1B | MR Egger | 3 | 0.4284 | 0.63(0.30,1.30) |
| SAH | MIP_1B | Weighted median | 3 | 0.7669 | 1.05(0.75,1.46) |
| SAH | MIP_1B | Simple mode | 3 | 0.7674 | 1.10(0.65,1.86) |
| SAH | SCGF_B | IVW | 5 | 0.8574 | 1.01(0.89,1.16) |
| SAH | SCGF_B | Maximum likelihood | 5 | 0.8821 | 1.01(0.86,1.19) |
| SAH | SCGF_B | MR Egger | 5 | 0.6793 | 0.85(0.43,1.68) |
| SAH | SCGF_B | Weighted median | 5 | 0.8577 | 1.02(0.83,1.24) |
| SAH | SCGF_B | Simple mode | 5 | 0.4233 | 0.86(0.62,1.20) |
| SAH | CTACK | IVW | 6 | 0.7742 | 1.02(0.90,1.16) |
| SAH | CTACK | Maximum likelihood | 6 | 0.7646 | 1.02(0.90,1.15) |
| SAH | CTACK | MR Egger | 6 | 0.6444 | 0.95(0.76,1.18) |
| SAH | CTACK | Weighted median | 6 | 0.8146 | 1.02(0.88,1.18) |
| SAH | CTACK | Simple mode | 6 | 0.6315 | 0.95(0.77,1.17) |
| SAH | HGF | IVW | 3 | 0.6369 | 0.92(0.65,1.30) |
| SAH | HGF | Maximum likelihood | 3 | 0.4734 | 0.92(0.73,1.16) |
| SAH | HGF | MR Egger | 3 | 0.5432 | 0.73(0.35,1.49) |
| SAH | HGF | Weighted median | 3 | 0.5501 | 0.92(0.69,1.22) |
| SAH | HGF | Simple mode | 3 | 0.7151 | 0.92(0.64,1.34) |
| SAH | IL_5 | IVW | 4 | 0.4918 | 1.11(0.82,1.51) |
| SAH | IL_5 | Maximum likelihood | 4 | 0.2769 | 1.12(0.91,1.38) |
| SAH | IL_5 | MR Egger | 4 | 0.3337 | 0.53(0.20,1.42) |
| SAH | IL_5 | Weighted median | 4 | 0.0688 | 1.27(0.98,1.64) |
| SAH | IL_5 | Simple mode | 4 | 0.3796 | 1.35(0.76,2.38) |
| SAH | IP_10 | IVW | 3 | 4.78E-06 | 0.91(0.88,0.95) |
| SAH | IP_10 | Maximum likelihood | 3 | 0.4038 | 0.91(0.73,1.13) |
| SAH | IP_10 | MR Egger | 3 | 0.6877 | 0.90(0.63,1.31) |
| SAH | IP_10 | Weighted median | 3 | 0.4503 | 0.91(0.70,1.17) |
| SAH | IP_10 | Simple mode | 3 | 0.5424 | 0.90(0.67,1.20) |
| SAH | MIG | IVW | 3 | 5.18E-05 | 0.79(0.70,0.88) |
| SAH | MIG | Maximum likelihood | 3 | 0.0699 | 0.79(0.60,1.02) |
| SAH | MIG | MR Egger | 3 | 0.9849 | 0.99(0.33,2.95) |
| SAH | MIG | Weighted median | 3 | 0.0760 | 0.76(0.56,1.03) |
| SAH | MIG | Simple mode | 3 | 0.2771 | 0.75(0.52,1.10) |
| SAH | EOTAXIN | IVW | 8 | 0.5079 | 0.95(0.82,1.10) |
| SAH | EOTAXIN | Maximum likelihood | 8 | 0.5036 | 0.95(0.82,1.10) |
| SAH | EOTAXIN | MR Egger | 8 | 0.2151 | 0.81(0.60,1.09) |
| SAH | EOTAXIN | Weighted median | 8 | 0.6656 | 0.96(0.79,1.16) |
| SAH | EOTAXIN | Simple mode | 8 | 0.3735 | 1.18(0.84,1.67) |
| SAH | M_CSF | IVW | 2 | 0.7868 | 0.93(0.56,1.54) |
| SAH | M_CSF | Maximum likelihood | 2 | 0.5720 | 0.93(0.71,1.21) |
| SAH | IL_2RA | IVW | 4 | 0.9005 | 1.00(0.93,1.08) |
| SAH | IL_2RA | Maximum likelihood | 4 | 0.9453 | 1.00(0.88,1.15) |
| SAH | IL_2RA | MR Egger | 4 | 0.8122 | 0.97(0.79,1.20) |
| SAH | IL_2RA | Weighted median | 4 | 0.9233 | 0.99(0.86,1.14) |
| SAH | IL_2RA | Simple mode | 4 | 0.9307 | 0.99(0.80,1.22) |
| SAH | SCF | IVW | 5 | 0.1477 | 1.20(0.94,1.53) |
| SAH | SCF | Maximum likelihood | 5 | 0.1425 | 1.20(0.94,1.54) |
| SAH | SCF | MR Egger | 5 | 0.2816 | 0.59(0.27,1.30) |
| SAH | SCF | Weighted median | 5 | 0.0885 | 1.30(0.96,1.75) |
| SAH | SCF | Simple mode | 5 | 0.1756 | 1.43(0.93,2.20) |
| SAH | IL_9 | IVW | 2 | 0.4005 | 1.09(0.89,1.34) |
| SAH | IL_9 | Maximum likelihood | 2 | 0.4902 | 1.09(0.85,1.41) |
| SAH | MCP_3 | IVW | 2 | 1.12E-06 | 1.28(1.26,1.29) |
| SAH | MCP_3 | Maximum likelihood | 2 | 0.0159 | 1.28(1.05,1.56) |
| SAH | PDGF_BB | IVW | 8 | 0.1235 | 0.94(0.88,1.02) |
| SAH | PDGF_BB | Maximum likelihood | 8 | 0.5724 | 0.94(0.77,1.15) |
| SAH | PDGF_BB | MR Egger | 8 | 0.8932 | 0.96(0.58,1.61) |
| SAH | PDGF_BB | Weighted median | 8 | 0.7333 | 0.96(0.75,1.23) |
| SAH | PDGF_BB | Simple mode | 8 | 0.8194 | 0.96(0.67,1.37) |
| SAH | IL_1RA | IVW | 3 | 0.1342 | 1.36(0.91,2.04) |
| SAH | IL_1RA | Maximum likelihood | 3 | 0.0223 | 1.39(1.05,1.84) |
| SAH | IL_1RA | MR Egger | 3 | 0.3228 | 0.07(0.00,1.26) |
| SAH | IL_1RA | Weighted median | 3 | 0.0342 | 1.48(1.03,2.12) |
| SAH | IL_1RA | Simple mode | 3 | 0.1733 | 1.68(1.03,2.74) |
| SAH | IL_16 | IVW | 5 | 0.8916 | 1.01(0.88,1.15) |
| SAH | IL_16 | Maximum likelihood | 5 | 0.8578 | 1.01(0.91,1.12) |
| SAH | IL_16 | MR Egger | 5 | 0.5386 | 1.08(0.88,1.32) |
| SAH | IL_16 | Weighted median | 5 | 0.3711 | 1.06(0.93,1.21) |
| SAH | IL_16 | Simple mode | 5 | 0.6397 | 1.07(0.82,1.40) |
| SAH | G_CSF | IVW | 2 | 0.0854 | 1.35(0.96,1.89) |
| SAH | G_CSF | Maximum likelihood | 2 | 0.1823 | 1.35(0.87,2.09) |
| SAH | IL_10 | IVW | 9 | 0.3384 | 1.10(0.90,1.34) |
| SAH | IL_10 | Maximum likelihood | 9 | 0.1720 | 1.10(0.96,1.28) |
| SAH | IL_10 | MR Egger | 9 | 0.1158 | 1.41(0.97,2.04) |
| SAH | IL_10 | Weighted median | 9 | 0.0526 | 1.19(1.00,1.41) |
| SAH | IL_10 | Simple mode | 9 | 0.1231 | 0.65(0.40,1.06) |
| SAH | IL_18 | IVW | 6 | 0.0002 | 0.90(0.85,0.95) |
| SAH | IL_18 | Maximum likelihood | 6 | 0.0695 | 0.90(0.80,1.01) |
| SAH | IL_18 | MR Egger | 6 | 0.3996 | 0.89(0.71,1.13) |
| SAH | IL_18 | Weighted median | 6 | 0.2457 | 0.92(0.80,1.06) |
| SAH | IL_18 | Simple mode | 6 | 0.5485 | 0.94(0.78,1.13) |
| SAH | TNF_A | Wald ratio | 1 | 0.9074 | 1.02(0.77,1.34) |
| SAH | IL_7 | IVW | 4 | 0.4238 | 1.09(0.89,1.34) |
| SAH | IL_7 | Maximum likelihood | 4 | 0.2310 | 1.09(0.95,1.26) |
| SAH | IL_7 | MR Egger | 4 | 0.1299 | 1.59(1.10,2.28) |
| SAH | IL_7 | Weighted median | 4 | 0.1817 | 1.11(0.95,1.30) |
| SAH | IL_7 | Simple mode | 4 | 0.4051 | 0.80(0.50,1.26) |
| SAH | IL_2 | IVW | 5 | 0.4719 | 1.06(0.91,1.24) |
| SAH | IL_2 | Maximum likelihood | 5 | 0.4915 | 1.06(0.90,1.25) |
| SAH | IL_2 | MR Egger | 5 | 0.6014 | 1.10(0.80,1.52) |
| SAH | IL_2 | Weighted median | 5 | 0.4998 | 1.07(0.87,1.33) |
| SAH | IL_2 | Simple mode | 5 | 0.5920 | 1.08(0.83,1.41) |
| SAH | IL_12_P70 | IVW | 6 | 0.0015 | 1.15(1.06,1.26) |
| SAH | IL_12_P70 | Maximum likelihood | 6 | 0.0262 | 1.15(1.02,1.31) |
| SAH | IL_12_P70 | MR Egger | 6 | 0.1113 | 1.23(1.01,1.51) |
| SAH | IL_12_P70 | Weighted median | 6 | 0.0142 | 1.18(1.03,1.34) |
| SAH | IL_12_P70 | Simple mode | 6 | 0.3097 | 1.20(0.87,1.65) |
| SAH | TRAIL | IVW | 8 | 0.0071 | 0.90(0.83,0.97) |
| SAH | TRAIL | Maximum likelihood | 8 | 0.0927 | 0.90(0.80,1.02) |
| SAH | TRAIL | MR Egger | 8 | 0.1148 | 0.87(0.74,1.01) |
| SAH | TRAIL | Weighted median | 8 | 0.0693 | 0.88(0.76,1.01) |
| SAH | TRAIL | Simple mode | 8 | 0.4504 | 0.90(0.70,1.16) |
| SAH | IL_17 | IVW | 3 | 0.0295 | 0.77(0.60,0.97) |
| SAH | IL_17 | Maximum likelihood | 3 | 0.1257 | 0.76(0.54,1.08) |
| SAH | IL_17 | MR Egger | 3 | 0.6017 | 2.54(0.20,32.03) |
| SAH | IL_17 | Weighted median | 3 | 0.1064 | 0.72(0.49,1.07) |
| SAH | IL_17 | Simple mode | 3 | 0.2682 | 0.69(0.43,1.11) |
| SAH | B_NGF | Wald ratio | 1 | 0.7993 | 0.94(0.57,1.54) |

MR, mendelian randomization; IA, intracranial aneurysm; uIA, unruptured aneurysm; SAH, subarachnoid hemorrhage; CI, confidence interval; IVW, inverse variance weighting

**Table S4** The MR-Egger results of causal effects of inflammatory cytokines on the IA, uIA, SAH in MR analysis

| **outcome** | **exposure** | **F-statistics** | **Method** | **egger_intercept** | **pval** |
| --- | --- | --- | --- | --- | --- |
| IA | RANTES | 25.89 | MR-Egger | 0.0006 | 0.9865 |
| IA | VEGF | 133.75 | MR-Egger | -0.0150 | 0.4000 |
| IA | MIP_1A | 22.79 | MR-Egger | -0.0509 | 0.6131 |
| IA | FGF_BASIC | 22.96 | MR-Egger | NA | NA |
| IA | TNF_B | 23.23 | MR-Egger | NA | NA |
| IA | IP_10 | 21.66 | MR-Egger | -0.0041 | 0.9266 |
| IA | MCP_3 | 21.47 | MR-Egger | NA | NA |
| IA | IL_18 | 48.77 | MR-Egger | -0.0029 | 0.8986 |
| IA | IL_12_P70 | 112.55 | MR-Egger | -0.0083 | 0.6326 |
| IA | IL_17 | 28.51 | MR-Egger | -0.0557 | 0.7456 |
| uIA | MIP_1A | 22.79 | MR-Egger | 0.0679 | 0.6986 |
| uIA | IFN_G | 21.99 | MR-Egger | 0.0063 | 0.9494 |
| uIA | FGF_BASIC | 22.96 | MR-Egger | NA | NA |
| uIA | IL_9 | 24.24 | MR-Egger | NA | NA |
| uIA | MCP_3 | 21.47 | MR-Egger | NA | NA |
| uIA | IL_7 | 58.90 | MR-Egger | -0.0012 | 0.9856 |
| SAH | RANTES | 25.89 | MR-Egger | -0.0047 | 0.9096 |
| SAH | VEGF | 133.75 | MR-Egger | -0.0207 | 0.3332 |
| SAH | FGF_BASIC | 22.96 | MR-Egger | NA | NA |
| SAH | IP_10 | 21.66 | MR-Egger | 0.0021 | 0.9675 |
| SAH | MIG | 25.49 | MR-Egger | -0.0478 | 0.7467 |
| SAH | MCP_3 | 21.47 | MR-Egger | NA | NA |
| SAH | IL_18 | 48.77 | MR-Egger | 0.0013 | 0.9615 |
| SAH | IL_12_P70 | 112.55 | MR-Egger | -0.0156 | 0.4580 |

IA, intracranial aneurysm; uIA, unruptured intracranial aneurysm; SAH, subarachnoid hemorrhage; MR, mendelian randomization; NA, not available

**Table S5** The Cochran’s test results of causal effects of inflammatory cytokines on the IA, uIA, SAH in MR analysis

| **outcome** | **exposure** | **method** | **Q** | **Q_pval** |
| --- | --- | --- | --- | --- |
| **IA** | RANTES | MR Egger | 3.34 | 0.5027 |
|  | RANTES | Inverse variance weighted | 3.34 | 0.6477 |
|  | VEGF | MR Egger | 1.40 | 0.9243 |
|  | VEGF | Inverse variance weighted | 2.25 | 0.8958 |
|  | MIP_1A | MR Egger | 0.12 | 0.7333 |
|  | MIP_1A | Inverse variance weighted | 0.60 | 0.7409 |
|  | FGF_BASIC | Inverse variance weighted | 0.01 | 0.9068 |
|  | TNF_B | Inverse variance weighted | 0.09 | 0.7614 |
|  | IP_10 | MR Egger | 0.45 | 0.5027 |
|  | IP_10 | Inverse variance weighted | 0.46 | 0.7935 |
|  | MCP_3 | Inverse variance weighted | 0.03 | 0.8531 |
|  | IL_18 | MR Egger | 1.14 | 0.8879 |
|  | IL_18 | Inverse variance weighted | 1.16 | 0.9488 |
|  | IL_12_P70 | MR Egger | 1.57 | 0.8149 |
|  | IL_12_P70 | Inverse variance weighted | 1.83 | 0.8717 |
|  | IL_17 | MR Egger | 0.22 | 0.6418 |
|  | IL_17 | Inverse variance weighted | 0.39 | 0.8209 |
| **uIA** | MIP_1A | MR Egger | 0.16 | 0.6877 |
|  | MIP_1A | Inverse variance weighted | 0.42 | 0.8089 |
|  | IFN_G | MR Egger | 0.01 | 0.9361 |
|  | IFN_G | Inverse variance weighted | 0.01 | 0.9936 |
|  | FGF_BASIC | Inverse variance weighted | 0.25 | 0.6193 |
|  | IL_9 | Inverse variance weighted | 0.05 | 0.8212 |
|  | MCP_3 | Inverse variance weighted | 0.01 | 0.9429 |
|  | IL_7 | MR Egger | 0.22 | 0.8943 |
|  | IL_7 | Inverse variance weighted | 0.22 | 0.9737 |
| **SAH** | RANTES | MR Egger | 3.16 | 0.5309 |
|  | RANTES | Inverse variance weighted | 3.18 | 0.6726 |
|  | VEGF | MR Egger | 1.93 | 0.8594 |
|  | VEGF | Inverse variance weighted | 3.07 | 0.7997 |
|  | FGF_BASIC | Inverse variance weighted | 0.44 | 0.5092 |
|  | IP_10 | MR Egger | 0.06 | 0.7990 |
|  | IP_10 | Inverse variance weighted | 0.07 | 0.9669 |
|  | MIG | MR Egger | 0.24 | 0.6247 |
|  | MIG | Inverse variance weighted | 0.42 | 0.8122 |
|  | MCP_3 | Inverse variance weighted | 0.00 | 0.9553 |
|  | IL_18 | MR Egger | 1.18 | 0.8810 |
|  | IL_18 | Inverse variance weighted | 1.18 | 0.9463 |
|  | IL_12_P70 | MR Egger | 1.79 | 0.7750 |
|  | IL_12_P70 | Inverse variance weighted | 2.46 | 0.7826 |

IA, intracranial aneurysm; uIA, unruptured intracranial aneurysm; SAH, subarachnoid hemorrhage; MR, mendelian randomization

**Table S6** The MR results of causal effects of inflammatory cytokines on the IA, uIA and SAH in FinnGen in replicate MR analysis

| **outcome** | **exposure** | **method** | **nsnp** | **se** | **pval** | **OR(95%CI)** |
| --- | --- | --- | --- | --- | --- | --- |
| **IA** | MCP_1_MCAF | IVW | 13 | 0.0926 | 0.2819 | 0.91(0.75,1.09) |
|  | MCP_1_MCAF | MR Egger | 13 | 0.2946 | 0.5769 | 0.84(0.47,1.50) |
|  | MCP_1_MCAF | Weighted median | 13 | 0.1721 | 0.2475 | 0.82(0.58,1.15) |
|  | MCP_1_MCAF | Maximum likelihood | 13 | 0.1213 | 0.4069 | 0.90(0.71,1.15) |
|  | MCP_1_MCAF | Simple mode | 13 | 0.2815 | 0.7696 | 0.92(0.53,1.60) |
|  | IL_6 | IVW | 9 | 0.3790 | 0.4105 | 1.37(0.65,2.87) |
|  | IL_6 | MR Egger | 9 | 0.8274 | 0.3527 | 0.44(0.09,2.22) |
|  | IL_6 | Weighted median | 9 | 0.2714 | 0.9195 | 1.03(0.60,1.75) |
|  | IL_6 | Maximum likelihood | 9 | 0.2071 | 0.0781 | 1.44(0.96,2.16) |
|  | IL_6 | Simple mode | 9 | 0.3642 | 0.6842 | 1.17(0.57,2.38) |
|  | IL_13 | IVW | 10 | 0.0390 | 0.0001 | 0.86(0.80,0.93) |
|  | IL_13 | MR Egger | 10 | 0.1589 | 0.6467 | 0.93(0.68,1.27) |
|  | IL_13 | Weighted median | 10 | 0.0982 | 0.2842 | 0.90(0.74,1.09) |
|  | IL_13 | Maximum likelihood | 10 | 0.0835 | 0.0734 | 0.86(0.73,1.01) |
|  | IL_13 | Simple mode | 10 | 0.1718 | 0.5236 | 0.89(0.64,1.25) |
|  | RANTES | IVW | 9 | 0.0970 | 0.3073 | 1.10(0.91,1.34) |
|  | RANTES | MR Egger | 9 | 0.3374 | 0.6757 | 0.86(0.45,1.67) |
|  | RANTES | Weighted median | 9 | 0.1613 | 0.7378 | 1.06(0.77,1.45) |
|  | RANTES | Maximum likelihood | 9 | 0.1279 | 0.4263 | 1.11(0.86,1.42) |
|  | RANTES | Simple mode | 9 | 0.2573 | 0.9208 | 1.03(0.62,1.70) |
|  | IL_1B | IVW | 4 | 0.0321 | 1.84E-11 | 0.81(0.76,0.86) |
|  | IL_1B | MR Egger | 4 | 0.6188 | 0.8729 | 0.89(0.27,3.01) |
|  | IL_1B | Weighted median | 4 | 0.2500 | 0.4069 | 0.81(0.50,1.33) |
|  | IL_1B | Maximum likelihood | 4 | 0.2059 | 0.2938 | 0.81(0.54,1.21) |
|  | IL_1B | Simple mode | 4 | 0.2983 | 0.5515 | 0.82(0.46,1.47) |
|  | SDF_1A | IVW | 4 | 0.1592 | 0.4154 | 1.14(0.83,1.56) |
|  | SDF_1A | MR Egger | 4 | 0.5487 | 0.5048 | 1.56(0.53,4.56) |
|  | SDF_1A | Weighted median | 4 | 0.3338 | 0.5063 | 1.25(0.65,2.40) |
|  | SDF_1A | Maximum likelihood | 4 | 0.2773 | 0.6369 | 1.14(0.66,1.96) |
|  | SDF_1A | Simple mode | 4 | 0.4287 | 0.6337 | 1.25(0.54,2.91) |
|  | IL_4 | IVW | 13 | 0.1614 | 0.0102 | 1.51(1.10,2.08) |
|  | IL_4 | MR Egger | 13 | 0.3312 | 0.6302 | 1.18(0.62,2.26) |
|  | IL_4 | Weighted median | 13 | 0.2303 | 0.0376 | 1.61(1.03,2.53) |
|  | IL_4 | Maximum likelihood | 13 | 0.1687 | 0.0119 | 1.53(1.10,2.13) |
|  | IL_4 | Simple mode | 13 | 0.4005 | 0.1008 | 2.04(0.93,4.47) |
|  | MIF | IVW | 7 | 0.1501 | 0.1001 | 1.28(0.95,1.72) |
|  | MIF | MR Egger | 7 | 0.3168 | 0.5640 | 1.22(0.65,2.26) |
|  | MIF | Weighted median | 7 | 0.1989 | 0.8656 | 1.03(0.70,1.53) |
|  | MIF | Maximum likelihood | 7 | 0.1492 | 0.0858 | 1.29(0.96,1.73) |
|  | MIF | Simple mode | 7 | 0.2911 | 0.9063 | 1.04(0.59,1.83) |
|  | VEGF | IVW | 12 | 0.0634 | 0.1073 | 0.90(0.80,1.02) |
|  | VEGF | MR Egger | 12 | 0.1270 | 0.9013 | 0.98(0.77,1.26) |
|  | VEGF | Weighted median | 12 | 0.0897 | 0.3113 | 0.91(0.77,1.09) |
|  | VEGF | Maximum likelihood | 12 | 0.0804 | 0.1986 | 0.90(0.77,1.06) |
|  | VEGF | Simple mode | 12 | 0.2239 | 0.1943 | 0.73(0.47,1.14) |
|  | MIP_1A | IVW | 3 | 0.1531 | 0.0011 | 0.61(0.45,0.82) |
|  | MIP_1A | MR Egger | 3 | 0.5916 | 0.9947 | 1.00(0.31,3.17) |
|  | MIP_1A | Weighted median | 3 | 0.2829 | 0.2066 | 0.70(0.40,1.22) |
|  | MIP_1A | Maximum likelihood | 3 | 0.2382 | 0.0334 | 0.60(0.38,0.96) |
|  | MIP_1A | Simple mode | 3 | 0.3350 | 0.3984 | 0.70(0.36,1.35) |
|  | GROA | IVW | 8 | 0.1151 | 0.8034 | 0.97(0.78,1.22) |
|  | GROA | MR Egger | 8 | 0.2934 | 0.5798 | 0.84(0.47,1.50) |
|  | GROA | Weighted median | 8 | 0.1074 | 0.3536 | 0.91(0.73,1.12) |
|  | GROA | Maximum likelihood | 8 | 0.0792 | 0.7108 | 0.97(0.83,1.13) |
|  | GROA | Simple mode | 8 | 0.1794 | 0.7179 | 0.93(0.66,1.33) |
|  | IL_8 | IVW | 8 | 0.1400 | 0.8231 | 0.97(0.74,1.28) |
|  | IL_8 | MR Egger | 8 | 0.2677 | 0.5592 | 1.18(0.70,1.99) |
|  | IL_8 | Weighted median | 8 | 0.1782 | 0.7017 | 1.07(0.76,1.52) |
|  | IL_8 | Maximum likelihood | 8 | 0.1428 | 0.8192 | 0.97(0.73,1.28) |
|  | IL_8 | Simple mode | 8 | 0.2872 | 0.7188 | 1.11(0.63,1.96) |
|  | IFN_G | IVW | 8 | 0.2711 | 0.5204 | 1.19(0.70,2.02) |
|  | IFN_G | MR Egger | 8 | 0.5142 | 0.5973 | 0.75(0.27,2.06) |
|  | IFN_G | Weighted median | 8 | 0.3034 | 0.5042 | 1.22(0.68,2.22) |
|  | IFN_G | Maximum likelihood | 8 | 0.2071 | 0.3567 | 1.21(0.81,1.82) |
|  | IFN_G | Simple mode | 8 | 0.5181 | 0.8837 | 1.08(0.39,2.99) |
|  | FGF_BASIC | IVW | 5 | 0.2628 | 0.4790 | 0.83(0.50,1.39) |
|  | FGF_BASIC | MR Egger | 5 | 0.6648 | 0.7778 | 1.23(0.33,4.52) |
|  | FGF_BASIC | Weighted median | 5 | 0.3033 | 0.4281 | 0.79(0.43,1.42) |
|  | FGF_BASIC | Maximum likelihood | 5 | 0.2431 | 0.4232 | 0.82(0.51,1.33) |
|  | FGF_BASIC | Simple mode | 5 | 0.5088 | 0.3285 | 0.57(0.21,1.54) |
|  | TNF_B | IVW | 4 | 0.0734 | 0.6507 | 1.03(0.90,1.19) |
|  | TNF_B | MR Egger | 4 | 0.1829 | 0.8868 | 1.03(0.72,1.47) |
|  | TNF_B | Weighted median | 4 | 0.1290 | 0.5674 | 1.08(0.84,1.39) |
|  | TNF_B | Maximum likelihood | 4 | 0.1126 | 0.7657 | 1.03(0.83,1.29) |
|  | TNF_B | Simple mode | 4 | 0.1902 | 0.6507 | 1.10(0.76,1.60) |
|  | MIP_1B | IVW | 17 | 0.0768 | 0.5933 | 0.96(0.83,1.12) |
|  | MIP_1B | MR Egger | 17 | 0.1125 | 0.0543 | 0.79(0.63,0.99) |
|  | MIP_1B | Weighted median | 17 | 0.0926 | 0.5410 | 0.94(0.79,1.13) |
|  | MIP_1B | Maximum likelihood | 17 | 0.0650 | 0.5242 | 0.96(0.84,1.09) |
|  | MIP_1B | Simple mode | 17 | 0.1553 | 0.8617 | 0.97(0.72,1.32) |
|  | SCGF_B | IVW | 17 | 0.0923 | 0.4584 | 1.07(0.89,1.28) |
|  | SCGF_B | MR Egger | 17 | 0.1692 | 0.5234 | 0.90(0.64,1.25) |
|  | SCGF_B | Weighted median | 17 | 0.1111 | 0.8795 | 1.02(0.82,1.26) |
|  | SCGF_B | Maximum likelihood | 17 | 0.0819 | 0.3826 | 1.07(0.91,1.26) |
|  | SCGF_B | Simple mode | 17 | 0.1998 | 0.4088 | 1.18(0.80,1.75) |
|  | CTACK | IVW | 13 | 0.0824 | 0.1782 | 1.12(0.95,1.31) |
|  | CTACK | MR Egger | 13 | 0.1750 | 0.8557 | 0.97(0.69,1.36) |
|  | CTACK | Weighted median | 13 | 0.1324 | 0.2155 | 1.18(0.91,1.53) |
|  | CTACK | Maximum likelihood | 13 | 0.0950 | 0.2323 | 1.12(0.93,1.35) |
|  | CTACK | Simple mode | 13 | 0.2205 | 0.2691 | 1.29(0.84,1.99) |
|  | HGF | IVW | 6 | 0.1861 | 0.5850 | 0.90(0.63,1.30) |
|  | HGF | MR Egger | 6 | 0.4896 | 0.7737 | 0.86(0.33,2.25) |
|  | HGF | Weighted median | 6 | 0.2655 | 0.8814 | 1.04(0.62,1.75) |
|  | HGF | Maximum likelihood | 6 | 0.2137 | 0.6267 | 0.90(0.59,1.37) |
|  | HGF | Simple mode | 6 | 0.3923 | 0.6715 | 1.19(0.55,2.57) |
|  | IL_5 | IVW | 8 | 0.1101 | 0.5268 | 0.93(0.75,1.16) |
|  | IL_5 | MR Egger | 8 | 0.2925 | 0.5734 | 1.19(0.67,2.11) |
|  | IL_5 | Weighted median | 8 | 0.1644 | 0.7433 | 0.95(0.69,1.31) |
|  | IL_5 | Maximum likelihood | 8 | 0.1288 | 0.5885 | 0.93(0.72,1.20) |
|  | IL_5 | Simple mode | 8 | 0.2590 | 0.8217 | 0.94(0.57,1.56) |
|  | IP_10 | IVW | 9 | 0.1178 | 0.0001 | 0.63(0.50,0.80) |
|  | IP_10 | MR Egger | 9 | 0.2653 | 0.0905 | 0.59(0.35,1.00) |
|  | IP_10 | Weighted median | 9 | 0.1707 | 0.0033 | 0.61(0.43,0.85) |
|  | IP_10 | Maximum likelihood | 9 | 0.1341 | 0.0006 | 0.63(0.48,0.82) |
|  | IP_10 | Simple mode | 9 | 0.2342 | 0.0565 | 0.59(0.38,0.94) |
|  | MIG | IVW | 10 | 0.1175 | 0.4611 | 0.92(0.73,1.15) |
|  | MIG | MR Egger | 10 | 0.2307 | 0.0743 | 0.62(0.40,0.98) |
|  | MIG | Weighted median | 10 | 0.1632 | 0.8767 | 1.03(0.74,1.41) |
|  | MIG | Maximum likelihood | 10 | 0.1199 | 0.4575 | 0.91(0.72,1.16) |
|  | MIG | Simple mode | 10 | 0.2657 | 0.5446 | 1.18(0.70,1.99) |
|  | EOTAXIN | IVW | 13 | 0.1553 | 0.9706 | 0.99(0.73,1.35) |
|  | EOTAXIN | MR Egger | 13 | 0.3344 | 0.5163 | 1.25(0.65,2.41) |
|  | EOTAXIN | Weighted median | 13 | 0.1760 | 0.6303 | 1.09(0.77,1.54) |
|  | EOTAXIN | Maximum likelihood | 13 | 0.1207 | 0.9608 | 0.99(0.78,1.26) |
|  | EOTAXIN | Simple mode | 13 | 0.3921 | 0.4873 | 1.32(0.61,2.86) |
|  | M_CSF | IVW | 9 | 0.0746 | 0.9704 | 1.00(0.86,1.15) |
|  | M_CSF | MR Egger | 9 | 0.1993 | 0.7634 | 1.06(0.72,1.57) |
|  | M_CSF | Weighted median | 9 | 0.1361 | 0.8772 | 1.02(0.78,1.33) |
|  | M_CSF | Maximum likelihood | 9 | 0.1029 | 0.9783 | 1.00(0.82,1.22) |
|  | M_CSF | Simple mode | 9 | 0.2052 | 0.4748 | 1.17(0.78,1.74) |
|  | IL_2RA | IVW | 6 | 0.1552 | 0.3420 | 0.86(0.64,1.17) |
|  | IL_2RA | MR Egger | 6 | 0.2501 | 0.3456 | 0.77(0.47,1.25) |
|  | IL_2RA | Weighted median | 6 | 0.1371 | 0.1753 | 0.83(0.63,1.09) |
|  | IL_2RA | Maximum likelihood | 6 | 0.1158 | 0.1874 | 0.86(0.68,1.08) |
|  | IL_2RA | Simple mode | 6 | 0.2771 | 0.3436 | 0.75(0.43,1.29) |
|  | SCF | IVW | 9 | 0.2043 | 0.2410 | 1.27(0.85,1.90) |
|  | SCF | MR Egger | 9 | 0.5302 | 0.4501 | 1.53(0.54,4.32) |
|  | SCF | Weighted median | 9 | 0.2386 | 0.4404 | 1.20(0.75,1.92) |
|  | SCF | Maximum likelihood | 9 | 0.1857 | 0.1750 | 1.29(0.89,1.85) |
|  | SCF | Simple mode | 9 | 0.4550 | 0.1508 | 2.06(0.84,5.03) |
|  | IL_9 | IVW | 4 | 0.1705 | 0.3910 | 0.86(0.62,1.21) |
|  | IL_9 | MR Egger | 4 | 1.1284 | 0.5946 | 2.03(0.22,18.53) |
|  | IL_9 | Weighted median | 4 | 0.2728 | 0.2739 | 0.74(0.43,1.27) |
|  | IL_9 | Maximum likelihood | 4 | 0.2254 | 0.5063 | 0.86(0.55,1.34) |
|  | IL_9 | Simple mode | 4 | 0.3583 | 0.3951 | 0.70(0.35,1.42) |
|  | MCP_3 | IVW | 5 | 0.0884 | 0.7345 | 0.97(0.82,1.15) |
|  | MCP_3 | MR Egger | 5 | 0.3240 | 0.8767 | 1.06(0.56,1.99) |
|  | MCP_3 | Weighted median | 5 | 0.1279 | 0.9193 | 1.01(0.79,1.30) |
|  | MCP_3 | Maximum likelihood | 5 | 0.1000 | 0.7574 | 0.97(0.80,1.18) |
|  | MCP_3 | Simple mode | 5 | 0.1720 | 0.9610 | 1.01(0.72,1.41) |
|  | PDGF_BB | IVW | 12 | 0.0942 | 0.0142 | 1.26(1.05,1.52) |
|  | PDGF_BB | MR Egger | 12 | 0.2245 | 0.6483 | 1.11(0.72,1.73) |
|  | PDGF_BB | Weighted median | 12 | 0.1436 | 0.0630 | 1.31(0.99,1.73) |
|  | PDGF_BB | Maximum likelihood | 12 | 0.1165 | 0.0434 | 1.27(1.01,1.59) |
|  | PDGF_BB | Simple mode | 12 | 0.2117 | 0.9184 | 1.02(0.68,1.55) |
|  | IL_1RA | IVW | 6 | 0.1336 | 0.0660 | 1.28(0.98,1.66) |
|  | IL_1RA | MR Egger | 6 | 0.4493 | 0.7877 | 0.88(0.36,2.12) |
|  | IL_1RA | Weighted median | 6 | 0.2083 | 0.1982 | 1.31(0.87,1.97) |
|  | IL_1RA | Maximum likelihood | 6 | 0.1712 | 0.1416 | 1.29(0.92,1.80) |
|  | IL_1RA | Simple mode | 6 | 0.3077 | 0.2999 | 1.43(0.78,2.61) |
|  | IL_16 | IVW | 9 | 0.1340 | 0.3743 | 0.89(0.68,1.15) |
|  | IL_16 | MR Egger | 9 | 0.2110 | 0.1075 | 0.68(0.45,1.02) |
|  | IL_16 | Weighted median | 9 | 0.1345 | 0.4363 | 0.90(0.69,1.17) |
|  | IL_16 | Maximum likelihood | 9 | 0.0987 | 0.2113 | 0.88(0.73,1.07) |
|  | IL_16 | Simple mode | 9 | 0.2209 | 0.4438 | 0.84(0.54,1.29) |
|  | G_CSF | IVW | 8 | 0.2137 | 0.9679 | 1.01(0.66,1.53) |
|  | G_CSF | MR Egger | 8 | 0.4143 | 0.7337 | 0.86(0.38,1.94) |
|  | G_CSF | Weighted median | 8 | 0.2667 | 0.2617 | 1.35(0.80,2.28) |
|  | G_CSF | Maximum likelihood | 8 | 0.1934 | 0.9639 | 1.01(0.69,1.47) |
|  | G_CSF | Simple mode | 8 | 0.3705 | 0.3213 | 1.48(0.72,3.07) |
|  | IL_10 | IVW | 12 | 0.1174 | 0.0144 | 0.75(0.60,0.94) |
|  | IL_10 | MR Egger | 12 | 0.2452 | 0.9298 | 0.98(0.60,1.58) |
|  | IL_10 | Weighted median | 12 | 0.1423 | 0.3275 | 0.87(0.66,1.15) |
|  | IL_10 | Maximum likelihood | 12 | 0.1198 | 0.0147 | 0.75(0.59,0.94) |
|  | IL_10 | Simple mode | 12 | 0.2840 | 0.7356 | 0.91(0.52,1.58) |
|  | IL_18 | IVW | 11 | 0.1409 | 0.6172 | 0.93(0.71,1.23) |
|  | IL_18 | MR Egger | 11 | 0.3437 | 0.6415 | 0.85(0.43,1.66) |
|  | IL_18 | Weighted median | 11 | 0.1475 | 0.8082 | 0.96(0.72,1.29) |
|  | IL_18 | Maximum likelihood | 11 | 0.0918 | 0.4309 | 0.93(0.78,1.11) |
|  | IL_18 | Simple mode | 11 | 0.2948 | 0.8607 | 1.05(0.59,1.88) |
|  | TNF_A | IVW | 5 | 0.1322 | 0.1885 | 1.19(0.92,1.54) |
|  | TNF_A | MR Egger | 5 | 0.2968 | 0.6186 | 0.85(0.47,1.52) |
|  | TNF_A | Weighted median | 5 | 0.2219 | 0.2684 | 1.28(0.83,1.97) |
|  | TNF_A | Maximum likelihood | 5 | 0.1720 | 0.3020 | 1.19(0.85,1.67) |
|  | TNF_A | Simple mode | 5 | 0.3096 | 0.3855 | 1.35(0.74,2.48) |
|  | IL_7 | IVW | 9 | 0.1136 | 0.7525 | 0.96(0.77,1.21) |
|  | IL_7 | MR Egger | 9 | 0.2650 | 0.6230 | 1.15(0.68,1.93) |
|  | IL_7 | Weighted median | 9 | 0.1237 | 0.3696 | 0.89(0.70,1.14) |
|  | IL_7 | Maximum likelihood | 9 | 0.0975 | 0.6993 | 0.96(0.80,1.17) |
|  | IL_7 | Simple mode | 9 | 0.2014 | 0.6071 | 0.90(0.60,1.33) |
|  | IL_2 | IVW | 7 | 0.1407 | 0.9629 | 1.01(0.76,1.33) |
|  | IL_2 | MR Egger | 7 | 0.2866 | 0.5490 | 1.20(0.69,2.11) |
|  | IL_2 | Weighted median | 7 | 0.1803 | 0.8712 | 0.97(0.68,1.38) |
|  | IL_2 | Maximum likelihood | 7 | 0.1358 | 0.9615 | 1.01(0.77,1.31) |
|  | IL_2 | Simple mode | 7 | 0.2712 | 0.5787 | 0.85(0.50,1.45) |
|  | IL_12_P70 | IVW | 12 | 0.0597 | 0.0122 | 0.86(0.77,0.97) |
|  | IL_12_P70 | MR Egger | 12 | 0.1703 | 0.7968 | 0.96(0.68,1.33) |
|  | IL_12_P70 | Weighted median | 12 | 0.1095 | 0.3455 | 0.90(0.73,1.12) |
|  | IL_12_P70 | Maximum likelihood | 12 | 0.0990 | 0.1331 | 0.86(0.71,1.05) |
|  | IL_12_P70 | Simple mode | 12 | 0.2311 | 0.9123 | 0.97(0.62,1.53) |
|  | TRAIL | IVW | 11 | 0.1063 | 0.6659 | 0.96(0.78,1.18) |
|  | TRAIL | MR Egger | 11 | 0.1406 | 0.9086 | 0.98(0.75,1.30) |
|  | TRAIL | Weighted median | 11 | 0.1141 | 0.3999 | 0.91(0.73,1.14) |
|  | TRAIL | Maximum likelihood | 11 | 0.0855 | 0.5873 | 0.95(0.81,1.13) |
|  | TRAIL | Simple mode | 11 | 0.2157 | 0.4692 | 0.85(0.56,1.30) |
|  | IL_17 | IVW | 7 | 0.2026 | 0.8914 | 0.97(0.65,1.45) |
|  | IL_17 | MR Egger | 7 | 0.4154 | 0.6626 | 0.82(0.37,1.86) |
|  | IL_17 | Weighted median | 7 | 0.2281 | 0.9514 | 1.01(0.65,1.59) |
|  | IL_17 | Maximum likelihood | 7 | 0.1812 | 0.8752 | 0.97(0.68,1.39) |
|  | IL_17 | Simple mode | 7 | 0.3333 | 0.5505 | 1.23(0.64,2.37) |
|  | B_NGF | IVW | 4 | 0.1690 | 0.3405 | 0.85(0.61,1.19) |
|  | B_NGF | MR Egger | 4 | 1.0338 | 0.3681 | 0.30(0.04,2.30) |
|  | B_NGF | Weighted median | 4 | 0.2232 | 0.2789 | 0.79(0.51,1.22) |
|  | B_NGF | Maximum likelihood | 4 | 0.1881 | 0.3796 | 0.85(0.59,1.23) |
|  | B_NGF | Simple mode | 4 | 0.2986 | 0.4357 | 0.76(0.43,1.37) |
| **uIA** | MCP_1_MCAF | IVW | 13 | 0.0729 | 0.7844 | 1.02(0.88,1.18) |
|  | MCP_1_MCAF | MR Egger | 13 | 0.2342 | 0.6736 | 1.11(0.70,1.75) |
|  | MCP_1_MCAF | Weighted median | 13 | 0.1253 | 0.6657 | 0.95(0.74,1.21) |
|  | MCP_1_MCAF | Maximum likelihood | 13 | 0.0964 | 0.8341 | 1.02(0.84,1.23) |
|  | MCP_1_MCAF | Simple mode | 13 | 0.2396 | 0.7444 | 0.92(0.58,1.48) |
|  | IL_6 | IVW | 9 | 0.3091 | 0.5208 | 1.22(0.67,9.24) |
|  | IL_6 | MR Egger | 9 | 0.7638 | 0.8503 | 0.86(0.19,9.85) |
|  | IL_6 | Weighted median | 9 | 0.2131 | 0.2242 | 0.77(0.51,9.17) |
|  | IL_6 | Maximum likelihood | 9 | 0.1643 | 0.1568 | 1.26(0.91,9.74) |
|  | IL_6 | Simple mode | 9 | 0.2803 | 0.2043 | 0.68(0.39,9.18) |
|  | IL_13 | IVW | 10 | 0.0588 | 0.0566 | 0.89(0.80,1.00) |
|  | IL_13 | MR Egger | 10 | 0.1263 | 0.2553 | 0.86(0.67,1.10) |
|  | IL_13 | Weighted median | 10 | 0.0805 | 0.3799 | 0.93(0.80,1.09) |
|  | IL_13 | Maximum likelihood | 10 | 0.0668 | 0.0882 | 0.89(0.78,1.02) |
|  | IL_13 | Simple mode | 10 | 0.1445 | 0.8658 | 0.98(0.73,1.29) |
|  | RANTES | IVW | 9 | 0.1164 | 0.6236 | 0.94(0.75,9.19) |
|  | RANTES | MR Egger | 9 | 0.3324 | 0.8599 | 0.94(0.49,9.81) |
|  | RANTES | Weighted median | 9 | 0.1414 | 0.4684 | 0.90(0.68,9.19) |
|  | RANTES | Maximum likelihood | 9 | 0.1030 | 0.5711 | 0.94(0.77,9.15) |
|  | RANTES | Simple mode | 9 | 0.2353 | 0.5901 | 0.88(0.55,9.39) |
|  | IL_1B | IVW | 4 | 0.1744 | 0.1605 | 1.28(0.91,4.80) |
|  | IL_1B | MR Egger | 4 | 0.4907 | 0.1729 | 2.78(1.06,4.27) |
|  | IL_1B | Weighted median | 4 | 0.2285 | 0.4127 | 1.21(0.77,4.89) |
|  | IL_1B | Maximum likelihood | 4 | 0.1684 | 0.1297 | 1.29(0.93,4.80) |
|  | IL_1B | Simple mode | 4 | 0.3072 | 0.5870 | 1.20(0.66,4.20) |
|  | SDF_1A | IVW | 4 | 0.2510 | 0.7714 | 0.93(0.57,4.52) |
|  | SDF_1A | MR Egger | 4 | 0.4310 | 0.2762 | 1.90(0.81,4.41) |
|  | SDF_1A | Weighted median | 4 | 0.2869 | 0.1828 | 0.68(0.39,4.20) |
|  | SDF_1A | Maximum likelihood | 4 | 0.2271 | 0.7314 | 0.93(0.59,4.44) |
|  | SDF_1A | Simple mode | 4 | 0.4047 | 0.4030 | 0.67(0.31,4.49) |
|  | IL_4 | IVW | 13 | 0.1212 | 0.1006 | 1.22(0.96,1.55) |
|  | IL_4 | MR Egger | 13 | 0.2628 | 0.9259 | 1.03(0.61,1.72) |
|  | IL_4 | Weighted median | 13 | 0.1805 | 0.4013 | 1.16(0.82,1.66) |
|  | IL_4 | Maximum likelihood | 13 | 0.1332 | 0.1182 | 1.23(0.95,1.60) |
|  | IL_4 | Simple mode | 13 | 0.3143 | 0.7681 | 1.10(0.59,1.04) |
|  | MIF | IVW | 7 | 0.0636 | 0.8230 | 0.99(0.87,7.12) |
|  | MIF | MR Egger | 7 | 0.2216 | 0.9785 | 0.99(0.64,7.53) |
|  | MIF | Weighted median | 7 | 0.1432 | 0.8380 | 1.03(0.78,7.36) |
|  | MIF | Maximum likelihood | 7 | 0.1158 | 0.9016 | 0.99(0.79,7.24) |
|  | MIF | Simple mode | 7 | 0.2021 | 0.8549 | 1.04(0.70,7.54) |
|  | VEGF | IVW | 12 | 0.0481 | 0.6025 | 0.98(0.89,1.07) |
|  | VEGF | MR Egger | 12 | 0.1012 | 0.7318 | 0.96(0.79,1.18) |
|  | VEGF | Weighted median | 12 | 0.0717 | 0.4583 | 0.95(0.82,1.09) |
|  | VEGF | Maximum likelihood | 12 | 0.0640 | 0.6924 | 0.97(0.86,1.11) |
|  | VEGF | Simple mode | 12 | 0.1829 | 0.7461 | 0.94(0.66,1.35) |
|  | MIP_1A | IVW | 3 | 0.078252 | 3.34E-12 | 0.58(0.50,3.68) |
|  | MIP_1A | MR Egger | 3 | 0.4707 | 0.4294 | 0.56(0.22,3.40) |
|  | MIP_1A | Weighted median | 3 | 0.22734 | 0.0120 | 0.57(0.36,3.88) |
|  | MIP_1A | Maximum likelihood | 3 | 0.193269 | 0.0047 | 0.58(0.40,3.85) |
|  | MIP_1A | Simple mode | 3 | 0.2614 | 0.1488 | 0.55(0.33,3.92) |
|  | GROA | IVW | 8 | 0.0557 | 0.1226 | 0.92(0.82,8.02) |
|  | GROA | MR Egger | 8 | 0.1497 | 0.3336 | 0.85(0.64,8.15) |
|  | GROA | Weighted median | 8 | 0.0755 | 0.2147 | 0.91(0.79,8.06) |
|  | GROA | Maximum likelihood | 8 | 0.0626 | 0.1641 | 0.92(0.81,8.04) |
|  | GROA | Simple mode | 8 | 0.1170 | 0.9488 | 0.99(0.79,8.25) |
|  | IL_8 | IVW | 8 | 0.1281 | 0.3393 | 0.88(0.69,8.14) |
|  | IL_8 | MR Egger | 8 | 0.2545 | 0.8387 | 0.95(0.58,8.56) |
|  | IL_8 | Weighted median | 8 | 0.1468 | 0.7337 | 0.95(0.71,8.27) |
|  | IL_8 | Maximum likelihood | 8 | 0.1133 | 0.2467 | 0.88(0.70,8.10) |
|  | IL_8 | Simple mode | 8 | 0.2328 | 0.7094 | 0.91(0.58,8.44) |
|  | IFN_G | IVW | 8 | 0.2056 | 0.8351 | 0.96(0.64,8.43) |
|  | IFN_G | MR Egger | 8 | 0.4140 | 0.5790 | 0.78(0.35,8.77) |
|  | IFN_G | Weighted median | 8 | 0.2076 | 0.9423 | 0.99(0.66,8.48) |
|  | IFN_G | Maximum likelihood | 8 | 0.1599 | 0.7732 | 0.95(0.70,8.31) |
|  | IFN_G | Simple mode | 8 | 0.3144 | 0.8669 | 1.06(0.57,8.96) |
|  | FGF_BASIC | IVW | 5 | 0.2096 | 0.5100 | 1.15(0.76,5.73) |
|  | FGF_BASIC | MR Egger | 5 | 0.4952 | 0.5904 | 0.74(0.28,5.96) |
|  | FGF_BASIC | Weighted median | 5 | 0.2357 | 0.3449 | 1.25(0.79,5.98) |
|  | FGF_BASIC | Maximum likelihood | 5 | 0.1941 | 0.4561 | 1.16(0.79,5.69) |
|  | FGF_BASIC | Simple mode | 5 | 0.4459 | 0.2906 | 1.72(0.72,5.12) |
|  | TNF_B | IVW | 4 | 0.0571 | 0.8179 | 0.99(0.88,4.10) |
|  | TNF_B | MR Egger | 4 | 0.1458 | 0.6248 | 1.09(0.82,4.45) |
|  | TNF_B | Weighted median | 4 | 0.1050 | 0.8854 | 0.98(0.80,4.21) |
|  | TNF_B | Maximum likelihood | 4 | 0.0896 | 0.8822 | 0.99(0.83,4.18) |
|  | TNF_B | Simple mode | 4 | 0.1457 | 0.4788 | 0.89(0.67,4.18) |
|  | MIP_1B | IVW | 17 | 0.060869 | 0.0362 | 0.88(0.78,1.99) |
|  | MIP_1B | MR Egger | 17 | 0.1018 | 0.3564 | 0.91(0.74,1.11) |
|  | MIP_1B | Weighted median | 17 | 0.0778 | 0.1079 | 0.88(0.76,1.03) |
|  | MIP_1B | Maximum likelihood | 17 | 0.052258 | 0.0153 | 0.88(0.80,1.98) |
|  | MIP_1B | Simple mode | 17 | 0.1497 | 0.4660 | 1.12(0.83,1.50) |
|  | SCGF_B | IVW | 17 | 0.059809 | 0.0420 | 1.13(1.00,1.27) |
|  | SCGF_B | MR Egger | 17 | 0.1193 | 0.9655 | 1.01(0.80,1.27) |
|  | SCGF_B | Weighted median | 17 | 0.0923 | 0.0886 | 1.17(0.98,1.40) |
|  | SCGF_B | Maximum likelihood | 17 | 0.0649 | 0.0527 | 1.13(1.00,1.29) |
|  | SCGF_B | Simple mode | 17 | 0.1831 | 0.2042 | 1.27(0.89,1.82) |
|  | CTACK | IVW | 13 | 0.0565 | 0.2526 | 1.07(0.95,1.19) |
|  | CTACK | MR Egger | 13 | 0.1394 | 0.7475 | 1.05(0.80,1.38) |
|  | CTACK | Weighted median | 13 | 0.0982 | 0.5299 | 1.06(0.88,1.29) |
|  | CTACK | Maximum likelihood | 13 | 0.0754 | 0.3872 | 1.07(0.92,1.24) |
|  | CTACK | Simple mode | 13 | 0.1751 | 0.9645 | 1.01(0.72,1.42) |
|  | HGF | IVW | 6 | 0.2693 | 0.3989 | 1.26(0.74,6.13) |
|  | HGF | MR Egger | 6 | 0.6966 | 0.5960 | 1.49(0.38,6.85) |
|  | HGF | Weighted median | 6 | 0.2456 | 0.3788 | 1.24(0.77,6.01) |
|  | HGF | Maximum likelihood | 6 | 0.1735 | 0.1591 | 1.28(0.91,6.79) |
|  | HGF | Simple mode | 6 | 0.4777 | 0.8515 | 0.91(0.36,6.32) |
|  | IL_5 | IVW | 8 | 0.0838 | 0.6783 | 1.04(0.88,8.22) |
|  | IL_5 | MR Egger | 8 | 0.2318 | 0.9726 | 1.01(0.64,8.59) |
|  | IL_5 | Weighted median | 8 | 0.1292 | 0.6658 | 0.95(0.73,8.22) |
|  | IL_5 | Maximum likelihood | 8 | 0.1021 | 0.7284 | 1.04(0.85,8.27) |
|  | IL_5 | Simple mode | 8 | 0.2006 | 0.7399 | 0.93(0.63,8.38) |
|  | IP_10 | IVW | 9 | 0.065928 | 0.0002 | 0.78(0.69,9.89) |
|  | IP_10 | MR Egger | 9 | 0.2116 | 0.2401 | 0.76(0.50,9.15) |
|  | IP_10 | Weighted median | 9 | 0.134181 | 0.0133 | 0.72(0.55,9.93) |
|  | IP_10 | Maximum likelihood | 9 | 0.103442 | 0.0172 | 0.78(0.64,9.96) |
|  | IP_10 | Simple mode | 9 | 0.1985 | 0.1222 | 0.71(0.48,9.05) |
|  | MIG | IVW | 10 | 0.109816 | 0.0305 | 0.79(0.64,1.98) |
|  | MIG | MR Egger | 10 | 0.19289 | 0.0260 | 0.59(0.40,1.86) |
|  | MIG | Weighted median | 10 | 0.140664 | 0.0311 | 0.74(0.56,1.97) |
|  | MIG | Maximum likelihood | 10 | 0.095274 | 0.0108 | 0.78(0.65,1.95) |
|  | MIG | Simple mode | 10 | 0.2618 | 0.1348 | 0.65(0.39,1.09) |
|  | EOTAXIN | IVW | 13 | 0.0848 | 0.5457 | 0.95(0.80,1.12) |
|  | EOTAXIN | MR Egger | 13 | 0.1998 | 0.7154 | 0.93(0.63,1.37) |
|  | EOTAXIN | Weighted median | 13 | 0.1308 | 0.8277 | 1.03(0.80,1.33) |
|  | EOTAXIN | Maximum likelihood | 13 | 0.0952 | 0.5890 | 0.95(0.79,1.14) |
|  | EOTAXIN | Simple mode | 13 | 0.2017 | 0.4444 | 1.17(0.79,1.74) |
|  | M_CSF | IVW | 9 | 0.0562 | 0.9147 | 0.99(0.89,9.11) |
|  | M_CSF | MR Egger | 9 | 0.1590 | 0.8460 | 1.03(0.76,9.41) |
|  | M_CSF | Weighted median | 9 | 0.1018 | 0.7334 | 0.97(0.79,9.18) |
|  | M_CSF | Maximum likelihood | 9 | 0.0821 | 0.9410 | 0.99(0.85,9.17) |
|  | M_CSF | Simple mode | 9 | 0.1566 | 0.9084 | 0.98(0.72,9.33) |
|  | IL_2RA | IVW | 6 | 0.1582 | 0.6051 | 0.92(0.68,6.26) |
|  | IL_2RA | MR Egger | 6 | 0.1769 | 0.0968 | 0.68(0.48,6.97) |
|  | IL_2RA | Weighted median | 6 | 0.1147 | 0.3868 | 0.91(0.72,6.13) |
|  | IL_2RA | Maximum likelihood | 6 | 0.0927 | 0.3506 | 0.92(0.76,6.10) |
|  | IL_2RA | Simple mode | 6 | 0.3236 | 0.2061 | 1.60(0.85,6.02) |
|  | SCF | IVW | 9 | 0.1172 | 0.1989 | 1.16(0.92,9.46) |
|  | SCF | MR Egger | 9 | 0.3537 | 0.1860 | 1.68(0.84,9.36) |
|  | SCF | Weighted median | 9 | 0.1936 | 0.4018 | 1.18(0.80,9.72) |
|  | SCF | Maximum likelihood | 9 | 0.1463 | 0.2937 | 1.17(0.88,9.55) |
|  | SCF | Simple mode | 9 | 0.2949 | 0.5894 | 1.18(0.66,9.10) |
|  | IL_9 | IVW | 4 | 0.1369 | 0.1690 | 0.83(0.63,4.08) |
|  | IL_9 | MR Egger | 4 | 0.8923 | 0.8467 | 0.82(0.14,4.73) |
|  | IL_9 | Weighted median | 4 | 0.2248 | 0.7731 | 0.94(0.60,4.46) |
|  | IL_9 | Maximum likelihood | 4 | 0.1786 | 0.2808 | 0.82(0.58,4.17) |
|  | IL_9 | Simple mode | 4 | 0.3206 | 0.8922 | 0.95(0.51,4.79) |
|  | MCP_3 | IVW | 5 | 0.1192 | 0.6977 | 1.05(0.83,5.32) |
|  | MCP_3 | MR Egger | 5 | 0.4190 | 0.6911 | 0.83(0.37,5.89) |
|  | MCP_3 | Weighted median | 5 | 0.1180 | 0.3498 | 1.12(0.89,5.41) |
|  | MCP_3 | Maximum likelihood | 5 | 0.0820 | 0.5391 | 1.05(0.90,5.23) |
|  | MCP_3 | Simple mode | 5 | 0.1979 | 0.4945 | 1.16(0.79,5.71) |
|  | PDGF_BB | IVW | 12 | 0.066248 | 0.0004 | 1.26(1.11,1.44) |
|  | PDGF_BB | MR Egger | 12 | 0.1787 | 0.6163 | 1.10(0.77,1.56) |
|  | PDGF_BB | Weighted median | 12 | 0.1212 | 0.0723 | 1.24(0.98,1.58) |
|  | PDGF_BB | Maximum likelihood | 12 | 0.092844 | 0.0103 | 1.27(1.06,1.52) |
|  | PDGF_BB | Simple mode | 12 | 0.2047 | 0.0507 | 1.57(1.05,1.34) |
|  | IL_1RA | IVW | 6 | 0.1387 | 0.1751 | 1.21(0.92,6.58) |
|  | IL_1RA | MR Egger | 6 | 0.3582 | 0.1068 | 2.10(1.04,6.24) |
|  | IL_1RA | Weighted median | 6 | 0.1754 | 0.4110 | 1.16(0.82,6.63) |
|  | IL_1RA | Maximum likelihood | 6 | 0.1374 | 0.1541 | 1.22(0.93,6.59) |
|  | IL_1RA | Simple mode | 6 | 0.2731 | 0.9725 | 1.01(0.59,6.72) |
|  | IL_16 | IVW | 9 | 0.1002 | 0.6049 | 0.95(0.78,9.16) |
|  | IL_16 | MR Egger | 9 | 0.1547 | 0.1330 | 0.77(0.57,9.04) |
|  | IL_16 | Weighted median | 9 | 0.0994 | 0.3025 | 0.90(0.74,9.10) |
|  | IL_16 | Maximum likelihood | 9 | 0.0789 | 0.4988 | 0.95(0.81,9.11) |
|  | IL_16 | Simple mode | 9 | 0.1554 | 0.5050 | 0.90(0.66,9.22) |
|  | G_CSF | IVW | 8 | 0.057353 | 0.0020 | 0.84(0.75,8.94) |
|  | G_CSF | MR Egger | 8 | 0.2747 | 0.5254 | 0.83(0.49,8.42) |
|  | G_CSF | Weighted median | 8 | 0.1807 | 0.3392 | 0.84(0.59,8.20) |
|  | G_CSF | Maximum likelihood | 8 | 0.1512 | 0.2379 | 0.84(0.62,8.13) |
|  | G_CSF | Simple mode | 8 | 0.2460 | 0.5027 | 0.84(0.52,8.36) |
|  | IL_10 | IVW | 12 | 0.1037 | 0.2184 | 0.88(0.72,1.08) |
|  | IL_10 | MR Egger | 12 | 0.2252 | 0.7801 | 0.94(0.60,1.46) |
|  | IL_10 | Weighted median | 12 | 0.1195 | 0.3738 | 0.90(0.71,1.14) |
|  | IL_10 | Maximum likelihood | 12 | 0.0952 | 0.1927 | 0.88(0.73,1.06) |
|  | IL_10 | Simple mode | 12 | 0.2110 | 0.5376 | 0.87(0.58,1.32) |
|  | IL_18 | IVW | 11 | 0.0622 | 0.4334 | 0.95(0.84,1.08) |
|  | IL_18 | MR Egger | 11 | 0.1639 | 0.8254 | 0.96(0.70,1.33) |
|  | IL_18 | Weighted median | 11 | 0.0966 | 0.8131 | 0.98(0.81,1.18) |
|  | IL_18 | Maximum likelihood | 11 | 0.0719 | 0.4885 | 0.95(0.83,1.10) |
|  | IL_18 | Simple mode | 11 | 0.1847 | 0.9114 | 1.02(0.71,1.47) |
|  | TNF_A | IVW | 5 | 0.1320 | 0.0982 | 0.80(0.62,5.04) |
|  | TNF_A | MR Egger | 5 | 0.2349 | 0.1313 | 0.62(0.39,5.98) |
|  | TNF_A | Weighted median | 5 | 0.1928 | 0.3997 | 0.85(0.58,5.24) |
|  | TNF_A | Maximum likelihood | 5 | 0.1384 | 0.1025 | 0.80(0.61,5.05) |
|  | TNF_A | Simple mode | 5 | 0.2426 | 0.5004 | 0.84(0.52,5.34) |
|  | IL_7 | IVW | 9 | 0.0938 | 0.6639 | 1.04(0.87,9.25) |
|  | IL_7 | MR Egger | 9 | 0.2089 | 0.2694 | 1.28(0.85,9.93) |
|  | IL_7 | Weighted median | 9 | 0.1031 | 0.4948 | 0.93(0.76,9.14) |
|  | IL_7 | Maximum likelihood | 9 | 0.0776 | 0.5928 | 1.04(0.90,9.21) |
|  | IL_7 | Simple mode | 9 | 0.2024 | 0.7350 | 0.93(0.63,9.39) |
|  | IL_2 | IVW | 7 | 0.1104 | 0.3234 | 1.12(0.90,7.38) |
|  | IL_2 | MR Egger | 7 | 0.2091 | 0.1105 | 1.50(1.00,7.26) |
|  | IL_2 | Weighted median | 7 | 0.1371 | 0.4791 | 1.10(0.84,7.44) |
|  | IL_2 | Maximum likelihood | 7 | 0.1086 | 0.3006 | 1.12(0.90,7.38) |
|  | IL_2 | Simple mode | 7 | 0.2110 | 0.8929 | 1.03(0.68,7.56) |
|  | IL_12_P70 | IVW | 12 | 0.0650 | 0.3473 | 0.94(0.83,1.07) |
|  | IL_12_P70 | MR Egger | 12 | 0.1357 | 0.7042 | 0.95(0.73,1.24) |
|  | IL_12_P70 | Weighted median | 12 | 0.0890 | 0.4289 | 0.93(0.78,1.11) |
|  | IL_12_P70 | Maximum likelihood | 12 | 0.0790 | 0.4349 | 0.94(0.81,1.10) |
|  | IL_12_P70 | Simple mode | 12 | 0.2140 | 0.3983 | 0.83(0.54,1.26) |
|  | TRAIL | IVW | 11 | 0.0803 | 0.8205 | 0.98(0.84,1.15) |
|  | TRAIL | MR Egger | 11 | 0.1052 | 0.8864 | 1.02(0.83,1.25) |
|  | TRAIL | Weighted median | 11 | 0.0887 | 0.6671 | 0.96(0.81,1.15) |
|  | TRAIL | Maximum likelihood | 11 | 0.0683 | 0.7875 | 0.98(0.86,1.12) |
|  | TRAIL | Simple mode | 11 | 0.1223 | 0.5844 | 0.93(0.73,1.19) |
|  | IL_17 | IVW | 7 | 0.1901 | 0.9960 | 1.00(0.69,7.45) |
|  | IL_17 | MR Egger | 7 | 0.3981 | 0.9622 | 1.02(0.47,7.23) |
|  | IL_17 | Weighted median | 7 | 0.1932 | 1.0000 | 1.00(0.68,7.46) |
|  | IL_17 | Maximum likelihood | 7 | 0.1457 | 0.9945 | 1.00(0.75,7.33) |
|  | IL_17 | Simple mode | 7 | 0.3014 | 0.7258 | 0.90(0.50,7.62) |
|  | B_NGF | IVW | 4 | 0.039137 | 2.68E-07 | 0.82(0.76,4.88) |
|  | B_NGF | MR Egger | 4 | 0.8225 | 0.8944 | 0.88(0.18,4.43) |
|  | B_NGF | Weighted median | 4 | 0.1726 | 0.1905 | 0.80(0.57,4.12) |
|  | B_NGF | Maximum likelihood | 4 | 0.1487 | 0.1750 | 0.82(0.61,4.09) |
|  | B_NGF | Simple mode | 4 | 0.2191 | 0.3595 | 0.79(0.51,4.21) |
| **SAH** | MCP_1_MCAF | IVW | 13 | 0.0407 | 0.6390 | 0.98(0.91,1.06) |
|  | MCP_1_MCAF | MR Egger | 13 | 0.1616 | 0.5034 | 1.12(0.81,1.54) |
|  | MCP_1_MCAF | Weighted median | 13 | 0.0829 | 0.8138 | 1.02(0.87,1.20) |
|  | MCP_1_MCAF | Maximum likelihood | 13 | 0.0665 | 0.7736 | 0.98(0.86,1.12) |
|  | MCP_1_MCAF | Simple mode | 13 | 0.1433 | 0.8915 | 0.98(0.74,1.30) |
|  | IL_6 | IVW | 9 | 0.2067 | 0.5076 | 1.15(0.76,9.72) |
|  | IL_6 | MR Egger | 9 | 0.4887 | 0.5960 | 0.76(0.29,9.99) |
|  | IL_6 | Weighted median | 9 | 0.1465 | 0.9037 | 0.98(0.74,9.31) |
|  | IL_6 | Maximum likelihood | 9 | 0.1127 | 0.1652 | 1.17(0.94,9.46) |
|  | IL_6 | Simple mode | 9 | 0.2095 | 0.5594 | 0.88(0.58,9.33) |
|  | IL_13 | IVW | 10 | 0.0454 | 0.6095 | 0.98(0.89,1.07) |
|  | IL_13 | MR Egger | 10 | 0.0918 | 0.7676 | 0.97(0.81,1.16) |
|  | IL_13 | Weighted median | 10 | 0.0557 | 0.9349 | 1.00(0.89,1.11) |
|  | IL_13 | Maximum likelihood | 10 | 0.0462 | 0.6085 | 0.98(0.89,1.07) |
|  | IL_13 | Simple mode | 10 | 0.1205 | 0.6024 | 0.94(0.74,1.19) |
|  | RANTES | IVW | 9 | 0.0614 | 0.9523 | 1.00(0.88,9.12) |
|  | RANTES | MR Egger | 9 | 0.1857 | 0.4192 | 1.17(0.81,9.69) |
|  | RANTES | Weighted median | 9 | 0.0895 | 0.8711 | 0.99(0.83,9.17) |
|  | RANTES | Maximum likelihood | 9 | 0.0705 | 0.9574 | 1.00(0.87,9.14) |
|  | RANTES | Simple mode | 9 | 0.1438 | 0.8492 | 1.03(0.78,9.36) |
|  | IL_1B | IVW | 4 | 0.1131 | 0.3518 | 1.11(0.89,4.39) |
|  | IL_1B | MR Egger | 4 | 0.3515 | 0.3668 | 1.50(0.75,4.99) |
|  | IL_1B | Weighted median | 4 | 0.1469 | 0.2988 | 1.16(0.87,4.55) |
|  | IL_1B | Maximum likelihood | 4 | 0.1137 | 0.3411 | 1.11(0.89,4.39) |
|  | IL_1B | Simple mode | 4 | 0.1978 | 0.3554 | 1.24(0.84,4.83) |
|  | SDF_1A | IVW | 4 | 0.0967 | 0.0655 | 0.84(0.69,4.01) |
|  | SDF_1A | MR Egger | 4 | 0.2966 | 0.8317 | 1.07(0.60,4.92) |
|  | SDF_1A | Weighted median | 4 | 0.1803 | 0.1814 | 0.79(0.55,4.12) |
|  | SDF_1A | Maximum likelihood | 4 | 0.1553 | 0.2403 | 0.83(0.61,4.13) |
|  | SDF_1A | Simple mode | 4 | 0.2692 | 0.3110 | 0.72(0.43,4.22) |
|  | IL_4 | IVW | 13 | 0.0993 | 0.2031 | 1.13(0.93,1.38) |
|  | IL_4 | MR Egger | 13 | 0.1983 | 0.8002 | 0.95(0.64,1.40) |
|  | IL_4 | Weighted median | 13 | 0.1341 | 0.8375 | 1.03(0.79,1.34) |
|  | IL_4 | Maximum likelihood | 13 | 0.0927 | 0.1472 | 1.14(0.95,1.37) |
|  | IL_4 | Simple mode | 13 | 0.2704 | 0.6792 | 0.89(0.52,1.52) |
|  | MIF | IVW | 7 | 0.0679 | 0.6744 | 0.97(0.85,7.11) |
|  | MIF | MR Egger | 7 | 0.1526 | 0.4797 | 1.12(0.83,7.52) |
|  | MIF | Weighted median | 7 | 0.1107 | 0.7891 | 0.97(0.78,7.21) |
|  | MIF | Maximum likelihood | 7 | 0.0806 | 0.7171 | 0.97(0.83,7.14) |
|  | MIF | Simple mode | 7 | 0.1523 | 0.7751 | 0.96(0.71,7.29) |
|  | VEGF | IVW | 12 | 0.0342 | 0.9292 | 1.00(0.94,1.07) |
|  | VEGF | MR Egger | 12 | 0.0699 | 0.9419 | 0.99(0.87,1.14) |
|  | VEGF | Weighted median | 12 | 0.0481 | 0.9815 | 1.00(0.91,1.10) |
|  | VEGF | Maximum likelihood | 12 | 0.0443 | 0.9452 | 1.00(0.92,1.09) |
|  | VEGF | Simple mode | 12 | 0.1151 | 0.5580 | 0.93(0.74,1.17) |
|  | MIP_1A | IVW | 3 | 0.107557 | 0.0013 | 0.71(0.57,3.87) |
|  | MIP_1A | MR Egger | 3 | 0.3250 | 0.9650 | 1.02(0.54,3.93) |
|  | MIP_1A | Weighted median | 3 | 0.1708 | 0.0788 | 0.74(0.53,3.04) |
|  | MIP_1A | Maximum likelihood | 3 | 0.135068 | 0.0089 | 0.70(0.54,3.92) |
|  | MIP_1A | Simple mode | 3 | 0.1957 | 0.2783 | 0.75(0.51,3.10) |
|  | GROA | IVW | 8 | 0.0550 | 0.3757 | 1.05(0.94,8.17) |
|  | GROA | MR Egger | 8 | 0.1315 | 0.5844 | 0.93(0.72,8.20) |
|  | GROA | Weighted median | 8 | 0.0586 | 0.5767 | 1.03(0.92,8.16) |
|  | GROA | Maximum likelihood | 8 | 0.0434 | 0.2505 | 1.05(0.97,8.14) |
|  | GROA | Simple mode | 8 | 0.0933 | 0.6997 | 1.04(0.86,8.25) |
|  | IL_8 | IVW | 8 | 0.1165 | 0.8454 | 0.98(0.78,8.23) |
|  | IL_8 | MR Egger | 8 | 0.2067 | 0.2854 | 0.78(0.52,8.18) |
|  | IL_8 | Weighted median | 8 | 0.1051 | 0.4942 | 1.07(0.87,8.32) |
|  | IL_8 | Maximum likelihood | 8 | 0.0801 | 0.7624 | 0.98(0.83,8.14) |
|  | IL_8 | Simple mode | 8 | 0.1476 | 0.5313 | 1.10(0.83,8.47) |
|  | IFN_G | IVW | 8 | 0.1001 | 0.7207 | 1.04(0.85,8.26) |
|  | IFN_G | MR Egger | 8 | 0.2067 | 0.9759 | 1.01(0.67,8.51) |
|  | IFN_G | Weighted median | 8 | 0.1486 | 0.8032 | 1.04(0.78,8.39) |
|  | IFN_G | Maximum likelihood | 8 | 0.1098 | 0.7319 | 1.04(0.84,8.29) |
|  | IFN_G | Simple mode | 8 | 0.2092 | 0.9282 | 1.02(0.68,8.54) |
|  | FGF_BASIC | IVW | 5 | 0.1434 | 0.9278 | 1.01(0.76,5.34) |
|  | FGF_BASIC | MR Egger | 5 | 0.3706 | 0.7131 | 0.86(0.42,5.78) |
|  | FGF_BASIC | Weighted median | 5 | 0.1720 | 0.8548 | 1.03(0.74,5.45) |
|  | FGF_BASIC | Maximum likelihood | 5 | 0.1327 | 0.9190 | 1.01(0.78,5.31) |
|  | FGF_BASIC | Simple mode | 5 | 0.2780 | 0.6677 | 1.14(0.66,5.96) |
|  | TNF_B | IVW | 4 | 0.0392 | 0.6649 | 0.98(0.91,4.06) |
|  | TNF_B | MR Egger | 4 | 0.1007 | 0.6572 | 1.05(0.86,4.28) |
|  | TNF_B | Weighted median | 4 | 0.0710 | 0.9663 | 1.00(0.87,4.15) |
|  | TNF_B | Maximum likelihood | 4 | 0.0619 | 0.7820 | 0.98(0.87,4.11) |
|  | TNF_B | Simple mode | 4 | 0.1096 | 0.9599 | 1.01(0.81,4.25) |
|  | MIP_1B | IVW | 17 | 0.0392 | 0.5727 | 0.98(0.91,1.06) |
|  | MIP_1B | MR Egger | 17 | 0.0658 | 0.6160 | 0.97(0.85,1.10) |
|  | MIP_1B | Weighted median | 17 | 0.0496 | 0.7903 | 1.01(0.92,1.12) |
|  | MIP_1B | Maximum likelihood | 17 | 0.0358 | 0.5309 | 0.98(0.91,1.05) |
|  | MIP_1B | Simple mode | 17 | 0.1053 | 0.8079 | 0.97(0.79,1.20) |
|  | SCGF_B | IVW | 17 | 0.0435 | 0.4179 | 1.04(0.95,1.13) |
|  | SCGF_B | MR Egger | 17 | 0.0833 | 0.8461 | 0.98(0.84,1.16) |
|  | SCGF_B | Weighted median | 17 | 0.0641 | 0.4315 | 1.05(0.93,1.19) |
|  | SCGF_B | Maximum likelihood | 17 | 0.0451 | 0.4296 | 1.04(0.95,1.13) |
|  | SCGF_B | Simple mode | 17 | 0.1187 | 0.2838 | 1.14(0.90,1.44) |
|  | CTACK | IVW | 13 | 0.0475 | 0.7345 | 1.02(0.93,1.12) |
|  | CTACK | MR Egger | 13 | 0.0963 | 0.2095 | 0.88(0.73,1.06) |
|  | CTACK | Weighted median | 13 | 0.0712 | 0.6050 | 1.04(0.90,1.19) |
|  | CTACK | Maximum likelihood | 13 | 0.0523 | 0.7509 | 1.02(0.92,1.13) |
|  | CTACK | Simple mode | 13 | 0.1280 | 0.6562 | 0.94(0.73,1.21) |
|  | HGF | IVW | 6 | 0.1467 | 0.8881 | 1.02(0.77,6.36) |
|  | HGF | MR Egger | 6 | 0.3583 | 0.5357 | 1.27(0.63,6.57) |
|  | HGF | Weighted median | 6 | 0.1580 | 0.9686 | 1.01(0.74,6.37) |
|  | HGF | Maximum likelihood | 6 | 0.1184 | 0.8550 | 1.02(0.81,6.29) |
|  | HGF | Simple mode | 6 | 0.2938 | 0.5783 | 0.84(0.47,6.49) |
|  | IL_5 | IVW | 8 | 0.023729 | 0.0045 | 1.07(1.02,8.12) |
|  | IL_5 | MR Egger | 8 | 0.1614 | 0.6074 | 1.09(0.80,8.50) |
|  | IL_5 | Weighted median | 8 | 0.0863 | 0.3585 | 1.08(0.91,8.28) |
|  | IL_5 | Maximum likelihood | 8 | 0.0703 | 0.3365 | 1.07(0.93,8.23) |
|  | IL_5 | Simple mode | 8 | 0.1287 | 0.3911 | 1.12(0.87,8.45) |
|  | IP_10 | IVW | 9 | 0.054039 | 8.29E-05 | 0.81(0.73,9.90) |
|  | IP_10 | MR Egger | 9 | 0.1455 | 0.0501 | 0.71(0.53,9.94) |
|  | IP_10 | Weighted median | 9 | 0.096841 | 0.0124 | 0.78(0.65,9.95) |
|  | IP_10 | Maximum likelihood | 9 | 0.072544 | 0.0027 | 0.80(0.70,9.93) |
|  | IP_10 | Simple mode | 9 | 0.1525 | 0.1029 | 0.76(0.56,9.02) |
|  | MIG | IVW | 10 | 0.0761 | 0.8669 | 0.99(0.85,1.15) |
|  | MIG | MR Egger | 10 | 0.1339 | 0.1437 | 0.80(0.62,1.05) |
|  | MIG | Weighted median | 10 | 0.0902 | 0.7012 | 0.97(0.81,1.15) |
|  | MIG | Maximum likelihood | 10 | 0.0664 | 0.8400 | 0.99(0.87,1.12) |
|  | MIG | Simple mode | 10 | 0.1673 | 0.6909 | 0.93(0.67,1.30) |
|  | EOTAXIN | IVW | 13 | 0.061055 | 0.0116 | 1.17(1.03,1.31) |
|  | EOTAXIN | MR Egger | 13 | 0.1383 | 0.1066 | 1.28(0.97,1.67) |
|  | EOTAXIN | Weighted median | 13 | 0.086942 | 0.0056 | 1.27(1.07,1.51) |
|  | EOTAXIN | Maximum likelihood | 13 | 0.066254 | 0.0174 | 1.17(1.03,1.33) |
|  | EOTAXIN | Simple mode | 13 | 0.1465 | 0.2379 | 1.20(0.90,1.60) |
|  | M_CSF | IVW | 9 | 0.0360 | 0.9450 | 1.00(0.93,9.08) |
|  | M_CSF | MR Egger | 9 | 0.1097 | 0.5546 | 1.07(0.86,9.33) |
|  | M_CSF | Weighted median | 9 | 0.0716 | 0.6112 | 1.04(0.90,9.19) |
|  | M_CSF | Maximum likelihood | 9 | 0.0566 | 0.9646 | 1.00(0.90,9.12) |
|  | M_CSF | Simple mode | 9 | 0.1103 | 0.8613 | 1.02(0.82,9.27) |
|  | IL_2RA | IVW | 6 | 0.0803 | 0.6401 | 0.96(0.82,6.13) |
|  | IL_2RA | MR Egger | 6 | 0.1172 | 0.3003 | 0.87(0.69,6.09) |
|  | IL_2RA | Weighted median | 6 | 0.0734 | 0.1998 | 0.91(0.79,6.05) |
|  | IL_2RA | Maximum likelihood | 6 | 0.0630 | 0.5389 | 0.96(0.85,6.09) |
|  | IL_2RA | Simple mode | 6 | 0.1720 | 0.2550 | 1.25(0.89,6.75) |
|  | SCF | IVW | 9 | 0.1636 | 0.2993 | 1.19(0.86,9.63) |
|  | SCF | MR Egger | 9 | 0.4032 | 0.1273 | 2.01(0.91,9.43) |
|  | SCF | Weighted median | 9 | 0.1528 | 0.5791 | 1.09(0.81,9.47) |
|  | SCF | Maximum likelihood | 9 | 0.1074 | 0.0945 | 1.20(0.97,9.48) |
|  | SCF | Simple mode | 9 | 0.2121 | 0.8125 | 1.05(0.70,9.60) |
|  | IL_9 | IVW | 4 | 0.1528 | 0.3948 | 0.88(0.65,4.18) |
|  | IL_9 | MR Egger | 4 | 0.8282 | 0.5865 | 1.70(0.34,4.63) |
|  | IL_9 | Weighted median | 4 | 0.1560 | 0.0900 | 0.77(0.57,4.04) |
|  | IL_9 | Maximum likelihood | 4 | 0.1267 | 0.2726 | 0.87(0.68,4.12) |
|  | IL_9 | Simple mode | 4 | 0.2426 | 0.2964 | 0.74(0.46,4.18) |
|  | MCP_3 | IVW | 5 | 0.0794 | 0.8273 | 1.02(0.87,5.19) |
|  | MCP_3 | MR Egger | 5 | 0.2847 | 0.7080 | 0.89(0.51,5.55) |
|  | MCP_3 | Weighted median | 5 | 0.0794 | 0.3940 | 0.93(0.80,5.09) |
|  | MCP_3 | Maximum likelihood | 5 | 0.0567 | 0.7405 | 1.02(0.91,5.14) |
|  | MCP_3 | Simple mode | 5 | 0.1044 | 0.4006 | 0.91(0.74,5.11) |
|  | PDGF_BB | IVW | 12 | 0.070492 | 0.0366 | 1.16(1.01,1.33) |
|  | PDGF_BB | MR Egger | 12 | 0.1316 | 0.9418 | 0.99(0.77,1.28) |
|  | PDGF_BB | Weighted median | 12 | 0.0851 | 0.0871 | 1.16(0.98,1.37) |
|  | PDGF_BB | Maximum likelihood | 12 | 0.064515 | 0.0195 | 1.16(1.02,1.32) |
|  | PDGF_BB | Simple mode | 12 | 0.1422 | 0.7732 | 1.04(0.79,1.38) |
|  | IL_1RA | IVW | 6 | 0.1000 | 0.9648 | 1.00(0.82,6.21) |
|  | IL_1RA | MR Egger | 6 | 0.2793 | 0.5238 | 1.22(0.70,6.10) |
|  | IL_1RA | Weighted median | 6 | 0.1182 | 0.7425 | 1.04(0.82,6.31) |
|  | IL_1RA | Maximum likelihood | 6 | 0.0946 | 0.9611 | 1.00(0.83,6.20) |
|  | IL_1RA | Simple mode | 6 | 0.1956 | 0.7804 | 1.06(0.72,6.55) |
|  | IL_16 | IVW | 9 | 0.0598 | 0.4559 | 0.96(0.85,9.08) |
|  | IL_16 | MR Egger | 9 | 0.1008 | 0.2267 | 0.87(0.72,9.07) |
|  | IL_16 | Weighted median | 9 | 0.0703 | 0.8888 | 0.99(0.86,9.14) |
|  | IL_16 | Maximum likelihood | 9 | 0.0541 | 0.3989 | 0.96(0.86,9.06) |
|  | IL_16 | Simple mode | 9 | 0.1221 | 0.8133 | 0.97(0.76,9.23) |
|  | G_CSF | IVW | 8 | 0.0516 | 0.1985 | 0.94(0.85,8.04) |
|  | G_CSF | MR Egger | 8 | 0.1885 | 0.7443 | 0.94(0.65,8.36) |
|  | G_CSF | Weighted median | 8 | 0.1335 | 0.7402 | 0.96(0.74,8.24) |
|  | G_CSF | Maximum likelihood | 8 | 0.1043 | 0.5229 | 0.94(0.76,8.15) |
|  | G_CSF | Simple mode | 8 | 0.1720 | 0.9704 | 1.01(0.72,8.41) |
|  | IL_10 | IVW | 12 | 0.0906 | 0.4778 | 0.94(0.79,1.12) |
|  | IL_10 | MR Egger | 12 | 0.1934 | 0.7957 | 1.05(0.72,1.54) |
|  | IL_10 | Weighted median | 12 | 0.0778 | 0.9496 | 1.00(0.85,1.16) |
|  | IL_10 | Maximum likelihood | 12 | 0.0663 | 0.3138 | 0.94(0.82,1.07) |
|  | IL_10 | Simple mode | 12 | 0.1486 | 0.8044 | 0.96(0.72,1.29) |
|  | IL_18 | IVW | 11 | 0.0542 | 0.9707 | 1.00(0.90,1.11) |
|  | IL_18 | MR Egger | 11 | 0.1299 | 0.6912 | 1.05(0.82,1.36) |
|  | IL_18 | Weighted median | 11 | 0.0708 | 0.9027 | 1.01(0.88,1.16) |
|  | IL_18 | Maximum likelihood | 11 | 0.0498 | 0.9682 | 1.00(0.91,1.10) |
|  | IL_18 | Simple mode | 11 | 0.1211 | 0.8980 | 1.02(0.80,1.29) |
|  | TNF_A | IVW | 5 | 0.0762 | 0.1709 | 0.90(0.78,5.05) |
|  | TNF_A | MR Egger | 5 | 0.1613 | 0.1575 | 0.74(0.54,5.01) |
|  | TNF_A | Weighted median | 5 | 0.1205 | 0.5961 | 0.94(0.74,5.19) |
|  | TNF_A | Maximum likelihood | 5 | 0.0940 | 0.2569 | 0.90(0.75,5.08) |
|  | TNF_A | Simple mode | 5 | 0.1676 | 0.5821 | 0.90(0.65,5.26) |
|  | IL_7 | IVW | 9 | 0.027023 | 0.0132 | 1.07(1.01,9.13) |
|  | IL_7 | MR Egger | 9 | 0.1206 | 0.6181 | 1.06(0.84,9.35) |
|  | IL_7 | Weighted median | 9 | 0.0679 | 0.4494 | 1.05(0.92,9.20) |
|  | IL_7 | Maximum likelihood | 9 | 0.0533 | 0.2074 | 1.07(0.96,9.19) |
|  | IL_7 | Simple mode | 9 | 0.1074 | 0.2139 | 1.16(0.94,9.43) |
|  | IL_2 | IVW | 7 | 0.0821 | 0.1841 | 1.12(0.95,7.31) |
|  | IL_2 | MR Egger | 7 | 0.1502 | 0.1219 | 1.32(0.99,7.78) |
|  | IL_2 | Weighted median | 7 | 0.0966 | 0.0845 | 1.18(0.98,7.43) |
|  | IL_2 | Maximum likelihood | 7 | 0.0750 | 0.1321 | 1.12(0.97,7.30) |
|  | IL_2 | Simple mode | 7 | 0.1253 | 0.1416 | 1.24(0.97,7.58) |
|  | IL_12_P70 | IVW | 12 | 0.0348 | 0.2989 | 1.04(0.97,1.11) |
|  | IL_12_P70 | MR Egger | 12 | 0.0939 | 0.9536 | 0.99(0.83,1.20) |
|  | IL_12_P70 | Weighted median | 12 | 0.0630 | 0.9515 | 1.00(0.89,1.14) |
|  | IL_12_P70 | Maximum likelihood | 12 | 0.0545 | 0.5037 | 1.04(0.93,1.15) |
|  | IL_12_P70 | Simple mode | 12 | 0.1420 | 0.6144 | 1.08(0.81,1.42) |
|  | TRAIL | IVW | 11 | 0.0420 | 0.0912 | 0.93(0.86,1.01) |
|  | TRAIL | MR Egger | 11 | 0.0594 | 0.3117 | 0.94(0.84,1.05) |
|  | TRAIL | Weighted median | 11 | 0.0593 | 0.1884 | 0.92(0.82,1.04) |
|  | TRAIL | Maximum likelihood | 11 | 0.0473 | 0.1354 | 0.93(0.85,1.02) |
|  | TRAIL | Simple mode | 11 | 0.0875 | 0.4097 | 0.93(0.78,1.10) |
|  | IL_17 | IVW | 7 | 0.1291 | 0.5867 | 0.93(0.72,7.20) |
|  | IL_17 | MR Egger | 7 | 0.2682 | 0.7253 | 0.91(0.54,7.53) |
|  | IL_17 | Weighted median | 7 | 0.1323 | 0.8745 | 1.02(0.79,7.32) |
|  | IL_17 | Maximum likelihood | 7 | 0.1027 | 0.4599 | 0.93(0.76,7.13) |
|  | IL_17 | Simple mode | 7 | 0.1879 | 0.9033 | 1.02(0.71,7.48) |
|  | B_NGF | IVW | 4 | 0.1099 | 0.2982 | 0.89(0.72,4.11) |
|  | B_NGF | MR Egger | 4 | 0.6476 | 0.5823 | 1.52(0.43,4.42) |
|  | B_NGF | Weighted median | 4 | 0.1350 | 0.2172 | 0.85(0.65,4.10) |
|  | B_NGF | Maximum likelihood | 4 | 0.1045 | 0.2578 | 0.89(0.72,4.09) |
|  | B_NGF | Simple mode | 4 | 0.2152 | 0.2872 | 0.76(0.50,4.15) |

MR, mendelian randomization; IA, intracranial aneurysm; uIA, unruptured aneurysm; SAH, subarachnoid hemorrhage; CI, confidence interval; IVW, inverse variance weighting; OR, odds ratio

**Table S7** The MR-Egger results of causal effects of inflammatory cytokines on the IA, uIA and SAH in FinnGen datasets in replicate MR analysis

| **outcome** | **exposure** | **F-statistics** | **method** | **egger_intercept** | **pval** |
| --- | --- | --- | --- | --- | --- |
| IA | IL_13 | 43.79 | MR-Egger | -0.0216 | 0.6078 |
| IA | IL_1B | 23.09 | MR-Egger | -0.0281 | 0.8754 |
| IA | MIP_1A | 22.17 | MR-Egger | -0.0963 | 0.5314 |
| IA | IP_10 | 23.14 | MR-Egger | 0.0147 | 0.7838 |
| uIA | MIP_1A | 22.17 | MR-Egger | 0.0085 | 0.9360 |
| uIA | IP_10 | 23.14 | MR-Egger | 0.0063 | 0.8824 |
| uIA | PDGF_BB | 45.36 | MR-Egger | 0.0253 | 0.3777 |
| uIA | G_CSF | 23.62 | MR-Egger | 0.0011 | 0.9750 |
| uIA | B_NGF | 25.22 | MR-Egger | -0.0114 | 0.9321 |
| SAH | MIP_1A | 22.17 | MR-Egger | -0.0709 | 0.4397 |
| SAH | IP_10 | 23.14 | MR-Egger | 0.0292 | 0.3375 |

MR, mendelian randomization; IA, intracranial aneurysm; uIA, unruptured aneurysm; SAH, subarachnoid hemorrhage

**Table S8** The Cochran’s test results of causal effects of inflammatory cytokines on the uIA and SAH in FinnGen datasets in replicate MR analysis

| **outcome** | **exposure** | **method** | **Q** | **Q_pval** |
| --- | --- | --- | --- | --- |
| **IA** | IL_13 | MR Egger | 1.70 | 0.9889 |
|  | IL_13 | Inverse variance weighted | 1.98 | 0.9917 |
|  | IL_1B | MR Egger | 0.04 | 0.9789 |
|  | IL_1B | Inverse variance weighted | 0.07 | 0.9947 |
|  | MIP_1A | MR Egger | 0.08 | 0.7714 |
|  | MIP_1A | Inverse variance weighted | 0.90 | 0.6361 |
|  | IP_10 | MR Egger | 6.72 | 0.4582 |
|  | IP_10 | Inverse variance weighted | 6.80 | 0.5578 |
| **uIA** | MIP_1A | MR Egger | 0.36 | 0.5471 |
|  | MIP_1A | Inverse variance weighted | 0.37 | 0.8300 |
|  | IP_10 | MR Egger | 3.34 | 0.8518 |
|  | IP_10 | Inverse variance weighted | 3.36 | 0.9095 |
|  | PDGF_BB | MR Egger | 4.87 | 0.8996 |
|  | PDGF_BB | Inverse variance weighted | 5.72 | 0.8912 |
|  | G_CSF | MR Egger | 1.02 | 0.9849 |
|  | G_CSF | Inverse variance weighted | 1.02 | 0.9945 |
|  | B_NGF | MR Egger | 0.20 | 0.9036 |
|  | B_NGF | Inverse variance weighted | 0.21 | 0.9756 |
| **SAH** | MIP_1A | MR Egger | 0.01 | 0.9253 |
|  | MIP_1A | Inverse variance weighted | 1.47 | 0.4789 |
|  | IP_10 | MR Egger | 3.68 | 0.8161 |
|  | IP_10 | Inverse variance weighted | 4.74 | 0.7853 |

MR, mendelian randomization; IA, intracranial aneurysm; uIA, unruptured aneurysm; SAH, subarachnoid hemorrhage

**Table S9** IVs used in reverse MR analysis

|  | **SNP** | **effect allele** | **other allele** | **eaf** | **beta** | **se** | **pval** |
| --- | --- | --- | --- | --- | --- | --- | --- |
| **IA** | rs72841270 | T | G | 0.8649 | 0.1735 | 0.0304 | 1.11E-08 |
|  | rs79780963 | T | C | 0.0784 | -0.2254 | 0.0389 | 6.82E-09 |
|  | rs12310399 | T | C | 0.6464 | -0.1383 | 0.0198 | 3.25E-12 |
|  | rs3742321 | T | C | 0.7644 | -0.1475 | 0.0223 | 4.10E-11 |
|  | rs10519203 | A | G | 0.6291 | -0.1203 | 0.0198 | 1.29E-09 |
|  | rs11646044 | T | G | 0.5538 | -0.149 | 0.0227 | 5.21E-11 |
|  | rs11661542 | A | C | 0.5157 | -0.1659 | 0.0205 | 5.74E-16 |
|  | rs39713 | T | C | 0.088 | 0.1823 | 0.0332 | 4.10E-08 |
|  | rs55965782 | T | C | 0.87 | 0.2644 | 0.0308 | 9.03E-18 |
|  | rs4705938 | T | C | 0.5494 | 0.1198 | 0.0189 | 2.55E-10 |
|  | rs6997005 | A | G | 0.4141 | 0.1489 | 0.0193 | 1.26E-14 |
|  | rs1537373 | T | G | 0.5144 | -0.1864 | 0.0192 | 2.60E-22 |
| **uIA** | rs1537373 | T | G | 0.5123 | -0.1954 | 0.0342 | 1.08E-08 |
| **SAH** | rs12310399 | T | C | 0.6471 | -0.1487 | 0.0236 | 3.19E-10 |
|  | rs11661542 | A | C | 0.5163 | -0.1715 | 0.0235 | 3.18E-13 |
|  | rs6841581 | A | G | 0.1319 | -0.2658 | 0.0351 | 3.52E-14 |
|  | rs62516550 | T | C | 0.3878 | 0.1783 | 0.0268 | 2.94E-11 |
|  | rs1537373 | T | G | 0.5187 | -0.1908 | 0.0227 | 4.84E-17 |

IV, instrumental variables; SNP, single-nucleotide polymorphisms; se, standard error; MR, mendelian randomization; IA, intracranial aneurysm; uIA, unruptured aneurysm; SAH, subarachnoid hemorrhage

**Table S10** The MR-Egger and Cochran’s Q test of IA, uIA, and SAH in reverse MR analyses

| Exposure | outcome | No. SNP | F-statistic |
| --- | --- | --- | --- |
| IA | MCP_1_MCAF | 12 | 50.50 |
| IA | IL_6 | 12 | 50.50 |
| IA | IL_13 | 11 | 51.99 |
| IA | RANTES | 11 | 51.99 |
| IA | IL_1B | 11 | 51.99 |
| IA | SDF_1A | 12 | 50.50 |
| IA | IL_4 | 12 | 50.50 |
| IA | MIF | 11 | 51.99 |
| IA | VEGF | 12 | 50.50 |
| IA | MIP_1A | 11 | 51.99 |
| IA | GROA | 11 | 51.99 |
| IA | IL_8 | 11 | 51.99 |
| IA | IFN_G | 12 | 50.50 |
| IA | FGF_BASIC | 12 | 50.50 |
| IA | TNF_B | 11 | 51.99 |
| IA | MIP_1B | 12 | 50.50 |
| IA | SCGF_B | 11 | 51.99 |
| IA | CTACK | 11 | 51.99 |
| IA | HGF | 12 | 50.50 |
| IA | IL_5 | 11 | 51.99 |
| IA | IP_10 | 11 | 51.99 |
| IA | MIG | 11 | 51.99 |
| IA | EOTAXIN | 12 | 50.50 |
| IA | M_CSF | 11 | 51.99 |
| IA | IL_2RA | 11 | 51.99 |
| IA | SCF | 12 | 50.50 |
| IA | IL_9 | 11 | 51.99 |
| IA | MCP_3 | 11 | 51.99 |
| IA | PDGF_BB | 12 | 50.50 |
| IA | IL_1RA | 11 | 51.99 |
| IA | IL_16 | 11 | 51.99 |
| IA | G_CSF | 12 | 50.50 |
| IA | IL_10 | 12 | 50.50 |
| IA | IL_18 | 11 | 51.99 |
| IA | TNF_A | 11 | 51.99 |
| IA | IL_7 | 11 | 51.99 |
| IA | IL_2 | 11 | 51.99 |
| IA | IL_12_P70 | 12 | 50.50 |
| IA | TRAIL | 12 | 50.50 |
| IA | IL_17 | 12 | 50.50 |
| IA | B_NGF | 11 | 51.99 |
| uIA | MCP_1_MCAF | 1 | 32.64 |
| uIA | IL_6 | 1 | 32.64 |
| uIA | IL_13 | 1 | 32.64 |
| uIA | RANTES | 1 | 32.64 |
| uIA | IL_1B | 1 | 32.64 |
| uIA | SDF_1A | 1 | 32.64 |
| uIA | IL_4 | 1 | 32.64 |
| uIA | MIF | 1 | 32.64 |
| uIA | VEGF | 1 | 32.64 |
| uIA | MIP_1A | 1 | 32.64 |
| uIA | GROA | 1 | 32.64 |
| uIA | IL_8 | 1 | 32.64 |
| uIA | IFN_G | 1 | 32.64 |
| uIA | FGF_BASIC | 1 | 32.64 |
| uIA | TNF_B | 1 | 32.64 |
| uIA | MIP_1B | 1 | 32.64 |
| uIA | SCGF_B | 1 | 32.64 |
| uIA | CTACK | 1 | 32.64 |
| uIA | HGF | 1 | 32.64 |
| uIA | IL_5 | 1 | 32.64 |
| uIA | IP_10 | 1 | 32.64 |
| uIA | MIG | 1 | 32.64 |
| uIA | EOTAXIN | 1 | 32.64 |
| uIA | M_CSF | 1 | 32.64 |
| uIA | IL_2RA | 1 | 32.64 |
| uIA | SCF | 1 | 32.64 |
| uIA | IL_9 | 1 | 32.64 |
| uIA | MCP_3 | 1 | 32.64 |
| uIA | PDGF_BB | 1 | 32.64 |
| uIA | IL_1RA | 1 | 32.64 |
| uIA | IL_16 | 1 | 32.64 |
| uIA | G_CSF | 1 | 32.64 |
| uIA | IL_10 | 1 | 32.64 |
| uIA | IL_18 | 1 | 32.64 |
| uIA | TNF_A | 1 | 32.64 |
| uIA | IL_7 | 1 | 32.64 |
| uIA | IL_2 | 1 | 32.64 |
| uIA | IL_12_P70 | 1 | 32.64 |
| uIA | TRAIL | 1 | 32.64 |
| uIA | IL_17 | 1 | 32.64 |
| uIA | B_NGF | 1 | 32.64 |
| SAH | MCP_1_MCAF | 5 | 53.18 |
| SAH | IL_6 | 5 | 53.18 |
| SAH | IL_13 | 5 | 53.18 |
| SAH | RANTES | 5 | 53.18 |
| SAH | IL_1B | 5 | 53.18 |
| SAH | SDF_1A | 5 | 53.18 |
| SAH | IL_4 | 5 | 53.18 |
| SAH | MIF | 5 | 53.18 |
| SAH | VEGF | 5 | 53.18 |
| SAH | MIP_1A | 5 | 53.18 |
| SAH | GROA | 5 | 53.18 |
| SAH | IL_8 | 5 | 53.18 |
| SAH | IFN_G | 5 | 53.18 |
| SAH | FGF_BASIC | 5 | 53.18 |
| SAH | TNF_B | 5 | 53.18 |
| SAH | MIP_1B | 5 | 53.18 |
| SAH | SCGF_B | 5 | 53.18 |
| SAH | CTACK | 5 | 53.18 |
| SAH | HGF | 5 | 53.18 |
| SAH | IL_5 | 5 | 53.18 |
| SAH | IP_10 | 5 | 53.18 |
| SAH | MIG | 5 | 53.18 |
| SAH | EOTAXIN | 5 | 53.18 |
| SAH | M_CSF | 5 | 53.18 |
| SAH | IL_2RA | 5 | 53.18 |
| SAH | SCF | 5 | 53.18 |
| SAH | IL_9 | 5 | 53.18 |
| SAH | MCP_3 | 5 | 53.18 |
| SAH | PDGF_BB | 5 | 53.18 |
| SAH | IL_1RA | 5 | 53.18 |
| SAH | IL_16 | 5 | 53.18 |
| SAH | G_CSF | 5 | 53.18 |
| SAH | IL_10 | 5 | 53.18 |
| SAH | IL_18 | 5 | 53.18 |
| SAH | TNF_A | 5 | 53.18 |
| SAH | IL_7 | 5 | 53.18 |
| SAH | IL_2 | 5 | 53.18 |
| SAH | IL_12_P70 | 5 | 53.18 |
| SAH | TRAIL | 5 | 53.18 |
| SAH | IL_17 | 5 | 53.18 |
| SAH | B_NGF | 5 | 53.18 |

IA, intracranial aneurysm; uIA, unruptured aneurysm; SAH, subarachnoid hemorrhage; SNP, single-nucleotide polymorphisms; MR, mendelian randomization

**Table S11** The MR results of IA, uIA, and SAH on inflammation cytokines in reverse MR analyses

| Exposure | Outcome | Methods | OR (95%CI) | *P* |
| --- | --- | --- | --- | --- |
| IA | MCP_1_MCAF | IVW | 1.04(0.96,1.12) | 0.2844 |
|  |  | MR-Egger | 1.21(0.83,1.75) | 0.3310 |
|  |  | Weighted median | 1.06(0.97,1.16) | 0.1629 |
|  |  | Maximum likelihood | 1.04(0.97,1.11) | 0.2100 |
|  |  | RAPS | 1.05(0.97,1.13) | 0.2015 |
| IA | IL_6 | IVW | 1.12(1.04,1.20) | 0.0013 |
|  |  | MR-Egger | 1.31(0.93,1.84) | 0.1469 |
|  |  | Weighted median | 1.06(0.96,1.17) | 0.1936 |
|  |  | Maximum likelihood | 1.12(1.05,1.20) | 0.0006 |
|  |  | RAPS | 1.10(1.03,1.18) | 0.0045 |
| IA | IL_13 | IVW | 1.07(0.96,1.19) | 0.2134 |
|  |  | MR-Egger | 1.48(0.89,2.46) | 0.1633 |
|  |  | Weighted median | 1.10(0.96,1.27) | 0.1486 |
|  |  | Maximum likelihood | 1.07(0.97,1.18) | 0.1694 |
|  |  | RAPS | 1.06(0.95,1.18) | 0.2850 |
| IA | RANTES | IVW | 1.04(0.95,1.15) | 0.3419 |
|  |  | MR-Egger | 0.95(0.58,1.57) | 0.8697 |
|  |  | Weighted median | 1.08(0.94,1.23) | 0.2367 |
|  |  | Maximum likelihood | 1.04(0.94,1.16) | 0.3562 |
|  |  | RAPS | 1.06(0.95,1.17) | 0.2655 |
| IA | IL_1B | IVW | 1.09(0.96,1.23) | 0.1553 |
|  |  | MR-Egger | 1.51(0.84,2.69) | 0.1954 |
|  |  | Weighted median | 1.05(0.91,1.22) | 0.4342 |
|  |  | Maximum likelihood | 1.09(0.98,1.21) | 0.0924 |
|  |  | RAPS | 1.09(0.98,1.22) | 0.0901 |
| IA | SDF_1A | IVW | 1.04(0.97,1.11) | 0.2099 |
|  |  | MR-Egger | 0.85(0.61,1.18) | 0.3639 |
|  |  | Weighted median | 1.03(0.94,1.13) | 0.4559 |
|  |  | Maximum likelihood | 1.04(0.97,1.11) | 0.2075 |
|  |  | RAPS | 1.05(0.97,1.12) | 0.1667 |
| IA | IL_4 | IVW | 1.13(1.06,1.20) | 0.0002 |
|  |  | MR-Egger | 1.25(0.90,1.74) | 0.2007 |
|  |  | Weighted median | 1.12(1.02,1.23) | 0.0128 |
|  |  | Maximum likelihood | 1.13(1.06,1.21) | 0.0002 |
|  |  | RAPS | 1.14(1.06,1.22) | 0.0001 |
| IA | MIF | IVW | 1.10(1.03,1.19) | 0.0045 |
|  |  | MR-Egger | 0.86(0.53,1.40) | 0.5730 |
|  |  | Weighted median | 1.10(0.96,1.26) | 0.1430 |
|  |  | Maximum likelihood | 1.11(1.01,1.22) | 0.0405 |
|  |  | RAPS | 1.11(1.01,1.24) | 0.0333 |
| IA | VEGF | IVW | 1.08(1.00,1.16) | 0.0289 |
|  |  | MR-Egger | 1.18(0.81,1.71) | 0.3999 |
|  |  | Weighted median | 1.08(0.98,1.19) | 0.1190 |
|  |  | Maximum likelihood | 1.08(1.01,1.16) | 0.0224 |
|  |  | RAPS | 1.07(0.99,1.16) | 0.0612 |
| IA | MIP_1A | IVW | 1.08(0.98,1.20) | 0.0989 |
|  |  | MR-Egger | 1.53(0.94,2.49) | 0.1174 |
|  |  | Weighted median | 1.10(0.96,1.26) | 0.1610 |
|  |  | Maximum likelihood | 1.09(0.98,1.20) | 0.0895 |
|  |  | RAPS | 1.09(0.97,1.22) | 0.1297 |
| IA | GROA | IVW | 0.99(0.90,1.10) | 0.9753 |
|  |  | MR-Egger | 0.89(0.52,1.50) | 0.6740 |
|  |  | Weighted median | 1.01(0.89,1.16) | 0.7989 |
|  |  | Maximum likelihood | 0.99(0.90,1.10) | 0.9748 |
|  |  | RAPS | 1.01(0.91,1.12) | 0.8314 |
| IA | IL_8 | IVW | 1.10(1.01,1.22) | 0.0485 |
|  |  | MR-Egger | 1.39(0.85,2.28) | 0.2156 |
|  |  | Weighted median | 1.09(0.95,1.25) | 0.1988 |
|  |  | Maximum likelihood | 1.10(1.00,1.22) | 0.0457 |
|  |  | RAPS | 1.10(0.99,1.22) | 0.0665 |
| IA | IFN_G | IVW | 1.13(1.06,1.21) | 0.0001 |
|  |  | MR-Egger | 1.23(0.89,1,72) | 0.2308 |
|  |  | Weighted median | 1.10(1.00,1.21) | 0.0379 |
|  |  | Maximum likelihood | 1.14(1.06,1.22) | 0.0001 |
|  |  | RAPS | 1.14(1.06,1.22) | 0.0003 |
| IA | FGF_BASIC | IVW | 1.06(1.00,1.13) | 0.0307 |
|  |  | MR-Egger | 1.31(0.94,1.82) | 0.1391 |
|  |  | Weighted median | 1.06(0.96,1.16) | 0.2089 |
|  |  | Maximum likelihood | 1.06(0.99,1.14) | 0.0550 |
|  |  | RAPS | 1.05(0.98,1.13) | 0.1144 |
| IA | TNF_B | IVW | 0.99(0.87,1.13) | 0.9652 |
|  |  | MR-Egger | 0.77(0.37,1.56) | 0.4897 |
|  |  | Weighted median | 0.98(0.80,1.19) | 0.8625 |
|  |  | Maximum likelihood | 0.99(0.85,1.15) | 0.9699 |
|  |  | RAPS | 0.99(0.85,1.16) | 0.9691 |
| IA | MIP_1B | IVW | 1.00(0.94,1.06) | 0.9507 |
|  |  | MR-Egger | 1.24(0.90,1.71) | 0.2028 |
|  |  | Weighted median | 1.03(0.94,1.12) | 0.4463 |
|  |  | Maximum likelihood | 1.00(0.93,1.06) | 0.9527 |
|  |  | RAPS | 1.01(0.94,1.08) | 0.7687 |
| IA | SCGF_B | IVW | 0.97(0.89,1.05) | 0.5507 |
|  |  | MR-Egger | 0.89(0.55,1.44) | 0.6628 |
|  |  | Weighted median | 0.97(0.85,1.10) | 0.6885 |
|  |  | Maximum likelihood | 0.97(0.88,1.07) | 0.6076 |
|  |  | RAPS | 0.97(0.88,1.07) | 0.6187 |
| IA | CTACK | IVW | 1.03(0.96,1.10) | 0.3042 |
|  |  | MR-Egger | 0.92(0.57,1.48) | 0.7438 |
|  |  | Weighted median | 1.04(0.91,1.18) | 0.5469 |
|  |  | Maximum likelihood | 1.03(0.93,1,14) | 0.4923 |
|  |  | RAPS | 1.03(0.93,1.14) | 0.5078 |
| IA | HGF | IVW | 1.02(0.95,1.08) | 0.5311 |
|  |  | MR-Egger | 0.93(0.67,1.28) | 0.6717 |
|  |  | Weighted median | 1.04(0.95,1.15) | 0.3090 |
|  |  | Maximum likelihood | 1.02(0.95,1.09) | 0.5247 |
|  |  | RAPS | 1.02(0.95,1.10) | 0.4302 |
| IA | IL_5 | IVW | 1.00(0.89,1.13) | 0.8879 |
|  |  | MR-Egger | 1.23(0.67,2.25) | 0.5059 |
|  |  | Weighted median | 1.03(0.90,1,18) | 0.5885 |
|  |  | Maximum likelihood | 1.00(0.91,1,11) | 0.8689 |
|  |  | RAPS | 1.03(0.93,1.15) | 0.4749 |
| IA | IP_10 | IVW | 0.95(0.86,1.04) | 0.3135 |
|  |  | MR-Egger | 1.22(0.76,1.96) | 0.4288 |
|  |  | Weighted median | 0.96(0.84,1.08) | 0.5309 |
|  |  | Maximum likelihood | 0.95(0.86,1.04) | 0.3167 |
|  |  | RAPS | 0.95(0.86,1.05) | 0.3839 |
| IA | MIG | IVW | 1.01(0.91,1.13) | 0.7382 |
|  |  | MR-Egger | 1.30(0.76,2.22) | 0.3596 |
|  |  | Weighted median | 1.01(0.89,1.16) | 0.8046 |
|  |  | Maximum likelihood | 1.01(0.92,1.12) | 0.7055 |
|  |  | RAPS | 1.02(0.91,1.13) | 0.7075 |
| IA | EOTAXIN | IVW | 1.01(0.97,1.05) | 0.5210 |
|  |  | MR-Egger | 1.02(0.74,1.41) | 0.8779 |
|  |  | Weighted median | 1.02(0.93,1.11) | 0.6253 |
|  |  | Maximum likelihood | 1.01(0.94,1.08) | 0.6715 |
|  |  | RAPS | 1.01(0.94,1.08) | 0.6812 |
| IA | M_CSF | IVW | 0.88(0.76,1.01) | 0.0770 |
|  |  | MR-Egger | 1.08(0.54,2.17) | 0.8219 |
|  |  | Weighted median | 0.89(0.74,1.07) | 0.2228 |
|  |  | Maximum likelihood | 0.88(5.78,0.99) | 0.0396 |
|  |  | RAPS | 0.88(0.75,1.02) | 0.1016 |
| IA | IL_2RA | IVW | 0.95(0.88,1.02) | 0.2068 |
|  |  | MR-Egger | 1.19(0.74,1.92) | 0.4845 |
|  |  | Weighted median | 0.97(0.85,1.11) | 0.7432 |
|  |  | Maximum likelihood | 0.95(0.86,1.05) | 0.3415 |
|  |  | RAPS | 0.95(0.86,1.05) | 0.4028 |
| IA | SCF | IVW | 0.98(0.91,1.05) | 0.6306 |
|  |  | MR-Egger | 0.99(0.69,1.40) | 0.9607 |
|  |  | Weighted median | 1.02(0.93,1.12) | 0.5700 |
|  |  | Maximum likelihood | 0.98(0.92,1.04) | 0.6092 |
|  |  | RAPS | 0.99(0.93,1.06) | 0.9582 |
| IA | IL_9 | IVW | 1.10(1.02,1.20) | 0.0133 |
|  |  | MR-Egger | 1.41(0.87,2.28) | 0.1886 |
|  |  | Weighted median | 1.08(0.94,1.23) | 0.2339 |
|  |  | Maximum likelihood | 1.11(1.00,1.22) | 0.0382 |
|  |  | RAPS | 1.11(1.00,1.23) | 0.0452 |
| IA | MCP_3 | IVW | 1.12(0.92,1.35) | 0.2472 |
|  |  | MR-Egger | 1.06(0.40,2.82) | 0.8950 |
|  |  | Weighted median | 1.12(0.87,1.44) | 0.3734 |
|  |  | Maximum likelihood | 1.12(0.93,1.34) | 0.2029 |
|  |  | RAPS | 1.11(0.91,1.35) | 0.2969 |
| IA | PDGF_BB | IVW | 1.02(0.96,1.09) | 0.3697 |
|  |  | MR-Egger | 1.15(0.84,1.59) | 0.3836 |
|  |  | Weighted median | 1.04(0.95,1.13) | 0.3511 |
|  |  | Maximum likelihood | 1.03(0.96,1.10) | 0.3754 |
|  |  | RAPS | 1.02(0.95,1.09) | 0.4498 |
| IA | IL_1RA | IVW | 1.18(1.05,1.32) | 0.0032 |
|  |  | MR-Egger | 1.78(1.07,2.98) | 0.0534 |
|  |  | Weighted median | 1.16(1.00,1.35) | 0.0461 |
|  |  | Maximum likelihood | 1.18(1.07,1.31) | 0.0006 |
|  |  | RAPS | 1.18(1.04,1.34) | 0.0081 |
| IA | IL_16 | IVW | 0.97(0.88,1.07) | 0.6330 |
|  |  | MR-Egger | 0.65(0.40,1.07) | 0.1258 |
|  |  | Weighted median | 0.98(0.85,1.13) | 0.7965 |
|  |  | Maximum likelihood | 0.97(0.88,1.07) | 0.6281 |
|  |  | RAPS | 0.98(0.88,1.09) | 0.7891 |
| IA | G_CSF | IVW | 1.06(1.01,1.12) | 0.0220 |
|  |  | MR-Egger | 1.15(0.83,1.60) | 0.3896 |
|  |  | Weighted median | 1.05(0.96,1.15) | 0.2538 |
|  |  | Maximum likelihood | 1.06(0.99,1.14) | 0.0614 |
|  |  | RAPS | 1.06(0.99,1.14) | 0.0601 |
| IA | IL_10 | IVW | 1.11(1.04,1.17) | 0.0002 |
|  |  | MR-Egger | 1.27(0.92,1.77) | 0.1730 |
|  |  | Weighted median | 1.10(1.00,1.20) | 0.0369 |
|  |  | Maximum likelihood | 1.11(1.03,1.19) | 0.0022 |
|  |  | RAPS | 1.10(1.03,1.18) | 0.0045 |
| IA | IL_18 | IVW | 0.98(0.92,1.04) | 0.5581 |
|  |  | MR-Egger | 1.12(0.69,1.81) | 0.6379 |
|  |  | Weighted median | 1.00(0.88,1.14) | 0.8944 |
|  |  | Maximum likelihood | 0.98(0.88,1.08) | 0.7092 |
|  |  | RAPS | 0.98(0.88,1.08) | 0.7154 |
| IA | TNF_A | IVW | 1.10(0.97,1.25) | 0.1216 |
|  |  | MR-Egger | 1.54(0.84,2.83) | 0.1952 |
|  |  | Weighted median | 1.13(0.97,1.32) | 0.1009 |
|  |  | Maximum likelihood | 1.10(1.00,1.22) | 0.0493 |
|  |  | RAPS | 1.13(0.99,1.29) | 0.0615 |
| IA | IL_7 | IVW | 1.07(0.95,1.21) | 0.2400 |
|  |  | MR-Egger | 1.38(0.75,2.53) | 0.3248 |
|  |  | Weighted median | 1.12(0.98,1.29) | 0.0881 |
|  |  | Maximum likelihood | 1.07(0.97,1.19) | 0.1484 |
|  |  | RAPS | 1.08(0.97,1.21) | 0.1467 |
| IA | IL_2 | IVW | 1.12(1.00,1.25) | 0.0437 |
|  |  | MR-Egger | 1.15(0.64,2.07) | 0.6336 |
|  |  | Weighted median | 1.11(0.96,1.29) | 0.1325 |
|  |  | Maximum likelihood | 1.12(1.01,1.24) | 0.0202 |
|  |  | RAPS | 1,13(0.99,1.28) | 0.0527 |
| IA | IL_12_P70 | IVW | 1.14(1.05,1.23) | 1.67e-03 |
|  |  | MR-Egger | 1.35(0.90,2.02) | 1.72e-01 |
|  |  | Weighted median | 1.09(0.99,1.20) | 6.58e-02 |
|  |  | Maximum likelihood | 1.14(1.07,1.22) | 7.64e-05 |
|  |  | RAPS | 1.14(1.04,1.24) | 3.70e-03 |
| IA | TRAIL | IVW | 1.01(0.96,1.06) | 0.6088 |
|  |  | MR-Egger | 1.06(0.77,1.45) | 0.7149 |
|  |  | Weighted median | 1.03(0.95,1.13) | 0.3989 |
|  |  | Maximum likelihood | 1.01(0.94,1.08) | 0.7082 |
|  |  | RAPS | 1.01(0.94,1.08) | 0.6515 |
| IA | IL_17 | IVW | 1.04(0.98,1.11) | 0.1462 |
|  |  | MR-Egger | 1.33(0.96,1.85) | 0.1141 |
|  |  | Weighted median | 1.05(0.96,1.16) | 0.2327 |
|  |  | Maximum likelihood | 1.05(0.98,1.12) | 0.1599 |
|  |  | RAPS | 1.05(0.98,1.13) | 0.1368 |
| IA | B_NGF | IVW | 1.02(0.94,1.10) | 0.6222 |
|  |  | MR-Egger | 0.80(0.49,1.30) | 0.4011 |
|  |  | Weighted median | 0.99(0.87,1.13) | 0.9811 |
|  |  | Maximum likelihood | 1.02(0.92,1.12) | 0.6922 |
|  |  | RAPS | 1.01(0.91,1.12) | 0.8397 |
| uIA | MCP_1_MCAF | Wald ratio | 1.12(0.96,1.32) | 0.1402 |
| uIA | IL_6 | Wald ratio | 1.36(1.16,1.60) | 0.0001 |
| uIA | IL_13 | Wald ratio | 1.37(1.08,1.75) | 0.0092 |
| uIA | RANTES | Wald ratio | 0.89(0.69,1.14) | 0.3757 |
| uIA | IL_1B | Wald ratio | 1.06(0.82,1.36) | 0.6455 |
| uIA | SDF_1A | Wald ratio | 1.150(0.97,1.35) | 0.0947 |
| uIA | IL_4 | Wald ratio | 1.22(1.04,1.44) | 0.0134 |
| uIA | MIF | Wald ratio | 0.89(0.70,1.14) | 0.3720 |
| uIA | VEGF | Wald ratio | 1.27(1.07,1.51) | 0.0058 |
| uIA | MIP_1A | Wald ratio | 1.29(1.01,1.65) | 0.0350 |
| uIA | GROA | Wald ratio | 1.09(0.85,1.38) | 0.4779 |
| uIA | IL_8 | Wald ratio | 1.06(0.84,1.36) | 0.5838 |
| uIA | IFN_G | Wald ratio | 1.27(1.07, 1.49) | 0.0045 |
| uIA | FGF_BASIC | Wald ratio | 1.25(1.06,1.48) | 0.0070 |
| uIA | TNF_B | Wald ratio | 1.13(0.79,1.63) | 0.4846 |
| uIA | MIP_1B | Wald ratio | 1.10(0.94,1.29) | 0.2248 |
| uIA | SCGF_B | Wald ratio | 1.10(0.87,1.39) | 0.4110 |
| uIA | CTACK | Wald ratio | 0.93(0.73,1.18) | 0.5936 |
| uIA | HGF | Wald ratio | 1.11(0.95,1.31) | 0.1743 |
| uIA | IL_5 | Wald ratio | 1.16(0.91,1.49) | 0.2150 |
| uIA | IP_10 | Wald ratio | 1.06(0.84,1.34) | 0.6051 |
| uIA | MIG | Wald ratio | 1.06(0.84,1.35) | 0.5788 |
| uIA | EOTAXIN | Wald ratio | 1.04(0.88,1.22) | 0.5975 |
| uIA | M_CSF | Wald ratio | 1.18(0.89,1.58) | 0.2405 |
| uIA | IL_2RA | Wald ratio | 1.05(0.83,1.33) | 0.6748 |
| uIA | SCF | Wald ratio | 1.02(0.87,1.20) | 0.7199 |
| uIA | IL_9 | Wald ratio | 1.29(1.01,1.63) | 0.0356 |
| uIA | MCP_3 | Wald ratio | 1.41(0.92,2.17) | 0.1105 |
| uIA | PDGF_BB | Wald ratio | 1.04(0.89,1.23) | 0.5610 |
| uIA | IL_1RA | Wald ratio | 1.50(1.18,1.91) | 0.0007 |
| uIA | IL_16 | Wald ratio | 1.03(0.80,1.31) | 0.8113 |
| uIA | G_CSF | Wald ratio | 1.17(0.99,1.38) | 0.0548 |
| uIA | IL_10 | Wald ratio | 1.26(1.07,1.49) | 0.0050 |
| uIA | IL_18 | Wald ratio | 1.10(0.87,1.40) | 0.3940 |
| uIA | TNF_A | Wald ratio | 1.29(1.01,1.655) | 0.0377 |
| uIA | IL_7 | Wald ratio | 1.17(0.91,1.50) | 0.2003 |
| uIA | IL_2 | Wald ratio | 1.23(0.96,1.56) | 0.0934 |
| uIA | IL_12_P70 | Wald ratio | 1.28(1.09,1.50) | 0.0025 |
| uIA | TRAIL | Wald ratio | 1.05(0.90,1.24) | 0.4996 |
| uIA | IL_17 | Wald ratio | 1.22(1.03,1.43) | 0.0183 |
| uIA | B_NGF | Wald ratio | 0.99(0.78,1.27) | 0.9901 |
| SAH | MCP_1_MCAF | IVW | 1.05(0.98,1.12) | 0.1050 |
|  |  | MR-Egger | 1.37(0.86,2.19) | 0.2718 |
|  |  | Weighted median | 1.07(0.97,1.17) | 0.1720 |
|  |  | Maximum likelihood | 1.05(0.97,1.14) | 0.1922 |
|  |  | RAPS | 1.05(0.97,1.14) | 0.2069 |
| SAH | IL_6 | IVW | 1.11(0.98,1.25) | 0.0835 |
|  |  | MR-Egger | 1.11(0.49,2.51) | 0.8017 |
|  |  | Weighted median | 1.06(0.94,1.18) | 0.3030 |
|  |  | Maximum likelihood | 1.11(1.02,1.21) | 0.0095 |
|  |  | RAPS | 1.08(0.97,1.21) | 0.1222 |
| SAH | IL_13 | IVW | 1.08(0.90,1.30) | 0.3723 |
|  |  | MR-Egger | 1.90(0.66,5.49) | 0.3182 |
|  |  | Weighted median | 1.12(0.95,1.32) | 0.1736 |
|  |  | Maximum likelihood | 1.09(0.96,1.23) | 0.1698 |
|  |  | RAPS | 1.08(0.90,1.31) | 0.3815 |
| SAH | RANTES | IVW | 0.96(0.84,1.11) | 0.6604 |
|  |  | MR-Egger | 1.50(0.69,3.26) | 0.3730 |
|  |  | Weighted median | 1.05(0.89,1.23) | 0.5190 |
|  |  | Maximum likelihood | 0.96(0.85,1.09) | 0.6236 |
|  |  | RAPS | 0.97(0.85,1.11) | 0.7538 |
| SAH | IL_1B | IVW | 1.00(0.85,1.18) | 0.9444 |
|  |  | MR-Egger | 2.21(1.06,4.59) | 0.1230 |
|  |  | Weighted median | 0.99(0.82,1.18) | 0.9224 |
|  |  | Maximum likelihood | 1.00(0.88,1.14) | 0.9280 |
|  |  | RAPS | 1.00(0.86,1.16) | 0.9495 |
| SAH | SDF_1A | IVW | 1.02(0.94,1.12) | 0.5005 |
|  |  | MR-Egger | 0.82(0.49,1.37) | 0.5138 |
|  |  | Weighted median | 0.99(0.89,1.11) | 0.9618 |
|  |  | Maximum likelihood | 1.03(0.94,1.12) | 0.4869 |
|  |  | RAPS | 1.03(0.94,1.12) | 0.5110 |
| SAH | IL_4 | IVW | 1.13(1.06,1.20) | 7.10e-05 |
|  |  | MR-Egger | 1.21(0.76,1.95) | 4.69e-01 |
|  |  | Weighted median | 1.13(1.01,1.27) | 2.29e-02 |
|  |  | Maximum likelihood | 1.13(1.04,1.23) | 3.29e-03 |
|  |  | RAPS | 1.13(1.04,1.23) | 4.26e-03 |
| SAH | MIF | IVW | 1.04(0.93,1.16) | 0.4274 |
|  |  | MR-Egger | 0.77(0.37,1.58) | 0.5381 |
|  |  | Weighted median | 1.08(0.92,1.25) | 0.3214 |
|  |  | Maximum likelihood | 1.04(0.92,1.18) | 0.4824 |
|  |  | RAPS | 1.04(0.91,1.18) | 0.4978 |
| SAH | VEGF | IVW | 1.10(1.00,1.23) | 0.0488 |
|  |  | MR-Egger | 1.03(0.52,2.04) | 0.9303 |
|  |  | Weighted median | 1.11(0.99,1.25) | 0.0672 |
|  |  | Maximum likelihood | 1.11(1.01,1.21) | 0.0197 |
|  |  | RAPS | 1.10(0.99,1.22) | 0.0626 |
| SAH | MIP_1A | IVW | 1.08(0.93,1.26) | 0.2772 |
|  |  | MR-Egger | 2.02(0.97,4.22) | 0.1566 |
|  |  | Weighted median | 0.99(0.83,1.17) | 0.9124 |
|  |  | Maximum likelihood | 1.09(0.96,1.23) | 0.1705 |
|  |  | RAPS | 1.09(0.93,1.28) | 0.2506 |
| SAH | GROA | IVW | 0.98(0.88,1.09) | 0.7363 |
|  |  | MR-Egger | 1.39(0.68,2.84) | 0.4297 |
|  |  | Weighted median | 1.00(0.86,1.17) | 0.9185 |
|  |  | Maximum likelihood | 0.98(0.86,1.11) | 0.7670 |
|  |  | RAPS | 0.98(0.86,1.11) | 0.7748 |
| SAH | IL_8 | IVW | 1.06(0.93,1.21) | 0.3386 |
|  |  | MR-Egger | 1.98(0.97,4.03) | 0.1540 |
|  |  | Weighted median | 1.07(0.91,1.26) | 0.3557 |
|  |  | Maximum likelihood | 1.06(0.94,1.20) | 0.3063 |
|  |  | RAPS | 1.06(0.93,1.20) | 0.3697 |
| SAH | IFN_G | IVW | 1.12(1.03,1.22) | 0.0055 |
|  |  | MR-Egger | 1.07(0.61,1.87) | 0.8121 |
|  |  | Weighted median | 1.09(0.97,1.22) | 0.1292 |
|  |  | Maximum likelihood | 1.12(1.03,1.22) | 0.0062 |
|  |  | RAPS | 1.12(1.02,1.22) | 0.0093 |
| SAH | FGF_BASIC | IVW | 1.08(0.99,1.19) | 0.0715 |
|  |  | MR-Egger | 1.17(0.64,2.12) | 0.6388 |
|  |  | Weighted median | 1.05(0.94,1.18) | 0.3635 |
|  |  | Maximum likelihood | 1.08(0.99,1.18) | 0.0525 |
|  |  | RAPS | 1.07(0.98,1.17) | 0.1013 |
| SAH | TNF_B | IVW | 0.95(0.84,1.08) | 0.4914 |
|  |  | MR-Egger | 0.87(0.31,2.48) | 0.8229 |
|  |  | Weighted median | 0.97(0.78,1.21) | 0.8189 |
|  |  | Maximum likelihood | 0.95(0.79,1.14) | 0.6338 |
|  |  | RAPS | 0.95(0.79,1.15) | 0.6441 |
| SAH | MIP_1B | IVW | 1.01(0.90,1.13) | 0.8326 |
|  |  | MR-Egger | 1.61(0.91,2.84) | 0.1944 |
|  |  | Weighted median | 1.07(0.96,1.19) | 0.1706 |
|  |  | Maximum likelihood | 1.01(0.93,1.09) | 0.7558 |
|  |  | RAPS | 1.02(0.91,1.14) | 0.6552 |
| SAH | SCGF_B | IVW | 0.95(0.84,1.08) | 0.4948 |
|  |  | MR-Egger | 0.82(0.35,1.90) | 0.6851 |
|  |  | Weighted median | 0.98(0.84,1.14) | 0.8729 |
|  |  | Maximum likelihood | 0.95(0.84,1.07) | 0.4662 |
|  |  | RAPS | 0.95(0.84,1.08) | 0.4822 |
| SAH | CTACK | IVW | 0.98(0.90,1.05) | 0.6179 |
|  |  | MR-Egger | 1.07(0.53,2.16) | 0.8532 |
|  |  | Weighted median | 0.99(0.86,1.13) | 0.8979 |
|  |  | Maximum likelihood | 0.98(0.86,1.10) | 0.7536 |
|  |  | RAPS | 0.98(0.86,1.11) | 0.7605 |
| SAH | HGF | IVW | 1.04(0.99,1.09) | 0.0637 |
|  |  | MR-Egger | 0.89(0.56,1.42) | 0.6758 |
|  |  | Weighted median | 1.04(0.95,1.15) | 0.3521 |
|  |  | Maximum likelihood | 1.04(0.96,1.13) | 0.2743 |
|  |  | RAPS | 1.04(0.96,1.13) | 0.2880 |
| SAH | IL_5 | IVW | 0.94(0.79,1.13) | 0.5516 |
|  |  | MR-Egger | 1.33(0.42,4.15) | 0.6565 |
|  |  | Weighted median | 0.98(0.83,1.15) | 0.8321 |
|  |  | Maximum likelihood | 0.94(0.83,1.07) | 0.3843 |
|  |  | RAPS | 0.96(0.81,1.14) | 0.6715 |
| SAH | IP_10 | IVW | 0.96(0.88,1.05) | 0.4617 |
|  |  | MR-Egger | 1.37(0.68,2.76) | 0.4319 |
|  |  | Weighted median | 0.97(0.84,1.11) | 0.7080 |
|  |  | Maximum likelihood | 0.96(0.85,1.08) | 0.5692 |
|  |  | RAPS | 0.96(0.88,1.05) | 0.5812 |
| SAH | MIG | IVW | 1.02(0.97,1.08) | 0.3397 |
|  |  | MR-Egger | 1.40(0.69,2.80) | 0.4114 |
|  |  | Weighted median | 1.01(0.88,1.17) | 0.8070 |
|  |  | Maximum likelihood | 1.02(0.91,1.15) | 0.6473 |
|  |  | RAPS | 1.02(0.90,1.16) | 0.6563 |
| SAH | EOTAXIN | IVW | 1.00(0.97,1.03) | 0.7995 |
|  |  | MR-Egger | 0.97(0.60,1.56) | 0.9240 |
|  |  | Weighted median | 0.99(0.90,1.09) | 0.9051 |
|  |  | Maximum likelihood | 1.00(0.92,1.08) | 0.9254 |
|  |  | RAPS | 1.00(0.92,1.09) | 0.9273 |
| SAH | M_CSF | IVW | 0.90(0.74,1.09) | 0.2971 |
|  |  | MR-Egger | 0.72(0.20,2.57) | 0.6481 |
|  |  | Weighted median | 0.81(0.65,1.00) | 0.0554 |
|  |  | Maximum likelihood | 0.89(0.77,1.04) | 0.1607 |
|  |  | RAPS | 0.89(0.73,1.09) | 0.2693 |
| SAH | IL_2RA | IVW | 0.96(0.88,1.04) | 0.3775 |
|  |  | MR-Egger | 1.06(0.52,2.13) | 0.8772 |
|  |  | Weighted median | 0.98(0.85,1.14) | 0.8498 |
|  |  | Maximum likelihood | 0.96(0.85,1.08) | 0.5446 |
|  |  | RAPS | 0.96(0.85,1.09) | 0.5575 |
| SAH | SCF | IVW | 0.99(0.93,1.05) | 0.8088 |
|  |  | MR-Egger | 0.74(0.47,1.19) | 0.3130 |
|  |  | Weighted median | 1.01(0.91,1.11) | 0.8043 |
|  |  | Maximum likelihood | 0.99(0.91,1.07) | 0.8461 |
|  |  | RAPS | 0.99(0.91,1.07) | 0.8511 |
| SAH | IL_9 | IVW | 1.07(0.94,1.22) | 0.2782 |
|  |  | MR-Egger | 1.65(0.79,3.43) | 0.2679 |
|  |  | Weighted median | 1.06(0.90,1.25) | 0.4405 |
|  |  | Maximum likelihood | 1.07(0.95,1.21) | 0.2336 |
|  |  | RAPS | 1.07(0.93,1.24) | 0.2953 |
| SAH | MCP_3 | IVW | 0.99(0.76,1.28) | 0.9597 |
|  |  | MR-Egger | 0.78(0.14,4.12) | 0.7929 |
|  |  | Weighted median | 0.88(0.65,1.19) | 0.4297 |
|  |  | Maximum likelihood | 0.99(0.79,1.23) | 0.9515 |
|  |  | RAPS | 0.98(0.75,1.29) | 0.9378 |
| SAH | PDGF_BB | IVW | 1.03(0.95,1.12) | 0.3537 |
|  |  | MR-Egger | 1.01(0.58,1.74) | 0.9687 |
|  |  | Weighted median | 1.03(0.92,1,14) | 0.5506 |
|  |  | Maximum likelihood | 1.04(0.95,1.12) | 0.3455 |
|  |  | RAPS | 1.03(0.95,1.13) | 0.4198 |
| SAH | IL_1RA | IVW | 1.13(0.93,1.38) | 0.2036 |
|  |  | MR-Egger | 2.12(0.70,6.42) | 0.2742 |
|  |  | Weighted median | 0.98(0.82,1.17) | 0.8426 |
|  |  | Maximum likelihood | 1,14(1.00,1.29) | 0.0358 |
|  |  | RAPS | 1.14(0.94,1.40) | 0.1755 |
| SAH | IL_16 | IVW | 0.95(0.82,1.09) | 0.4968 |
|  |  | MR-Egger | 0.58(0.27,1.25) | 0.2617 |
|  |  | Weighted median | 0.94(0.79,1.10) | 0.4667 |
|  |  | Maximum likelihood | 0.95(0.83,1.07) | 0.4347 |
|  |  | RAPS | 0.95(0.83,1.08) | 0.4631 |
| SAH | G_CSF | IVW | 1.05(0.99,1.12) | 0.0933 |
|  |  | MR-Egger | 1.16(0.72,1.86) | 0.5790 |
|  |  | Weighted median | 1.03(0.93,1.14) | 0.5425 |
|  |  | Maximum likelihood | 1.05(0.97,1.14) | 0.1945 |
|  |  | RAPS | 1.05(0.96,1.15) | 0.2082 |
| SAH | IL_10 | IVW | 1.11(1.02,1.20) | 0.0083 |
|  |  | MR-Egger | 1.24(0.74,2.06) | 0.4642 |
|  |  | Weighted median | 1.07(0.96,1.19) | 0.1887 |
|  |  | Maximum likelihood | 1.11(1.02,1.21) | 0.0139 |
|  |  | RAPS | 1.10(1.01,1.20) | 0.0244 |
| SAH | IL_18 | IVW | 1.01(0.94,1.09) | 0.7188 |
|  |  | MR-Egger | 1.04(0.52,2.11) | 0.9004 |
|  |  | Weighted median | 1.00(0.87,1.16) | 0.9146 |
|  |  | Maximum likelihood | 1.01(0.89,1.14) | 0.8279 |
|  |  | RAPS | 1.01(0.89,1.14) | 0.8327 |
| SAH | TNF_A | IVW | 1.04(0.85,1.27) | 0.6799 |
|  |  | MR-Egger | 2.16(0.75,6.18) | 0.2456 |
|  |  | Weighted median | 1.02(0.85,1.23) | 0.7736 |
|  |  | Maximum likelihood | 1.04(0.92,1.18) | 0.4940 |
|  |  | RAPS | 1.06(0.86,1.30) | 0.5548 |
| SAH | IL_7 | IVW | 1.02(0.87,1.21) | 0.7291 |
|  |  | MR-Egger | 1.63(0.61,4.36) | 0.4009 |
|  |  | Weighted median | 1.09(0.93,1.27) | 0.2552 |
|  |  | Maximum likelihood | 1.03(0.90,1.16) | 0.6400 |
|  |  | RAPS | 1.04(0.89,1.21) | 0.5730 |
| SAH | IL_2 | IVW | 1.01(0.88,1.16) | 0.8399 |
|  |  | MR-Egger | 1.50(0.67,3.34) | 0.3923 |
|  |  | Weighted median | 0.97(0.83,1.14) | 0.7537 |
|  |  | Maximum likelihood | 1.01(0.89,1.14) | 0.8206 |
|  |  | RAPS | 1.01(0.87,1.17) | 0.8277 |
| SAH | IL_12_P70 | IVW | 1.15(1.03,1.29) | 0.0082 |
|  |  | MR-Egger | 1.35(0.67,2.73) | 0.4546 |
|  |  | Weighted median | 1.17(1.04,1.31) | 0.0058 |
|  |  | Maximum likelihood | 1.16(1.06,1.26) | 0.0004 |
|  |  | RAPS | 1.15(1.03,1.29) | 0.0110 |
| SAH | TRAIL | IVW | 1.03(0.98,1.07) | 0.1537 |
|  |  | MR-Egger | 1.18(0.73,1.88) | 0.5380 |
|  |  | Weighted median | 1.04(0.95,1.14) | 0.3233 |
|  |  | Maximum likelihood | 1.03(0.95,1.12) | 0.4408 |
|  |  | RAPS | 1.03(0.94,1.12) | 0.4540 |
| SAH | IL_17 | IVW | 1.08(1.00,1.17) | 0.0464 |
|  |  | MR-Egger | 1.23(0.75,2.03) | 0.4645 |
|  |  | Weighted median | 1.07(0.96,1.19) | 0.2172 |
|  |  | Maximum likelihood | 1.08(0.99,1.18) | 0.0625 |
|  |  | RAPS | 1.08(0.99,1.18) | 0.0783 |
| SAH | B_NGF | IVW | 0.97(0.92,1.02) | 0.3852 |
|  |  | MR-Egger | 0.76(0.37,1.54) | 0.5044 |
|  |  | Weighted median | 0.99(0.85,1.14) | 0.8999 |
|  |  | Maximum likelihood | 0.97(0.86,1.10) | 0.7129 |
|  |  | RAPS | 0.97(0.86,1.10) | 0.7204 |

IA, intracranial aneurysm; uIA, unruptured aneurysm; SAH, subarachnoid hemorrhage; OR, odds ratio; CI, confidence interval; IVW, inverse variance weighting; RAPS, robust adjusted profile score; MR, mendelian randomization

**Table S12** The MR-Egger and Cochran’s test results of IA, uIA, and SAH on forty-one inflammation cytokines in the reverse MR analysis

| Exposure | Outcome | Methods | Egger_intercept | P-Egger_intercept | Cochran’s | Cochran’s *P* |
| --- | --- | --- | --- | --- | --- | --- |
| IA | IL_6 | IVW |  |  | 12.51 | 0.3263 |
|  |  | MR-Egger | -0.0260 | 0.3720 | 11.50 | 0.3193 |
| IA | IL_4 | IVW |  |  | 10.92 | 0.4492 |
|  |  | MR-Egger | -0.0172 | 0.5351 | 10.49 | 0.3980 |
| IA | MIF | IVW |  |  | 5.14 | 0.8811 |
|  |  | MR-Egger | 0.0407 | 0.3327 | 4.09 | 0.9048 |
| IA | VEGF | IVW |  |  | 12.00 | 0.3635 |
|  |  | MR-Egger | -0.0138 | 0.6580 | 11.75 | 0.3016 |
| IA | IL_8 | IVW |  |  | 10.16 | 0.4263 |
|  |  | MR-Egger | -0.0380 | 0.3671 | 9.23 | 0.4156 |
| IA | IFN_G | IVW |  |  | 10.22 | 0.5103 |
|  |  | MR-Egger | -0.0139 | 0.6142 | 9.95 | 0.4445 |
| IA | FGF_BASIC | IVW |  |  | 8.50 | 0.6670 |
|  |  | MR-Egger | -0.0335 | 0.2423 | 6.96 | 0.7287 |
| IA | IL_9 | IVW |  |  | 6.86 | 0.7378 |
|  |  | MR-Egger | -0.0399 | 0.3328 | 5.82 | 0.7577 |
| IA | IL_1RA | IVW |  |  | 13.32 | 0.2060 |
|  |  | MR-Egger | -0.0672 | 0.1430 | 10.36 | 0.3220 |
| IA | G_CSF | IVW |  |  | 7.19 | 0.7829 |
|  |  | MR-Egger | -0.0140 | 0.6078 | 6.91 | 0.7333 |
| IA | IL_10 | IVW |  |  | 7.80 | 0.7302 |
|  |  | MR-Egger | -0.0230 | 0.4103 | 7.07 | 0.7187 |
| IA | IL_2 | IVW |  |  | 12.81 | 0.2343 |
|  |  | MR-Egger | -0.0049 | 0.9199 | 12.79 | 0.1720 |
| IA | IL_12_P70 | IVW |  |  | 17.26 | 0.1002 |
|  |  | MR-Egger | -0.0279 | 0.4155 | 16.10 | 0.0966 |
| SAH | IL_4 | IVW |  |  | 2.24 | 0.6899 |
|  |  | MR-Egger | -0.0139 | 0.7753 | 2.15 | 0.5414 |
| SAH | VEGF | IVW |  |  | 5.61 | 0.2297 |
|  |  | MR-Egger | 0.0134 | 0.8492 | 5.53 | 0.1364 |
| SAH | IFN_G | IVW |  |  | 3.96 | 0.4102 |
|  |  | MR-Egger | 0.0085 | 0.8819 | 3.93 | 0.2686 |
| SAH | IP_10 | IVW |  |  | 2.37 | 0.6672 |
|  |  | MR-Egger | -0.0674 | 0.3833 | 1.33 | 0.7204 |
| SAH | IL_12_P70 | IVW |  |  | 7.19 | 0.1257 |
|  |  | MR-Egger | -0.0300 | 0.6818 | 6.74 | 0.0806 |
| SAH | IL_17 | IVW |  |  | 3.50 | 0.4765 |
|  |  | MR-Egger | -0.0252 | 0.6322 | 3.20 | 0.3607 |

IA, intracranial aneurysm; uIA, unruptured aneurysm; SAH, subarachnoid hemorrhage; MR, mendelian randomization; IVW, inverse variance weighting;

**Table S13** The MR-Egger and Cochran’s Q test of IA, uIA, and SAH in FinnGen datasets in replicate reverse MR analyses

| Exposure | outcome | No. SNP | F-statistic |
| --- | --- | --- | --- |
| IA-finngen | MCP_1_MCAF | 2 | 35.49 |
| IA-finngen | IL_6 | 2 | 35.49 |
| IA-finngen | IL_13 | 2 | 35.49 |
| IA-finngen | RANTES | 2 | 35.49 |
| IA-finngen | IL_1B | 2 | 35.49 |
| IA-finngen | SDF_1A | 2 | 35.49 |
| IA-finngen | IL_4 | 2 | 35.49 |
| IA-finngen | MIF | 2 | 35.49 |
| IA-finngen | VEGF | 2 | 35.49 |
| IA-finngen | MIP_1A | 2 | 35.49 |
| IA-finngen | GROA | 2 | 35.49 |
| IA-finngen | IL_8 | 2 | 35.49 |
| IA-finngen | IFN_G | 2 | 35.49 |
| IA-finngen | FGF_BASIC | 2 | 35.49 |
| IA-finngen | TNF_B | 2 | 35.49 |
| IA-finngen | MIP_1B | 2 | 35.49 |
| IA-finngen | SCGF_B | 2 | 35.49 |
| IA-finngen | CTACK | 2 | 35.49 |
| IA-finngen | HGF | 2 | 35.49 |
| IA-finngen | IL_5 | 2 | 35.49 |
| IA-finngen | IP_10 | 2 | 35.49 |
| IA-finngen | MIG | 2 | 35.49 |
| IA-finngen | EOTAXIN | 2 | 35.49 |
| IA-finngen | M_CSF | 2 | 35.49 |
| IA-finngen | IL_2RA | 2 | 35.49 |
| IA-finngen | SCF | 2 | 35.49 |
| IA-finngen | IL_9 | 2 | 35.49 |
| IA-finngen | MCP_3 | 2 | 35.49 |
| IA-finngen | PDGF_BB | 2 | 35.49 |
| IA-finngen | IL_1RA | 2 | 35.49 |
| IA-finngen | IL_16 | 2 | 35.49 |
| IA-finngen | G_CSF | 2 | 35.49 |
| IA-finngen | IL_10 | 2 | 35.49 |
| IA-finngen | IL_18 | 2 | 35.49 |
| IA-finngen | TNF_A | 2 | 35.49 |
| IA-finngen | IL_7 | 2 | 35.49 |
| IA-finngen | IL_2 | 2 | 35.49 |
| IA-finngen | IL_12_P70 | 2 | 35.49 |
| IA-finngen | TRAIL | 2 | 35.49 |
| IA-finngen | IL_17 | 2 | 35.49 |
| IA-finngen | B_NGF | 2 | 35.49 |
| uIA-finngen | MCP_1_MCAF | 2 | 30.92 |
| uIA-finngen | IL_6 | 2 | 30.92 |
| uIA-finngen | IL_13 | 2 | 30.92 |
| uIA-finngen | RANTES | 2 | 30.92 |
| uIA-finngen | IL_1B | 2 | 30.92 |
| uIA-finngen | SDF_1A | 2 | 30.92 |
| uIA-finngen | IL_4 | 2 | 30.92 |
| uIA-finngen | MIF | 2 | 30.92 |
| uIA-finngen | VEGF | 2 | 30.92 |
| uIA-finngen | MIP_1A | 2 | 30.92 |
| uIA-finngen | GROA | 2 | 30.92 |
| uIA-finngen | IL_8 | 2 | 30.92 |
| uIA-finngen | IFN_G | 2 | 30.92 |
| uIA-finngen | FGF_BASIC | 2 | 30.92 |
| uIA-finngen | TNF_B | 2 | 30.92 |
| uIA-finngen | MIP_1B | 2 | 30.92 |
| uIA-finngen | SCGF_B | 2 | 30.92 |
| uIA-finngen | CTACK | 2 | 30.92 |
| uIA-finngen | HGF | 2 | 30.92 |
| uIA-finngen | IL_5 | 2 | 30.92 |
| uIA-finngen | IP_10 | 2 | 30.92 |
| uIA-finngen | MIG | 2 | 30.92 |
| uIA-finngen | EOTAXIN | 2 | 30.92 |
| uIA-finngen | M_CSF | 2 | 30.92 |
| uIA-finngen | IL_2RA | 2 | 30.92 |
| uIA-finngen | SCF | 2 | 30.92 |
| uIA-finngen | IL_9 | 2 | 30.92 |
| uIA-finngen | MCP_3 | 2 | 30.92 |
| uIA-finngen | PDGF_BB | 2 | 30.92 |
| uIA-finngen | IL_1RA | 2 | 30.92 |
| uIA-finngen | IL_16 | 2 | 30.92 |
| uIA-finngen | G_CSF | 2 | 30.92 |
| uIA-finngen | IL_10 | 2 | 30.92 |
| uIA-finngen | IL_18 | 2 | 30.92 |
| uIA-finngen | TNF_A | 2 | 30.92 |
| uIA-finngen | IL_7 | 2 | 30.92 |
| uIA-finngen | IL_2 | 2 | 30.92 |
| uIA-finngen | IL_12_P70 | 2 | 30.92 |
| uIA-finngen | TRAIL | 2 | 30.92 |
| uIA-finngen | IL_17 | 2 | 30.92 |
| uIA-finngen | B_NGF | 2 | 30.92 |
| SAH-finngen | MCP_1_MCAF | 4 | 37.56 |
| SAH-finngen | IL_6 | 4 | 37.56 |
| SAH-finngen | IL_13 | 4 | 37.56 |
| SAH-finngen | RANTES | 4 | 37.56 |
| SAH-finngen | IL_1B | 4 | 37.56 |
| SAH-finngen | SDF_1A | 4 | 37.56 |
| SAH-finngen | IL_4 | 4 | 37.56 |
| SAH-finngen | MIF | 4 | 37.56 |
| SAH-finngen | VEGF | 4 | 37.56 |
| SAH-finngen | MIP_1A | 4 | 37.56 |
| SAH-finngen | GROA | 4 | 37.56 |
| SAH-finngen | IL_8 | 4 | 37.56 |
| SAH-finngen | IFN_G | 4 | 37.56 |
| SAH-finngen | FGF_BASIC | 4 | 37.56 |
| SAH-finngen | TNF_B | 4 | 37.56 |
| SAH-finngen | MIP_1B | 4 | 37.56 |
| SAH-finngen | SCGF_B | 4 | 37.56 |
| SAH-finngen | CTACK | 4 | 37.56 |
| SAH-finngen | HGF | 4 | 37.56 |
| SAH-finngen | IL_5 | 4 | 37.56 |
| SAH-finngen | IP_10 | 4 | 37.56 |
| SAH-finngen | MIG | 4 | 37.56 |
| SAH-finngen | EOTAXIN | 4 | 37.56 |
| SAH-finngen | M_CSF | 4 | 37.56 |
| SAH-finngen | IL_2RA | 4 | 37.56 |
| SAH-finngen | SCF | 4 | 37.56 |
| SAH-finngen | IL_9 | 4 | 37.56 |
| SAH-finngen | MCP_3 | 4 | 37.56 |
| SAH-finngen | PDGF_BB | 4 | 37.56 |
| SAH-finngen | IL_1RA | 4 | 37.56 |
| SAH-finngen | IL_16 | 4 | 37.56 |
| SAH-finngen | G_CSF | 4 | 37.56 |
| SAH-finngen | IL_10 | 4 | 37.56 |
| SAH-finngen | IL_18 | 4 | 37.56 |
| SAH-finngen | TNF_A | 4 | 37.56 |
| SAH-finngen | IL_7 | 4 | 37.56 |
| SAH-finngen | IL_2 | 4 | 37.56 |
| SAH-finngen | IL_12_P70 | 4 | 37.56 |
| SAH-finngen | TRAIL | 4 | 37.56 |
| SAH-finngen | IL_17 | 4 | 37.56 |
| SAH-finngen | B_NGF | 4 | 37.56 |

IA, intracranial aneurysm; uIA, unruptured aneurysm; SAH, subarachnoid hemorrhage; MR, mendelian randomization; SNP, single-nucleotide polymorphisms

**Table S14** The MR results of IA, uIA, and SAH on inflammation cytokines in FinnGen datasets in replicate reverse MR analyses

| Exposure | Outcome | Methods | OR (95%) | *P* |
| --- | --- | --- | --- | --- |
| IA-finngen | MCP_1_MCAF | IVW | 1.02(0.93,1.12) | 0.6064 |
|  |  | Maximum likelihood | 1.02(0.94,1.11) | 0.5454 |
|  |  | RAPS | 1.02(0.94,1.11) | 0.5603 |
| IA-finngen | IL_6 | IVW | 1.07(0.81,1.41) | 0.6021 |
|  |  | Maximum likelihood | 1.09(0.99,1.19) | 0.0640 |
|  |  | RAPS | 1.05(0.84,1.32) | 0.6327 |
| IA-finngen | IL_13 | IVW | 1.04(0.73,1.47) | 0.8167 |
|  |  | Maximum likelihood | 1.04(0.92,1.19) | 0.4796 |
|  |  | RAPS | 1.01(0.76,1.35) | 0.9043 |
| IA-finngen | RANTES | IVW | 1.01(0.85,1.21) | 0.8372 |
|  |  | Maximum likelihood | 1.01(0.89,1.15) | 0.7698 |
|  |  | RAPS | 1.02(0.88,1.17) | 0.7723 |
| IA-finngen | IL_1B | IVW | 1.03(1.01,1.06) | 0.0129 |
|  |  | Maximum likelihood | 1.03(0.91,1.17) | 0.5810 |
|  |  | RAPS | 1.03(0.91,1.17) | 0.5906 |
| IA-finngen | SDF_1A | IVW | 1.01(0.84,1.20) | 0.8986 |
|  |  | Maximum likelihood | 1.01(0.92,1.10) | 0.7779 |
|  |  | RAPS | 1.00(0.86,1.16) | 0.9769 |
| IA-finngen | IL_4 | IVW | 1.08(0.96,1.22) | 0.1625 |
|  |  | Maximum likelihood | 1.09(1.00,1.18) | 0.0429 |
|  |  | RAPS | 1.08(0.98,1.20) | 0.0858 |
| IA-finngen | MIF | IVW | 1.03(0.80,1.32) | 0.8028 |
|  |  | Maximum likelihood | 1.03(0.91,1.17) | 0.5978 |
|  |  | RAPS | 1.04(0.84,1.29) | 0.6618 |
| IA-finngen | VEGF | IVW | 1.07(0.87,1.32) | 0.4847 |
|  |  | Maximum likelihood | 1.08(0.98,1.18) | 0.0941 |
|  |  | RAPS | 1.06(0.89,1.26) | 0.4652 |
| IA-finngen | MIP_1A | IVW | 1.08(0.88,1.34) | 0.4385 |
|  |  | Maximum likelihood | 1.09(0.96,1.23) | 0.1768 |
|  |  | RAPS | 1.08(0.90,1.28) | 0.3825 |
| IA-finngen | GROA | IVW | 0.99(0.82,1.20) | 0.9477 |
|  |  | Maximum likelihood | 0.99(0.87,1.12) | 0.9178 |
|  |  | RAPS | 0.98(0.84,1.15) | 0.8830 |
| IA-finngen | IL_8 | IVW | 1.01(0.92,1.10) | 0.8060 |
|  |  | Maximum likelihood | 1.01(0.89,1.14) | 0.8556 |
|  |  | RAPS | 1.01(0.89,1.14) | 0.8599 |
| IA-finngen | IFN_G | IVW | 1.11(0.98,1.25) | 0.0910 |
|  |  | Maximum likelihood | 1.11(1.02,1.21) | 0.0159 |
|  |  | RAPS | 1.11(1.00,1.22) | 0.0358 |
| IA-finngen | FGF_BASIC | IVW | 1.08(0.91,1.28) | 0.3398 |
|  |  | Maximum likelihood | 1.09(0.99,1.19) | 0.0581 |
|  |  | RAPS | 1.07(0.93,1.23) | 0.2816 |
| IA-finngen | TNF_B | IVW | 1.02(0.88,1.19) | 0.7214 |
|  |  | Maximum likelihood | 1.02(0.85,1.23) | 0.7695 |
|  |  | RAPS | 1.02(0.84,1.24) | 0.7765 |
| IA-finngen | MIP_1B | IVW | 1.02(0.92,1.14) | 0.6157 |
|  |  | Maximum likelihood | 1.02(0.94,1.11) | 0.5059 |
|  |  | RAPS | 1.02(0.94,1.12) | 0.5323 |
| IA-finngen | SCGF_B | IVW | 1.09(0.99,1.19) | 0.0596 |
|  |  | Maximum likelihood | 1.09(0.96,1.23) | 0.1579 |
|  |  | RAPS | 1.09(0.96,1.23) | 0.1723 |
| IA-finngen | CTACK | IVW | 0.97(0.92,1.02) | 0.2920 |
|  |  | Maximum likelihood | 0.97(0.86,1.09) | 0.6709 |
|  |  | RAPS | 0.97(0.86,1.10) | 0.6791 |
| IA-finngen | HGF | IVW | 1.02(0.90,1.14) | 0.7172 |
|  |  | Maximum likelihood | 1.02(0.94,1.10) | 0.5947 |
|  |  | RAPS | 1.02(0.92,1.12) | 0.6823 |
| IA-finngen | IL_5 | IVW | 0.97(0.74,1.29) | 0.8819 |
|  |  | Maximum likelihood | 0.97(0.85,1.11) | 0.7323 |
|  |  | RAPS | 0.96(0.76,1.21) | 0.7505 |
| IA-finngen | IP_10 | IVW | 1.02(0.99,1.06) | 0.0855 |
|  |  | Maximum likelihood | 1.02(0.91,1.15) | 0.6449 |
|  |  | RAPS | 1.02(0.90,1.16) | 0.6536 |
| IA-finngen | MIG | IVW | 1.01(0.97,1.05) | 0.4937 |
|  |  | Maximum likelihood | 1.01(0.90,1.14) | 0.8064 |
|  |  | RAPS | 1.01(0.89,1.14) | 0.8114 |
| IA-finngen | EOTAXIN | IVW | 0.99(0.90,1.09) | 0.9151 |
|  |  | Maximum likelihood | 0.99(0.91,1.07) | 0.8993 |
|  |  | RAPS | 0.99(0.91,1.08) | 0.9032 |
| IA-finngen | M_CSF | IVW | 1.02(0.84,1.25) | 0.7778 |
|  |  | Maximum likelihood | 1.02(0.88,1.19) | 0.6948 |
|  |  | RAPS | 1.02(0.87,1.20) | 0.7429 |
| IA-finngen | IL_2RA | IVW | 0.97(0.83,1.14) | 0.7918 |
|  |  | Maximum likelihood | 0.97(0.86,1.10) | 0.7210 |
|  |  | RAPS | 0.97(0.85,1.11) | 0.7309 |
| IA-finngen | SCF | IVW | 0.95(0.84,1.08) | 0.5173 |
|  |  | Maximum likelihood | 0.95(0.88,1.03) | 0.3060 |
|  |  | RAPS | 0.95(0.86,1.05) | 0.3888 |
| IA-finngen | IL_9 | IVW | 1.10(0.97,1.26) | 0.1158 |
|  |  | Maximum likelihood | 1.11(0.98,1.25) | 0.0973 |
|  |  | RAPS | 1.11(0.97,1.26) | 0.1082 |
| IA-finngen | MCP_3 | IVW | 1.01(0.56,1.81) | 0.9554 |
|  |  | Maximum likelihood | 1.01(0.80,1.28) | 0.8761 |
|  |  | RAPS | 0.96(0.59,1.57) | 0.9006 |
| IA-finngen | PDGF_BB | IVW | 1.00(0.92,1.09) | 0.9515 |
|  |  | Maximum likelihood | 1.00(0.92,1.08) | 0.9487 |
|  |  | RAPS | 1.00(0.92,1.09) | 0.9506 |
| IA-finngen | IL_1RA | IVW | 1.10(0.76,1.60) | 0.5915 |
|  |  | Maximum likelihood | 1.12(0.98,1.28) | 0.0832 |
|  |  | RAPS | 1.08(0.79,1.46) | 0.6220 |
| IA-finngen | IL_16 | IVW | 0.98(0.90,1.06) | 0.7020 |
|  |  | Maximum likelihood | 0.98(0.87,1.11) | 0.8016 |
|  |  | RAPS | 0.98(0.86,1.11) | 0.8075 |
| IA-finngen | G_CSF | IVW | 1.07(0.96,1.20) | 0.1974 |
|  |  | Maximum likelihood | 1.07(0.98,1.17) | 0.0854 |
|  |  | RAPS | 1.07(0.98,1.17) | 0.1056 |
| IA-finngen | IL_10 | IVW | 1.05(0.85,1.30) | 0.6250 |
|  |  | Maximum likelihood | 1.06(0.97,1.15) | 0.1945 |
|  |  | RAPS | 1.04(0.87,1.24) | 0.6439 |
| IA-finngen | IL_18 | IVW | 1.03(0.93,1.13) | 0.5139 |
|  |  | Maximum likelihood | 1.03(0.91,1.16) | 0.5991 |
|  |  | RAPS | 1.03(0.91,1.17) | 0.6103 |
| IA-finngen | TNF_A | IVW | 1.08(0.87,1.34) | 0.4794 |
|  |  | Maximum likelihood | 1.08(0.95,1.23) | 0.2104 |
|  |  | RAPS | 1.07(0.89,1.28) | 0.4315 |
| IA-finngen | IL_7 | IVW | 1.01(0.83,1.22) | 0.8820 |
|  |  | Maximum likelihood | 1.01(0.89,1.15) | 0.8182 |
|  |  | RAPS | 1.01(0.86,1.18) | 0.8993 |
| IA-finngen | IL_2 | IVW | 1.08(0.95,1.23) | 0.2237 |
|  |  | Maximum likelihood | 1.08(0.95,1.23) | 0.1900 |
|  |  | RAPS | 1.08(0.95,1.23) | 0.2048 |
| IA-finngen | IL_12_P70 | IVW | 1.05(0.82,1.35) | 0.6673 |
|  |  | Maximum likelihood | 1.06(0.97,1.16) | 0.1549 |
|  |  | RAPS | 1.03(0.84,1.28) | 0.7135 |
| IA-finngen | TRAIL | IVW | 0.98(0.88,1.08) | 0.7135 |
|  |  | Maximum likelihood | 0.98(0.90,1.06) | 0.6273 |
|  |  | RAPS | 0.97(0.89,1.06) | 0.6435 |
| IA-finngen | IL_17 | IVW | 1.04(0.85,1.28) | 0.6769 |
|  |  | Maximum likelihood | 1.04(0.96,1.14) | 0.2868 |
|  |  | RAPS | 1.03(0.87,1.22) | 0.7111 |
| IA-finngen | B_NGF | IVW | 0.96(0.91,1.02) | 0.2886 |
|  |  | Maximum likelihood | 0.96(0.85,1.09) | 0.6086 |
|  |  | RAPS | 0.96(0.85,1.09) | 0.6188 |
| uIA-finngen | MCP_1_MCAF | IVW | 0.97(0.75,1.26) | 0.8370 |
|  |  | MR-Egger |  |  |
|  |  | Maximum likelihood | 0.97(0.86,1.09) | 0.6187 |
|  |  | RAPS | 0.97(0.78,1.20) | 0.7985 |
| uIA-finngen | IL_6 | IVW | 1.17(0.93,1.47) | 0.1764 |
|  |  | MR-Egger |  |  |
|  |  | Maximum likelihood | 1.18(1.04,1.34) | 0.0076 |
|  |  | RAPS | 1.17(0.97,1.42) | 0.0925 |
| uIA-finngen | IL_13 | IVW | 1.13(0.88,1.46) | 0.3019 |
|  |  | MR-Egger |  |  |
|  |  | Maximum likelihood | 1.14(0.95,1.36) | 0.1342 |
|  |  | RAPS | 1.14(0.93,1.40) | 0.2002 |
| uIA-finngen | RANTES | IVW | 0.99(0.82,1.19) | 0.9392 |
|  |  | MR-Egger |  |  |
|  |  | Maximum likelihood | 0.99(0.83,1.18) | 0.9338 |
|  |  | RAPS | 0.99(0.82,1.19) | 0.9364 |
| uIA-finngen | IL_1B | IVW | 1.01(0.99,1.03) | 0.1635 |
|  |  | MR-Egger |  |  |
|  |  | Maximum likelihood | 1.01(0.85,1.21) | 0.8633 |
|  |  | RAPS | 1.01(0.84,1.21) | 0.8667 |
| uIA-finngen | SDF_1A | IVW | 1.00(0.79,1.27) | 0.9533 |
|  |  | MR-Egger |  |  |
|  |  | Maximum likelihood | 1.00(0.89,1.13) | 0.9033 |
|  |  | RAPS | 1.00(0.83,1.22) | 0.9421 |
| uIA-finngen | IL_4 | IVW | 1.10(0.94,1.30) | 0.2181 |
|  |  | MR-Egger |  |  |
|  |  | Maximum likelihood | 1.11(0.98,1.25) | 0.0778 |
|  |  | RAPS | 1.11(0.97,1.27) | 0.1250 |
| uIA-finngen | MIF | IVW | 1.02(0.77,1.35) | 0.8418 |
|  |  | MR-Egger |  |  |
|  |  | Maximum likelihood | 1.03(0.86,1.22) | 0.7410 |
|  |  | RAPS | 1.03(0.81,1.29) | 0.7973 |
| uIA-finngen | VEGF | IVW | 1.18(1.04,1.34) | 0.0080 |
|  |  | MR-Egger |  |  |
|  |  | Maximum likelihood | 1.18(1.04,1.35) | 0.0091 |
|  |  | RAPS | 1.18(1.03,1.35) | 0.0115 |
| uIA-finngen | MIP_1A | IVW | 1.24(1.23,1.25) | 1e-8 |
|  |  | MR-Egger |  |  |
|  |  | Maximum likelihood | 1.24(1.03,1.48) | 0.0178 |
|  |  | RAPS | 1.24(1.03,1.49) | 0.0209 |
| uIA-finngen | GROA | IVW | 1.00(0.95,1.06) | 0.7900 |
|  |  | MR-Egger |  |  |
|  |  | Maximum likelihood | 1.00(0.84,1.19) | 0.9288 |
|  |  | RAPS | 1.00(0.84,1.20) | 0.9307 |
| uIA-finngen | IL_8 | IVW | 1.07(0.93,1.22) | 0.3027 |
|  |  | MR-Egger |  |  |
|  |  | Maximum likelihood | 1.07(0.90,1,27) | 0.4273 |
|  |  | RAPS | 1.07(0.89,1.28) | 0.4425 |
| uIA-finngen | IFN_G | IVW | 1.11(0.94,1.31) | 0.1821 |
|  |  | MR-Egger |  |  |
|  |  | Maximum likelihood | 1.12(0.99,1.26) | 0.0667 |
|  |  | RAPS | 1.12(0.97,1.28) | 0.0968 |
| uIA-finngen | FGF_BASIC | IVW | 1.09(0.87,1.36) | 0.4434 |
|  |  | MR-Egger |  |  |
|  |  | Maximum likelihood | 1.09(0.97,1.24) | 0.1382 |
|  |  | RAPS | 1.09(0.90,1.31) | 0.3385 |
| uIA-finngen | TNF_B | IVW | 0.95(0.66,1.36) | 0.7829 |
|  |  | MR-Egger |  |  |
|  |  | Maximum likelihood | 0.94(0.73,1.23) | 0.6938 |
|  |  | RAPS | 0.94(0.70,1.27) | 0.7278 |
| uIA-finngen | MIP_1B | IVW | 1.01(0.90,1.14) | 0.7687 |
|  |  | MR-Egger |  |  |
|  |  | Maximum likelihood | 1.01(0.90,1.13) | 0.7599 |
|  |  | RAPS | 1.01(0.90,1.14) | 0.7684 |
| uIA-finngen | SCGF_B | IVW | 1.06(1.02,1.10) | 0.0019 |
|  |  | MR-Egger |  |  |
|  |  | Maximum likelihood | 1.06(0.89,1.25) | 0.5026 |
|  |  | RAPS | 1.06(0.89,1.26) | 0.4913 |
| uIA-finngen | CTACK | IVW | 0.99(0.86,1.14) | 0.9557 |
|  |  | MR-Egger |  |  |
|  |  | Maximum likelihood | 0.99(0.84,1.17) | 0.9639 |
|  |  | RAPS | 0.99(0.83,1.18) | 0.9651 |
| uIA-finngen | HGF | IVW | 0.99(0.83,1.19) | 0.9862 |
|  |  | MR-Egger |  |  |
|  |  | Maximum likelihood | 0.99(0.89,1.11) | 0.9772 |
|  |  | RAPS | 0.99(0.86,1.15) | 0.9850 |
| uIA-finngen | IL_5 | IVW | 1.08(1.01,1.16) | 0.0196 |
|  |  | MR-Egger |  |  |
|  |  | Maximum likelihood | 1.08(0.90,1.29) | 0.3648 |
|  |  | RAPS | 1.08(0.90,1.30) | 0.3777 |
| uIA-finngen | IP_10 | IVW | 0.98(0.96,1.00) | 0.0997 |
|  |  | MR-Egger |  |  |
|  |  | Maximum likelihood | 0.98(0.83,1.16) | 0.8518 |
|  |  | RAPS | 0.98(0.82(1.16) | 0.8556 |
| uIA-finngen | MIG | IVW | 1.10(0.88,1.37) | 0.3716 |
|  |  | MR-Egger |  |  |
|  |  | Maximum likelihood | 1.10(0.93,1.31) | 0.2436 |
|  |  | RAPS | 1.10(0.92,1.32) | 0.2683 |
| uIA-finngen | EOTAXIN | IVW | 0.98(0.84,1.14) | 0.8339 |
|  |  | MR-Egger |  |  |
|  |  | Maximum likelihood | 0.98(0.87,1.10) | 0.7732 |
|  |  | RAPS | 0.98(0.86,1.11) | 0.7946 |
| uIA-finngen | M_CSF | IVW | 1.09(0.85,1.40) | 0.4517 |
|  |  | MR-Egger |  |  |
|  |  | Maximum likelihood | 1.10(0.89,1.35) | 0.3661 |
|  |  | RAPS | 1.10(0.88,1.36) | 0.3857 |
| uIA-finngen | IL_2RA | IVW | 0.89(0.70,1.13) | 0.3650 |
|  |  | MR-Egger |  |  |
|  |  | Maximum likelihood | 0.89(0.75,1.06) | 0.1976 |
|  |  | RAPS | 0.89(0.73,1.08) | 0.2604 |
| uIA-finngen | SCF | IVW | 1.03(1.02,1.04) | 1.56e-29 |
|  |  | MR-Egger |  |  |
|  |  | Maximum likelihood | 1.03(0.92,1.15) | 5.82e-01 |
|  |  | RAPS | 1.03(0.91,1.15) | 5.92e-01 |
| uIA-finngen | IL_9 | IVW | 1.21(1.18,1.25) | 6.21e-48 |
|  |  | MR-Egger |  |  |
|  |  | Maximum likelihood | 1.21(1.02,1.45) | 2.76e-02 |
|  |  | RAPS | 1.21(1.01,1.45) | 3.18e-02 |
| uIA-finngen | MCP_3 | IVW | 1.34(1.13,1.58) | 0.0005 |
|  |  | MR-Egger |  |  |
|  |  | Maximum likelihood | 1.34(0.98,1.83) | 0.0648 |
|  |  | RAPS | 1.34(0.97,1.85) | 0.0727 |
| uIA-finngen | PDGF_BB | IVW | 1.03(1.01,1.05) | 0.0003 |
|  |  | MR-Egger |  |  |
|  |  | Maximum likelihood | 1.03(0.92,1.16) | 0.5272 |
|  |  | RAPS | 1.03(0.92,1.16) | 0.5378 |
| uIA-finngen | IL_1RA | IVW | 1.25(0.99,1.59) | 0.0579 |
|  |  | MR-Egger |  |  |
|  |  | Maximum likelihood | 1.26(1.05,1.52) | 0.0101 |
|  |  | RAPS | 1.26(1.04,1.54) | 0.0184 |
| uIA-finngen | IL_16 | IVW | 0.91(0.82,1.02) | 0.1231 |
|  |  | MR-Egger |  |  |
|  |  | Maximum likelihood | 0.91(0.77,1.09) | 0.3311 |
|  |  | RAPS | 0.91(0.76,1.09) | 0.3460 |
| uIA-finngen | G_CSF | IVW | 1.08(1.01,1.14) | 0.0101 |
|  |  | MR-Egger |  |  |
|  |  | Maximum likelihood | 1.08(0.96,1.21) | 0.1840 |
|  |  | RAPS | 1.08(0.96,1.21) | 0.1961 |
| uIA-finngen | IL_10 | IVW | 1.13(1.02,1.26) | 0.0188 |
|  |  | MR-Egger |  |  |
|  |  | Maximum likelihood | 1.14(1.00,1.28) | 0.0345 |
|  |  | RAPS | 1.14(1.00,1.29) | 0.0402 |
| uIA-finngen | IL_18 | IVW | 0.98(0.87,1.09) | 0.7343 |
|  |  | MR-Egger |  |  |
|  |  | Maximum likelihood | 0.98(0.82,1.16) | 0.8178 |
|  |  | RAPS | 0.98(0.82,1.16) | 0.8233 |
| uIA-finngen | TNF_A | IVW | 1.25(1.20,1.30) | 1.75e-29 |
|  |  | MR-Egger |  |  |
|  |  | Maximum likelihood | 1.25(1.04,1.50) | 1.41e-02 |
|  |  | RAPS | 1.25(1.04,1.51) | 1.68e-02 |
| uIA-finngen | IL_7 | IVW | 1.03(1.01,1.06) | 0.0016 |
|  |  | MR-Egger |  |  |
|  |  | Maximum likelihood | 1.03(0.87,1.23) | 0.6819 |
|  |  | RAPS | 1.03(0.86,1.24) | 0.6896 |
| uIA-finngen | IL_2 | IVW | 1.12(1.01,1.24) | 0.0259 |
|  |  | MR-Egger |  |  |
|  |  | Maximum likelihood | 1.12(0.94,1.34) | 0.1816 |
|  |  | RAPS | 1.12(0.94,1.34) | 0.1943 |
| uIA-finngen | IL_12_P70 | IVW | 1.13(0.96,1.32) | 0.1206 |
|  |  | MR-Egger |  |  |
|  |  | Maximum likelihood | 1.13(1.00,1.27) | 0.0363 |
|  |  | RAPS | 1.13(0.99,1.29) | 0.0533 |
| uIA-finngen | TRAIL | IVW | 1.03(1.01,1.07) | 0.0423 |
|  |  | MR-Egger |  |  |
|  |  | Maximum likelihood | 1.03(0.92,1.16) | 0.5094 |
|  |  | RAPS | 1.03(0.92,1.16) | 0.5209 |
| uIA-finngen | IL_17 | IVW | 1.11(0.97,1.27) | 0.1138 |
|  |  | MR-Egger |  |  |
|  |  | Maximum likelihood | 1.11(0.99,1.26) | 0.0679 |
|  |  | RAPS | 1.11(0.98,1.26) | 0.0780 |
| uIA-finngen | B_NGF | IVW | 0.99(0.98,1.00) | 0.3560 |
|  |  | MR-Egger |  |  |
|  |  | Maximum likelihood | 0.99(0.83,1.18) | 0.9547 |
|  |  | RAPS | 0.99(0.83,1.18) | 0.9558 |
| SAH-finngen | MCP_1_MCAF | IVW | 0.96(0.82,1.11) | 0.6268 |
|  |  | MR-Egger | 1.78(0.65,4.80) | 0.3732 |
|  |  | Weighted median | 0.99(0.87,1.13) | 0.9314 |
|  |  | Maximum likelihood | 0.96(0.86,1.06) | 0.4554 |
|  |  | RAPS | 0.96(0.83,1.12) | 0.6703 |
| SAH-finngen | IL_6 | IVW | 0.95(0.83,1.09) | 0.5223 |
|  |  | MR-Egger | 1.20(0.38,3.73) | 0.7788 |
|  |  | Weighted median | 0.91(0.80,1.04) | 0.1865 |
|  |  | Maximum likelihood | 0.95(0.86,1.05) | 0.3840 |
|  |  | RAPS | 0.95(0.82,1.09) | 0.4713 |
| SAH-finngen | IL_13 | IVW | 0.89(0.75,1.05) | 0.1781 |
|  |  | MR-Egger | 0.39(0.13,1.16) | 0.2341 |
|  |  | Weighted median | 0.86(0.71,1.04) | 0.1433 |
|  |  | Maximum likelihood | 0.88(0.76,1.03) | 0.1377 |
|  |  | RAPS | 0.88(0.74,1.05) | 0.1675 |
| SAH-finngen | RANTES | IVW | 1.07(0.97,1.19) | 0.1534 |
|  |  | MR-Egger | 0.88(0.29,2.65) | 0.8441 |
|  |  | Weighted median | 1.08(0.90,1.29) | 0.3997 |
|  |  | Maximum likelihood | 1.08(0.92,1.26) | 0.3326 |
|  |  | RAPS | 1.08(0.91,1.26) | 0.3487 |
| SAH-finngen | IL_1B | IVW | 0.92(0.75,1.12) | 0.4243 |
|  |  | MR-Egger | 0.50(0.10,2.33) | 0.4751 |
|  |  | Weighted median | 1.00(0.83,1.22) | 0.9346 |
|  |  | Maximum likelihood | 0.91(0.78,1.07) | 0.3039 |
|  |  | RAPS | 0.93(0.79,1.10) | 0.4377 |
| SAH-finngen | SDF_1A | IVW | 0.95(0.84,1.06) | 0.4036 |
|  |  | MR-Egger | 1.26(0.48,3.26) | 0.6772 |
|  |  | Weighted median | 0.93(0.81,1.06) | 0.2989 |
|  |  | Maximum likelihood | 0.94(0.85,1.05) | 0.3360 |
|  |  | RAPS | 0.94(0.83,1.07) | 0.4127 |
| SAH-finngen | IL_4 | IVW | 0.96(0.87,1.06) | 0.4526 |
|  |  | MR-Egger | 1.00(0.42,2.38) | 0.9904 |
|  |  | Weighted median | 1.00(0.89,1.13) | 0.9442 |
|  |  | Maximum likelihood | 0.96(0.86,1.06) | 0.4564 |
|  |  | RAPS | 0.96(0.86,1.06) | 0.4738 |
| SAH-finngen | MIF | IVW | 1.03(0.87,1.23) | 0.6661 |
|  |  | MR-Egger | 0.96(0.21,4.42) | 0.9683 |
|  |  | Weighted median | 0.98(0.80,1.19) | 0.8624 |
|  |  | Maximum likelihood | 1.04(0.89,1.21) | 0.6178 |
|  |  | RAPS | 1.02(0.87,1,20) | 0.7575 |
| SAH-finngen | VEGF | IVW | 1.01(0.94,1.09) | 0.6198 |
|  |  | MR-Egger | 0.89(0.41,1.92) | 0.7981 |
|  |  | Weighted median | 1.01(0.89,1.15) | 0.7925 |
|  |  | Maximum likelihood | 1.01(0.91,1.13) | 0.7393 |
|  |  | RAPS | 1.01(0.91,1.13) | 0.7473 |
| SAH-finngen | MIP_1A | IVW | 0.90(0.75,1.08) | 0.2790 |
|  |  | MR-Egger | 0.31(0.10,0.93) | 0.1725 |
|  |  | Weighted median | 0.88(0.73,1.05) | 0.2105 |
|  |  | Maximum likelihood | 0.90(0.77,1.05) | 0.1925 |
|  |  | RAPS | 0.89(0.74,1.07) | 0.2256 |
| SAH-finngen | GROA | IVW | 0.92(0.82,1.02) | 0.1424 |
|  |  | MR-Egger | 0.97(0.32,2.86) | 0.9614 |
|  |  | Weighted median | 0.88(0.74,1.06) | 0.2017 |
|  |  | Maximum likelihood | 0.92(0.78,1.07) | 0.2936 |
|  |  | RAPS | 0.92(0.78,1.07) | 0.3099 |
| SAH-finngen | IL_8 | IVW | 0.89(0.75,1.06) | 0.2105 |
|  |  | MR-Egger | 0.32(0.10,0.95) | 0.1771 |
|  |  | Weighted median | 0.93(0.77,1.13) | 0.5180 |
|  |  | Maximum likelihood | 0.89(0.76,1.04) | 0.1474 |
|  |  | RAPS | 0.90(0.77,1.06) | 0.2327 |
| SAH-finngen | IFN_G | IVW | 1.03(0.95,1.11) | 0.4021 |
|  |  | MR-Egger | 1.24(0.59,2.60) | 0.6167 |
|  |  | Weighted median | 1.05(0.93,1.19) | 0.3893 |
|  |  | Maximum likelihood | 1.03(0.93,1.14) | 0.5476 |
|  |  | RAPS | 1.03(0.92,1.14) | 0.5607 |
| SAH-finngen | FGF_BASIC | IVW | 0.95(0.90,1.00) | 0.0689 |
|  |  | MR-Egger | 1.21(0.57,2.55) | 0.6646 |
|  |  | Weighted median | 0.97(0.86,1.10) | 0.7126 |
|  |  | Maximum likelihood | 0.95(0.85,1.06) | 0.3876 |
|  |  | RAPS | 0.95(0.85,1.06) | 0.4014 |
| SAH-finngen | TNF_B | IVW | 0.88(0.85,0.91) | 1.24e-15 |
|  |  | MR-Egger | 0.95(0.18,4.93) | 9.61e-01 |
|  |  | Weighted median | 0.88(0.68,1.14) | 3.53e-01 |
|  |  | Maximum likelihood | 0.88(0.70,1.11) | 2.95e-01 |
|  |  | RAPS | 0.88(0.69,1.12) | 3.08e-01 |
| SAH-finngen | MIP_1B | IVW | 0.98(0.90,1.01) | 0.7506 |
|  |  | MR-Egger | 1.67(0.81,3.41) | 0.2942 |
|  |  | Weighted median | 0.98(0.87,1.10) | 0.7563 |
|  |  | Maximum likelihood | 0.98(0.89,1.09) | 0.7777 |
|  |  | RAPS | 0.98(0.88,1.09) | 0.7854 |
| SAH-finngen | SCGF_B | IVW | 0.89(0.70,1.15) | 0.4013 |
|  |  | MR-Egger | 0.39(0.06,2.51) | 0.4309 |
|  |  | Weighted median | 0.87(0.71,1.06) | 0.1843 |
|  |  | Maximum likelihood | 0.89(0.76,1.04) | 0.1538 |
|  |  | RAPS | 0.86(0.67,1.09) | 0.2291 |
| SAH-finngen | CTACK | IVW | 1.14(1.00,1.30) | 0.0401 |
|  |  | MR-Egger | 2.00(0.69,5.84) | 0.3292 |
|  |  | Weighted median | 1.13(0.94,1.35) | 0.1701 |
|  |  | Maximum likelihood | 1.14(0.98,1.33) | 0.0777 |
|  |  | RAPS | 1.14(0.97,1.34) | 0.0877 |
| SAH-finngen | HGF | IVW | 0.91(0.84,0.98) | 0.0126 |
|  |  | MR-Egger | 1.30(0.64,2.67) | 0.5353 |
|  |  | Weighted median | 0.94(0.83,1.05) | 0.3153 |
|  |  | Maximum likelihood | 0.91(0.82,1.00) | 0.0749 |
|  |  | RAPS | 0.91(0.82,1.01) | 0.0853 |
| SAH-finngen | IL_5 | IVW | 0.87(0.75,1.02) | 0.1085 |
|  |  | MR-Egger | 0.50(0.16,1.60) | 0.3685 |
|  |  | Weighted median | 0.94(0.78,1.14) | 0.5545 |
|  |  | Maximum likelihood | 0.87(0.74,1.02) | 0.1033 |
|  |  | RAPS | 0.87(0.74,1.03) | 0.1174 |
| SAH-finngen | IP_10 | IVW | 0.95(0.87,1.05) | 0.3701 |
|  |  | MR-Egger | 0.76(0.26,2.20) | 0.6674 |
|  |  | Weighted median | 0.95(0.79,1.13) | 0.5852 |
|  |  | Maximum likelihood | 0.95(0.82,1.11) | 0.5862 |
|  |  | RAPS | 0.95(0.82,1.11) | 0.5972 |
| SAH-finngen | MIG | IVW | 0.99(0.84,1.16) | 0.9180 |
|  |  | MR-Egger | 0.43(0.14,1.24) | 0.2612 |
|  |  | Weighted median | 0.94(0.78,1.13) | 0.5188 |
|  |  | Maximum likelihood | 0.99(0.85,1.15) | 0.9125 |
|  |  | RAPS | 0.98(0.83,1.15) | 0.8448 |
| SAH-finngen | EOTAXIN | IVW | 0.92(0.85,0.99) | 0.0428 |
|  |  | MR-Egger | 0.97(0.47,2.00) | 0.9564 |
|  |  | Weighted median | 0.89(0.79,1.01) | 0.0869 |
|  |  | Maximum likelihood | 0.92(0.83,1.01) | 0.1111 |
|  |  | RAPS | 0.92(0.82,1.02) | 0.1236 |
| SAH-finngen | M_CSF | IVW | 0.98(0.81,1.19) | 0.8940 |
|  |  | MR-Egger | 0.97(0.19,4.94) | 0.9814 |
|  |  | Weighted median | 0.93(0.74,1.16) | 0.5373 |
|  |  | Maximum likelihood | 0.98(0.82,1.18) | 0.8897 |
|  |  | RAPS | 0.98(0.81,1.19) | 0.8962 |
| SAH-finngen | IL_2RA | IVW | 0.85(0.72,1.02) | 0.0861 |
|  |  | MR-Egger | 1.74(0.55,5.47) | 0.4384 |
|  |  | Weighted median | 0.89(0.73,1.08) | 0.2479 |
|  |  | Maximum likelihood | 0.85(0.73,0.99) | 0.0453 |
|  |  | RAPS | 0.85(0.71,1.03) | 0.1076 |
| SAH-finngen | SCF | IVW | 0.89(0.81,0.98) | 0.0244 |
|  |  | MR-Egger | 0.93(0.39,2.19) | 0.8892 |
|  |  | Weighted median | 0.88(0.78,0.99) | 0.0489 |
|  |  | Maximum likelihood | 0.89(0.80,0.98) | 0.0283 |
|  |  | RAPS | 0.89(0.80,0.99) | 0.0353 |
| SAH-finngen | IL_9 | IVW | 1.02(0.87,1.20) | 0.7867 |
|  |  | MR-Egger | 0.37(0.13,1.10) | 0.2171 |
|  |  | Weighted median | 1.04(0.87,1.25) | 0.6452 |
|  |  | Maximum likelihood | 1.02(0.87,1.19) | 0.7696 |
|  |  | RAPS | 1.02(0.87,1.20) | 0.7169 |
| SAH-finngen | MCP_3 | IVW | 0.81(0.54,1.20) | 0.3068 |
|  |  | MR-Egger | 0.43(0.01,2.80) | 0.6771 |
|  |  | Weighted median | 0.76(0.50,1.14) | 0.1903 |
|  |  | Maximum likelihood | 0.80(0.60,1.07) | 0.1460 |
|  |  | RAPS | 0.76(0.51,1.13) | 0.1885 |
| SAH-finngen | PDGF_BB | IVW | 0.99(0.94,1.04) | 0.7438 |
|  |  | MR-Egger | 1.02(0.50,2.08) | 0.9568 |
|  |  | Weighted median | 0.99(0.88,1.10) | 0.8676 |
|  |  | Maximum likelihood | 0.99(0.89,1.09) | 0.8683 |
|  |  | RAPS | 0.99(0.89,1.09) | 0.8721 |
| SAH-finngen | IL_1RA | IVW | 1.00(0.79,1.25) | 0.9919 |
|  |  | MR-Egger | 0.59(0.09,3.74) | 0.6327 |
|  |  | Weighted median | 0.97(0.79,1.19) | 0.7910 |
|  |  | Maximum likelihood | 1.00(0.85,1,16) | 0.9875 |
|  |  | RAPS | 0.96(0.76,1,21) | 0.7601 |
| SAH-finngen | IL_16 | IVW | 1.02(0.92,1.13) | 0.6781 |
|  |  | MR-Egger | 1.32(0.44,3.90) | 0.6654 |
|  |  | Weighted median | 1.03(0.87,1.23) | 0.6878 |
|  |  | Maximum likelihood | 1.02(0.87,1.19) | 0.7811 |
|  |  | RAPS | 1.02(0.87,1.19) | 0.7878 |
| SAH-finngen | G_CSF | IVW | 0.96(0.88,1.04) | 0.3399 |
|  |  | MR-Egger | 0.80(0.38,1.66) | 0.6214 |
|  |  | Weighted median | 0.98(0.87,1.10) | 0.8134 |
|  |  | Maximum likelihood | 0.95(0.86,1.06) | 0.4306 |
|  |  | RAPS | 0.95(0.86,1.06) | 0.4459 |
| SAH-finngen | IL_10 | IVW | 0.96(0.88,1.05) | 0.4594 |
|  |  | MR-Egger | 0.63(0.30,1.33) | 0.3577 |
|  |  | Weighted median | 0.98(0.87,1.10) | 0.7793 |
|  |  | Maximum likelihood | 0.96(0.87,1.07) | 0.5135 |
|  |  | RAPS | 0.96(0.86,1.07) | 0.5284 |
| SAH-finngen | IL_18 | IVW | 0.98(0.83,1.15) | 0.8229 |
|  |  | MR-Egger | 2.03(0.69,5.90) | 0.3233 |
|  |  | Weighted median | 1.03(0.85,1.23) | 0.7491 |
|  |  | Maximum likelihood | 0.98(0.84,1.14) | 0.8064 |
|  |  | RAPS | 0.98(0.82,1.17) | 0.8543 |
| SAH-finngen | TNF_A | IVW | 0.91(0.86,0.96) | 0.0025 |
|  |  | MR-Egger | 0.64(0.21,1.91) | 0.5086 |
|  |  | Weighted median | 0.92(0.77,1.10) | 0.3789 |
|  |  | Maximum likelihood | 0.91(0.78,1.06) | 0.2519 |
|  |  | RAPS | 0.91(0.77,1.07) | 0.2646 |
| SAH-finngen | IL_7 | IVW | 0.90(0.85,0.95) | 0.0001 |
|  |  | MR-Egger | 0.81(0.27,2.46) | 0.7556 |
|  |  | Weighted median | 0.89(0.75,1.06) | 0.2214 |
|  |  | Maximum likelihood | 0.90(0.77,1.05) | 0.2023 |
|  |  | RAPS | 0.90(0.76,1.06) | 0.2148 |
| SAH-finngen | IL_2 | IVW | 0.98(0.77,1.24) | 0.8900 |
|  |  | MR-Egger | 0.42(0.07,2.23) | 0.4162 |
|  |  | Weighted median | 1.00(0.81,1.24) | 0.9497 |
|  |  | Maximum likelihood | 0.98(0.84,1.14) | 0.8289 |
|  |  | RAPS | 1.01(0.86,1.19) | 0.8236 |
| SAH-finngen | IL_12_P70 | IVW | 0.96(0.88,1.05) | 0.4522 |
|  |  | MR-Egger | 0.89(0.41,1.91) | 0.7953 |
|  |  | Weighted median | 0.96(0.85,1.09) | 0.6075 |
|  |  | Maximum likelihood | 0.96(0.87,1.06) | 0.5020 |
|  |  | RAPS | 0.96(0.86,1.07) | 0.5179 |
| SAH-finngen | TRAIL | IVW | 0.94(0.89,0.99) | 0.0355 |
|  |  | MR-Egger | 0.95(0.46,1.94) | 0.9101 |
|  |  | Weighted median | 0.96(0.85,1.09) | 0.5904 |
|  |  | Maximum likelihood | 0.94(0.85,1.04) | 0.2851 |
|  |  | RAPS | 0.94(0.85,1.05) | 0.2997 |
| SAH-finngen | IL_17 | IVW | 0.93(0.87,0.99) | 0.0399 |
|  |  | MR-Egger | 1.07(0.51,2.24) | 0.8715 |
|  |  | Weighted median | 0.91(0.81,1.03) | 0.1630 |
|  |  | Maximum likelihood | 0.93(0.84,1.03) | 0.1888 |
|  |  | RAPS | 0.93(0.83,1.03) | 0.2035 |
| SAH-finngen | B_NGF | IVW | 0.88(0.81,0.95) | 0.0011 |
|  |  | MR-Egger | 0.55(0.18,1,64) | 0.4023 |
|  |  | Weighted median | 0.89(0.75,1.06) | 0.2129 |
|  |  | Maximum likelihood | 0.88(0.75,1.02) | 0,.1062 |
|  |  | RAPS | 0.88(0.75,1.03) | 0.1159 |

IA, intracranial aneurysm; uIA, unruptured aneurysm; SAH, subarachnoid hemorrhage; MR, mendelian randomization; SNP, single-nucleotide polymorphisms; IVW, inverse variance weighting; RAPS, robust adjusted profile score

**Table S15** The MR-Egger and Cochran’s test results of IA, uIA, and SAH on forty-one inflammation cytokines in FinnGen datasets in replicate reverse MR analysis

| Exposure | Outcome | Methods | Egger_intercept | P-Egger_intercept | Cochran’s | Cochran’s *P* |
| --- | --- | --- | --- | --- | --- | --- |
| IA-finngen | IL_1B | IVW |  |  | 0.04 | 0.8238 |
|  |  | MR-Egger | NA | NA | NA | NA |
| uIA-finngen | VEGF | IVW |  |  | 1.07 | 0.2996 |
|  |  | MR-Egger | NA | NA | NA | NA |
| uIA-finngen | MIP_1A | IVW |  |  | 1.5e-4 | 0.9686 |
|  |  | MR-Egger | NA | NA | NA | NA |
| uIA-finngen | SCGF_B | IVW |  |  | 0.04 | 0.8232 |
|  |  | MR-Egger | NA | NA | NA | NA |
| uIA-finngen | IL_5 | IVW |  |  | 0.15 | 0.6960 |
|  |  | MR-Egger | NA | NA | NA | NA |
| uIA-finngen | SCF | IVW |  |  | 2.3e-3 | 0.9610 |
|  |  | MR-Egger | NA | NA | NA | NA |
| uIA-finngen | IL_9 | IVW |  |  | 0.02 | 0.8746 |
|  |  | MR-Egger | NA | NA | NA | NA |
| uIA-finngen | MCP_3 | IVW |  |  | 0.30 | 0.5813 |
|  |  | MR-Egger | NA | NA | NA | NA |
| uIA-finngen | PDGF_BB | IVW |  |  | 0.03 | 0.8597 |
|  |  | MR-Egger | NA | NA | NA | NA |
| uIA-finngen | G_CSF | IVW |  |  | 0.27 | 0.6008 |
|  |  | MR-Egger | NA | NA | NA | NA |
| uIA-finngen | IL_10 | IVW |  |  | 0.86 | 0.3519 |
|  |  | MR-Egger | NA | NA | NA | NA |
| uIA-finngen | TNF_A | IVW |  |  | 0.05 | 0.8188 |
|  |  | MR-Egger | NA | NA | NA | NA |
| uIA-finngen | IL_7 | IVW |  |  | 0.01 | 0.8962 |
|  |  | MR-Egger | NA | NA | NA | NA |
| uIA-finngen | IL_2 | IVW |  |  | 0.36 | 0.5434 |
|  |  | MR-Egger | NA | NA | NA | NA |
| uIA-finngen | TRAIL | IVW |  |  | 0.106 | 0.7445 |
|  |  | MR-Egger | NA | NA | NA | NA |
| SAH-finngen | TNF_B | IVW |  |  | 0.05 | 0.9969 |
|  |  | MR-Egger | -0.0141 | 0.9339 | 0.04 | 0.9787 |
| SAH-finngen | CTACK | IVW |  |  | 2.23 | 0.5251 |
|  |  | MR-Egger | -0.1022 | 0.4074 | 1.15 | 0.5620 |
| SAH-finngen | HGF | IVW |  |  | 1.54 | 0.6718 |
|  |  | MR-Egger | -0.0657 | 0.4215 | 0.53 | 0.7635 |
| SAH-finngen | EOTAXIN | IVW |  |  | 1.86 | 0.6006 |
|  |  | MR-Egger | -0.0108 | 0.8850 | 1.83 | 0.3986 |
| SAH-finngen | SCF | IVW |  |  | 2.88 | 0.4096 |
|  |  | MR-Egger | -0.0079 | 0.9283 | 2.87 | 0.2380 |
| SAH-finngen | TNF_A | IVW |  |  | 0.43 | 0.9332 |
|  |  | MR-Egger | 0.0647 | 0.5863 | 0.02 | 0.9896 |
| SAH-finngen | IL_7 | IVW |  |  | 0.33 | 0.9528 |
|  |  | MR-Egger | 0.0181 | 0.8749 | 0.30 | 0.8583 |
| SAH-finngen | TRAIL | IVW |  |  | 0.77 | 0.8551 |
|  |  | MR-Egger | -0.0014 | 0.9839 | 0.77 | 0.6783 |
| SAH-finngen | IL_17 | IVW |  |  | 1.23 | 0.7456 |
|  |  | MR-Egger | -0.0251 | 0.7464 | 1.09 | 0.5788 |
| SAH-finngen | B_NGF | IVW |  |  | 0.74 | 0.8624 |
|  |  | MR-Egger | 0.0829 | 0.4925 | 0.05 | 0.9744 |

IA, intracranial aneurysm; uIA, unruptured aneurysm; SAH, subarachnoid hemorrhage; MR, mendelian randomization; IVW, inverse variance weighting


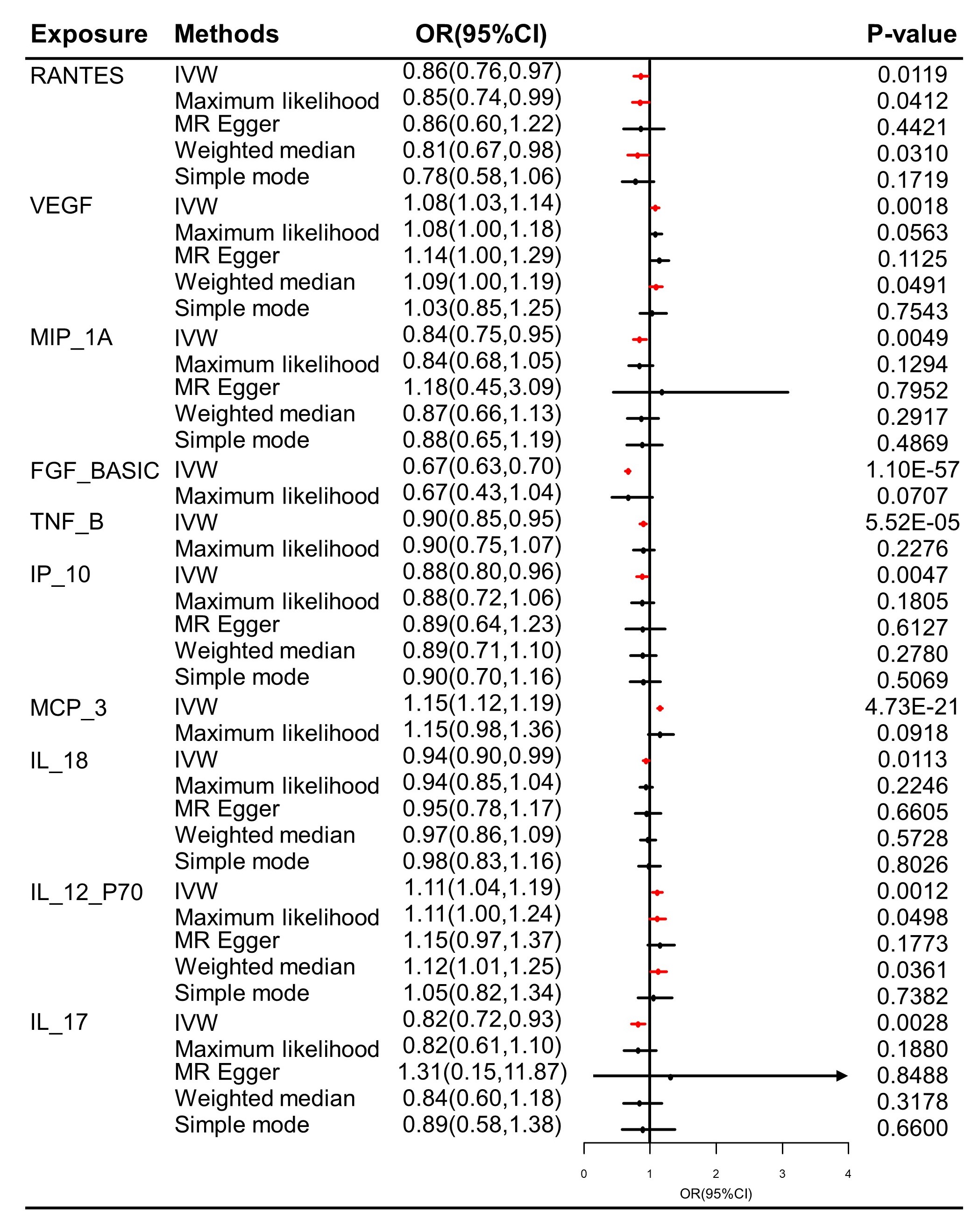


**Figure S1** Causal association between inflammatory cytokines and IA in MR analysis. MR, mendelian randomization; IA, intracranial aneurysm


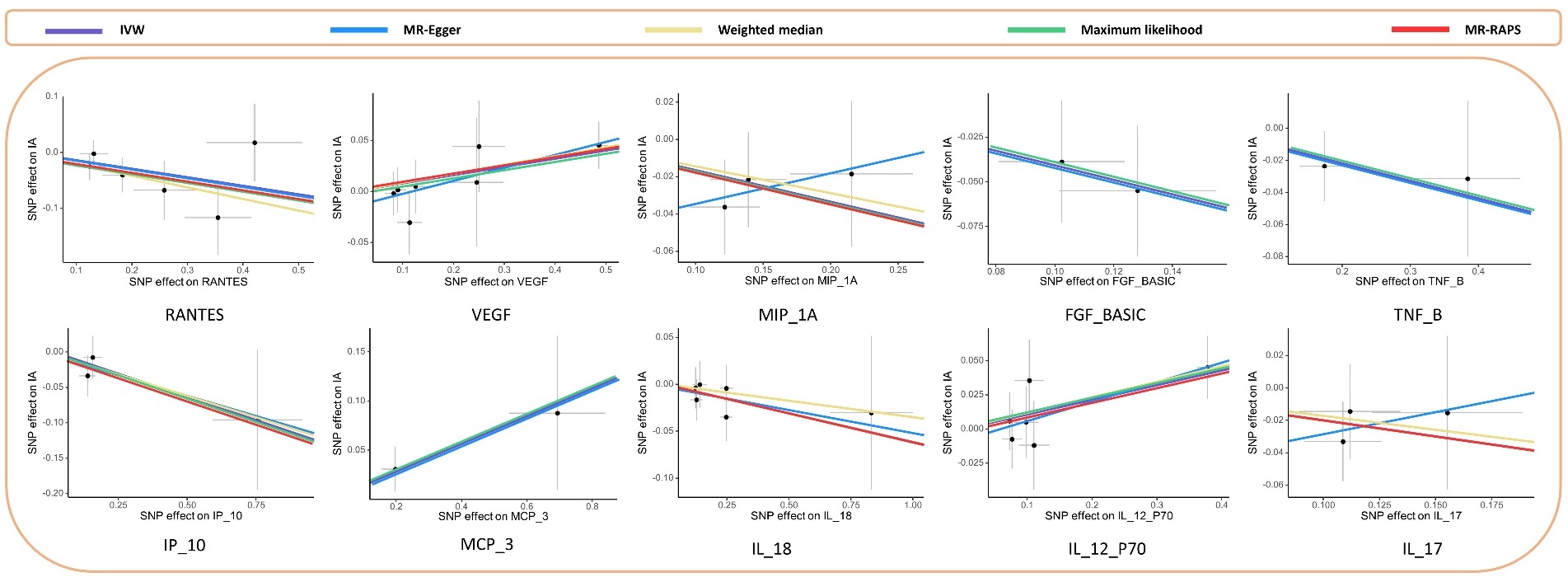


**Figure S2** The scatter plots of the association between genetically predicted inflammation cytokines on IA in MR analysis. MR, mendelian randomization; IA, intracranial aneurysm


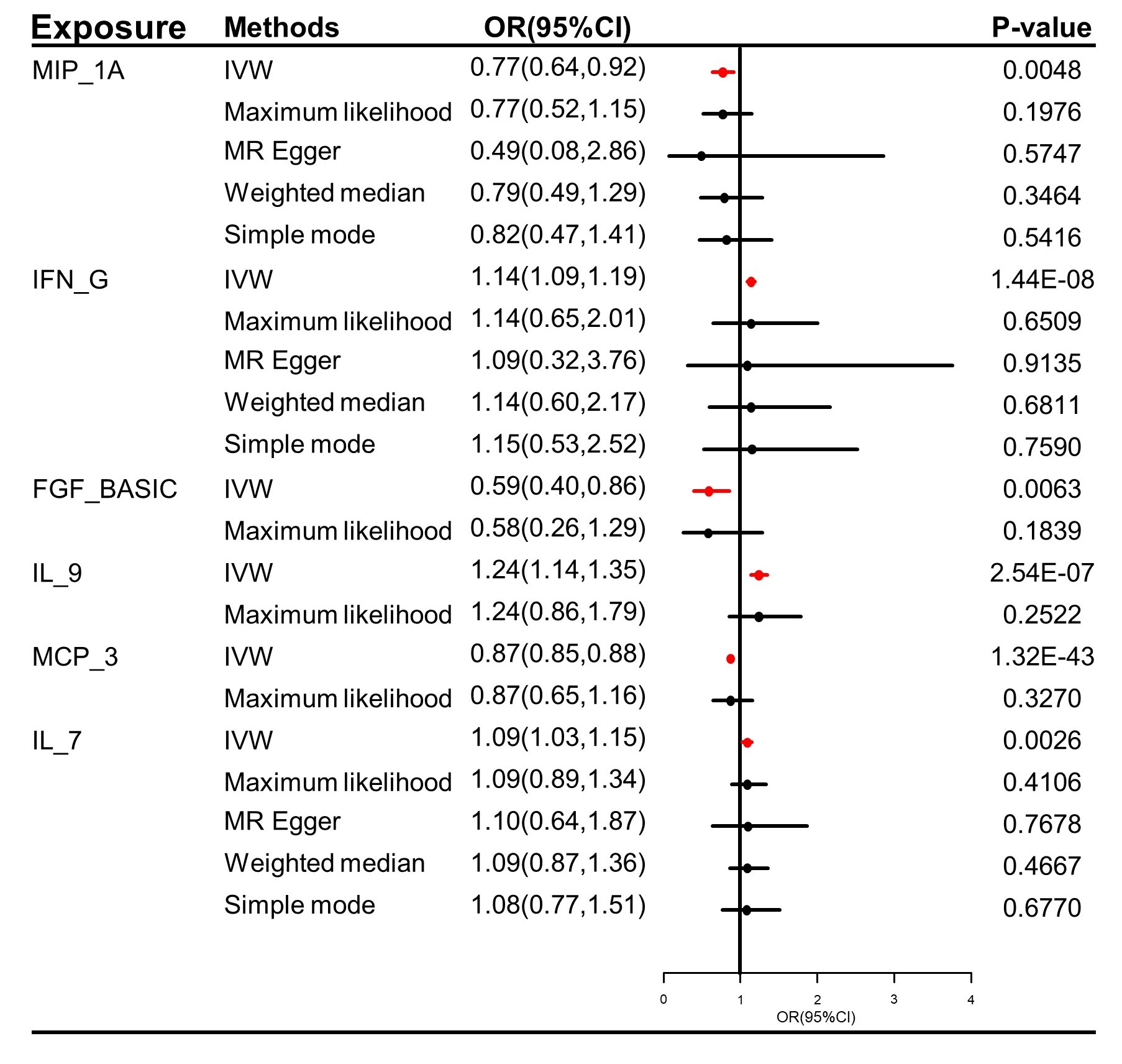


**Figure S3** Causal association between inflammatory cytokines and uIA in MR analysis. MR, mendelian randomization; uIA, unruptured intracranial aneurysm


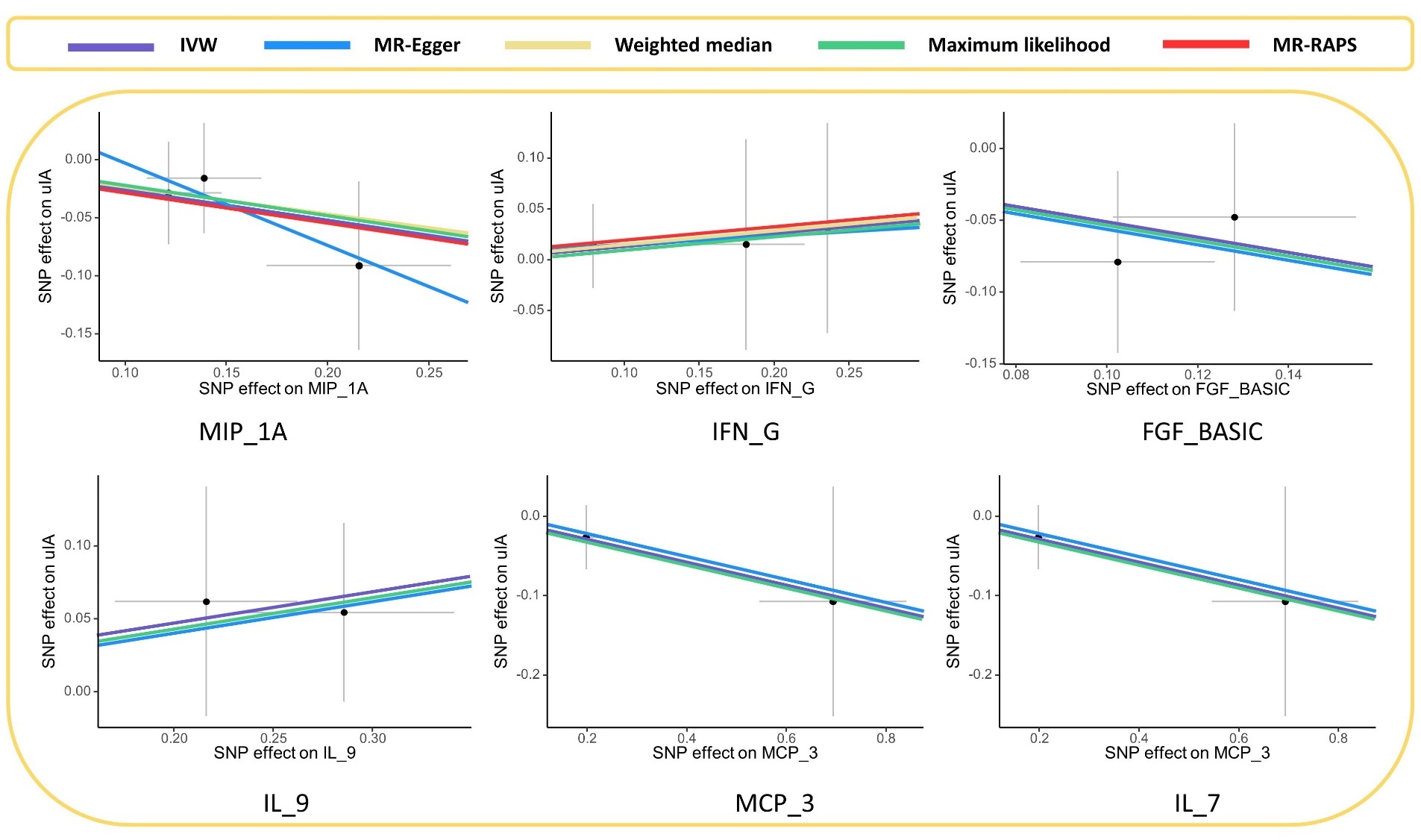


**Figure S4** The scatter plots of the association between genetically predicted inflammation cytokines on uIA in MR analysis. MR, mendelian randomization; uIA, unruptured intracranial aneurysm


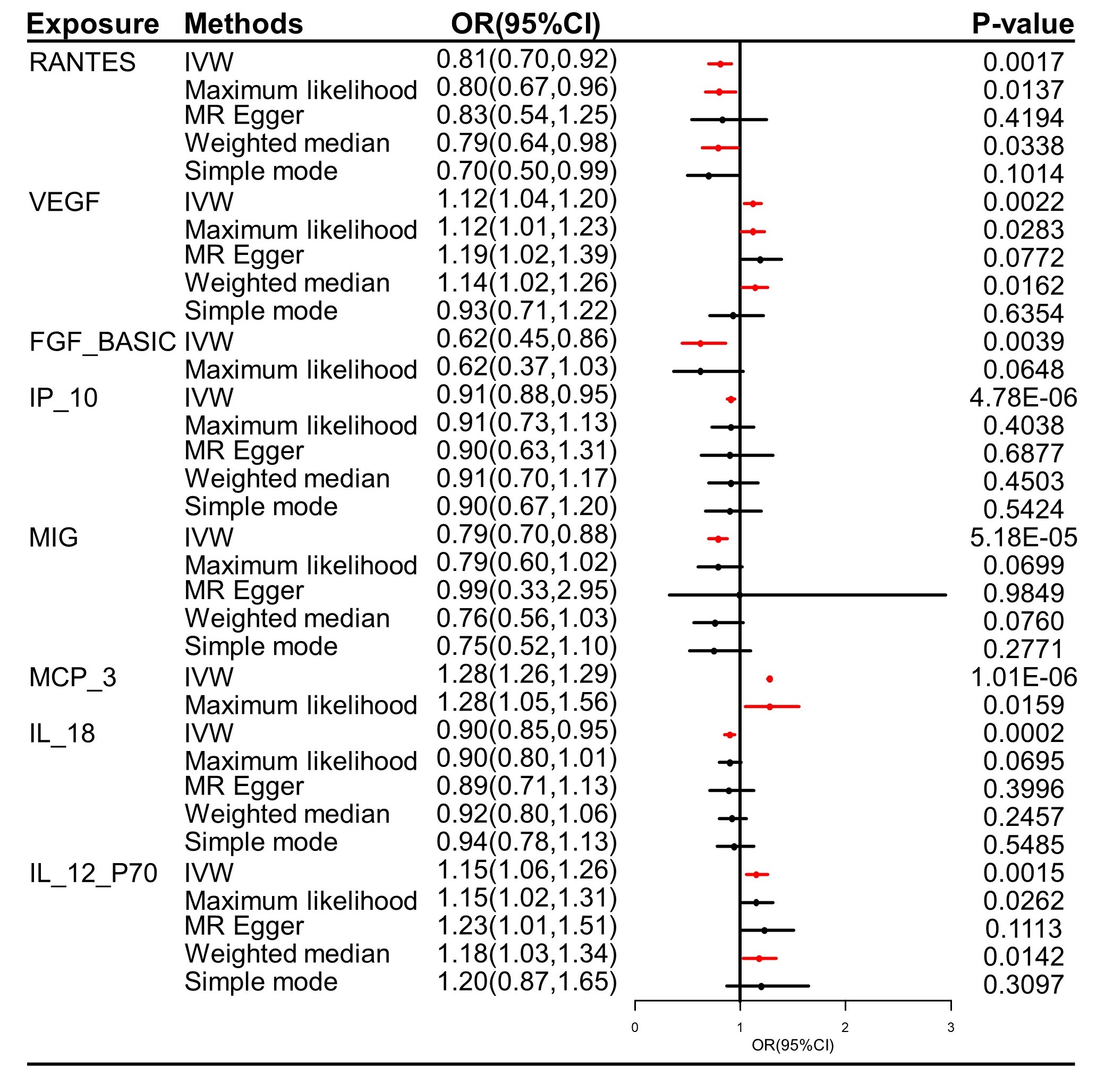


**Figure S5** Causal association between inflammatory cytokines and SAH in MR analysis. MR, mendelian randomization; SAH, subarachnoid hemorrhage


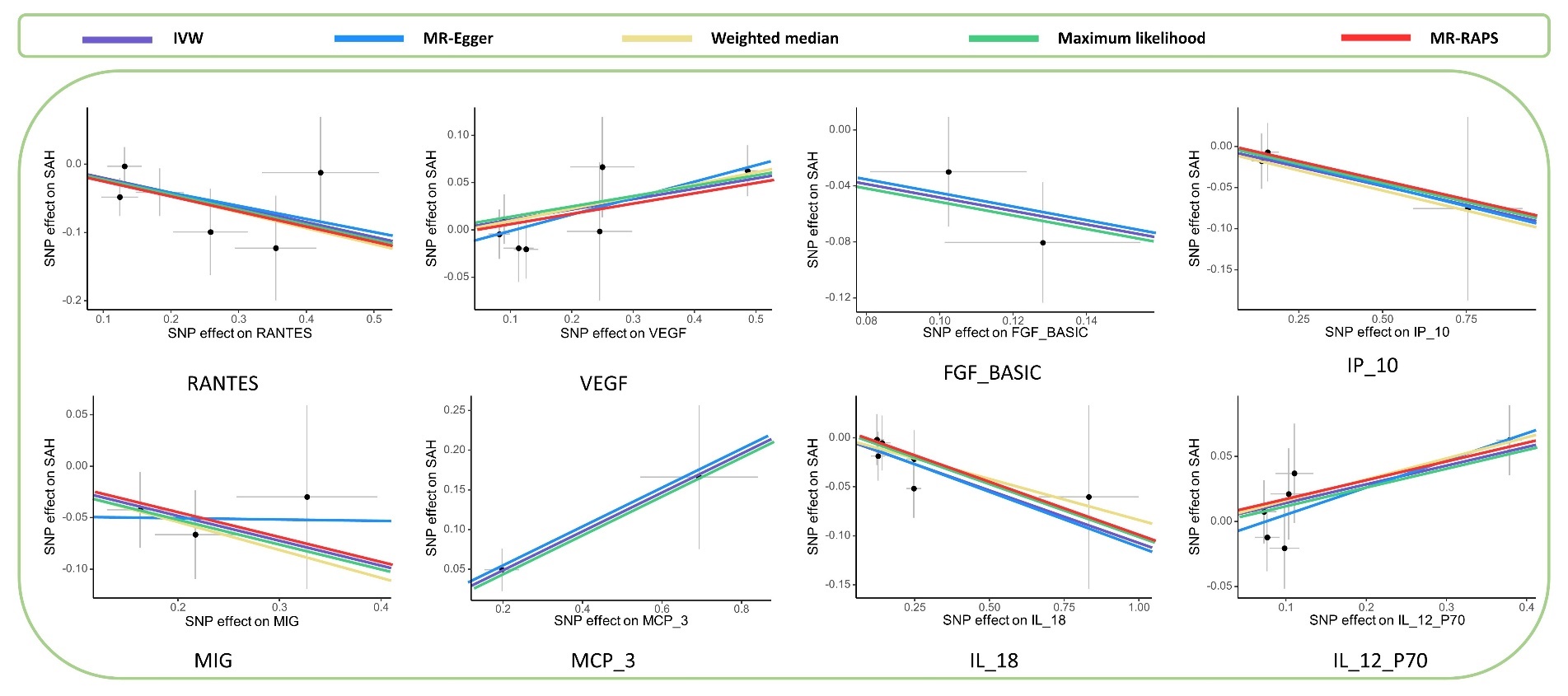


**Figure S6** The scatter plots of the association between genetically predicted inflammation cytokines on SAH in MR analysis. MR, mendelian randomization; SAH, subarachnoid hemorrhage


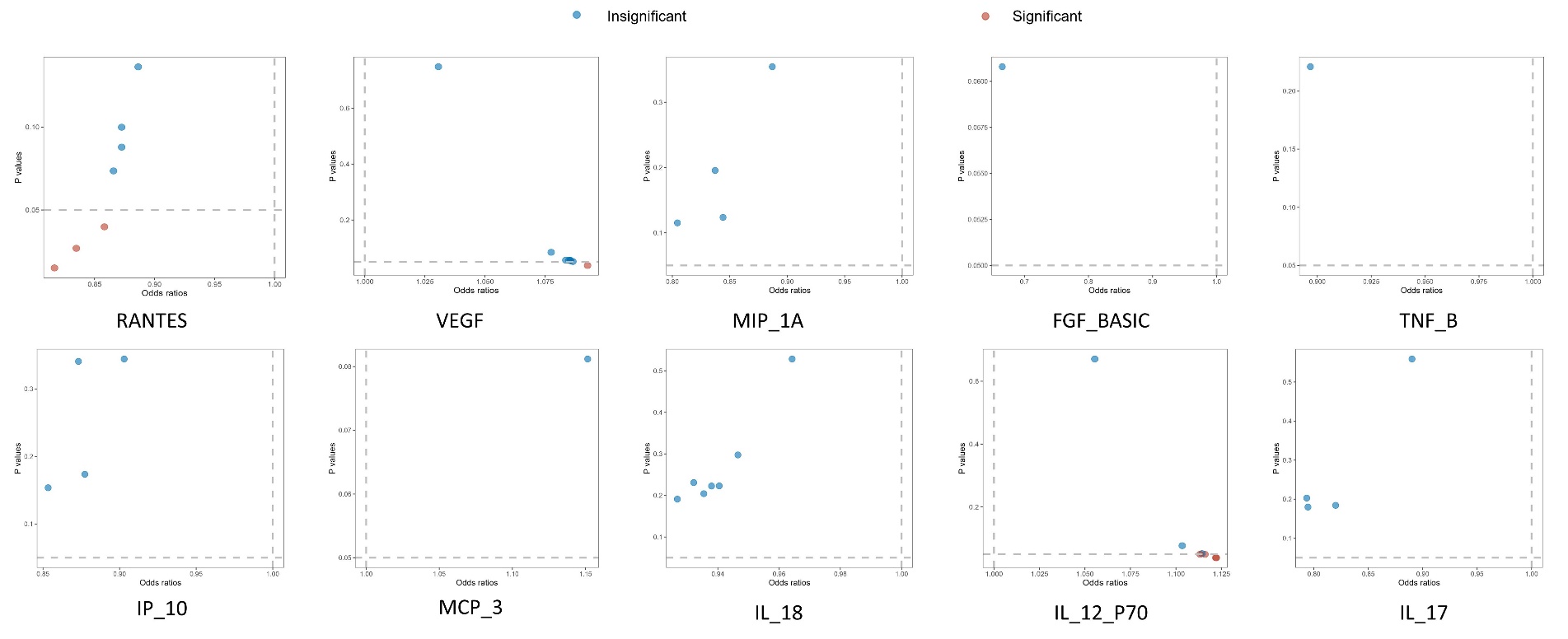


**Figure S7** The leave-one-out analysis for causal association between inflammation cytokines and IA in MR analysis. MR, mendelian randomization; IA, intracranial aneurysm


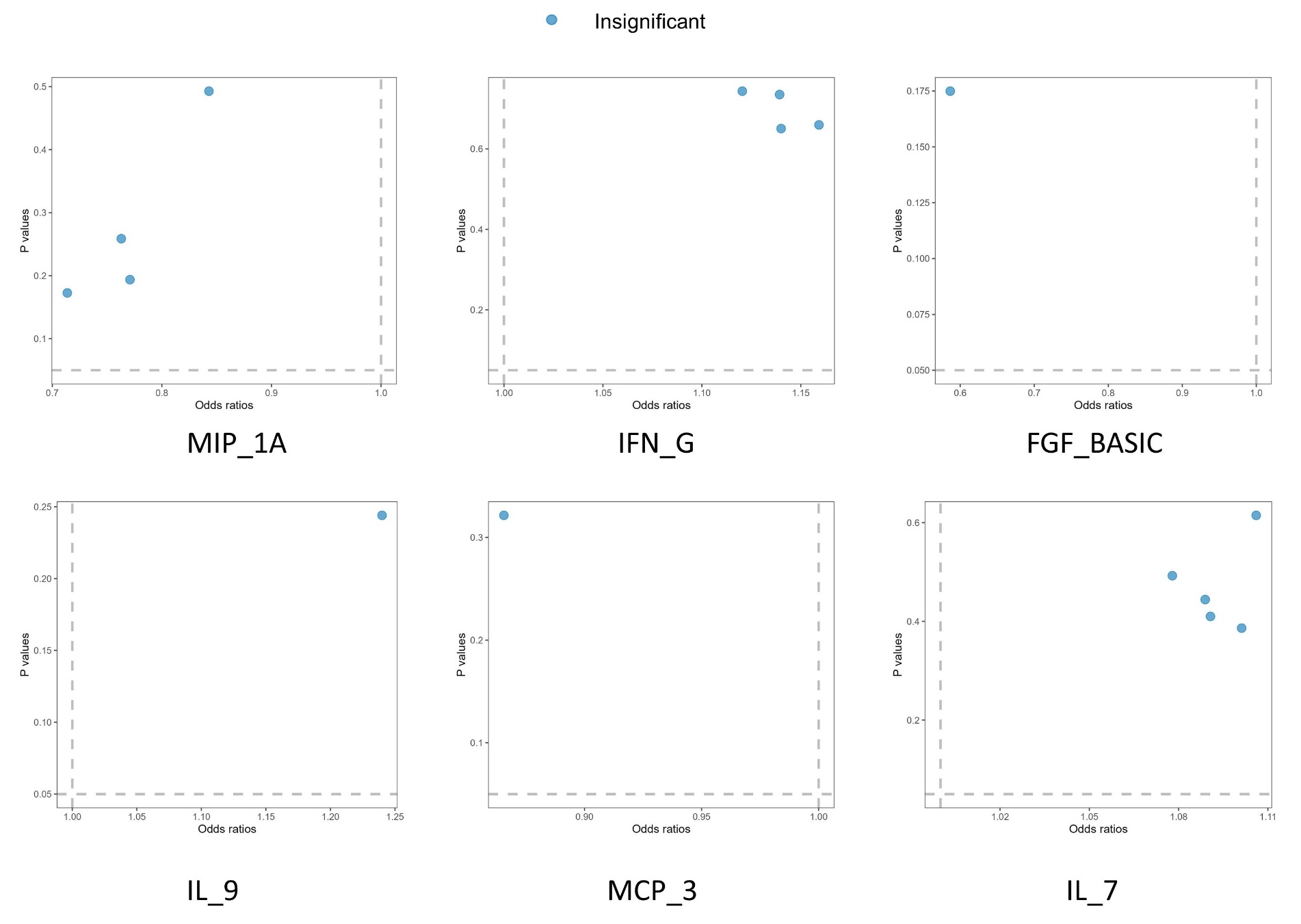


**Figure S8** The leave-one-out analysis for causal association between inflammation cytokines and uIA in MR analysis. MR, mendelian randomization; uIA, unruptured intracranial aneurysm


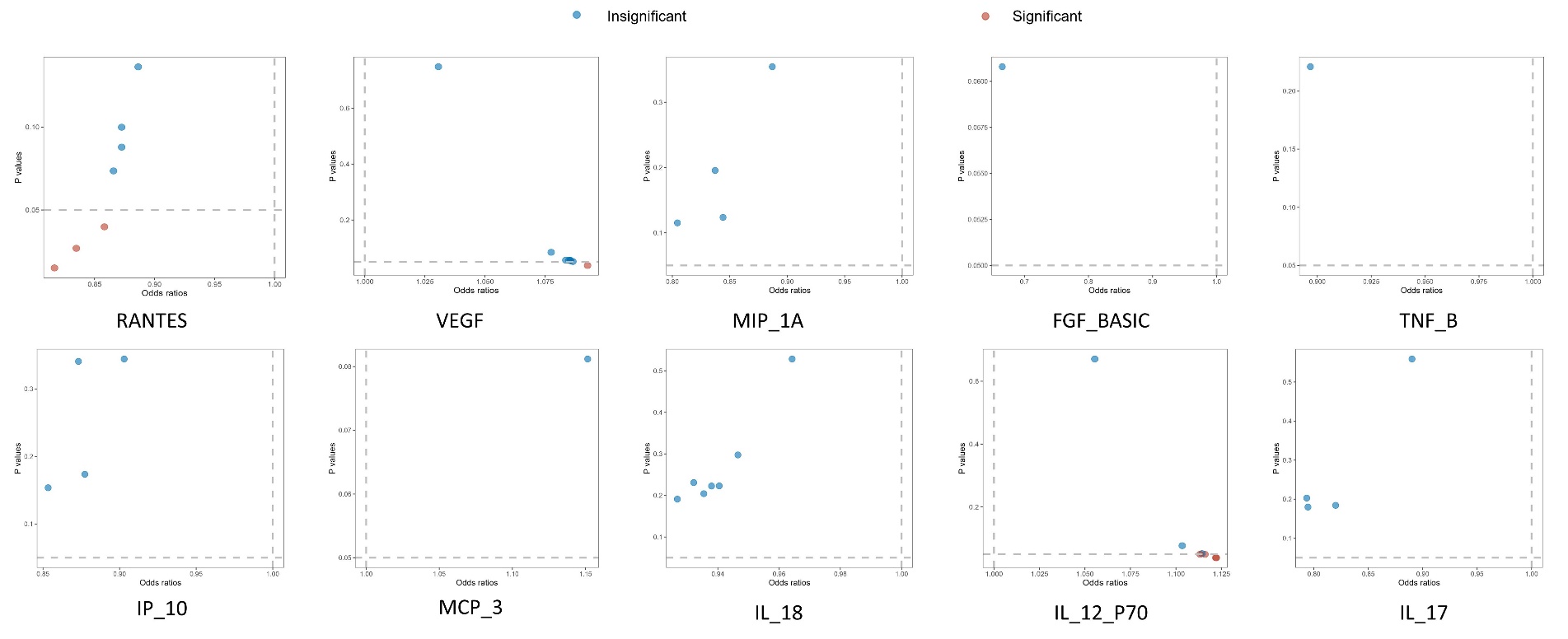


**Figure S9** The leave-one-out analysis for causal association between inflammation cytokines and SAH in MR analysis. MR, mendelian randomization; SAH, subarachnoid hemorrhage


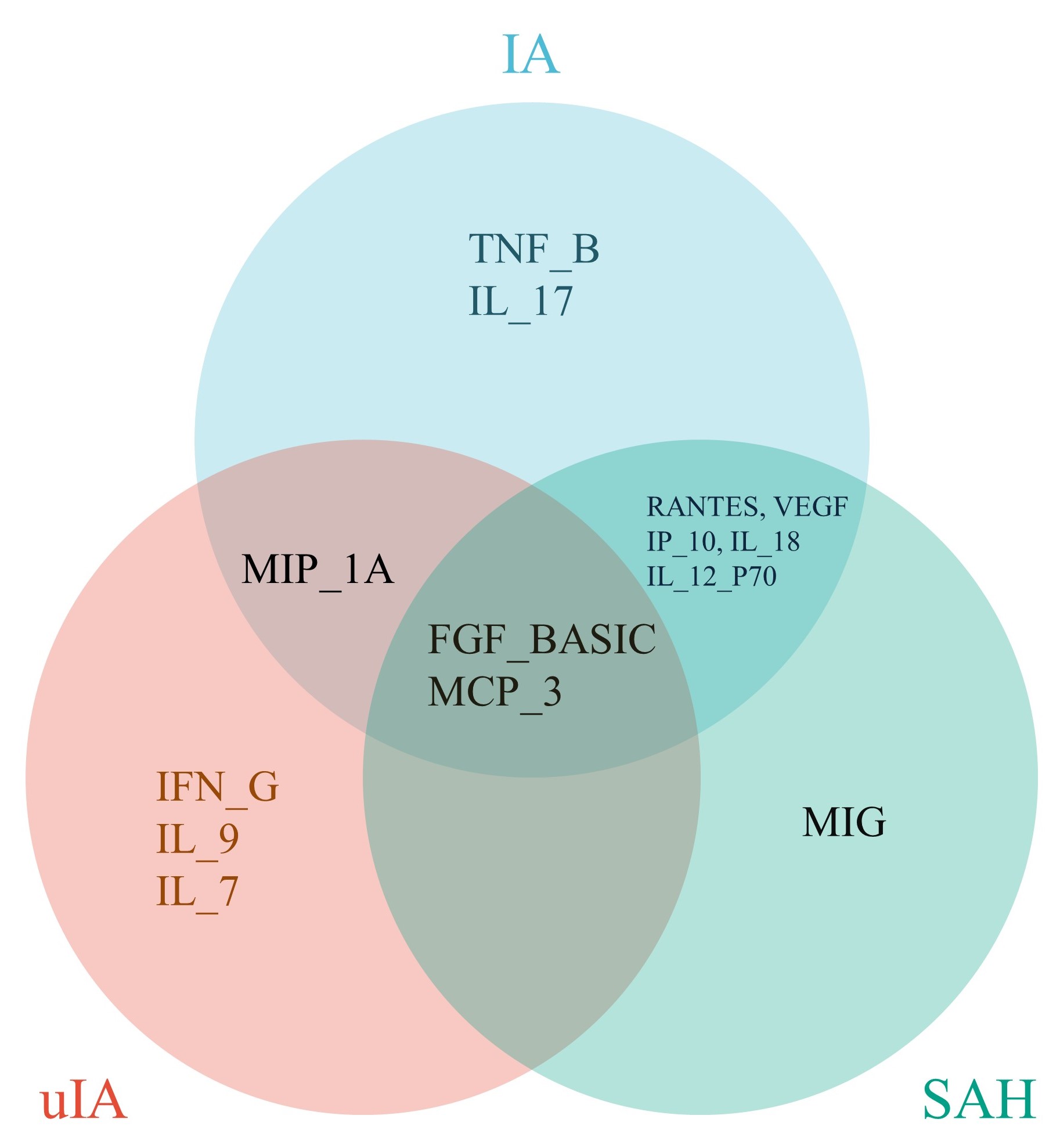


**Figure S10** The common inflammation cytokines among IA, uIA, and SAH in MR analysis. IA, intracranial aneurysm; uIA, unruptured intracranial aneurysm, SAH, subarachnoid hemorrhage; MR, mendelian randomization.


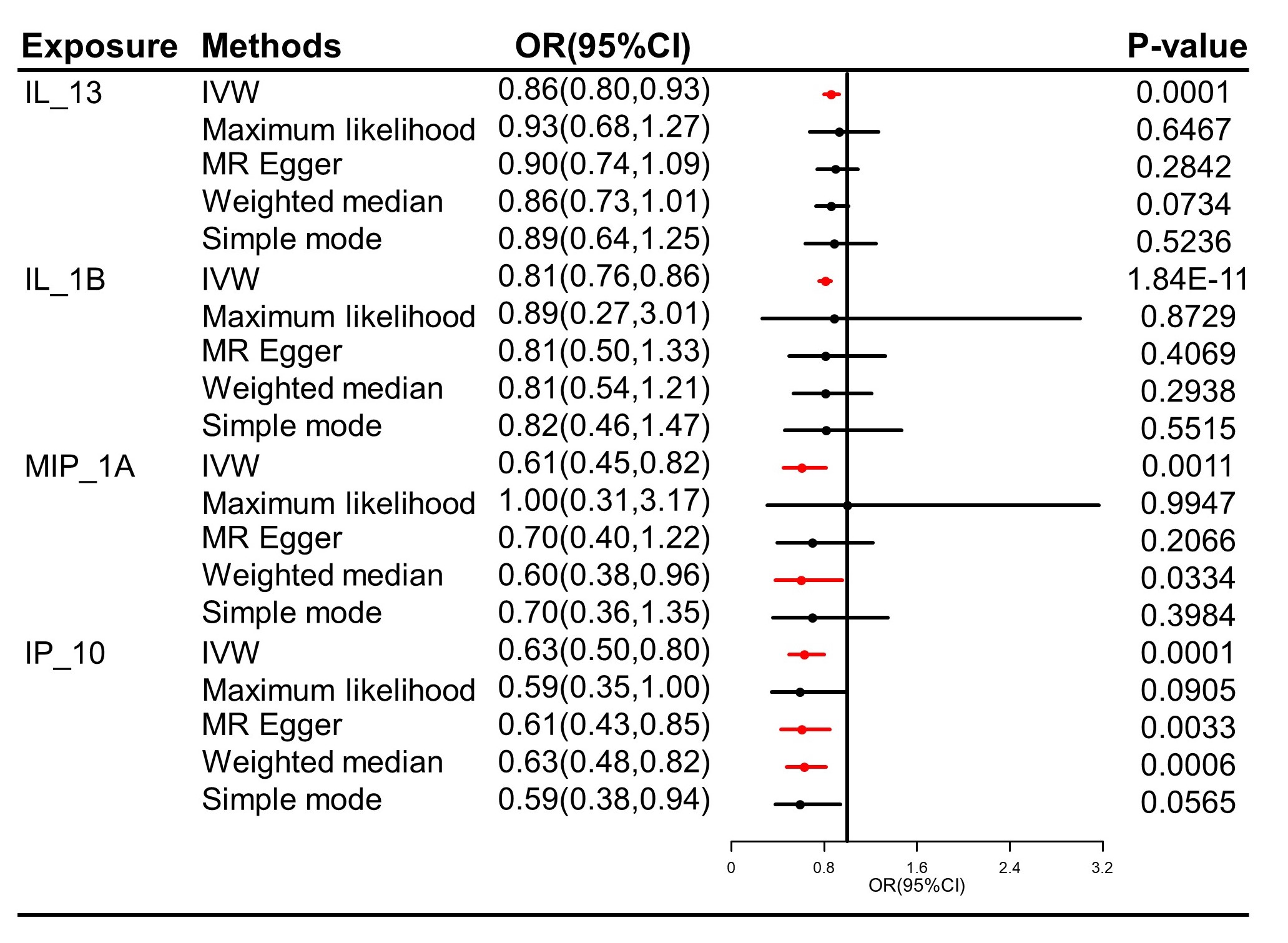


**Figure S11** Causal association between inflammatory cytokines and IA in replicate MR analysis. IA, intracranial aneurysm; MR, mendelian randomization.


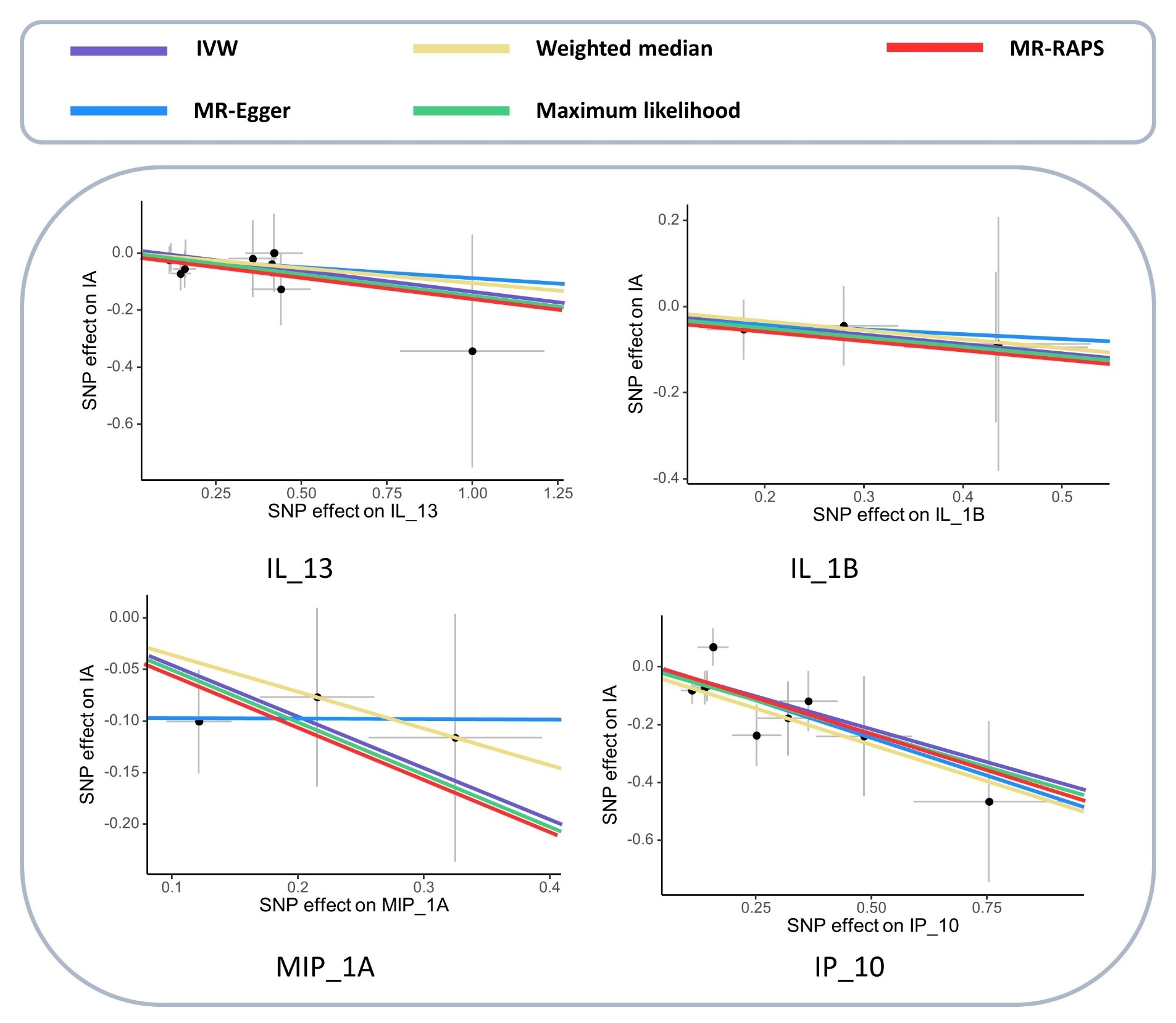


**Figure S12** The scatter plots of the association between genetically predicted inflammation cytokines on IA in FinnGen datasets in replicate MR analysis. IA, intracranial aneurysm; MR, mendelian randomization.


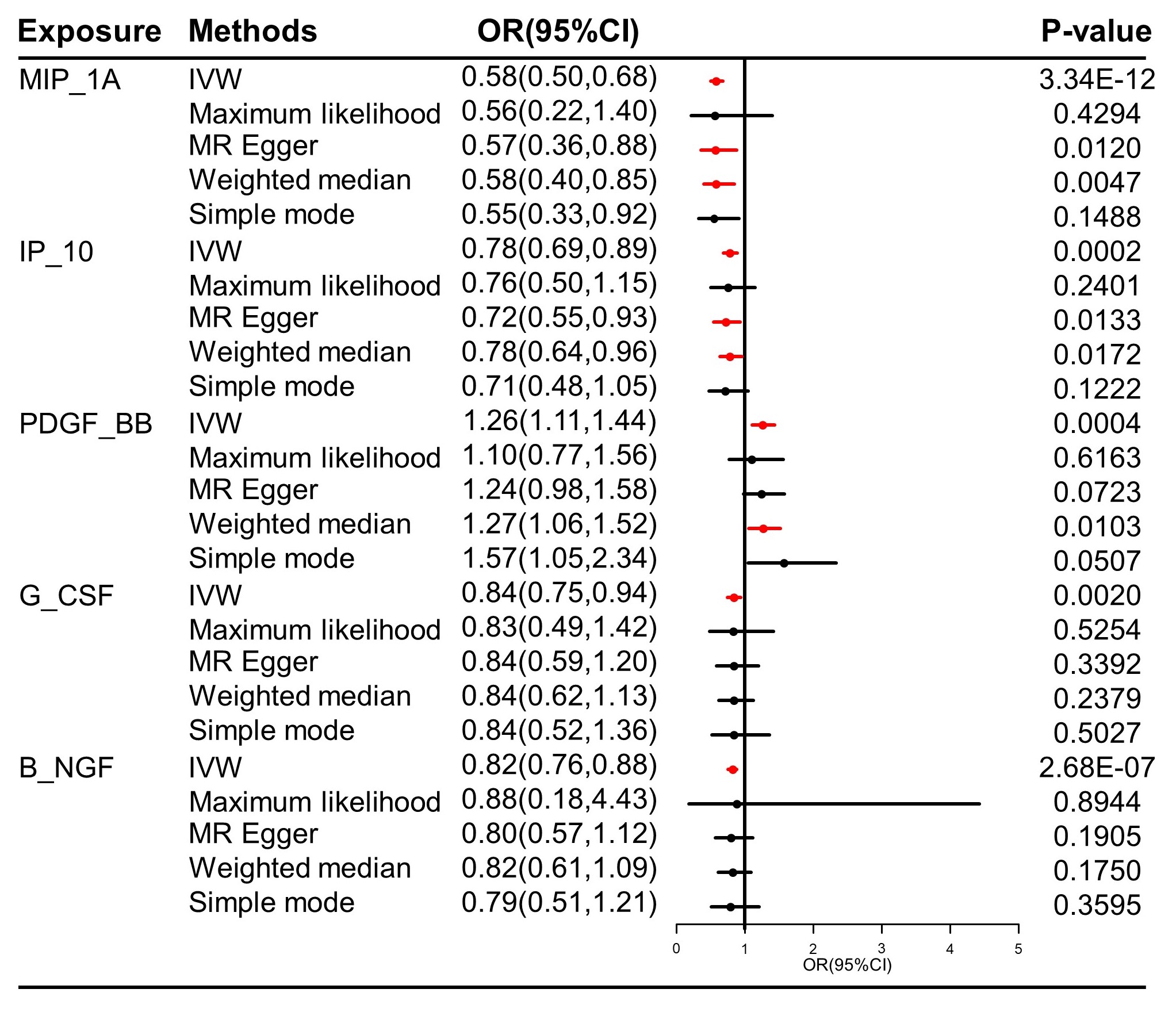


**Figure S13** Causal association between inflammatory cytokines and uIA in replicate MR analysis. uIA, unruptured intracranial aneurysm; MR, mendelian randomization.


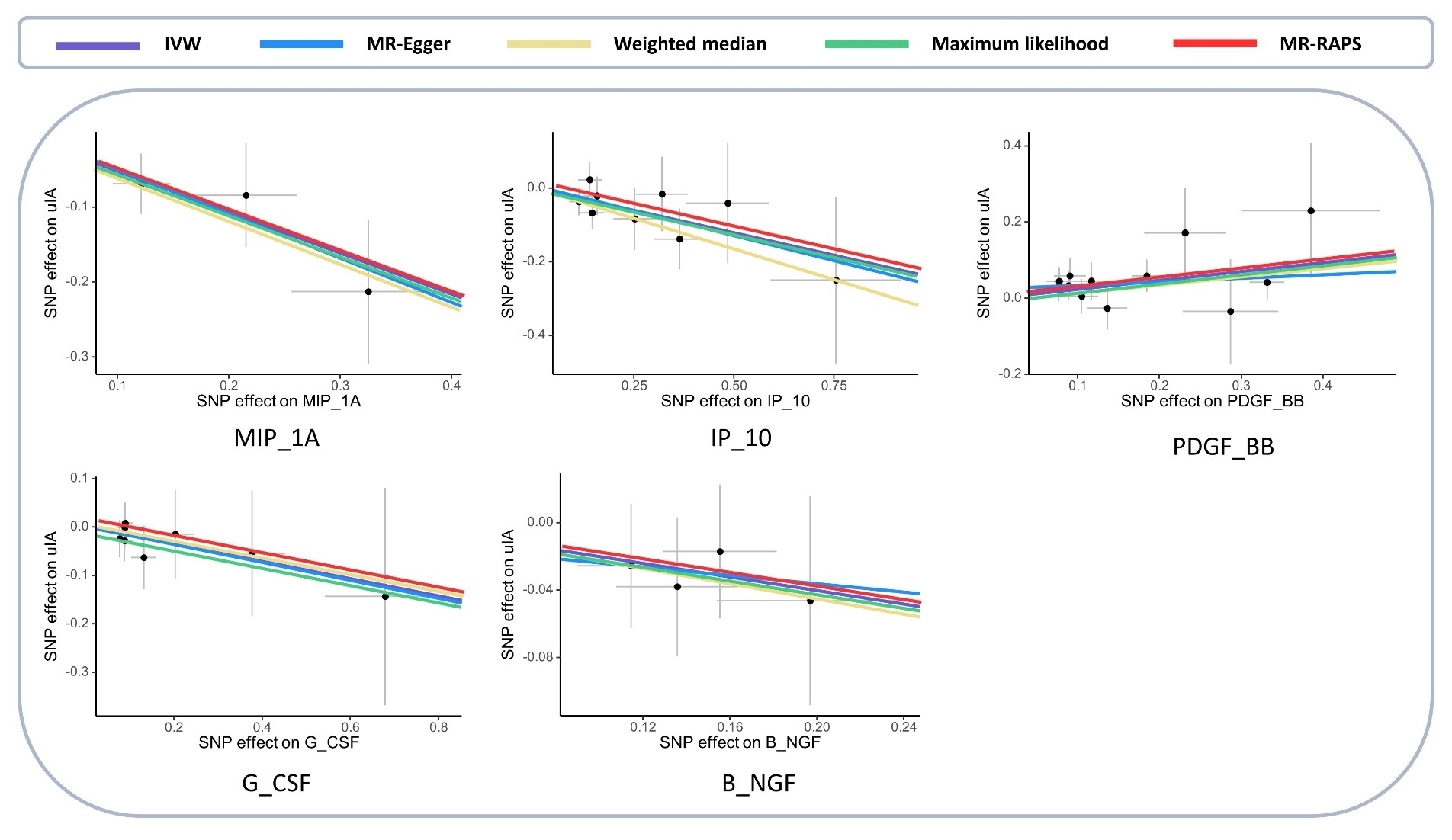


**Figure S14** The scatter plots of the association between genetically predicted inflammation cytokines on uIA in FinnGen datasets in replicate MR analysis. uIA, unruptured intracranial aneurysm; MR, mendelian randomization.


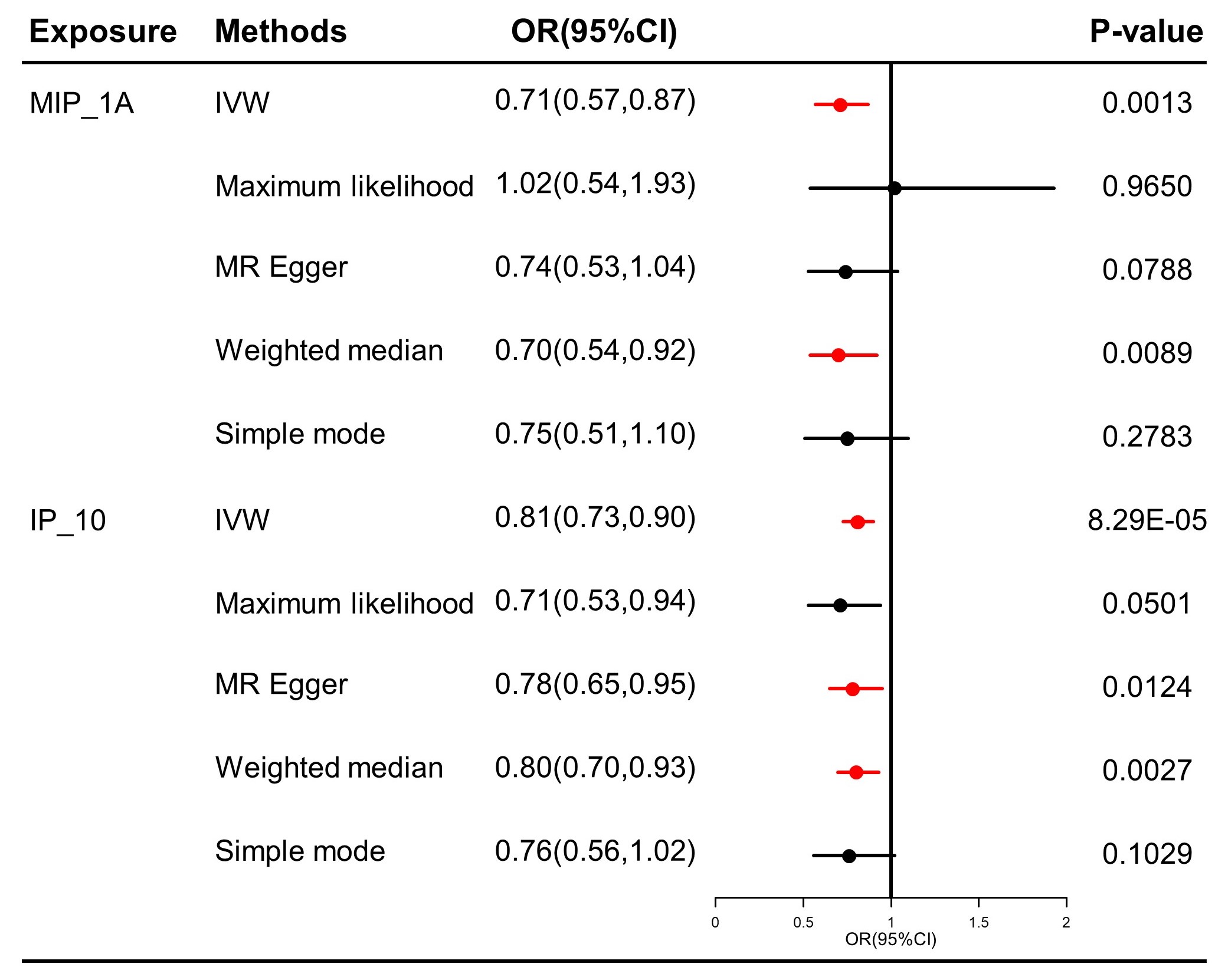


**Figure S15** Causal association between inflammatory cytokines and SAH in replicate MR analysis. SAH, subarachnoid hemorrhage; MR, mendelian randomization.


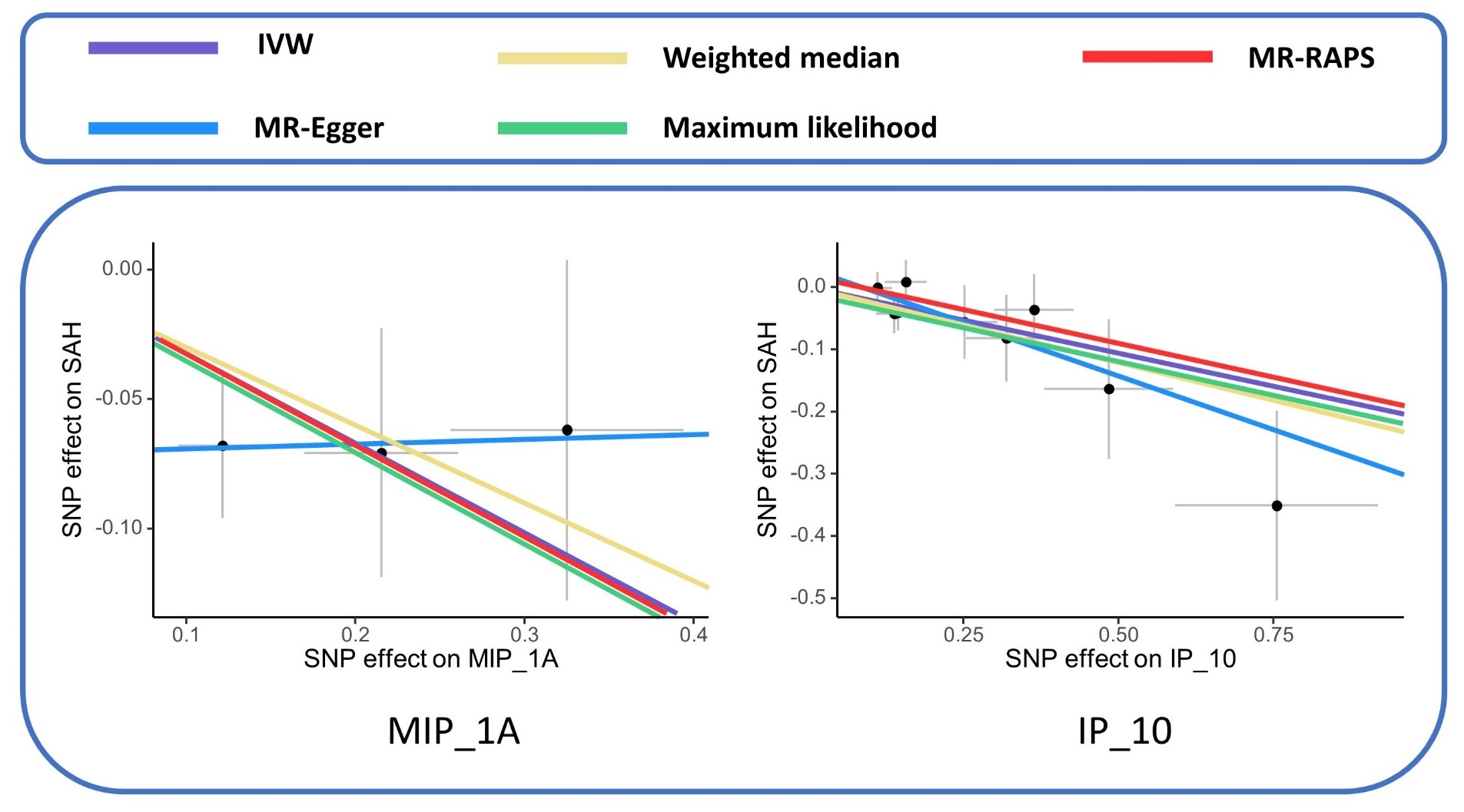


**Figure S16** The scatter plots of the association between genetically predicted inflammation cytokines on SAH in FinnGen datasets in replicate MR analysis. SAH, subarachnoid hemorrhage; MR, mendelian randomization.


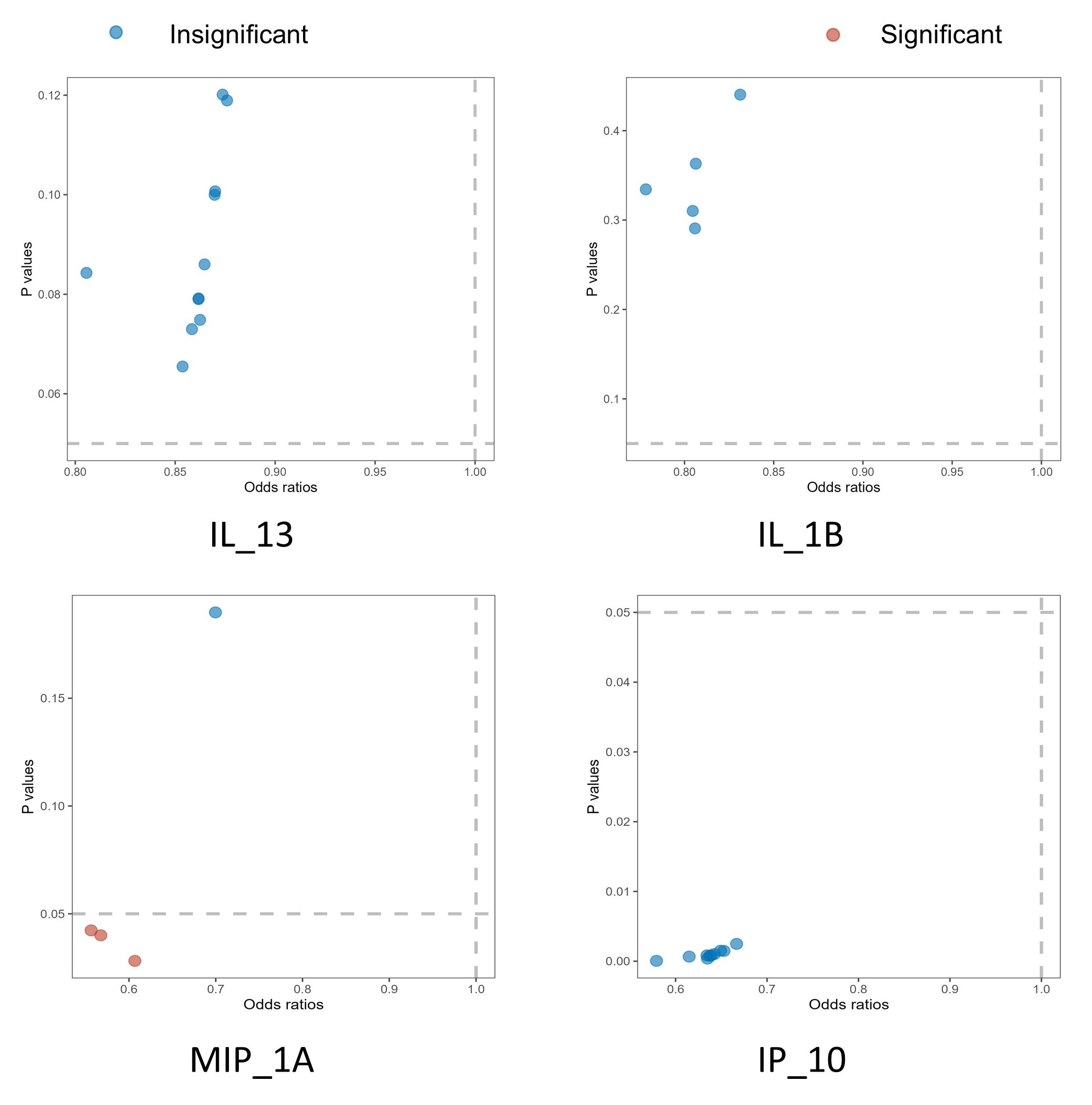


**Figure S17** The leave-one-out analysis for causal association between inflammation cytokines and IA in FinnGen datasets in replicate MR analysis. IA, intracranial aneurysm; MR, mendelian randomization.


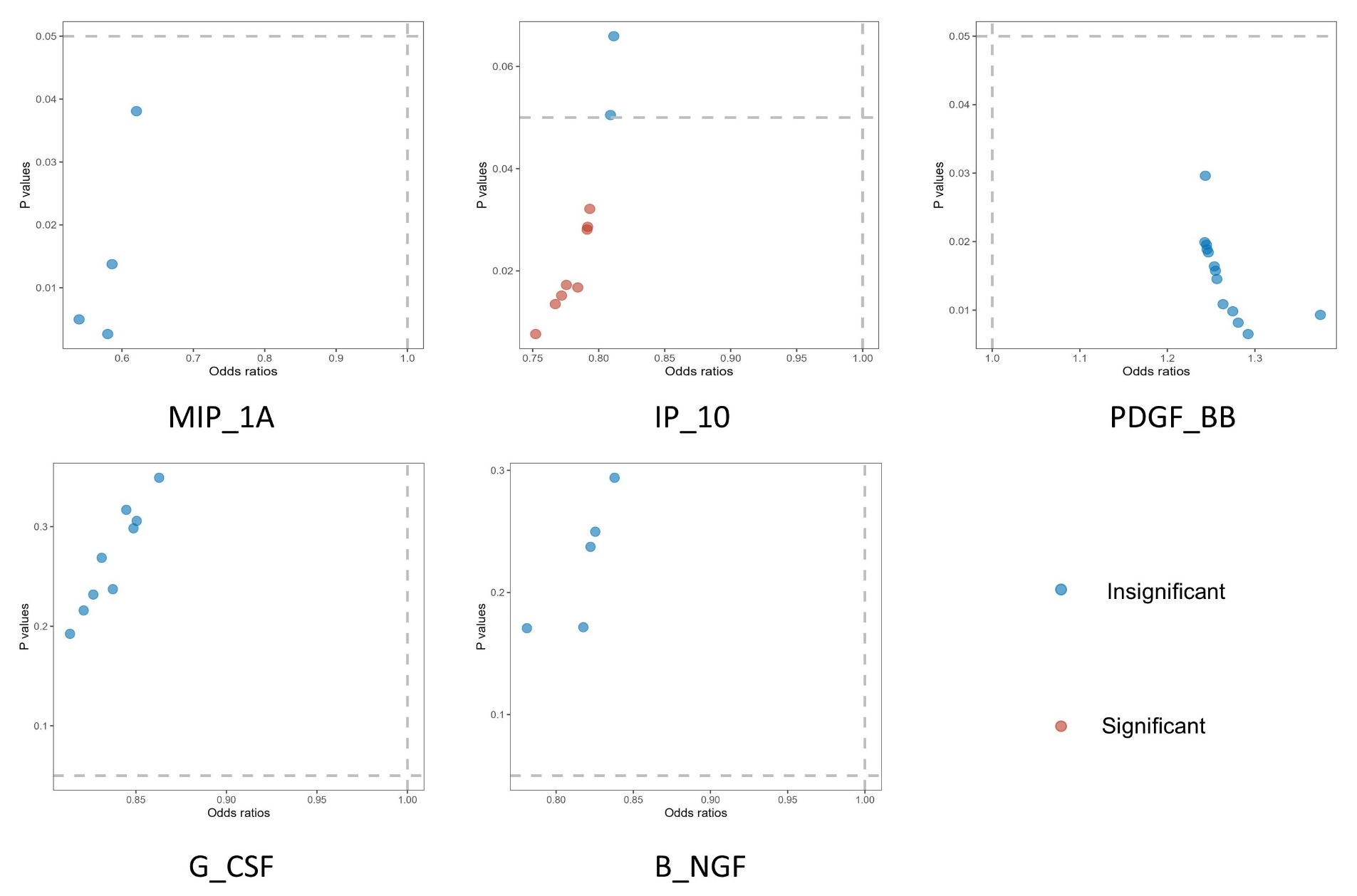


**Figure S18** The leave-one-out analysis for causal association between inflammation cytokines and uIA in FinnGen datasets in replicate MR analysis. uIA, unruptured intracranial aneurysm; MR, mendelian randomization.


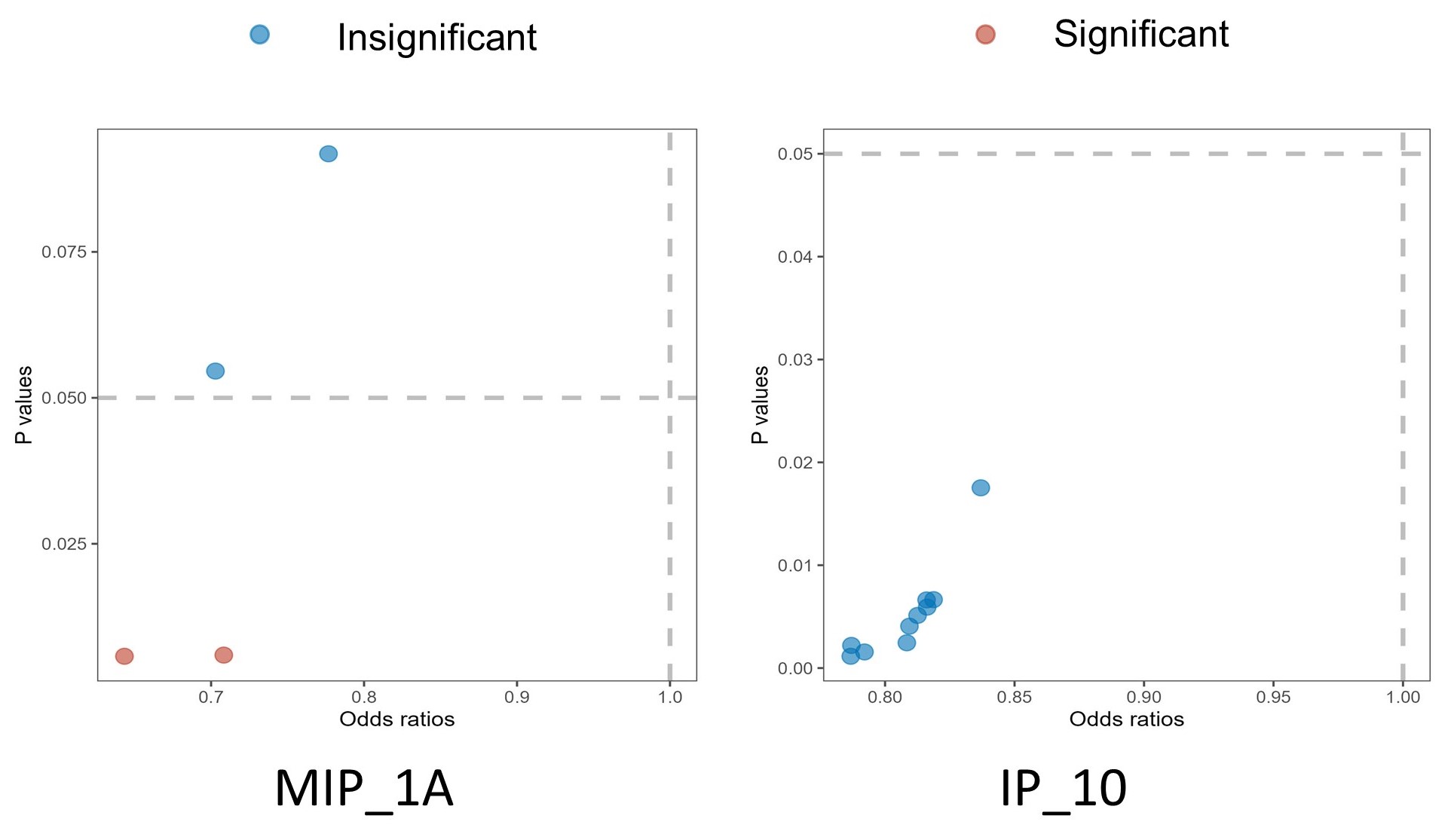


**Figure S19** The leave-one-out analysis for causal association between inflammation cytokines and SAH in FinnGen datasets in replicate MR analysis. SAH, subarachnoid hemorrhage; MR, mendelian randomization.


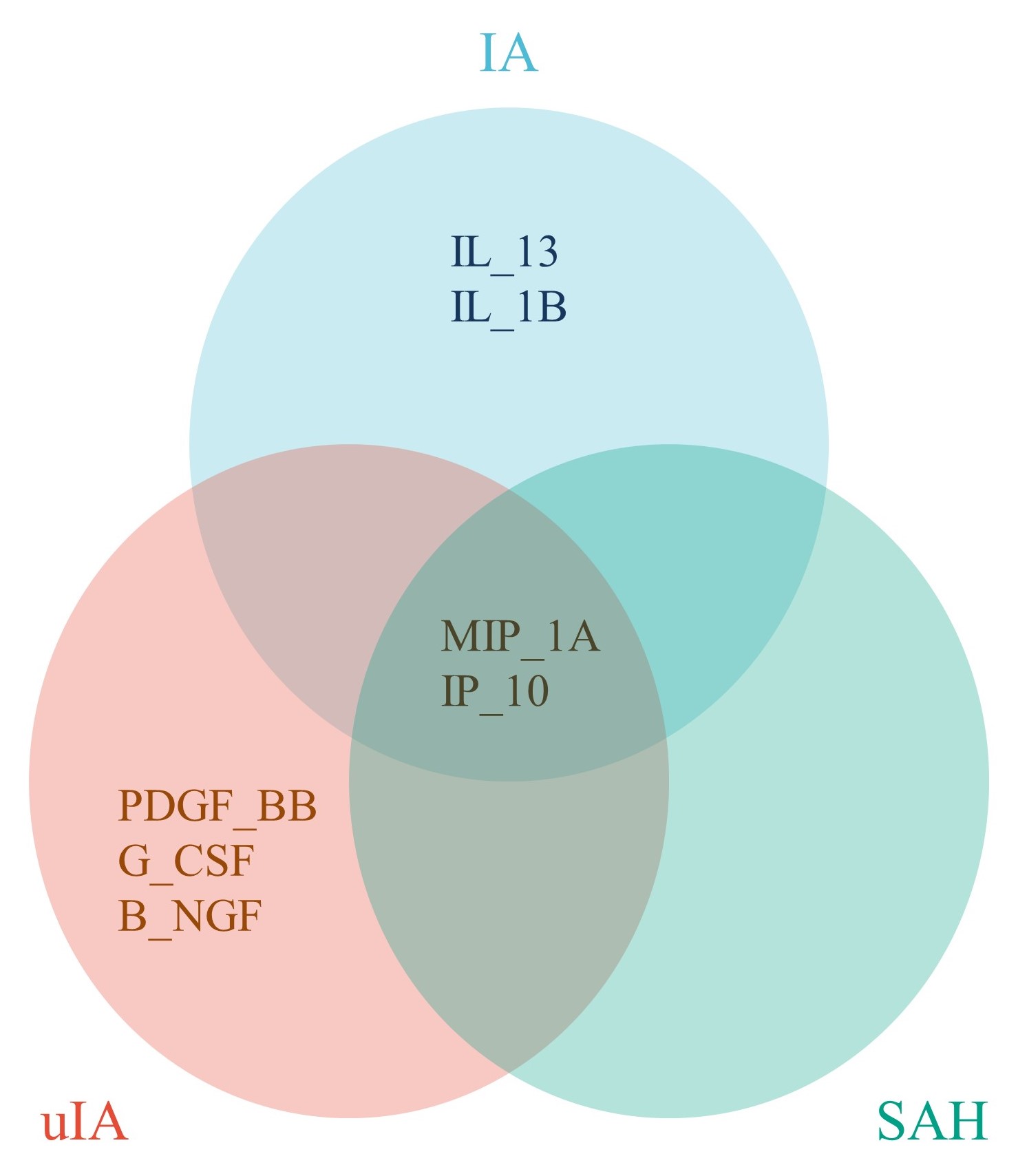


**Figure S20** The common inflammation cytokines among IA, uIA, and SAH in FinnGen datasets in replicate MR analysis. IA, intracranial aneurysm; uIA, unruptured intracranial aneurysm; SAH, subarachnoid hemorrhage; MR, mendelian randomization.


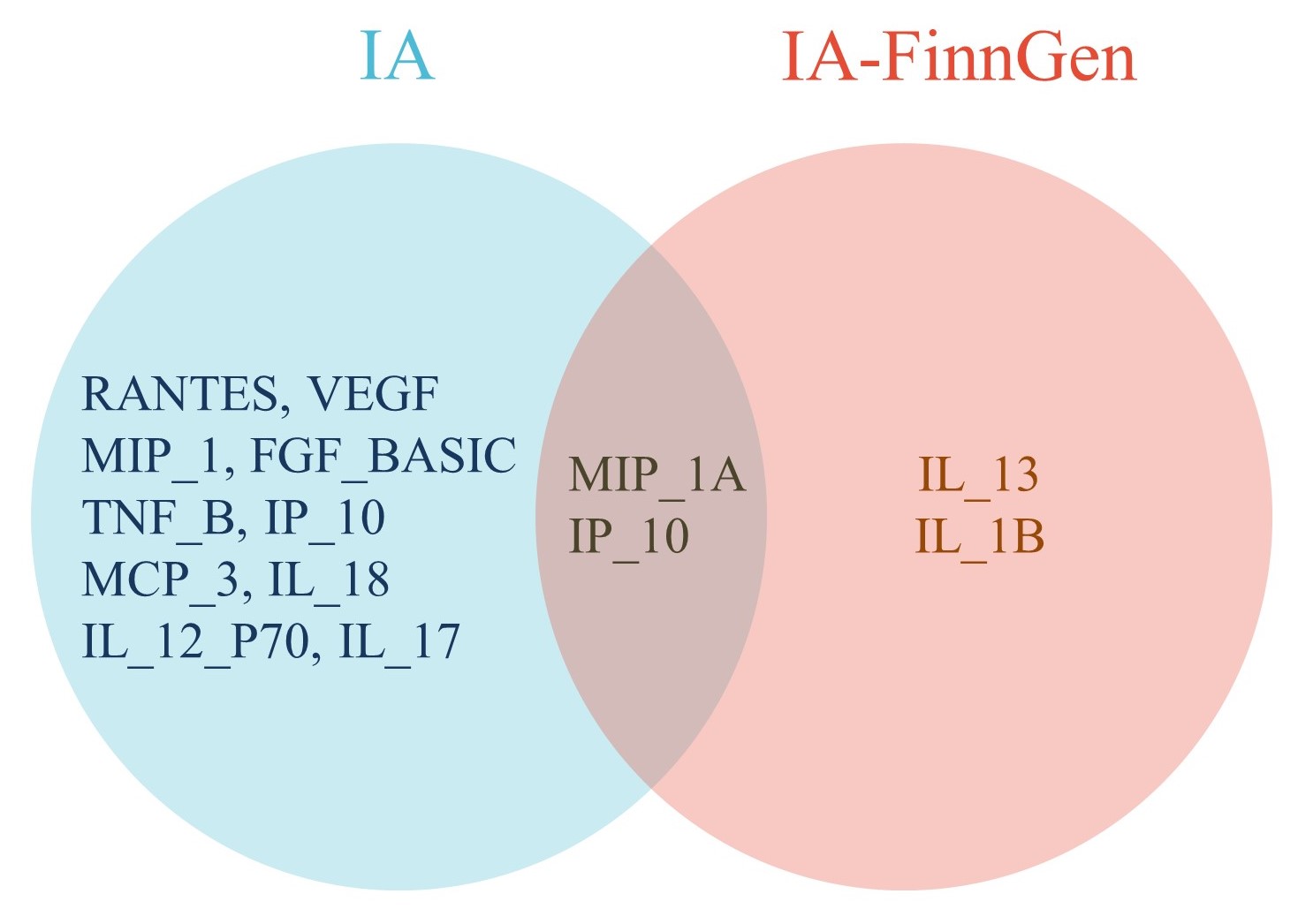


**Figure S21** The common inflammation cytokines for IA between MR and replicate MR analysis. IA, intracranial aneurysm; MR, mendelian randomization.


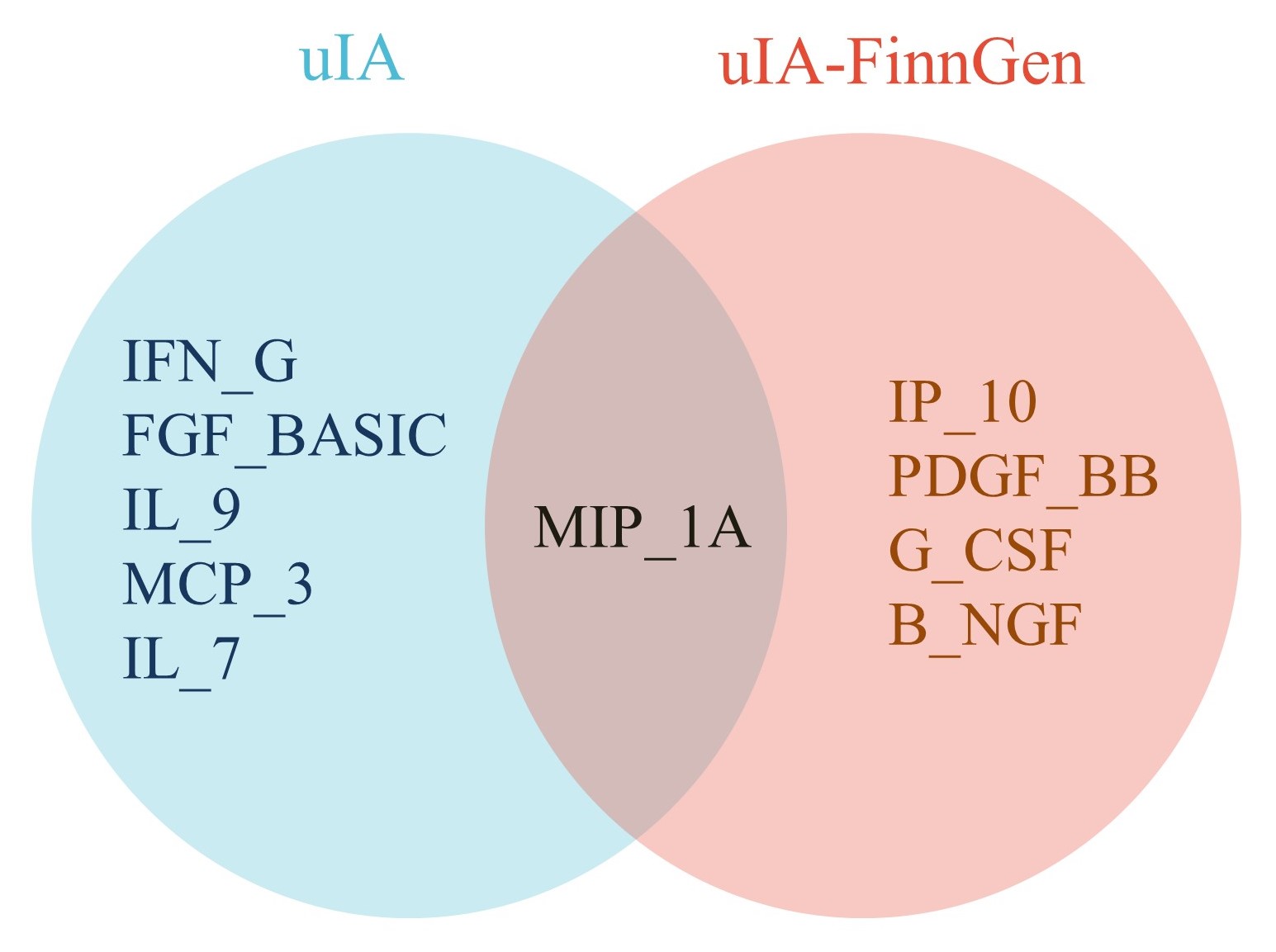


**Figure S22** The common inflammation cytokines for uIA between MR and replicate MR analysis. uIA, unruptured intracranial aneurysm; MR, mendelian randomization.


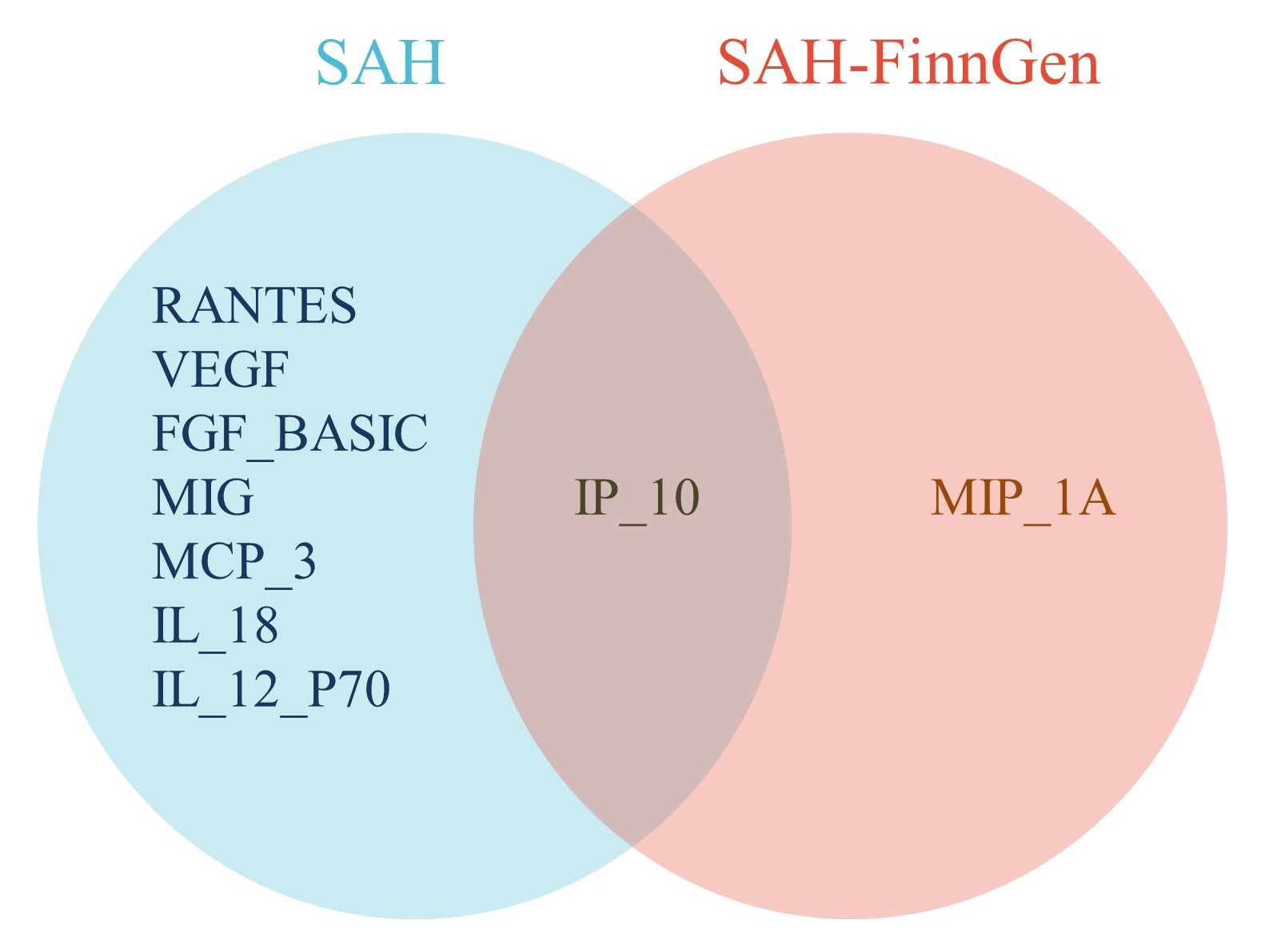


**Figure S23** The common inflammation cytokines for SAH between MR and replicate MR analysis. SAH, subarachnoid hemorrhage; MR, mendelian randomization.


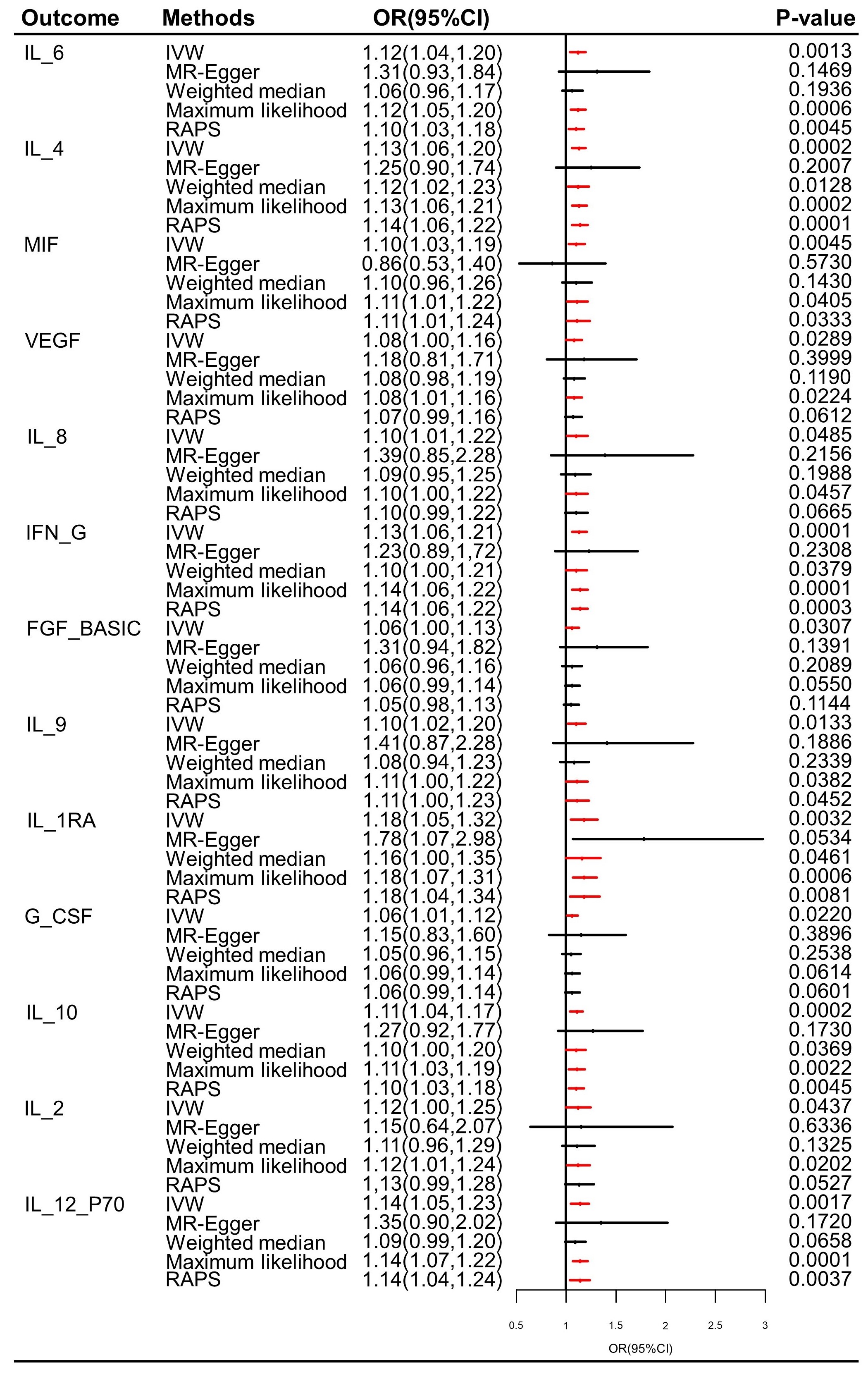


**Figure S24** Causal association between inflammatory cytokines and IA in reverse MR analysis. IA, intracranial aneurysm; MR, mendelian randomization.


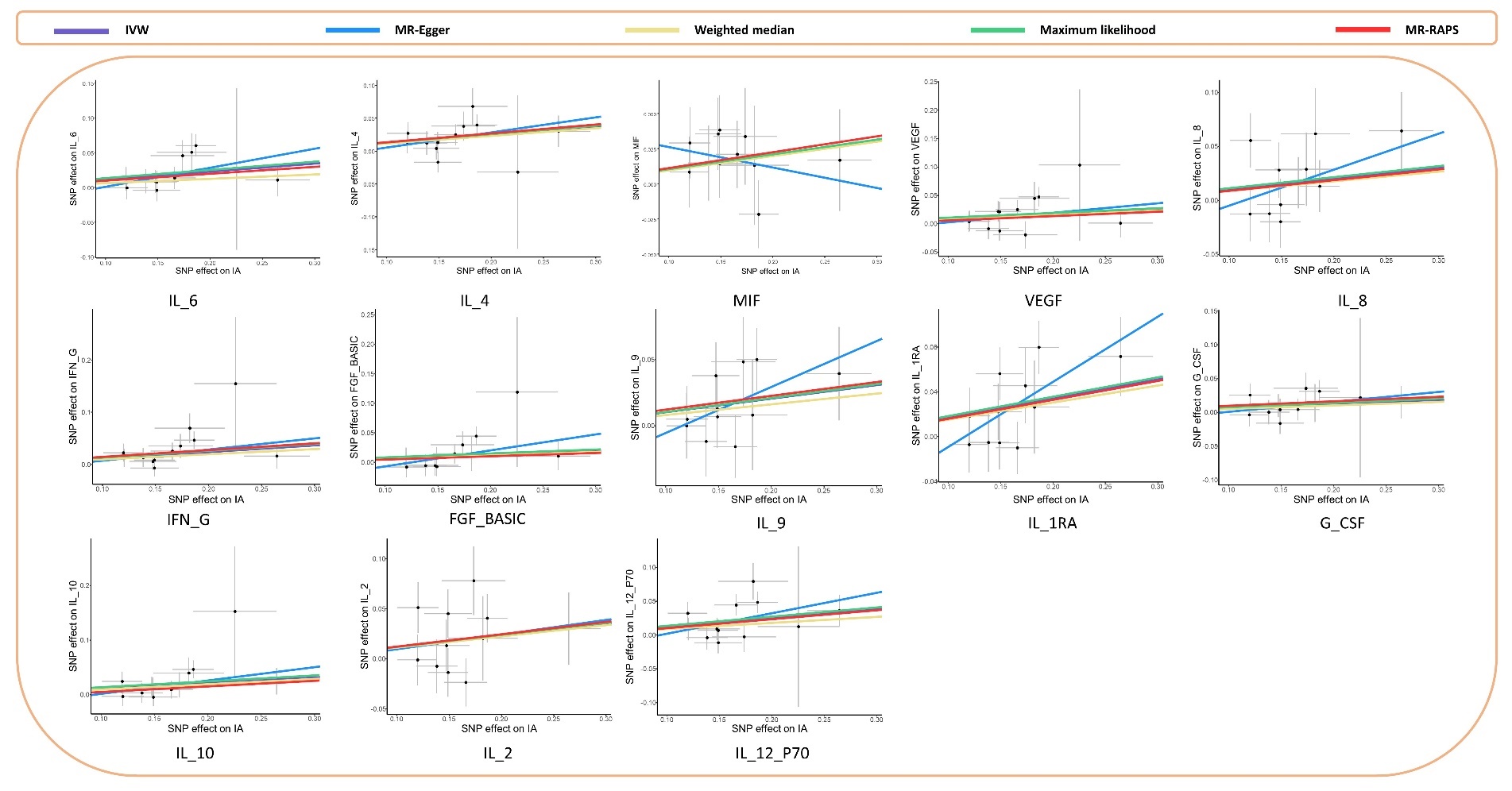


**Figure S25** The scatter plots of the association between genetically predicted IA on inflammation cytokines in reverse MR analysis. IA, intracranial aneurysm; MR, mendelian randomization.


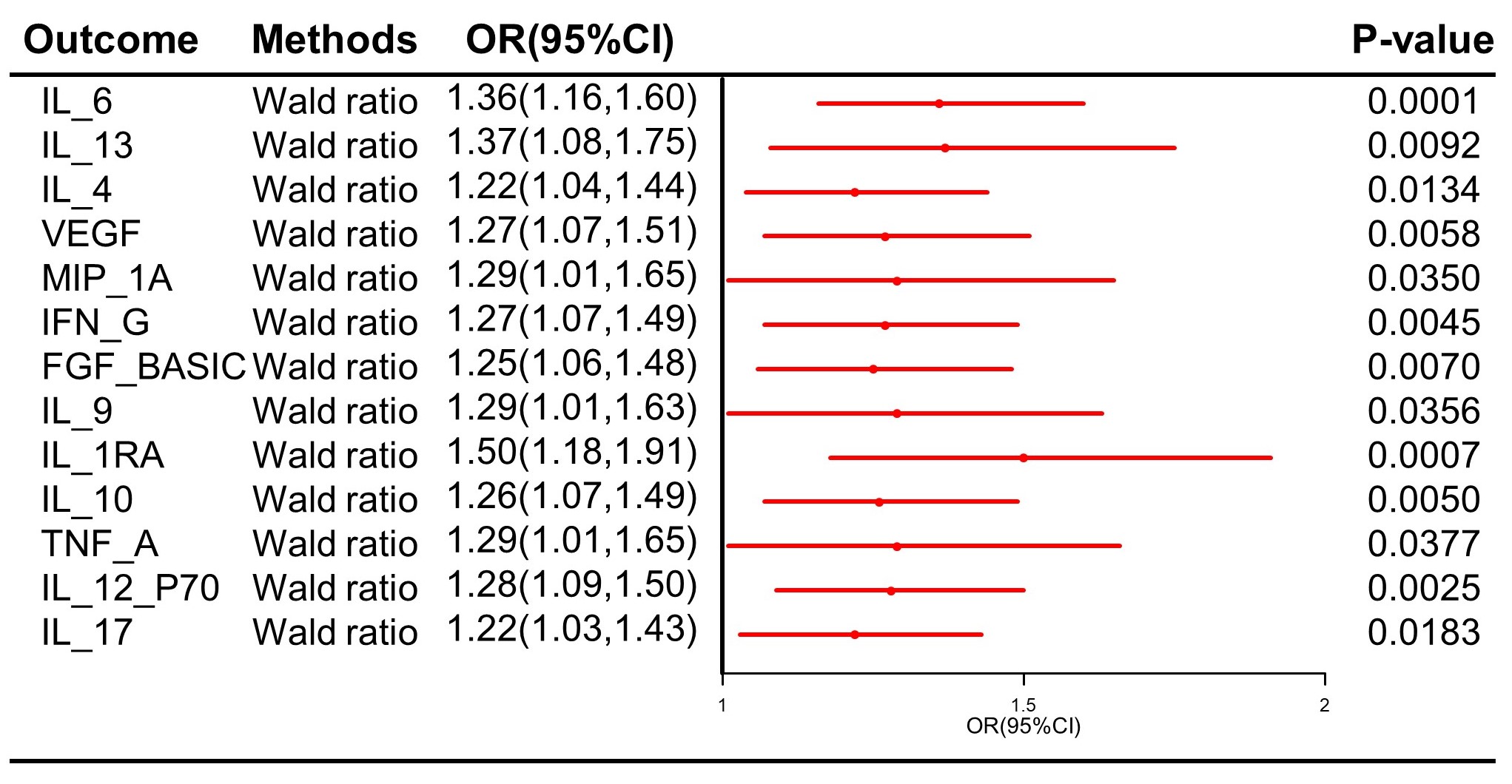


**Figure S26** Causal association between inflammatory cytokines and uIA in reverse MR analysis. uIA, unruptured intracranial aneurysm; MR, mendelian randomization.


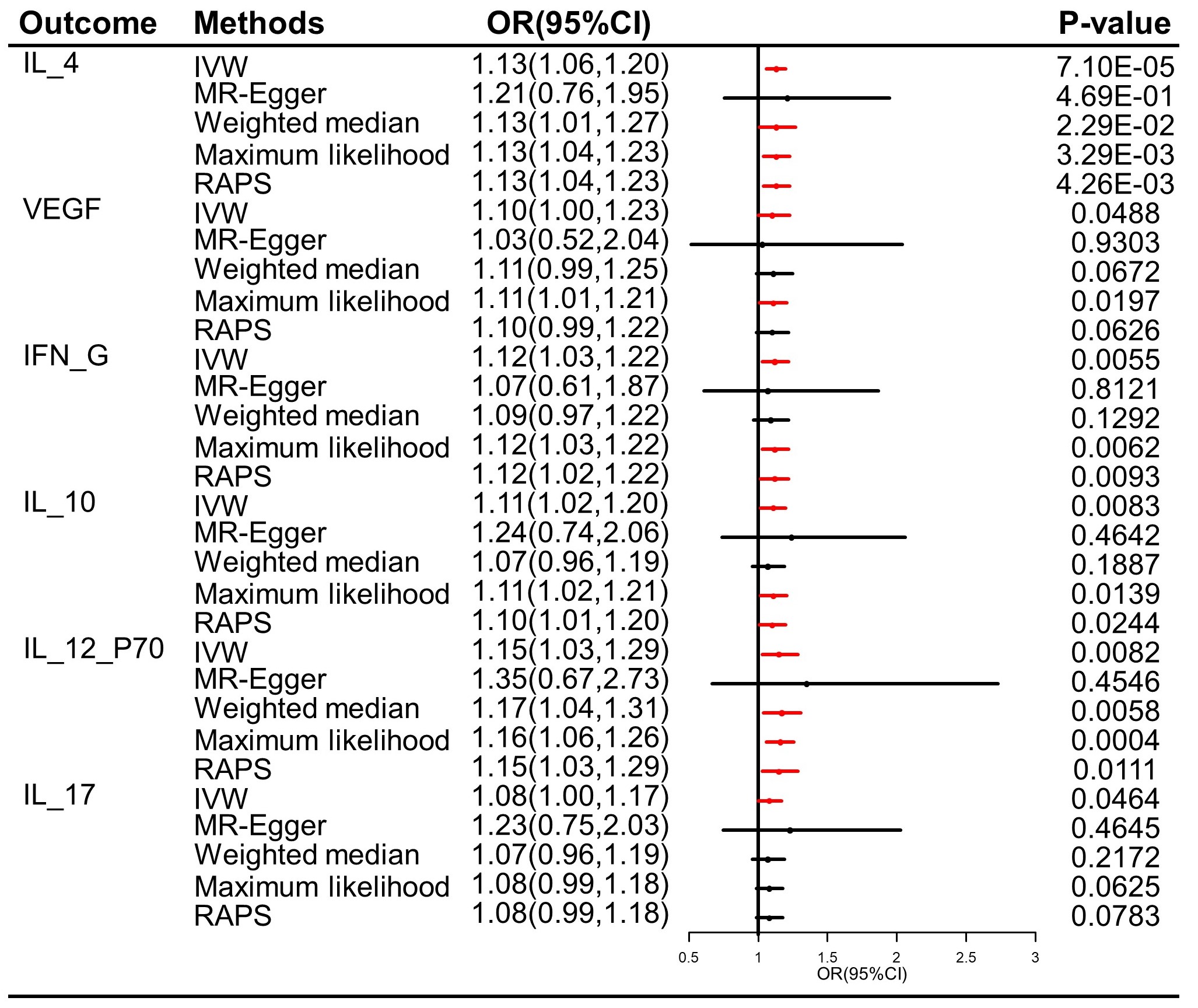


**Figure S27** Causal association between inflammatory cytokines and SAH in reverse MR analysis. SAH, subarachnoid hemorrhage; MR, mendelian randomization.


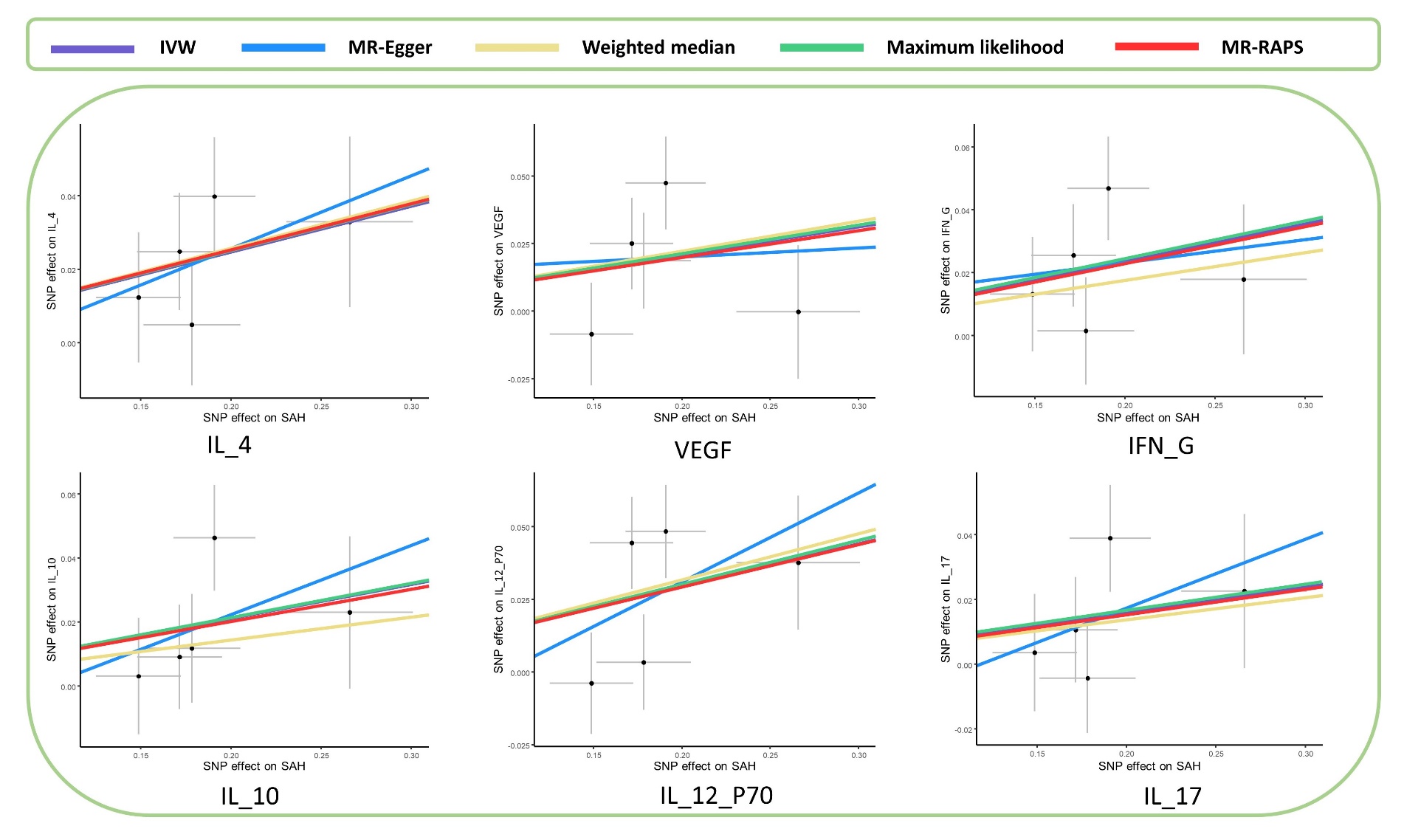


**Figure S28** The scatter plots of the association between genetically predicted SAH on inflammation cytokines in reverse MR analysis. SAH, subarachnoid hemorrhage; MR, mendelian randomization.


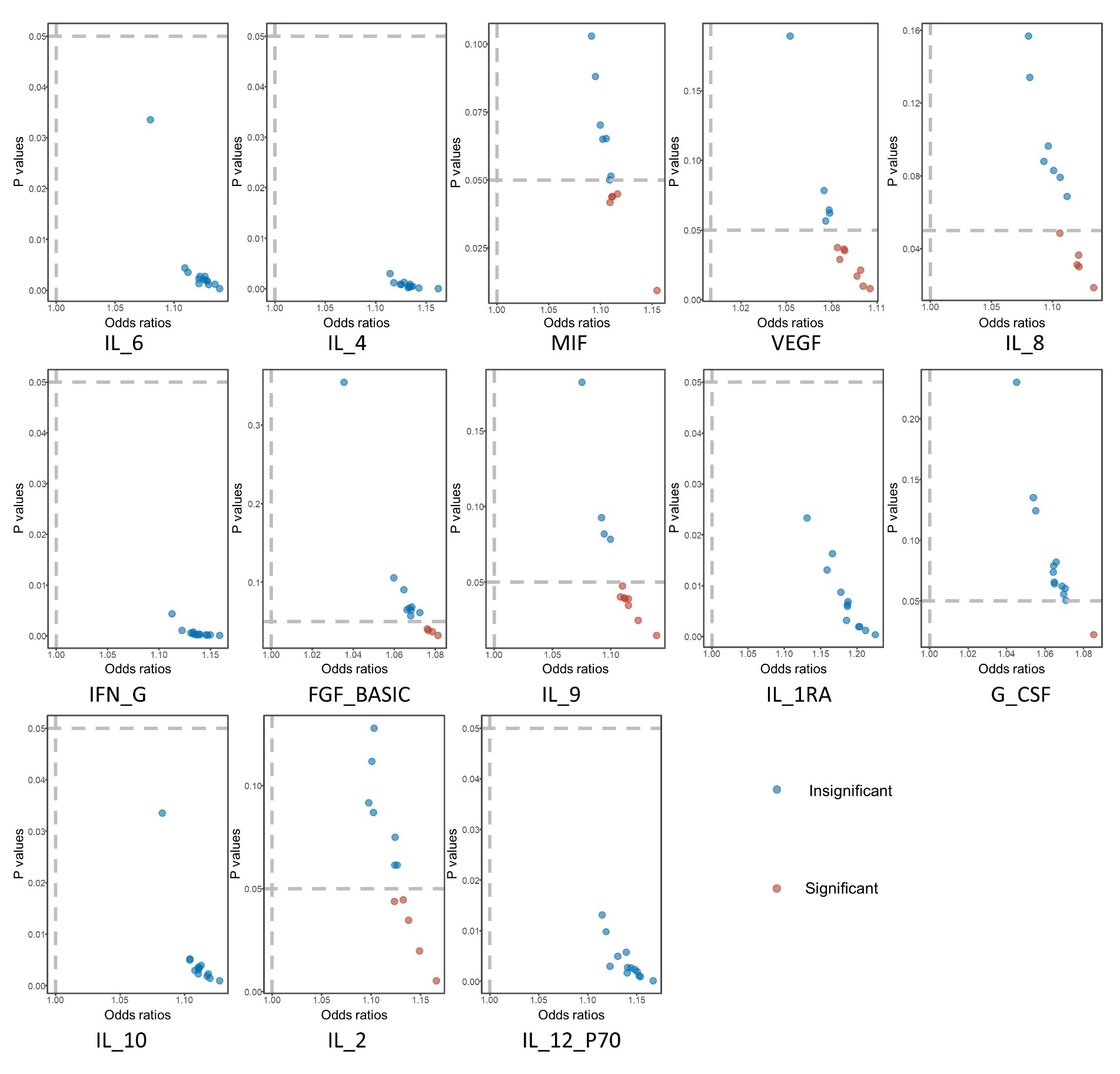


**Figure S29** The leave-one-out analysis for causal association between IA and inflammation cytokines in reverse MR analysis. IA, intracranial aneurysm; MR, mendelian randomization.


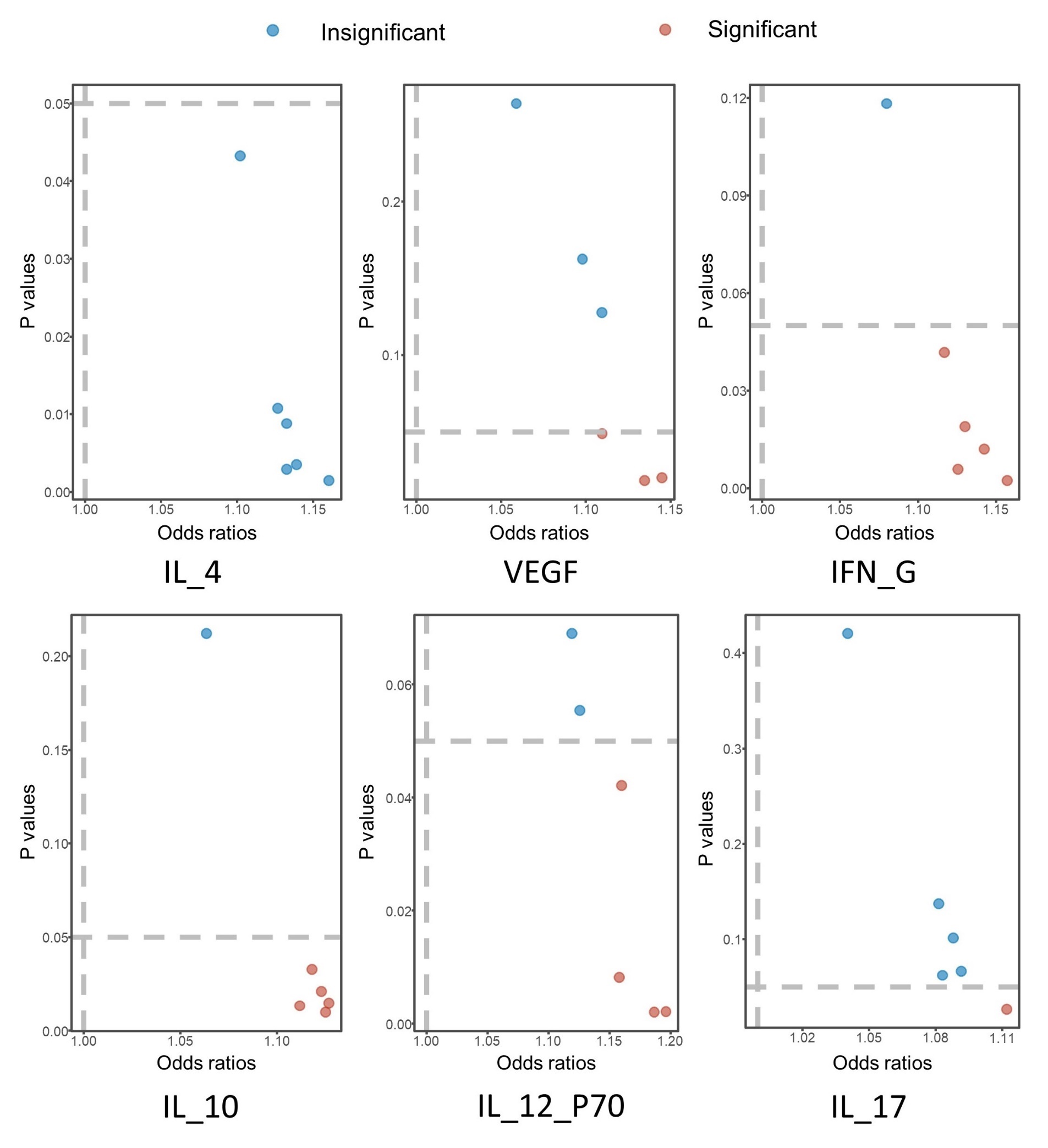


**Figure S30** The leave-one-out analysis for causal association between SAH and inflammation cytokines in reverse MR analysis. SAH, subarachnoid hemorrhage; MR, mendelian randomization.


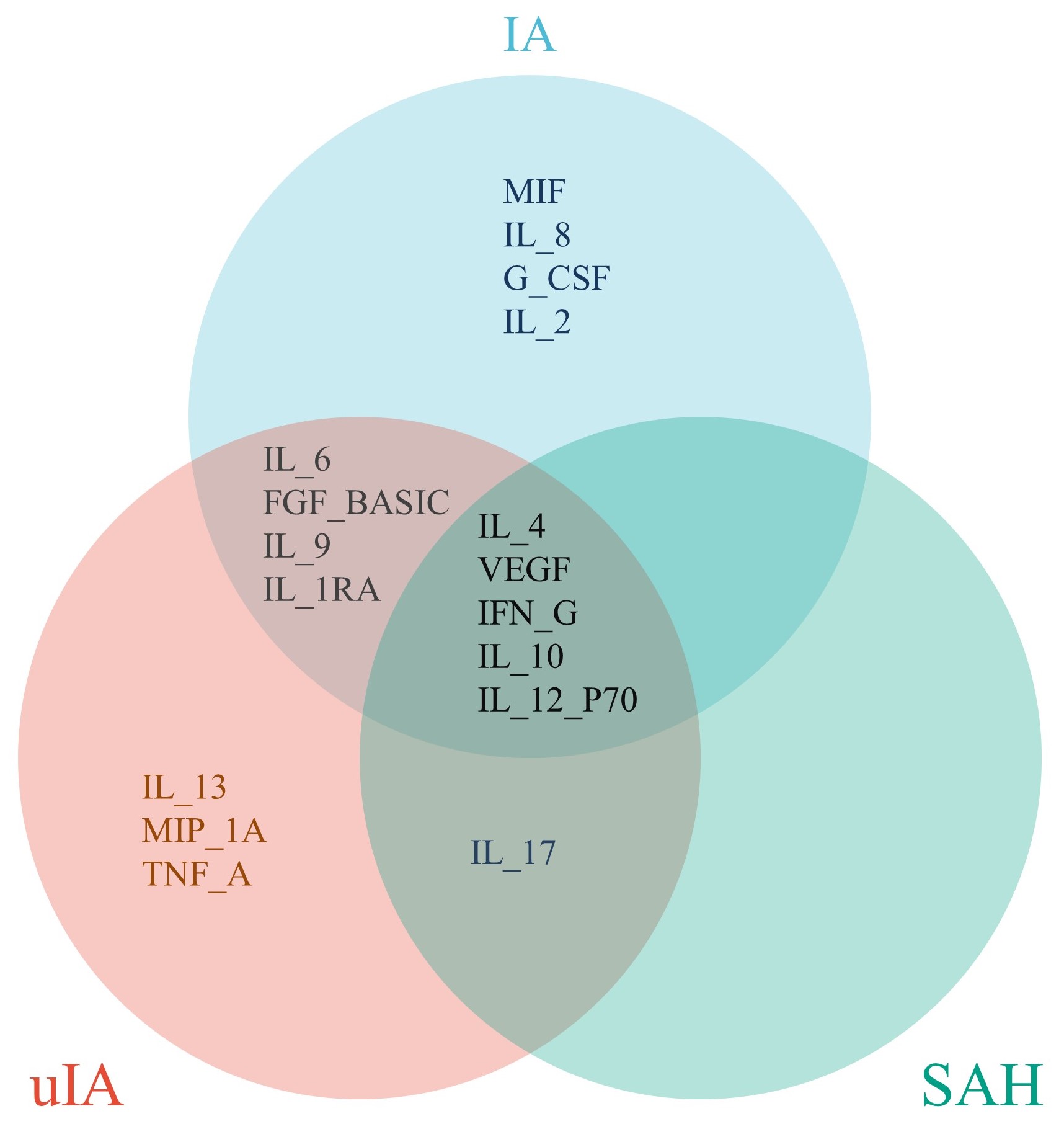


**Figure S31** The common inflammation cytokines among IA, uIA, and SAH in reverse MR analysis. IA, intracranial aneurysm; uIA, unruptured intracranial aneurysm; SAH, subarachnoid hemorrhage; MR, mendelian randomization.


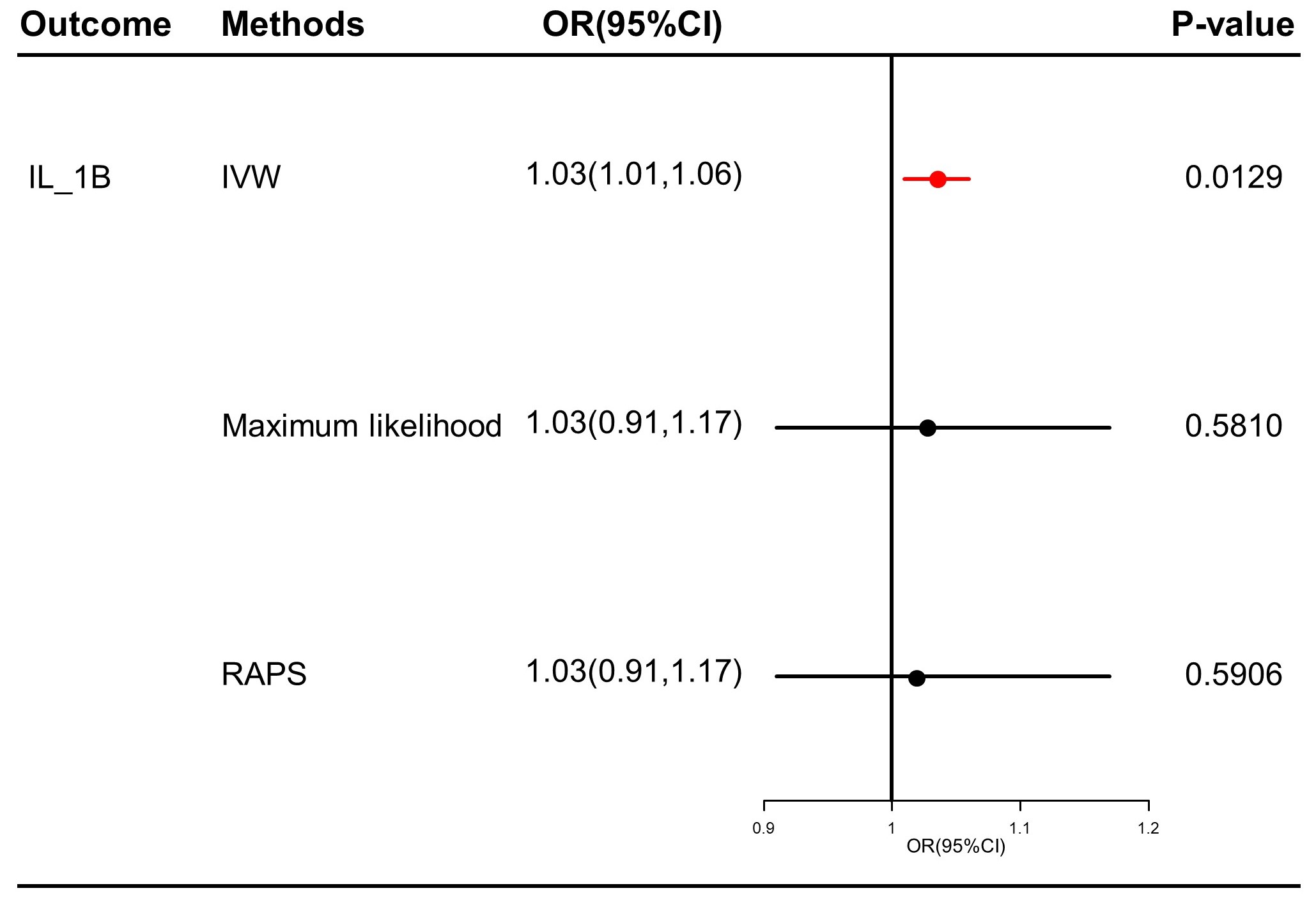


**Figure S32** Causal association between inflammatory cytokines and IA in FinnGen datasets in replicate reverse MR analysis. IA, intracranial aneurysm; MR, mendelian randomization.


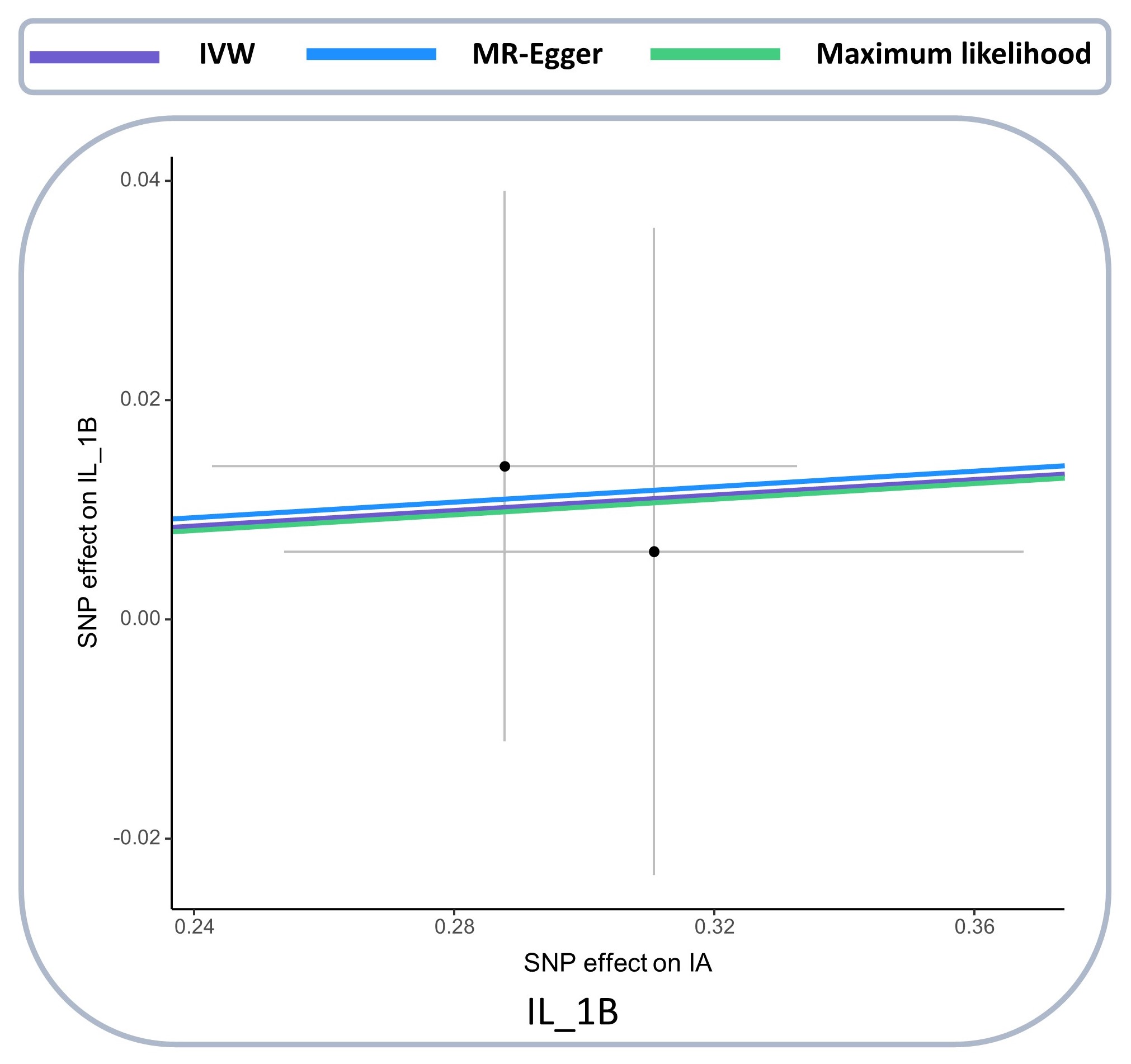


**Figure S33** The scatter plots of the association between genetically predicted IA on inflammation cytokines in FinnGen datasets in replicate reverse MR analysis. IA, intracranial aneurysm; MR, mendelian randomization.


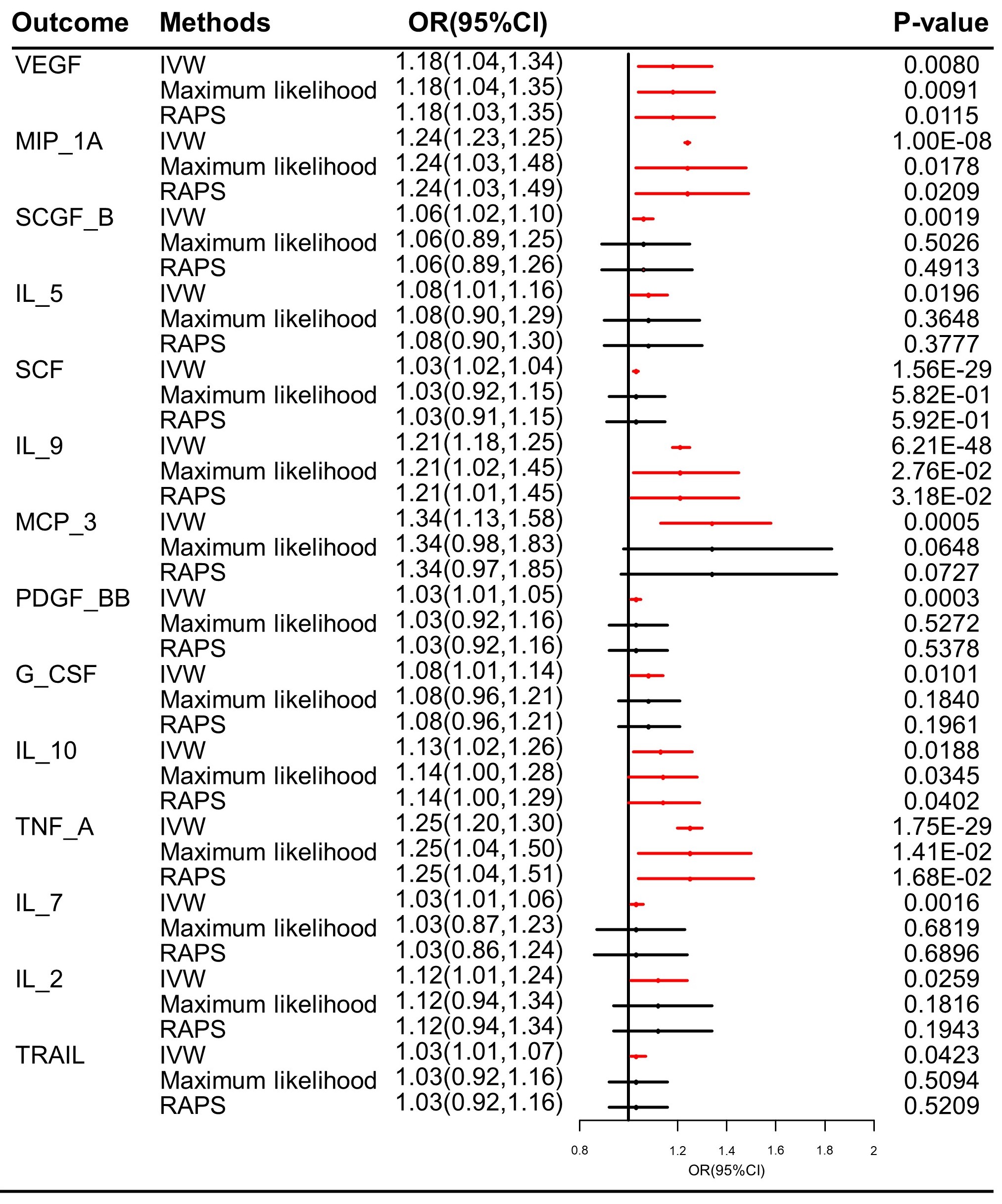


**Figure S34** Causal association between inflammatory cytokines and uIA in FinnGen datasets in replicate reverse MR analysis. uIA, unruptured intracranial aneurysm; MR, mendelian randomization.


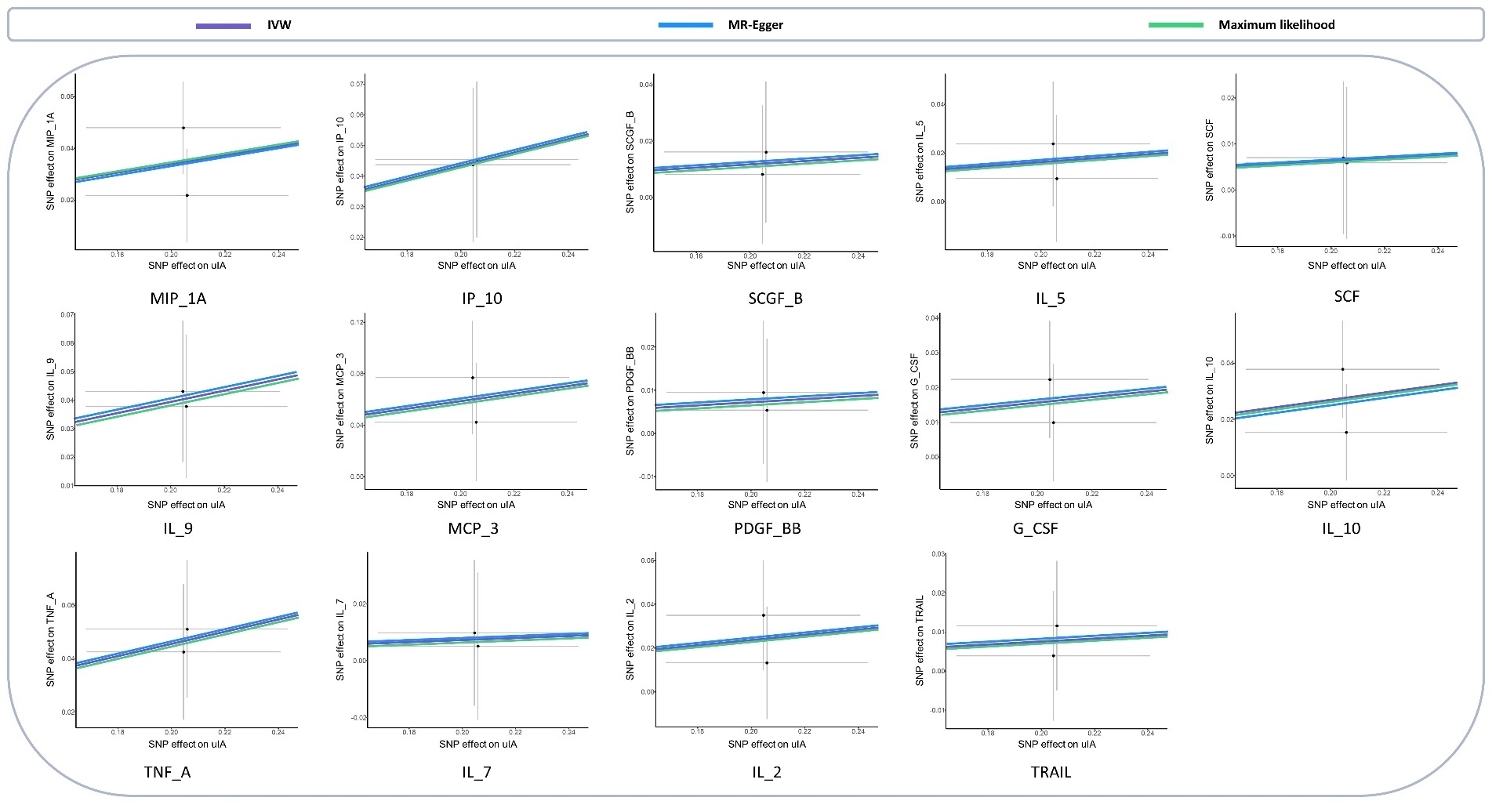


**Figure S35** The scatter plots of the association between genetically predicted uIA on inflammation cytokines in FinnGen datasets in replicate reverse MR analysis. uIA, unruptured intracranial aneurysm; MR, mendelian randomization.


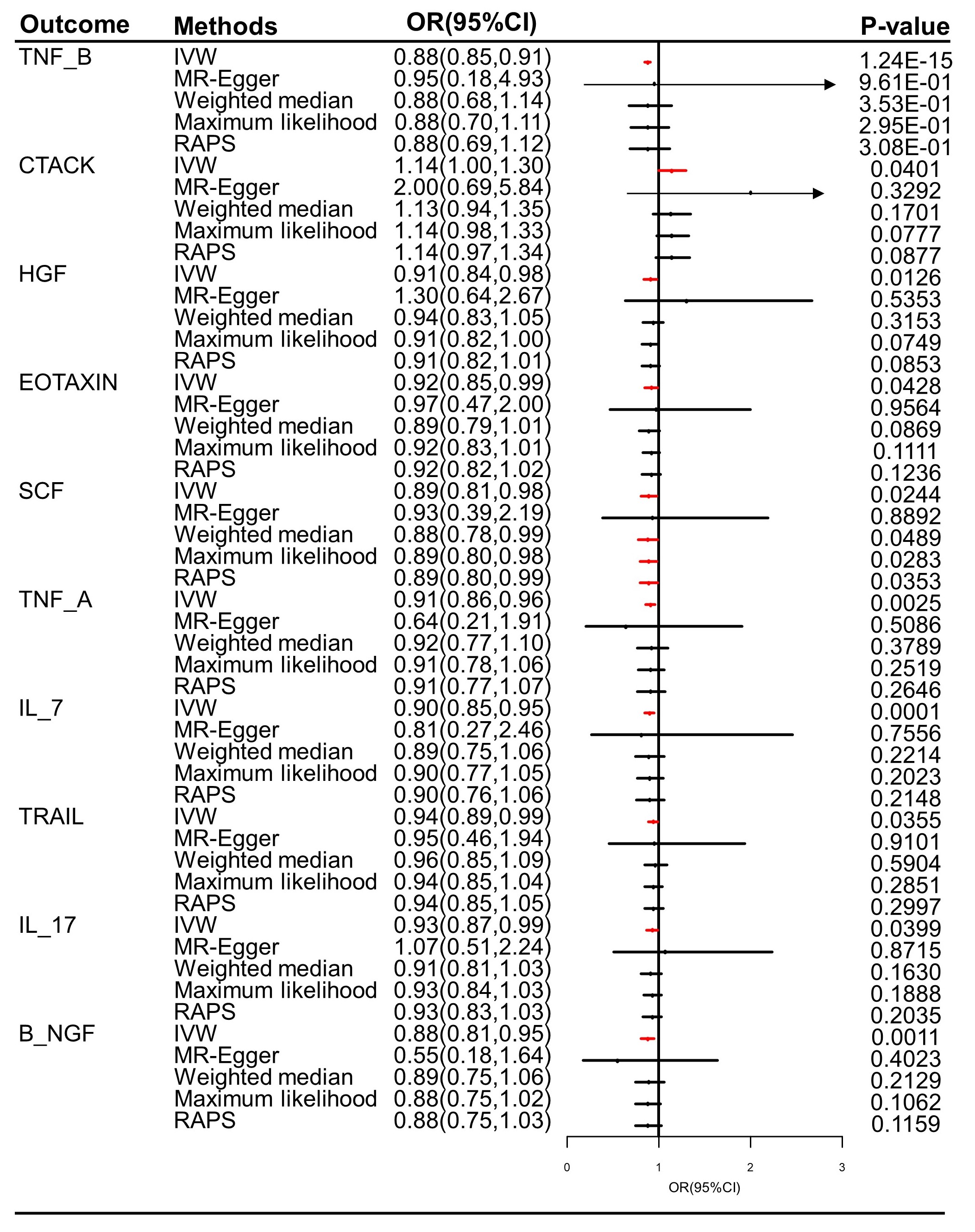


**Figure S36** Causal association between inflammatory cytokines and SAH in FinnGen datasets in replicate reverse MR analysis. SAH, subarachnoid hemorrhage; MR, mendelian randomization.


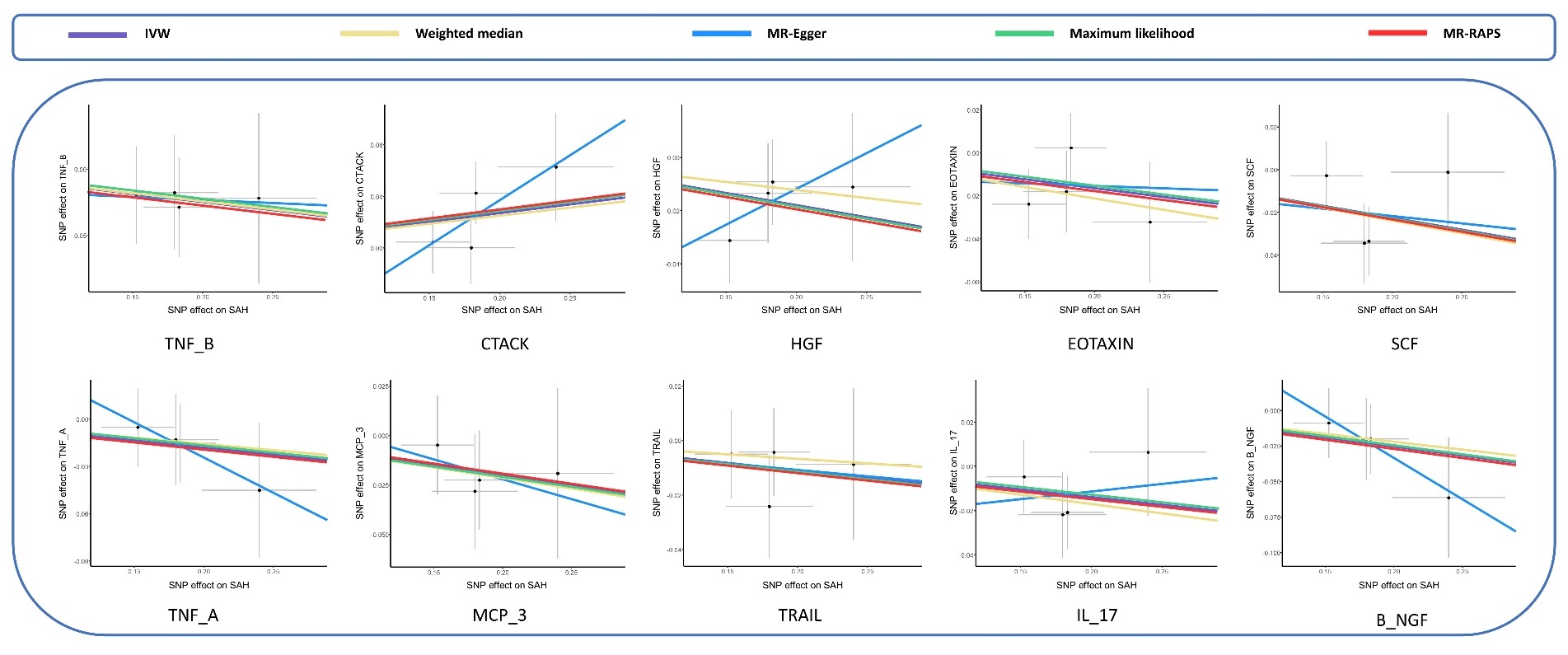


**Figure S37** The scatter plots of the association between genetically predicted SAH on inflammation cytokines in FinnGen datasets in replicate reverse MR analysis. SAH, subarachnoid hemorrhage; MR, mendelian randomization.


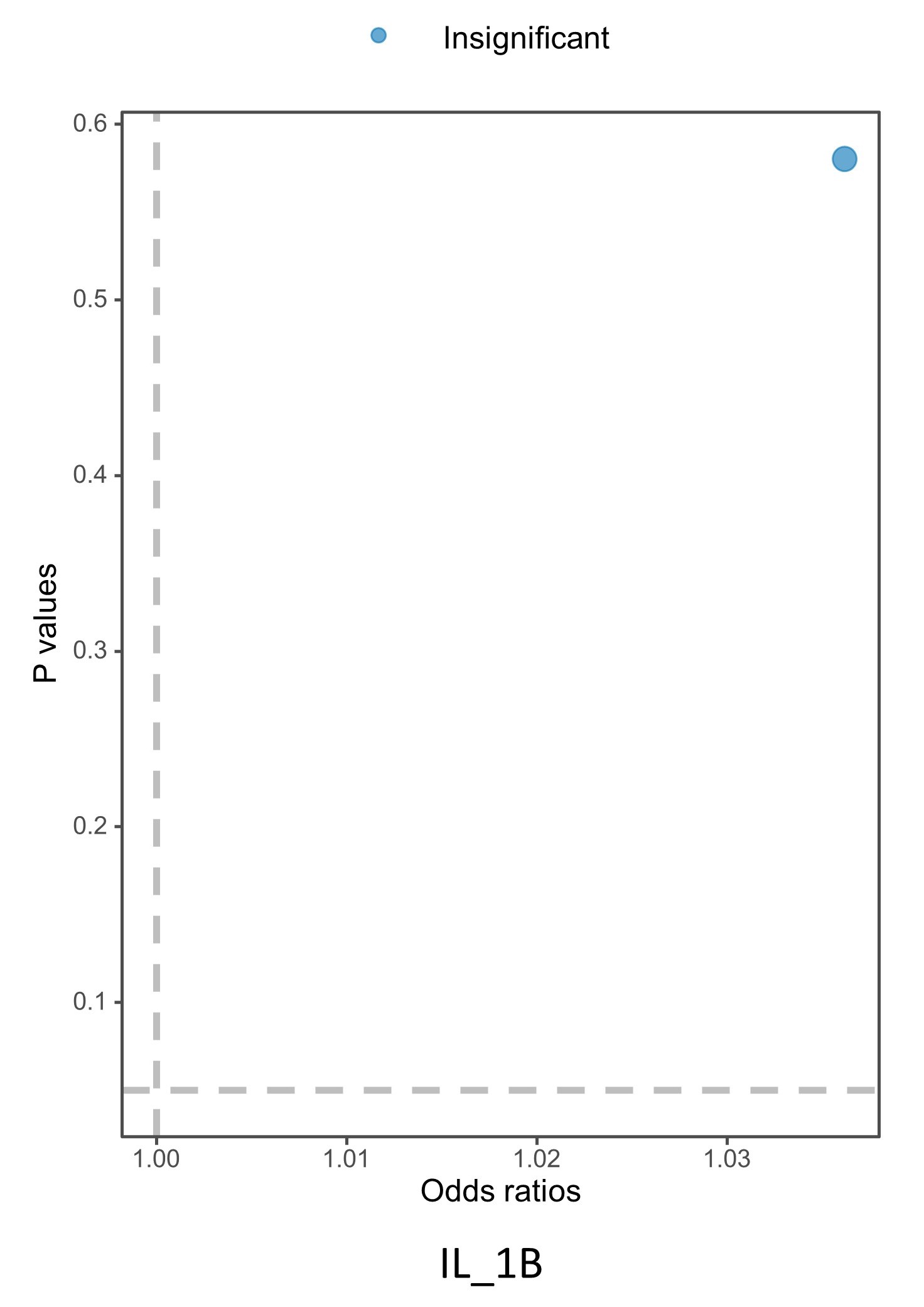


**Figure S38** The leave-one-out analysis for causal association between IA and inflammation cytokines in FinnGen datasets in replicate reverse MR analysis. IA, intracranial aneurysm; MR, mendelian randomization.


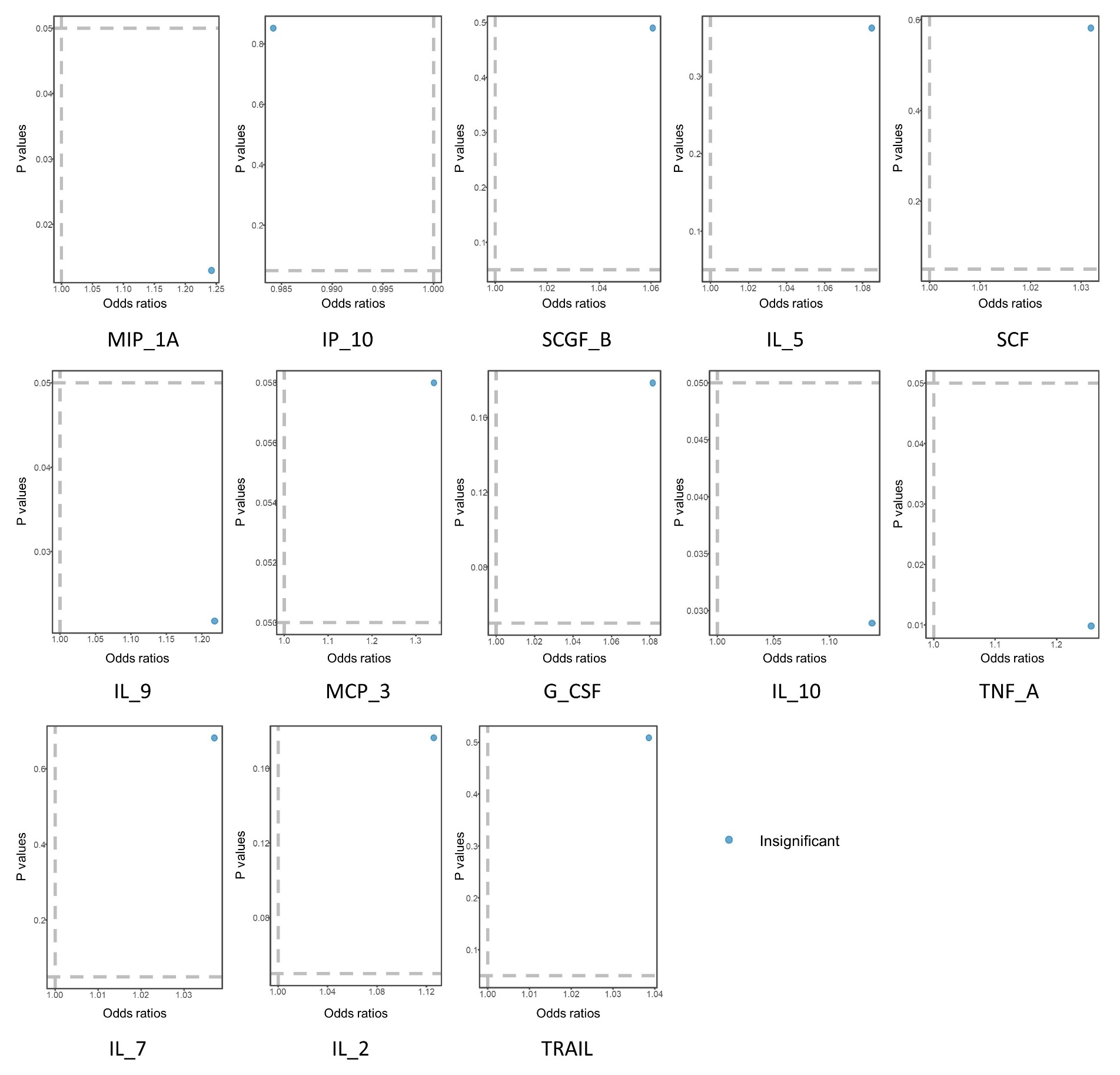


**Figure S39** The leave-one-out analysis for causal association between uIA and inflammation cytokines in FinnGen datasets in replicate reverse MR analysis. uIA, unruptured intracranial aneurysm; MR, mendelian randomization.


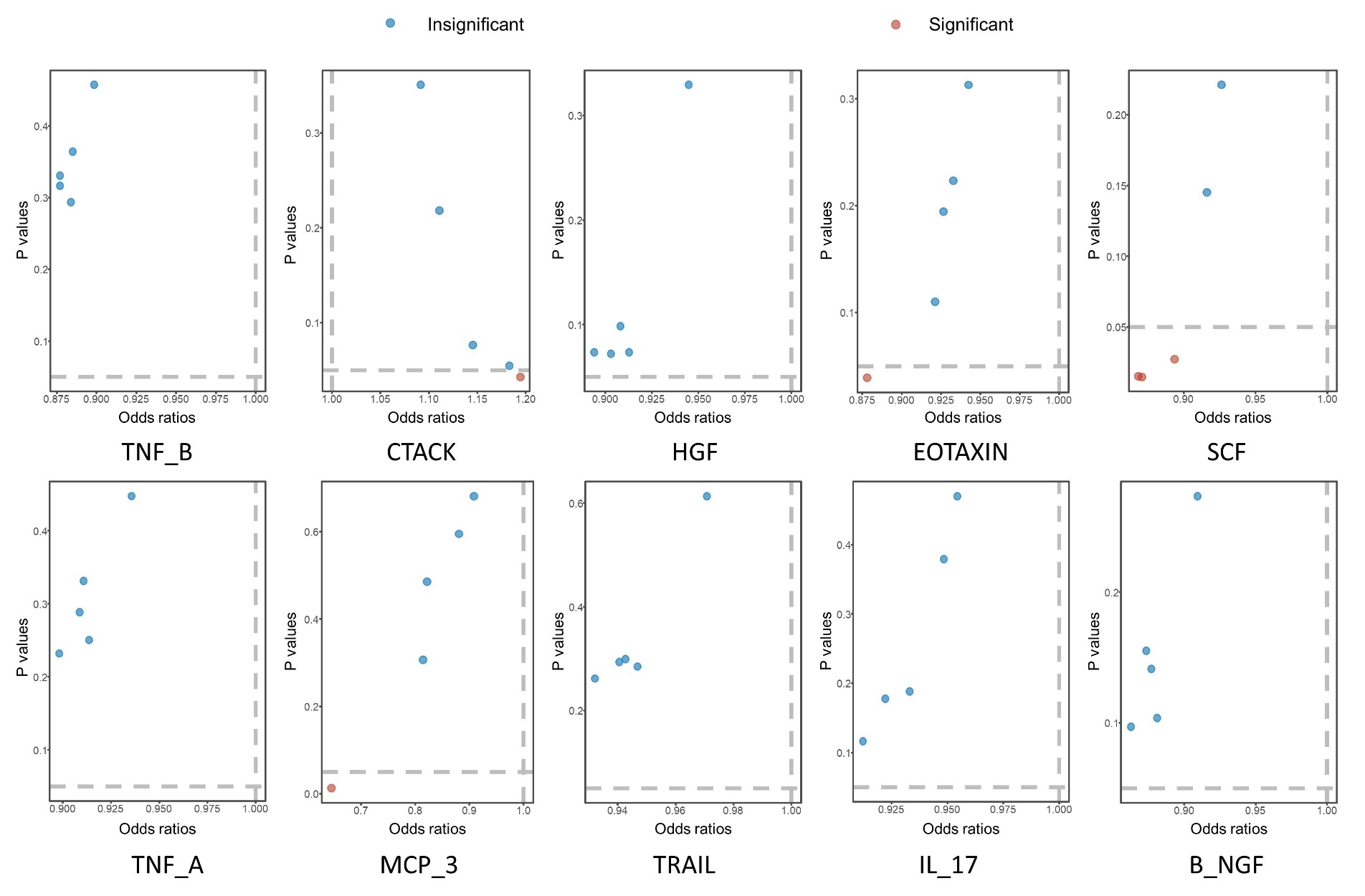


**Figure S40** The leave-one-out analysis for causal association between SAH and inflammation cytokines in FinnGen datasets in replicate reverse MR analysis. SAH, subarachnoid hemorrhage; MR, mendelian randomization.


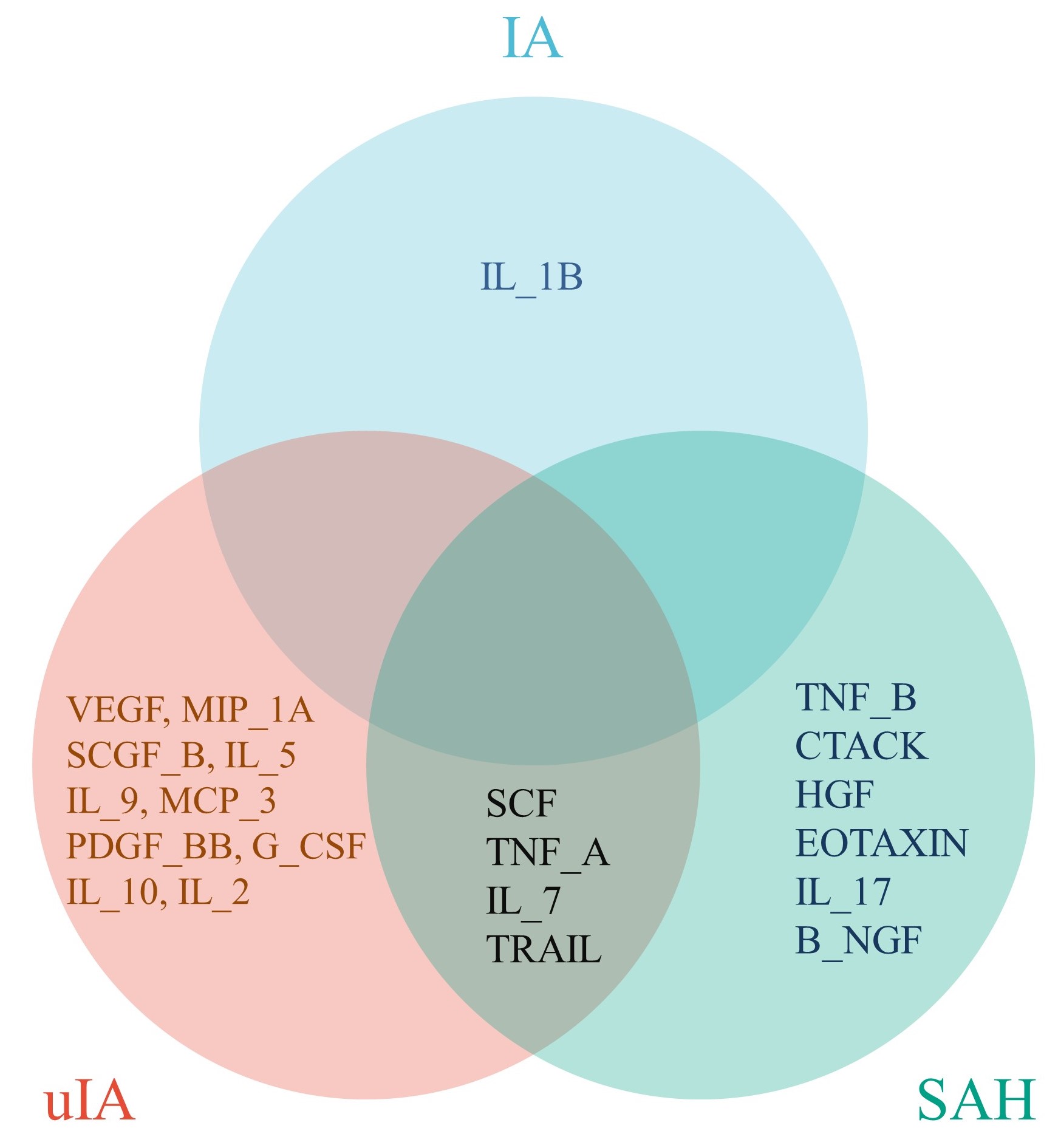


**Figure S41** The common inflammation cytokines among IA, uIA, and SAH in FinnGen datasets in replicate reverse MR analysis. IA, intracranial aneurysm; uIA, unruptured intracranial aneurysm; SAH, subarachnoid hemorrhage; MR, mendelian randomization.


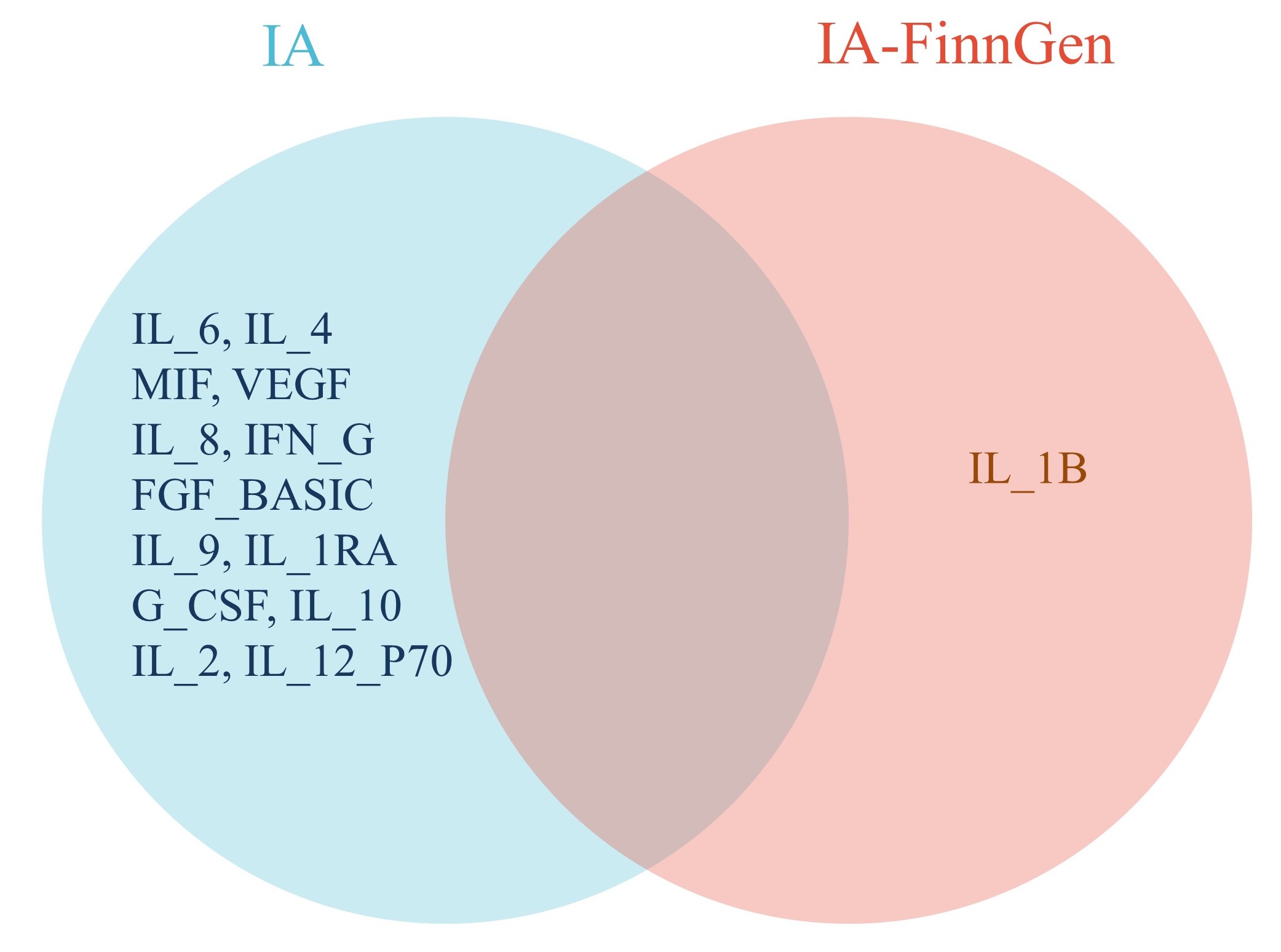


**Figure S42** The common inflammation cytokines for IA between reverse MR and replicate reverse MR analysis. IA, intracranial aneurysm; MR, mendelian randomization.


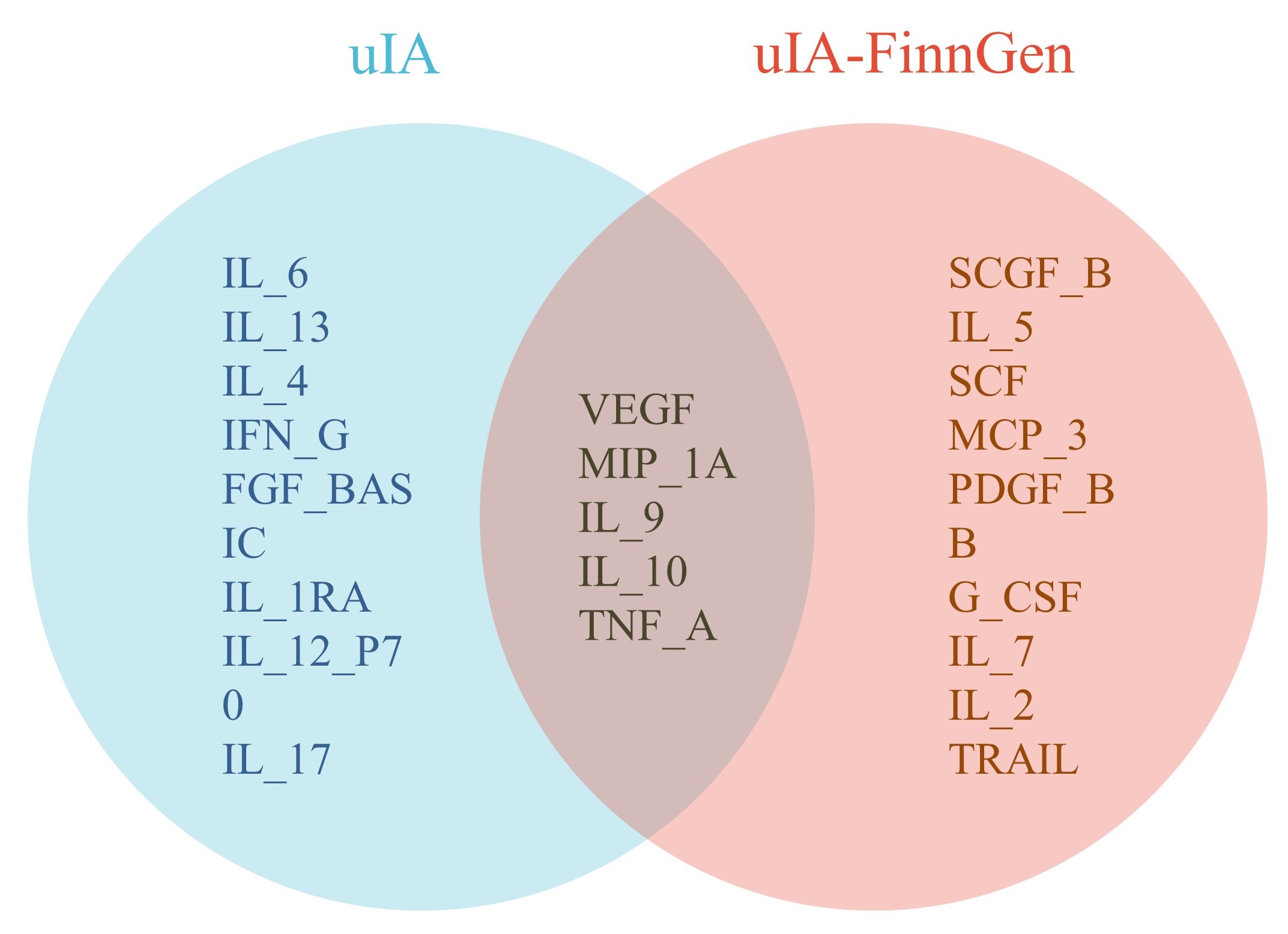


**Figure S43** The common inflammation cytokines for uIA between reverse MR and replicate reverse MR analysis. uIA, unruptured intracranial aneurysm; MR, mendelian randomization.


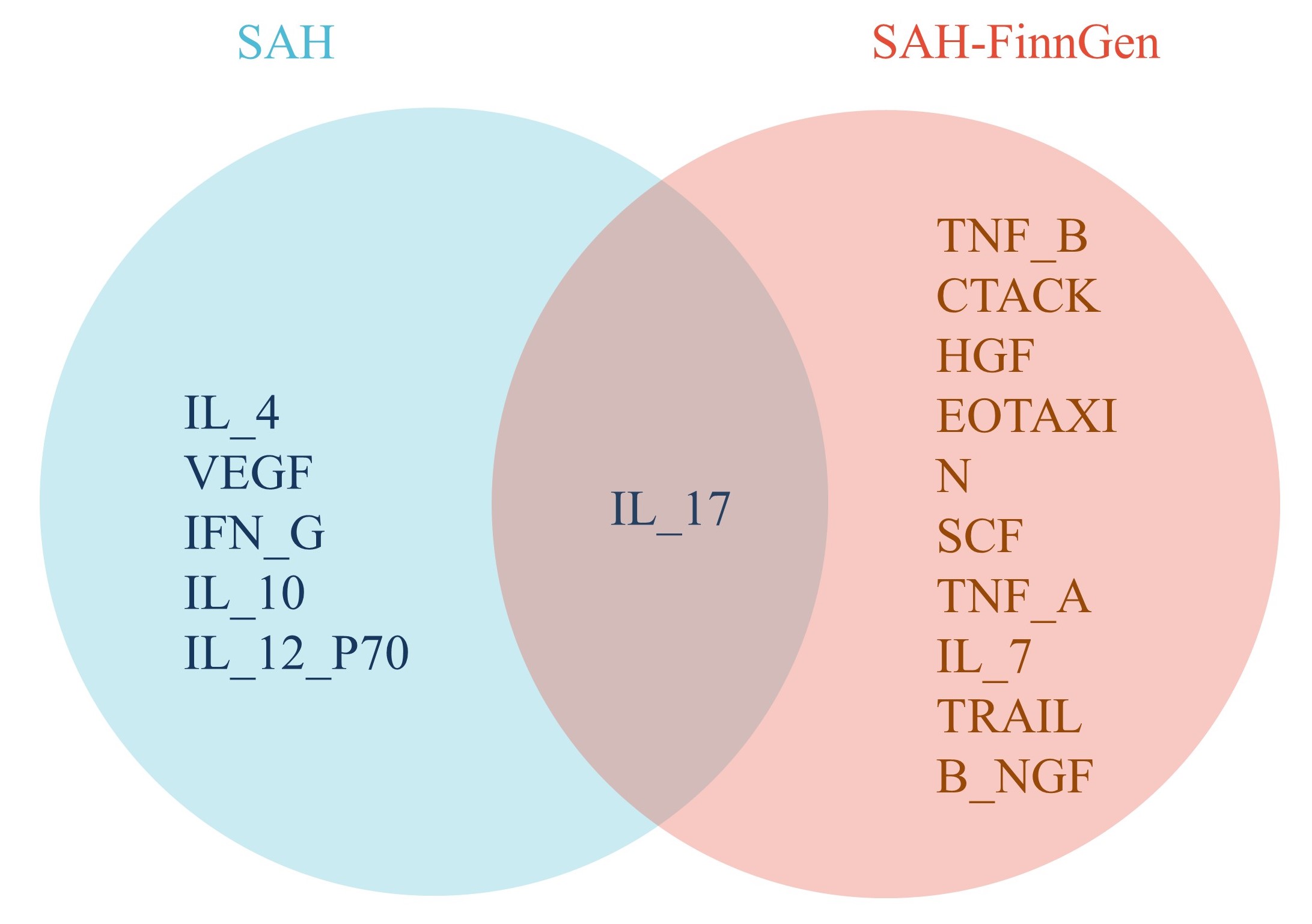


**Figure S44** The common inflammation cytokines for SAH between reverse MR and replicate reverse MR analysis. SAH, subarachnoid hemorrhage; MR, mendelian randomization.

**References**

1. Skrivankova VW, Richmond RC, Woolf BAR, Yarmolinsky J, Davies NM, Swanson SA, VanderWeele TJ, Higgins JPT, Timpson NJ, Dimou N, et al. Strengthening the Reporting of Observational Studies in Epidemiology Using Mendelian Randomization: The STROBE-MR Statement. *JAMA*. 2021;326:1614-1621. doi: 10.1001/jama.2021.18236

2. Skrivankova VW, Richmond RC, Woolf BAR, Davies NM, Swanson SA, VanderWeele TJ, Timpson NJ, Higgins JPT, Dimou N, Langenberg C, et al. Strengthening the reporting of observational studies in epidemiology using mendelian randomisation (STROBE-MR): explanation and elaboration. *BMJ*. 2021;375:n2233. doi: 10.1136/bmj.n2233

3. Bowden J, Davey Smith G, Burgess S. Mendelian randomization with invalid instruments: effect estimation and bias detection through Egger regression. *Int J Epidemiol*. 2015;44:512-525. doi: 10.1093/ije/dyv080

4. Bowden J, Davey Smith G, Haycock PC, Burgess S. Consistent Estimation in Mendelian Randomization with Some Invalid Instruments Using a Weighted Median Estimator. *Genet Epidemiol*. 2016;40:304-314. doi: 10.1002/gepi.21965

5. Hartwig FP, Davey Smith G, Bowden J. Robust inference in summary data Mendelian randomization via the zero modal pleiotropy assumption. *Int J Epidemiol*. 2017;46:1985-1998. doi: 10.1093/ije/dyx102

6. Pierce BL, Burgess S. Efficient design for Mendelian randomization studies: subsample and 2-sample instrumental variable estimators. *Am J Epidemiol*. 2013;178:1177-1184. doi: 10.1093/aje/kwt084

7. Verbanck M, Chen CY, Neale B, Do R. Detection of widespread horizontal pleiotropy in causal relationships inferred from Mendelian randomization between complex traits and diseases. *Nat Genet*. 2018;50:693-698. doi: 10.1038/s41588-018-0099-7

8. Zhao Q, Chen Y, Wang J, Small DS. Powerful three-sample genome-wide design and robust statistical inference in summary-data Mendelian randomization. *Int J Epidemiol*. 2019;48:1478-1492. doi: 10.1093/ije/dyz142
